# xRead: a coverage-guided approach for scalable construction of read overlapping graph

**DOI:** 10.1093/gigascience/giaf007

**Published:** 2025-02-17

**Authors:** Tangchao Kong, Yadong Wang, Bo Liu

**Affiliations:** Center for Bioinformatics, Faculty of Computing, Harbin Institute of Technology, Harbin, Heilongjiang 150001, China; Key Laboratory of Biological Bigdata, Ministry of Education, Harbin Institute of Technology, Harbin, Heilongjiang 150001, China; Center for Bioinformatics, Faculty of Computing, Harbin Institute of Technology, Harbin, Heilongjiang 150001, China; Key Laboratory of Biological Bigdata, Ministry of Education, Harbin Institute of Technology, Harbin, Heilongjiang 150001, China; Center for Bioinformatics, Faculty of Computing, Harbin Institute of Technology, Harbin, Heilongjiang 150001, China; Key Laboratory of Biological Bigdata, Ministry of Education, Harbin Institute of Technology, Harbin, Heilongjiang 150001, China

**Keywords:** read overlapping graph, *de novo* assembly, long-read sequencing, long-read alignment

## Abstract

**Background:**

The development of long-read sequencing is promising for the high-quality and comprehensive *de novo* assembly for various species around the world. However, it is still challenging for assemblers to handle thousands of genomes, tens of gigabase-level assembly sizes, and terabase-level datasets efficiently, which is a bottleneck to large-scale *de novo* sequencing studies. A major cause is the read overlapping graph construction that state-of-the-art tools usually have to cost terabyte-level RAM space and tens of days for large genomes. Such lower performance and scalability are not suited to handle the numerous samples being sequenced.

**Findings:**

Herein, we propose xRead, a novel iterative overlapping graph construction approach that achieves high performance, scalability, and yield simultaneously. Under the guidance of its coverage-based model, xRead converts read-overlapping to heuristic read-mapping and incremental graph construction tasks with highly controllable RAM space and faster speed. It enables the processing of very large datasets (such as the 1.28 Tb *Ambystoma mexicanum* dataset) with less than 64 GB RAM and obviously lower time costs. Moreover, benchmarks suggest that it can produce highly accurate and well-connected overlapping graphs, which are also supportive of various kinds of downstream assembly strategies.

**Conclusions:**

xRead is able to break through the major bottleneck to graph construction and lays a new foundation for *de novo* assembly. This tool is suited to handle a large number of datasets from large genomes and may play important roles in many *de novo* sequencing studies.

## Introduction

*De novo* assembly is to reconstruct a donor genome sequence from reads without reference, which is fundamental to genomics studies. The rapid advances in long-read sequencing technologies, such as single-molecule real-time (SMRT) sequencing [1] and nanopore sequencing [2], have been able to produce reads having >10 kbp median length and >100 kbp maximum length [3]. They have superior repeat-spanning ability to resolve complex repetitive regions, which greatly helps to achieve high-quality assemblies such as telomere-to-telomere [4] and haplotype assembly [5]. However, the assembly of large genomes is still nontrivial (such as *Pinus taeda* [6], *Ambystoma mexicanum* [7], *Euphausia superba* [8], etc.). One of the bottlenecks is the computation intensity (i.e., most of the state-of-the-art assemblers require terabytes of RAM space and cost thousands of CPU hours for such tasks) [9, 10]. In this situation, employed tools are not scalable enough to handle many large genomes with commonly used computational environments since the costs of time and internal memory are prohibitive. Thus, it becomes a major bottleneck to large-scale *de novo* sequencing studies like the Vertebrate Genomes Project [11] and Earth Biogenome Project [12].

A primary cause of the bottleneck is the all-against-all read alignments to construct the initial read overlapping graph, which is a fundamental step in the overlap-layout-consensus (OLC) approach. OLC is one of the most commonly used approaches adopted by state-of-the-art long-read–based assemblers [13–18] (and de Bruijn graph–based approaches as well [19–21]). However, in theory, the upper limit of time complexity of all-against-all read alignment can be very high (i.e., ${\mathrm{O}}( {{m}^2{n}^2} )$, where *m* and *n* are the number and length of the reads, respectively). Many efforts have been made to effectively lower this complexity (see below), but the time cost is still non-neglectable in absolute terms and thus still needs further improvements. Meanwhile, the RAM usage is also high, especially due to all the read information being kept in memory. This is usually a bottleneck for large-scale studies due to the lack of computers with high RAM configuration. Moreover, the crosstalk of genome repeats and sequencing noise also affect the quality of the graph.

State-of-the-art tools use various heuristics to reduce the use of computational resources while improving the yield of read overlapping. Seed-and-extension is one of the most commonly used heuristics. Such approaches retrieve short matches (i.e., seeds) between various reads (usually through indexing data structures) and conduct extended alignments around them to confirm the actual overlapped parts of the reads. The time cost can be significantly reduced with them, as the alignments focus on some pairs of reads being matched whose number is much lower than ${\mathrm{O}}( {{m}^2} )$. However, the real cost depends on the various adopted strategies as well. HGAP [22] is one of the earliest tools tailored to the assembly of noisy long reads. It heuristically indexes a proportion of the longest read and employs a typical seed-and-extension alignment tool (BLASR [23]) to align them with other reads. FALCON assembler [14] employs DALIGNER [24], which partitions reads into blocks and uses sorted *k*-mers within them as the index; further, the blocks are merged to discover read overlaps. Wtdbg2 [17] indexes a quarter of *k*-mers as seeds and takes each tiling 256-bp subsequence as a bin for each read. Further, it employs 256-bp bin-based dynamic programming for extension instead of base-level alignment. Flye [25] collects frequent *k*-mers in reads as seeds and estimates the overlaps by finding the longest common subpath with a fast dynamic programming algorithm. Shasta [18] randomly selects *k*-mers (seeds) to find candidate overlaps with the LowHash algorithm and performs a tailored marker alignment approach for extension. Minimap2 [26] is a minimizer-based generic aligner suited to find the overlaps of long reads in various lengths and error rates, which is also employed by several state-of-the-art assemblers such as Raven [27], PECAT [28], and Nextdenovo [29]. It essentially uses minimizer-based [30, 31] seeding and chaining to detect read overlaps and also supports the base-level alignment of anchored reads if necessary. Similar to Minimap2, Hifiasm [32] uses a minimizer-based approach tailored for HiFi reads to implement read overlap alignment as the initial step and further achieve haplotype-resolved assembly. BLEND [33] uses SimHash [34, 35] to generate the same hash value for both identical and similar *k*-mers (seeds) to find fuzzy matches and detect read overlaps.

Some of the previous studies also focus on the acceleration of the base-level alignment to reduce the cost of extension. Most of them take advantage of single instruction multiple data (SIMD) instructions, such as Intel AVX instructions or Compute Unified Device Architecture (CUDA) in Nvidia GPU. Manavski and Valle [36] proposed an implementation of the Smith–Waterman algorithm under the CUDA framework. Libssa [37] uses AVX2 instructions to accelerate the classical Smith–Waterman and Needleman–Wunsch algorithms. Parasail [38] is a SIMD-based implementation of global, semi-global, and local alignments that supports a couple of instruction sets such as SSE2, SSE4.1, AVX2, AltiVec, and NEON. Suzuki and Kasahara [39] developed a fast SIMD-based alignment algorithm named libgaba, and it was further improved in KSW2 [26] and employed by Minimap2.

The overall cost of seed-and-extension approaches is still high due to many issues such as the large number of reads, high sequencing errors, and ubiquitous repeats. Alignment-free approaches are also proposed. Most of them use compact sequence representations (usually termed sketches [40]) to directly measure read similarities. MHAP [13] uses the MinHash technique, which employs 256 to 1,512 hash functions to construct sketches and use them to estimate the Jaccard similarity of the reads. Canu [15] uses adaptive *k*-mer weighting to improve MinHash, which reduces the effect of repetitive *k*-mers. MECAT [16] splits reads into blocks and finds candidate overlaps with at least 1 matched block. Low-similarity overlaps are then filtered based on distance difference factor (DDF) scores. NECAT [41] extends DDF scoring by sorting all *k*-mer pairs and chaining them together to remove false-positive *k*-mers. This is more suited to the sequencing errors of ONT reads. Such approaches avoid full alignment, but their computational cost is also non-neglectable since many query and merging operations are usually needed to make a number of sketches to achieve high sensitivity.

More efficient and scalable long-read assembly approaches are in wide demand to deal with the ever-increasing sizes and numbers of *de novo* sequencing genomes. Moreover, there are still a number of false positives/negatives in overlapping graphs caused by the various trade-offs on sensitivity, precision, and performance. Herein, we propose xRead, an incremental overlapping graph construction approach that enables one to achieve high scalability, performance, and yields simultaneously. Guided by a novel read coverage–based objective function, xRead iteratively builds the overlapping graph with heuristic read indexing and lightweight alignment skeletons. The approach has 3 major contributions to break through the bottleneck to the high-performance genome assembly. First, it has outstanding scalability for memory usage, which enables one to build the overlapping graphs for the datasets of large genomes with low and controllable RAM space cost. For example, it can build an overlapping graph for the 32× PacBio sequencing dataset (1.9 Terabyte) of the Axolotl genome with 64 GB or lower RAM. Second, it has high speed for various-sized genomes (e.g., several times faster on average than that of Minimap2 on the long-read datasets from small bacteria to large mammal genomes). Third, it is able to achieve high precision and connectivity in graph construction.

## Findings

### Overview of the xRead approach

**Figure 1:**
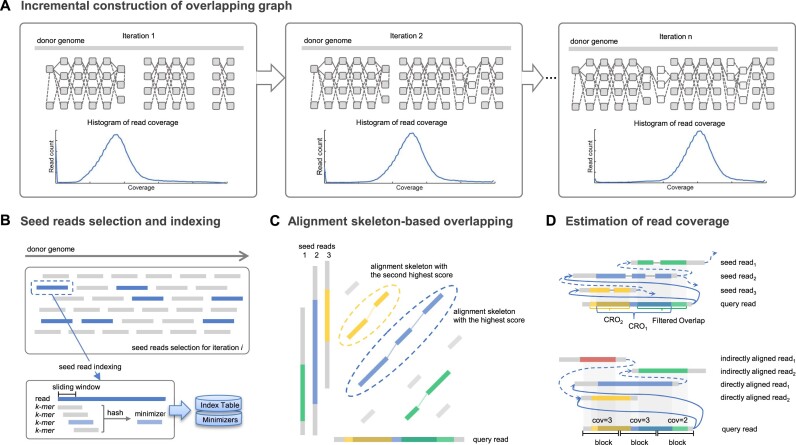
A schematic illustration of xRead. (A) The incremental construction of the overlapping graph. The subplots represent the connections among reads being incrementally recovered within various iterations. The gray blocks indicate the sequencing reads. The dashed and solid lines respectively indicate the overlaps between the seed and the query reads, as well as the seed reads themselves. The histograms in the lower part indicate the distributions of read coverage, which are updated in various iterations. (B) The selection and indexing of seed reads. xRead selects a portion of lowly covered reads as seed reads (marked as blue bars), and a list of minimizers is extracted using a hash function. A minimizer-based index is then built by a hash table-based data structure. Meanwhile, the same hash function is also used to generate minimizers for query reads. (C) Alignment skeleton-based read overlapping. For a given query read, xRead finds the MBs (marked as colored bars) between it and all the seed reads via the index. Further, it uses the SDP approach to generate 1 or more alignment skeletons (the dashed ovals indicate the skeletons with the first and second highest scores). (D) Estimation of read coverage. The upper subplot represents the selection of CROs. The first 2 highest-scored skeletons are selected as CROs, which bring new edges (represented by solid lines) to the overlapping graph. Other skeletons with lower scores are filtered out. The lower subplot represents the (re)estimation of read coverage. xRead splits the reads into nonoverlapping blocks and then counts the reads directly (marked as solid lines) and indirectly (marked as dashed lines) being aligned to it.

Unlike state-of-the-art overlapping tools that in essence push each of the reads to explore all the other ones with heuristics, xRead adopts a “mimicking-and-mapping” design. Mainly, it is motivated by that each read can be seen as a representative of some part of the donor genome. A selected set of seed reads covering the whole genome can mimic a “virtual reference” to reveal read overlaps through a read-to-reference mapping, that is, the reads from the same region can be implicitly aligned to the corresponding seed read(s) in the virtual reference. So, all the overlaps between seed and nonseed reads can be detected and the various parts of the genome can also be connected by such alignments. Guided by a novel coverage-based strategy of seed read selection (“the completeness of virtual reference,” see below), xRead constructs the overlapping graph in an iterative process (Fig. 1). In each iteration, it implements graph construction and refinement in 3 major steps as follows (also refer to the Methods section for more detailed information).

Step 1: xRead selects a proportion of reads with relatively low coverage and high length as seed reads and builds a partial read index for them.
Step 2: xRead employs a lightweight alignment skeleton approach to discover new read overlaps between the seed reads and other less covered reads (also termed as query reads).
Step 3: xRead constructs/refines the overlapping graph based on the produced alignment skeletons. Further, it (re)estimates the read coverage and stops the process if most reads have high enough coverage; otherwise, it turns to step 1 for a new iteration.

This approach lays a new foundation for genome assembly in 2 aspects. First, it is apparent that the cost can be substantially reduced by converting all-against-all read alignment to a read-to-reference mapping task. Second, the produced graph can also be correctly connected (i.e., achieve high precision and connectivity simultaneously), which is supportive to both of the popular strategies in long-read assembly (i.e., correction-then-assembly and assembly-then-correction; also refer to the Discussion section for a more detailed explanation). These are done by some tailored implementations for several critical issues as follows.

1. The completeness of virtual reference. To achieve the best performance, it is fundamental to select a set of seed reads covering the whole genome and having as few as possible redundant reads, but nontrivial. xRead achieves this goal by using a fact of the read mapping. That is, given an arbitrary set of seed reads, relatively high coverage (i.e., times of being aligned) can be derived for all the reads belonging to the virtual-reference covered regions, which is done by exploring the produced alignments and their transitive relationships. Meanwhile, the reads from uncovered regions have much lower coverage. Thus, the coverage becomes a useful indicator to iteratively select the reads from those still uncovered regions, and the whole genome can be implicitly and progressively explored. This assumption could be oversimplified for the reads from ultra-long repeats, but it does not affect the overlapping task much (refer to Discussion section for more details).
2. The mappability of the virtual reference. A high mappability of the virtual reference (seed reads) is useful to achieve confident read mapping. Thanks to the randomness and less bias of long-read sequencing [42–44], there is no obvious correlation between the read lengths and positions. Thus, with the arbitrary selection assumption mentioned above, it is feasible to straightforwardly select the longest ones from the remaining reads to maximize the mappability of the virtual reference.
3. The correctness of read overlapping. xRead is more precision-oriented in the precision–sensitivity trade-off during overlapping detection. The virtual reference helps to reduce false-positive overlaps systematically with its high mappability. Moreover, xRead also employs a conservative alignment-scoring approach to construct an accurate overlapping graph. Following this strategy, xRead only keeps the most confident read overlaps (CROs; see Methods) with high alignment scores. Motivated by that successful assembly, which may come from a correctly connected graph, the high-precision design of xRead is useful, especially with good graph connectivity (see below).
4. The connectivity of the produced graph. Although not implementing a comprehensive overlap directly, xRead also produces highly connected graphs. The key point is the completeness of the virtual reference. From a donor genome point of view, the seed reads are ubiquitously placed, and no large gap exists between 2 nearby seed reads. Thus, each non–seed read can be aligned (i.e., connected) to 1 or more seed reads, and then the non–seed reads aligned to the same seed read compose a connected component. Further, these components can be connected comprehensively since any pair of nearby seed reads can be either directly aligned (if they are overlapped) or connected via 1 or more non–seed reads having overlaps to both of them.
5. The optimization of performance. xRead optimizes speed and RAM usage by several tailored implementations to achieve outstanding performance and scalability. One is the use of the lightweight alignment skeleton (step 2) to speed up each iteration, referring to previous studies [45–47]. Another is that, followed by the coverage-guided overlapping strategy, the batch size of the seed reads is highly tunable so that the whole process can be run with controllable RAM usage.

### The datasets used in benchmarks

We simulated 17 datasets by PBSIM [48, 49] and also employed 7 real datasets (Table 1) for the benchmark of read overlapping and genome assembly. The datasets are from 9 genomes (Supplementary Table S1) having small (<1 Gbp, i.e., *Escherichia coli, Saccharomyces cerevisiae, Caenorhabditis elegans, Arabidopsis thaliana*, and *Drosophila melanogaster*), large (>1 Gbp, i.e., *Zea mays, Mus musculus*, and *Homo sapiens*), and very large (>10 Gbp, i.e., *A. mexicanum*) sizes. It is also worth noting that we partitioned the chromosomes of the *A. mexicanum* genome in the simulation due to the limit of PBSIM (15 of the chromosomes were divided into 30 <1-Gbp ones in advance).

**Table 1:** Detailed information of simulated and real datasets

No.	Reference genome	Error model/platform	File size	Number of reads	Error rate	Average read length	Coverage
**Simulated ONT datasets with an average accuracy of 87%** ^a^							
1	*Escherichia coli*	R103	443 MB	15,378	13.0%	13,000	50×
2	*Saccharomyces cerevisiae*	R103	1.2 GB	40,562	13.0%	13,000	50×
3	*Caenorhabditis elegans*	R103	9.4 GB	334,603	13.0%	13,000	50×
4	*Arabidopsis thaliana*	R103	12 GB	398,748	13.0%	13,000	50×
5	*Drosophila melanogaster*	R103	14 GB	561,293	13.0%	13,000	50×
6	*Zea mays (SK)*	R103	202 GB	7,724,943	13.0%	13,000	50×
7	*Mus musculus*	R103	255 GB	9,376,380	13.0%	13,000	50×
8	*Homo sapiens*	R103	291 GB	12,215,872	13.0%	13,000	50×
9	*Ambystoma mexicanum*	R103	2 TB	95,447,945	13.0%	13,000	50x
**Simulated ONT datasets with an average accuracy of 94%** ^b^							
10	*Escherichia coli*	QSHMM-ONT-HQ	443 MB	15,613	6.0%	15,000	50x
11	*Arabidopsis thaliana*	QSHMM-ONT-HQ	12 GB	402,487	6.0%	15,000	50×
12	*Drosophila melanogaster*	QSHMM-ONT-HQ	14 GB	561,999	6.0%	15,000	50×
13	*Homo sapiens*	QSHMM-ONT-HQ	291 GB	10,475,665	6.0%	15,000	50×
**Simulated HiFi datasets with an average accuracy >99.5%** ^c^							
14	*Escherichia coli*	—	443 MB	13,939	<0.5%	16,606	50×
15	*Arabidopsis thaliana*	—	12 GB	360,265	<0.5%	16,606	50×
16	*Drosophila melanogaster*	—	13 GB	414,157	<0.5%	16,606	50×
17	*Homo sapiens*	—	291 GB	9,385,394	<0.5%	16,606	50×
**Real datasets**							
18	*Escherichia coli*	ONT R9.3	775 MB	34,389	—	11,766	86×
19	*Caenorhabditis elegans*	ONT R9.3	21 GB	789,871	—	13,724	108×
20	*Drosophila melanogaster*	ONT R9.3	14 GB	640,215	—	11,142	50×
21	*Homo sapiens*	ONT R9.3	162 GB	10,836,442	—	7,903	28×
22	*Homo sapiens*	ONT R10.4	419 GB	18,125,024	—	12,360	80×
23	*Homo sapiens*	PacBio HiFi	166 GB	6,596,012	—	13,478	28×
24	*Ambystoma mexicanum*	PacBio RS II	1.9 TB	105,847,426	—	9,576	32×

a The datasets were simulated by PBSIM2 using the pretrained R103 chemistry model based on the reference genomes in Table 1. The read depth, ratio of sequencing errors (mismatches: insertions: deletions), mean length, and total error rate were configured as 50, 23:31:46, 13,000, and 13%, respectively.

b Four datasets were simulated by PBSIM3 using the provided QSHMM-ONT-HQ quality score model. The read depth, ratio of sequencing errors (mismatches: insertions: deletions), mean length, and total error rate were configured as 50, 39:24:36, 15,000, and 6%, respectively.

c Four PacBio HiFi datasets were simulated using PBSIM3 sampling-based simulation. The read length and quality score of simulated datasets are sampled from a real HiFi dataset with mean read length and accuracy of 16,606 and 99.6%, respectively.

To assess the performance of xRead in various error rates, we respectively simulated 9 low-quality, 4 high-quality ONT-like, and 4 HiFi-like datasets (50× coverage each) as follows.

1. The 9 low-quality ONT datasets were simulated for all 9 genomes with the pretrained R103 chemistry model of PBSIM. The mean read length and total error rate were 13 kbp and 13%, respectively, referring to a previous study [3]. These datasets are highly noisy like the fast base-calling mode of ONT platforms and employed to assess the robustness of xRead to sequencing errors for various-sized genomes.
2. The 4 high-quality ONT datasets were also simulated from 4 genomes (*E. coli, A. thaliana, D. melanogaster*, and *H. sapiens*). The QSHMM-ONT-HQ model was used (the mean read length and average accuracy were 15 kbp and 94%, respectively) to mimic the data produced by mostly used ONT platforms (especially for its HAC mode).
3. The 4 HiFi-like datasets were produced by sample-based simulation of PBSIM with a real sequencing dataset (mean read length: 16.6 kbp and average base accuracy: 99.6%) from 4 genomes (*E. coli, A. thaliana, D. melanogaster*, and *H. sapiens*). These datasets mimic the data produced by currently used PacBio platforms.

Moreover, the 7 real datasets are as follows. Three of them are from smaller genomes (*E. coli, C. elegans*, and *D. melanogaster*) and produced by ONT platforms. Three are real human datasets from the well-studied GIAB sample HG002 (NA24385); 2 are ONT datasets in fast and super-high-accuracy base-calling modes, respectively; and the other one is a PacBio HiFi dataset. The ONT super-high-accuracy and PacBio HiFi datasets were employed to assess the ability of xRead on high-quality long-read datasets. Furthermore, a PacBio CLR dataset from the *A. mexicanum* genome was also used to assess the ability of the tools on very large genomes.

### Simulation benchmark for read overlapping

**Figure 2:**
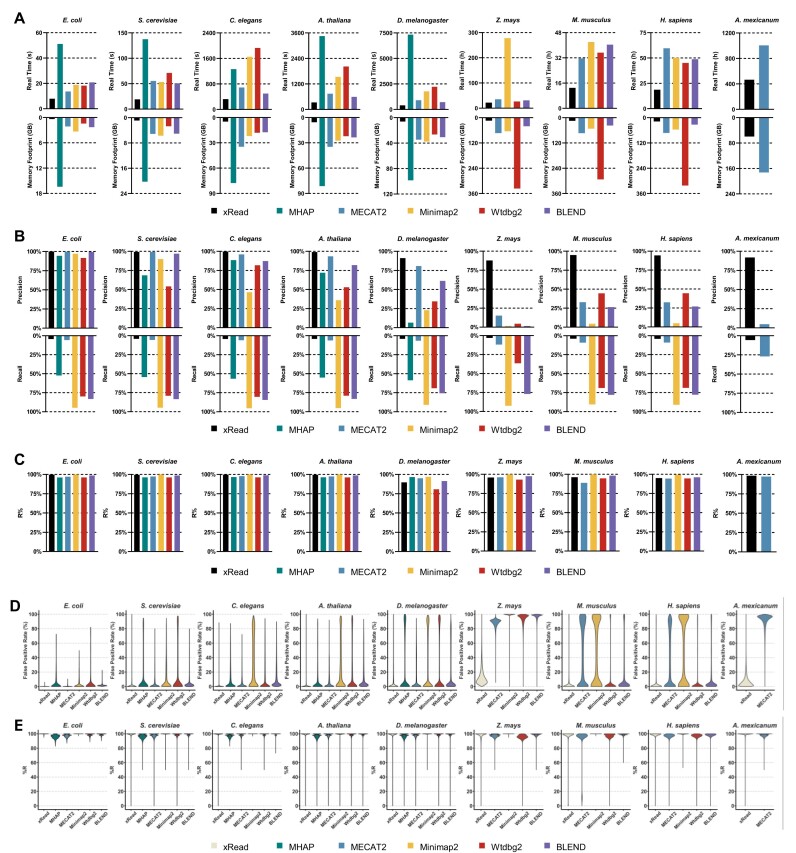
Results on simulated datasets. The figure depicts the real-time, peak memory (A), precision and sensitivity (B), R% (C), and violin plots of FPR (D) and R% (E) of each overlapper on 9 simulated datasets of various-sized genomes (from *E. coli* to *A. mexicanum*). (A–C) In each subplot of (A), (B), and (C), the colors black, green, blue, orange, and red respectively represent the overlapper xRead, MHAP, MECAT2, Minimap2, wtdbg2, and BLEND. (D, E) The violin plot of FPR and R% for various datasets. For a given tool, the absence of the results for some datasets is due to its failure during benchmarking. Refer to supplementary material for detailed information on datasets and results.

We implemented benchmarks on the 9 low-quality and 4 high-quality ONT-like simulated datasets to assess the baseline performance of xRead. Five state-of-the-art tools (MHAP, MECAT2, Minimap2, wtdbg2, and BLEND) were employed for comparison. Their runtimes, memory footprints, precisions, and sensitivities are in Fig. 2 (refer to Supplementary Tables S3–S4 for numerical information). Mainly, 3 key points were observed from the results as follows.

#### xRead has outstanding scalability for various-sized genomes

xRead allows controllable RAM usage, and we limited it to 16 GB, 24 GB, and 64 GB for the datasets of small, large, and very large genomes, respectively. A larger RAM space is used for *A. mexicanum* to reduce Input/Output (I/O) operations, although it is still much lower than that of comparing tools. xRead accomplishes all the tasks successfully (Fig. 2A and Supplementary Table S3), suggesting that the coverage-guided design is able to well-handle large datasets with medium-sized workstations. Meanwhile, the speed of xRead is about 1.2 to 18 times (4 times on average) faster in real time than that of other tools for the low-quality datasets and about 2 to 29 times (8.5 times on average) for the high-quality datasets, respectively. Overall, the performance suggests that xRead has good scalability to various-sized genomes.

Other tools were run without any limitation on RAM space, and their memory footprints are 3 to 47 times higher than that of xRead with relatively lower speed on all simulated datasets. This could be caused by the following 2 kinds of read overlapping strategies. One is to load all the reads and process them in memory, like MHAP and wtdbg2, whose RAM usage is quite high. The other one is to divide the whole datasets into batches of reads and separately handle each of them, like MECAT2, Minimap2, and BLEND. For each batch, the involved reads are indexed in time, and other reads are aligned to them for overlaps. This is more similar to xRead, but they also required significantly higher RAM space (e.g., MECAT2 used over 172 GB RAM to process the *A. mexicanum* dataset with 64 CPU threads). Moreover, the lower speed of MECAT2, Minimap2, and BLEND could be also due to their own designs (i.e., they straightforwardly divide the datasets and align all the reads to a specific batch of reads in each iteration). It is also worth noting that only xRead and MECAT2 finished the *A. mexicanum* task, and the real time of xRead is 60% off compared to that of MECAT2, which saved about 25 days. For this dataset, MHAP and wtdbg2 ran out of memory (over 1 Terabyte). Minimap2 and BLEND showed even lower speeds—that is, Minimap2 cost about 178 hours (with 64 CPU threads) to process only 11 read batches (about 3.29% of the dataset), and BLEND cost about 175 hours to process 28 read batches (about 8.38% of the dataset). Considering the high time cost (estimated over 225 and 87 days, respectively), we early stopped the programs.

To further investigate the effect of RAM usage on the speed of xRead, we evaluated 4 other configurations (2 GB, 8 GB, 16 GB, and 32 GB) using 4 low-quality simulated datasets and 4 real datasets (*E. coli, D. melanogaster, M. musculus*, and *H. sapiens*; Supplementary Fig. S1A). The results showed that xRead still kept relatively high speeds even with very low RAM usage (e.g., 2 GB), and the runtime gain lowered with larger RAM space (e.g., 32 GB), suggesting that a small RAM space is enough for xRead. Mainly, the time–space trade-off comes from the number of reads being indexed. That is, xRead has to load fewer reads at one time with less RAM, and this leads to not only more iterations but also a higher number of index-query operations in each iteration due to more matching-failures happening during the generation of the alignment skeletons.

We also assessed the usage of external memory. xRead showed a 50% to 99% off on external memory compared to other tools (Supplementary Fig. S1B and Supplementary Table S10). It is also worth noting that xRead showed superior scalability with large-scale datasets. For example, xRead only required 9.2 GB of external memory on the 2-TB simulated *A. mexicanum* dataset, while MECAT2 consumed 869 GB. This advantage comes from that xRead only records the confident overlaps with high scores, but it also avoids transitive overlaps between nonseed reads, which reduces redundancy.

#### xRead produces accurate read overlaps and has the potential to achieve high sensitivity

We evaluated the overall precisions and sensitivities of the tools (Fig. 2B and Supplementary Table S4, Precision and Sensitivity columns; also refer to the Methods section for more details about assessment, including the definitions of ground truth, true-positive and false-positive overlaps, and the computation of precision and sensitivity). The results of the tools varied, indicating their different strategies and trade-offs. With the relatively conservative CRO strategy, xRead outputs a set of “core overlaps,” which connect seed reads and all the other reads with high scores, achieving the highest precisions on all the datasets. MHAP, MECAT2, and wtdbg2 are more likely to pursue the balance between precision and sensitivity, so that their sensitivities are higher than that of xRead, but precisions are lowered. Minimap2 and BLEND try to recover read overlaps comprehensively. Minimap2 outputs the highest number of read overlaps and achieves the highest overall sensitivities on all the datasets, while BLEND achieves higher precision at the cost of the sensitivities, possibly due to its ability to find both fuzzy and exact seed matches. However, the precision of Minimap2 is lowest (i.e., there are lots of false positives in the graphs, not only for the large but also the relatively small genomes, such as *A. thaliana* and *D. melanogaster*).

We further investigated more detailed information of the produced graphs with 3 additional metrics, R%, C%, and Con. Num. The results indicate that the xRead graphs are connective and also have the potential to achieve high sensitivity through additional transitive operations if required.

R% indicates the proportion of the reads having at least 1 ground-truth overlap being recovered. This metric indicates the proportion of the reads meeting the least requirement to correctly connect to the graph. The result (Fig. 2C and Supplementary Table S4, R% column) suggests that Minimap2 has the highest R% due to its sensitivity. For other tools, their R% is also very high and comparable to that of Minimap2, indicating that most of the reads have 1 or more correct overlaps being recovered. For xRead, this indicates that most of the reads can be correctly aligned to seed reads. Therefore, it also holds the possibility of comprehensive graphs with postprocessing. Moreover, for all the tools, R% is improved on high-quality datasets, suggesting that the true-positive overlaps are more distinguishable from those false positives with the improved data quality.

C% indicates the percentage of the donor genome being covered by the connected reads. Herein, connected reads indicate the reads having at least 1 edge (overlap) in the produced graph. This metric reflects the coverage of the graph to the donor genome, which is fundamental to complete assembly. It was observed that nearly all the tools achieved very high C% (close to 100%) for various datasets, indicating that the produced graphs had no or very few uncovered regions (the C% and Gap Num. columns of Supplementary Table S4). For xRead, this result suggests that the seeds and their connected reads can cover the whole genome, which is helpful to fully recover the genome sequence. Only the Gap Num. of BLEND becomes obviously higher on low-quality datasets, indicating that it could not handle low-quality reads in some genomic regions.

Con. Num. indicates the number of connected components in produced graphs, which measures their connectivity. It was observed (Con. Num. column of Supplementary Table S4) that on various datasets, nearly all the produced graphs had relatively few connected components. This is partially due to the superior read lengths, but it also indicates that tools enable the effective alignment and connection of the reads. Moreover, the Con. Num. of all tools decreased on high-quality datasets, suggesting that more potential connections can be revealed with improved sequencing quality. With the highly connected graphs, all the tools have the chance to indirectly infer all the read overlaps in the same components via transitive relationships and achieve high sensitivity. For xRead, the number of connected components is comparable to that of other tools on various datasets, but it slightly increased for some of the genomes. We investigated the graphs and found that most of the reads were connected and clustered in a few components, and other components only had very few (mostly 2 or 3) reads. This is mainly due to a small proportion of selected seed reads being error-prone, and some of the reads were aligned to them by accident. It usually happened in large datasets, since more outliers having serious sequencing errors occurred with increased read numbers. Other tools also had such a trend, but by filtering out those extremely small ones, all the tools had very similar numbers of connected components.

Considering the precision, coverage, and connectivity, we realized that the graphs produced by xRead are suited to function as a core graph to guide successful read assembly or error correction, referring to previous studies [22]. Furthermore, xRead also provides an additional tool to optionally expand the core graphs to meet the requirements of various genome assembly approaches. Mainly, it is implemented by a transitive rule-based width-first searching approach to iteratively recover the overlaps among nonseed reads (refer to the Methods section for more details). The tool enables one to recover as many overlaps as possible. Meanwhile, it also supports fine-tuning the number of iterations for various trade-offs between sensitivity and precision. The overall sensitivities of the graphs expanded by 1, 3, and 5 iterations are in Supplementary Table S5. It is observed that the sensitivity of the produced graphs greatly improved by even only 1 iteration. Moreover, the number of overlaps saturated after 5 iterations for most of the reads and the sensitivity metrics (overall sensitivity and R%) can be higher than or close to that of Minimap2.

#### xRead keeps high yields along the whole genomes

We assessed the read overlaps in various local regions to track the behaviors of xRead under various genomic contexts. Mainly, the reference genome was split into nonoverlapping blocks (size: 1 kbp and 10 kbp for small and large/very large genomes, respectively), and the reads were assigned to corresponding blocks according to the ground truth. Further, the false-positive overlap rates (FPRs) and R% in various blocks were separately calculated. Herein, for a given block, FPR is defined as the proportion of false positives against all overlaps that at least one of the involved reads belongs to the block, and R% is the proportion of the reads belonging to the block that has at least 1 ground-truth overlap recalled. The violin plots of FPR and R% for various datasets are in Fig. 2D, E.

The FPR plots show that xRead is able to keep relatively low FPR along the whole genome and has a significantly higher number of zero FPR blocks. We further investigated the high FPR blocks of xRead and found that most of them are of complex repetitive regions. Such cases are quite complicated and challenging due to the combination of a high similarity of repeat copies and sequencing noise. An example is the *Z. mays* dataset, where the genome is highly repetitive and all of the tools cannot maintain very low FPR. However, with the relatively conservative CRO strategy, xRead still maintained relatively high precision, that is, for most of the blocks, it had <20% FPR, while other tools produced ubiquitous high FPR (>80%) blocks.

The R% plots show that all of the tools have high R% along the whole genome (i.e., they have the ability to recover true positive overlaps in various regions). For xRead, this derives from the nearly nongap coverage of the whole genome by the seed reads and the high ability of alignment skeletons to capture the nonseed reads from the same local regions. We further investigated those rare low R% blocks of xRead and observed that they also concentrated in highly repetitive regions. This is mainly due to that the reads from such regions, especially the ones having relatively short lengths and lower repeat spanning ability, are more likely to be aligned to the seed reads from other copies of the same repeats and the incorrect CROs mislead the calculation of read coverage. Thus, such reads may have high coverage in the first several iterations of graph construction and lead to a collapsed graph. However, this issue does not affect the connectivity of the graph, and the missing overlaps in the same repeat copy can still be recovered by transitive relationships. Thus, it keeps the chance for further steps (read correction and/or layout) to well-handle them and produce correct assemblies. Also refer to the Discussion section for more detailed explanations.

### Real-data benchmark for read overlapping

The tools were further benchmarked with 7 real datasets from ONT and PacBio platforms (Table 1). The same limitation on RAM space was applied for xRead and no limitation for other tools. The performance, precision, and sensitivity were assessed, and similar trends were observed.

**Figure 3:**
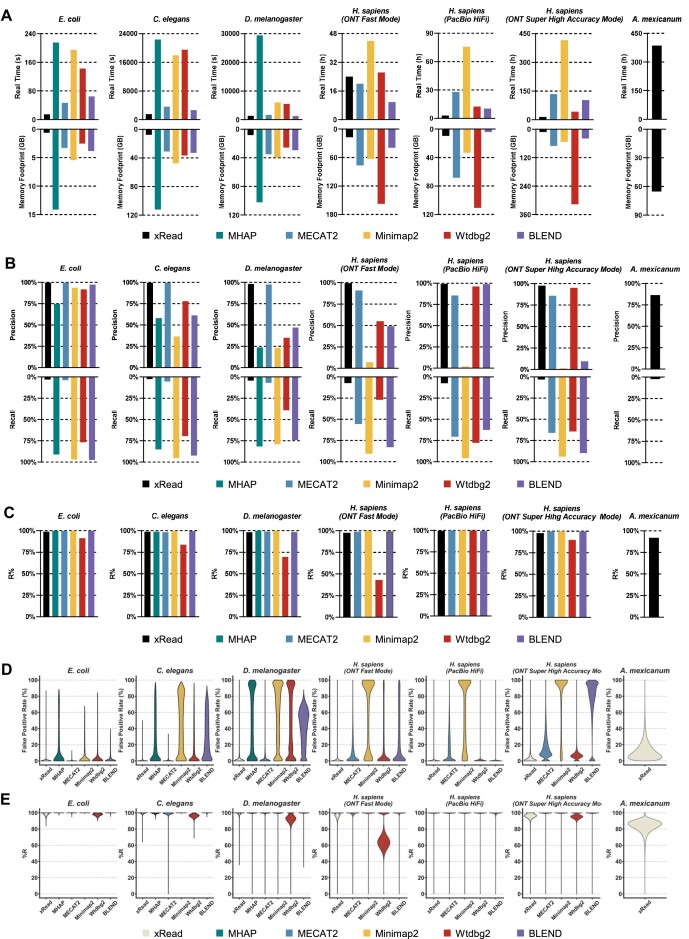
Results on real datasets. The figure depicts the real-time, peak memory (A), precision, sensitivity (B), R% (C), and violin plots of FPR (D) and R% (E) of each overlapper on 7 real datasets of various-sized genomes (*E. coli, C. elegans, D. melanogaster*, 3 *H. sapiens* datasets from different platforms, and *A. mexicanum*). (A–C) In each subplot of (A), (B), and (C), the colors black, green, blue, orange, and red respectively represent the overlapper xRead, MHAP, MECAT2, Minimap2, and wtdbg2, as well as BLEND. (D, E) The violin plot of FPR and R% for various datasets. For a given tool, the absence of the results for some datasets is due to its failure during benchmarking. Refer to supplementary material for detailed information on datasets and results.

xRead still had high performance (Fig. 3A and Supplementary Table S6) (i.e., overall faster speed with lower memory footprints than all of the other tools). It is also worth noting that xRead had higher performance on the ONT super-high-accuracy and PacBio HiFi datasets (several to tens of times faster than other tools), indicating that it is also suited to high-quality long reads. An exception occurred on the ONT fast mode human dataset that the speed of xRead is slower than that of MECAT2 and BLEND. It is mainly caused by the repeats of the human genome (i.e., xRead spent much time on the sorting and linking of the numerous minimizers from highly repetitive regions). Although higher, the time cost is still acceptable in absolute terms. The performance also shows that xRead is more suited to handle very large genomes. It is the only tool that accomplished the real *A. mexicanum* dataset successfully on the employed server. MHAP and wtdbg2 were out of memory (>1 Terabytes). MECAT2 raised an error signal and terminated. The speed of Minimap2 and BLEND was still very low like that of the simulated datasets and was stopped early considering its very high estimated time cost.

The precision of xRead was still higher than that of other tools on the real datasets (Fig. 3B and Supplementary Table S7, Precision column). MECAT2 also achieved similar precision on smaller genomes, but it decreased on the 3 human datasets, potentially due to the characteristics of its DDF score model. The precision of wtdbg2 and BLEND was relatively high on PacBio datasets while significantly lowered on ONT datasets, indicating that they could be not suited to more error-prone reads. The precisions of MHAP and Minimap2 were significantly lower.

The overall sensitivities of the tools still varied due to their different designs, while Minimap2 was the highest one in contrast to its precision. On the R% metric (Fig. 3C and Supplementary Table S7, R% column), the tools were high and close to each other (except for wtdbg2 on ONT datasets), indicating that they potentially had similar abilities to detect the overlaps of real sequencing reads. Mainly, the reads having no true-positive overlap recalled were from highly repetitive regions. In this situation, all of the tools were likely to be affected by the reads from various copies of the repeats and were hard to distinguish between true- and false-positive overlaps effectively.

In addition, we also found another 2 issues that slightly lowered the R% of xRead. One is the crosstalk of the genomic repeats and the sequencing errors in some parts of the reads (see more detailed discussion below). The other is the lack of ground truth. That is, most of the ambiguous reads are potentially from the incomplete regions (i.e., the regions marked as “N” bases) of the reference genome and cannot be mapped. The overlaps with such reads were discarded in the evaluation since their correctness cannot be determined. Thus, some of the reads were recognized as nonoverlapped since the ambiguous reads were the seed reads they overlapped with. We checked a portion of such overlaps and realized that they were also reasonable. That is, most of them were from ambiguous reads to the reads that could be mapped around the incomplete regions with high scores. Supplementary Table S8 gives more detailed information about the proportions of the reads having no correct overlap caused by various issues. Overall, considering that the R% of xRead is still high in absolute terms, it could be not problematic for genome assembly.

The metrics about the coverage and connectivity of the produced graphs were further assessed (C%, Gap Num., and Con. Num. columns of Supplementary Table S7). Similarly, the graphs are highly connected (i.e., relatively low numbers of connected components) and able to cover nearly the whole genomes with few gaps. It is also worth noting that for all of the tools, the number of connected components slightly increased. This is also due to real sequencing reads being more error-prone and likely to cause small components consisting of a few accidentally aligned reads. Partial evidence is that all the tools have obviously lower numbers of connected components on the human PacBio HiFi dataset than that of the ONT fast mode dataset. However, such small and noisy components could also not seriously affect genome assembly since it is not difficult to implement filtration by the size and quality of the overlaps. Furthermore, we also implemented graph expansion for the graphs produced by xRead (Supplementary Table S9). Consistent with that of simulated datasets, the overall sensitivity of the graphs obviously improved and was nearly saturated after 5 iterations.

The read overlaps in local regions were also investigated. The violin plots of FPRs and R% of various genomic blocks are in Fig. 3D, E. xRead had the highest number of blocks with zero FPR, and the distributions of the R% of various tools were quite similar. Moreover, it was observed that most of the reads having false-positive and missing overlaps concentrated in a few blocks, leading to local high FPRs and low R%. We further investigated those blocks and found that the false positives mainly derived from highly repetitive regions. However, by separately investigating low R% blocks, we observed that the overlaps missed by xRead were caused by more complicated interactions of sequencing errors and repeats as follows.

First, most of the missing overlaps were caused by error-prone read parts. In real data, some parts of the long reads have very high errors (especially ONT reads), so there is a lack of matches in those local regions. If the error-prone part is very long and in the inner region of the read, a lowly scored skeleton could be produced and filtered out by xRead. Another case is, as mentioned above, that the low-quality part is near either of the ends of the read where xRead could skip the skeleton due to the placement of the overlap.

Second, a small proportion of the missing overlaps were due to repeats as well as the design of xRead. During the construction of alignment skeletons, xRead initially does not use highly repetitive minimizers, which occur more than a threshold of times in the index since most of them bring false-positive matches, which may affect both precision and performance. In most cases, the remaining minimizers are enough to build correct alignment skeletons. However, some of the reads from repetitive regions could lack matches, and xRead employs an additional approach to solve the problem. That is, for the low-scored skeletons having a high number of repetitive minimizers, xRead uses the initial matches of the less repetitive minimizers as anchors and re-searches the matches between repetitive minimizers around the anchors to refine the skeletons. This helps to resolve most of the repetitive reads. However, in very rare cases, the reads are short and enclosed in long repeats. Their skeletons are of low specificity and could bring many false positives so that xRead discards them.

### The use of xRead for *de novo* assembly

The effect of xRead on genome assembly was further assessed. We tried to integrate xRead into a couple of state-of-the-art assemblers, such as NextDenovo [29], Wtdbg2 [17], Hifiasm [32], Shasta [18], Flye [25], and Canu [15]. This is nontrivial since for most of them, the various modules (such as overlapping, layout, and consensus) are coupled with specific in-memory data structures (also lack of published details), which do not allow flexible plug-ins. Under such circumstances, we made efforts to build an in-house assembly pipeline that uses the xRead graph as input and borrows 2 open-source modules from other assemblers, that is, the layout module of NextDenovo (NextGraph) and the consensus module of Wtdbg2 (wtpoa-cns). Further, an in-house postprocessing script developed in one of our previous telomere-to-telomere (T2T) assembly studies [50] was used to reduce the structural misassemblies caused by the incompatibility of the modules. In the benchmark, we assessed the final assemblies (termed as xRead-pipe) as well as the intermediate outputs through NextGraph and wtpoa-cns modules (termed xRead-nd and xRead-wtpoa, respectively).

Twelve simulated datasets (Table 1) from 4 genomes (*E. coli, A. thaliana, D. melanogaster*, and *H. sapiens*) in 3 error models (low-quality ONT, high-quality ONT, and PacBio HiFi sequencing) were used for benchmarks. Moreover, 3 ONT real datasets (*C. elegans, D. melanogaster*, and *H. sapiens*) and a human HiFi dataset (Table 1) were also employed. We tried to implement 6 pipelines (i.e., xRead-pipe, NextDenovo, Wtdbg2, Flye, Shasta, and Hifiasm) on all 12 (simulated) + 4 (real) datasets for comparison. Since Shasta is not suited to low-quality ONT datasets and Hifiasm is specifically designed for HiFi data, their corresponding results were excluded. QUAST [42] (version 5.2.0) was employed to evaluate the assemblies by the following metrics: assembly length, the number of contigs, N10/N50/N90 statistics, genome fraction, the number of all and structural misassemblies. It is worth noting that we distinguished the assembly errors by local sequence errors (small-scale sequence expansions and collapses) or structural errors (large-scale misjoins) since the latter are usually much more difficult to resolve and should be more focused.

The results are in Table 2 (real) and Supplementary Tables S11–S12 (simulated), respectively. It indicates that the completeness, correctness, and contiguity of xRead-pipe are close to that of state-of-the-art assemblers, while its performance is obviously higher. Some intermediate results also suggested that some features of xRead were also not being fully utilized by this straightforward integration, and it also has the potential to further improve assembly quality by a more tailored layout and consensus modules. Mainly, 3 issues were observed as follows.

**Table 2:** Statistics of assembly results on real datasets

Datasets	Tool^a^	Assembly length (bp)^b^	#Ctgs^c^	N10 (kb)^d^	N50 (kb)^d^	N90 (kb)^d^	Genome fraction (%)^e^	#Misassemblies^f^	#SEs^g^	Real time^h^	Peak memory (GB)^i^
***C. elegans***	xRead-nd	106,383,147	50	6,183.59	3,561.75	970.09	97.989	186	18	360.4 (s)	7.97
	xRead-wtpoa	102,908,753	50	7,218.37	3,645.98	948.68	98.893	45	11	753.8 (s)	18.93
	xRead-pipe	102,454,687	98	4,753.84	2,176.12	637.66	99.155	39	0	—	—
	NextDenovo	103,679,213	32	15,672.21	5,776.25	2136.32	99.547	76	2	7,979.5 (s)	12.94
	Wtdbg2	99,706,610	82	10,659.58	3,841.09	941.37	97.711	62	11	1,591.5 (s)	20.47
	Flye	102,735,830	57	6,598.99	3,310.01	1,190.73	99.745	81	3	9,046.0 (s)	45.57
	Shasta	99,144,317	66	5,428.85	2,831.68	950.70	97.369	38	1	611.5 (s)	40.47
	Hifiasm	—	—	—	—	—	—	—	—	—	—
***D. melanogaster***	xRead-nd	145,917,905	64	21,360.15	14,378.63	1,523.78	89.955	598	82	719.2 (s)	12.56
	xRead-wtpoa	146,420,770	64	21,611.07	14,367.83	1,508.29	90.973	393	77	1,210.0 (s)	20.42
	xRead-pipe	142,950,657	151	15,209.22	10,453.23	532.13	90.979	333	1	—	—
	NextDenovo	136,332,796	31	27,936.35	22,718.49	2,307.26	92.572	205	13	7,444.5 (s)	19.45
	Wtdbg2	155,277,279	933	21,514.16	7,152.20	66.96	91.496	336	31	1,753.0 (s)	27.92
	Flye	139,561,820	165	27,939.08	21,917.50	950.81	93.763	301	8	7,841.6 (s)	50.84
	Shasta	133,568,381	152	27,932.29	21,763.69	944.65	91.181	242	6	457.5 (s)	27.07
	Hifiasm	—	—	—	—	—	—	—	—	—	—
***H. sapiens*** ***(ONT fast mode)***	xRead-nd	2,858,898,337	971	27,779.62	6,055.25	1,386.12	90.401	3,018	358	20.89 (h)	22.76
	xRead-wtpoa	2,856,254,648	971	27,779.99	6,216.56	1,554.92	92.459	686	261	24.18 (h)	37.05
	xRead-pipe	2,850,159,937	1410	21,912.39	4,486.01	1,155.18	92.405	372	29	—	—
	NextDenovo	2,782,862,247	638	57,559.56	25,059.84	3,780.30	91.614	329	108	71.89 (h)	168.75
	Wtdbg2	2,729,955,397	4257	31,781.95	11,412.92	1,354.65	88.623	522	400	20.12 (h)	158.35
	Flye	2,847,205,020	3048	59,028.68	21,863.18	2,830.99	93.101	964	177	32.39 (h)	217.85
	Shasta	2,768,613,021	5627	4,426.75	1,775.63	340.55	90.738	366	34	1.45 (h)	293.24
	Hifiasm	—	—		—	—	—	—	—	—	—
***H. sapiens (PacBioHiFi)***	xRead-nd	2,972,271,147	2,478	31,619.87	12,035.12	662.49	95.209	3,269	253	7.16 (h)	20.61
	xRead-wtpoa	2,957,818,291	2,478	32,009.50	12,031.25	688.13	94.894	2,975	157	9.44 (h)	22.88
	xRead-pipe	2,893,264,635	2,607	26,185.02	8,411.10	509.92	93.775	1,583	12	—	—
	NextDenovo	2,852,886,493	1,689	77,210.10	20,583.16	1,351.32	93.392	769	79	16.42 (h)	86.67
	Wtdbg2	2,769,287,788	2,295	44,134.56	14,168.42	2,005.26	90.804	619	135	10.32 (h)	110.71
	Flye	2,918,011,841	3,121	59,296.15	24,686.38	2,047.19	94.656	2,678	107	25.88 (h)	151.04
	Shasta	3,060,152,361	11,261	90,749.00	31,895.10	878.29	97.218	3,357	53	3.84 (h)	547.01
	Hifiasm	3,089,368,991	664	139,105.11	87,025.29	9,357.14	98.353	3,693	854	6.07 (h)	93.43

a The assemblies were generated using 8 pipelines and benchmarked using QUAST and an in-house assessment script to assess the misassemblies.

b The total number of bases in all contigs.

c The total number of contigs.

d N10/N50/N90: The length of the shortest contig at 10%/50%/90% of the assembly.

e The percentage of aligned bases of the reference genome.

f The number of all misassemblies, including both local sequence errors and structural errors.

g The number of structural errors.

h The overall real time of the tools’ cost on simulated datasets using 30 threads; the results marked by “s” and “h” indicate CPU hours and CPU seconds, respectively.

i The peak memory of the tools (in GB) on simulated datasets.

#### xRead-pipe reduces the overall computational cost of the genome assembly

For nearly all the datasets, both the runtime and memory usage of xRead-pipe are lower than most of the other assemblers (Table 2 and Supplementary Table S11, Real time and Peak Memory columns), suggesting that xRead is beneficial to the overall performance of assembly. Especially, xRead-pipe outperforms the original NextDenovo and Wtdbg2 pipelines by on average 2.5 times speedup and 4.2 times lower memory footprints. It is also worth noting that Shasta is the fastest one on human datasets, while it also has the highest memory footprints, indicating a trade-off between time and memory cost. One example is that Shasta has a 776-GB peak memory on the simulated human HiFi dataset (17 and 37 GB for xRead-nd and xRead-wtpoa, respectively), which is a non-neglectable requirement for computational resource and could affect its scalability due to hardware limitation.

#### xRead-pipe is able to produce correct assemblies

The QUAST evaluation (Table 2, Supplementary Table S12) indicates that the overall xRead-pipe made comparable or a lower numbers of mistakes than that of other pipelines, with quite similar assembly completeness (assembly lengths and genome fractions). This suggests that, using the xRead graph, the assembly quality of the pipeline can reach the same level of state-of-the-art tools. We further investigated the types of misassemblies and found that, for all the assemblers, most of the misassemblies were local sequence errors, and the numbers of structural errors were much lower. Especially, the assemblies of xRead-pipe had the lowest numbers of structural errors among all the assemblers. This should be praised since the small errors are easier to fix by advanced error correction and consensus sequence generation approaches. However, nearly all the large misjoins are related to complex repeats and are nontrivial to deal with.

The results of xRead-nd, xRead-wtpoa, and xRead-pipe showed decreasing numbers of misassemblies, indicating the improvement in correctness by various modules. We further tracked their behaviors and found that a primary cause of the errors was the incompatibility of the layout module. That is, NextDenovo is in a correction-then-assembly design that takes advantage of a tailored correction module to reduce sequencing errors and recluster reads in advance. This is critical to read layout, but it does not allow xRead graphs as input. Under such circumstances, the forced layout module (NextGraph) produced more errors. Further, a large proportion of the errors were amended by wtpoa and the in-house correction script. Wtpoa mainly improved the quality of consensus sequence and reduced the number of sequence errors, especially the ones caused by ONT sequencing noise. It also rescued a proportion of structural error. Further, by contig realignment (refer to the Methods section), the in-house script more effectively detected misjoin regions and largely eliminated the structural errors. With the 2 modules, xRead-pipe is able to catch up or outperform the original NextDenovo pipeline (and other assemblers as well) at assembly correctness.

There are still errors caused by the crosstalk of xRead and NextGraph that cannot be eliminated. One is due to the very long repeats such as segmental duplications that the read length is still not enough to solve them thoroughly. Other assemblers also have such problems as well. On the other hand, it is also non-neglectable that NextGraph may not fully take advantage of xRead. In the xRead graphs, seed reads are critical to connect the various parts of the donor genome. They should be given higher weights and used as the backbone during assembly. However, NextGraph does not have such a weight-tuning function. It implemented graph simplification by iteratively resolving nonlinear structures using some heuristics based on read overlap lengths, identities, and depths. With its own heuristics, some of the overlaps between seed reads were mistakenly discarded and leaving structural errors. A typical case is the removal of the z-clip structure (an example is shown in Supplementary Fig. S2), that is, due to the lack of adaptive weighting, some critical edges in nonlinear structures like z-clip structures were mistakenly removed and resulted in the misjoins between nonsuccessive genomic parts.

#### xRead-pipe has comparable continuity to state-of-the-art approaches

The results (Table 2 and Supplementary Table S12, N10/N50/N90 columns) suggest that the continuity of xRead-pipe is comparable to or slightly lower than that of other assemblers. We investigated the intermediate results of xRead-pipe and found that the decreased continuity is caused by the removal of some critical edges by NextGraph, which made assembly gaps. For most of the assembly gaps in the results of xRead-pipe, the successive genome parts were initially connected in the graphs of xRead. The primary cause is still the heuristics of NextGraph to handle the z-clip structures. As mentioned above, the seed reads in z-clip structures should serve as the backbone of the xRead graph. However, NextGraph selected the branches by its own rules and did not consider the importance of seed reads, and thus mistakes occurred (an example is in Supplementary Fig. S3, and a more complicated one is in Supplementary Fig. S4). Therefore, to further improve assembly continuity, a tailored layout method should be developed for xRead to better use the topology as well as other evidence provided by the graph.

Moreover, it is also non-neglectable that a trade-off can be observed between the continuity and correctness of assembly. That is, the assemblers producing longer contigs also made higher numbers of structural errors. Such errors have no correlation with the lengths of contigs (Supplementary Fig. S5) but are related to genomic contexts such as repeats, indicating that they could be partially caused by some relatively aggressive repeat-handling strategies of the assemblers. We realigned the error-containing contigs and investigated the mapping positions of their various parts. With the annotation of RepeatMasker, we found that the errors mostly happened around those well-known repetitive elements such as short interspersed nuclear elements (SINEs), long interspersed nuclear elements (LINEs), simple tandem repeats, and centromeric repeat patterns.

It is worth noting that the errors may also occur in very long contigs, which are usually produced with high confidence. An example is in Supplementary Fig. S6. This is a contig of Flye whose length is 50.0 Mb, close to its N10 statistics. A clipping was detected around contig_2932: 42,085,000, which indicates a translocation. The contig was then split into 2 segments (42.1 Mb and 6.9 Mb, respectively), which can be aligned to reference at the positions Chr3: 88,844,116 and Chr6: 19,746,930, respectively. Another example is in Supplementary Fig. S7. This is a contig of NextDenovo whose length is 43.5 Mb, also close to its N10 statistics. A clipping event was observed at ctg003710: 12,544,314, indicating a translocation error in the contig. The contig then can be split aligned to reference at 2 positions: Chr3: 91,045,032 and Chr6: 57,667,858.

## Discussion

Long-read sequencing technologies are promising for the high-quality genome assembly of various species while they also make new requests to efficiently process thousands of genomes, tens of gigabase-level assembly sizes, and terabase-level datasets. Read-overlapping graph construction is the most computationally intensive step in this task and especially gets challenged. Herein, we propose xRead, a highly scalable graph construction approach. With its novel coverage-guided model and lightweight alignment skeletons, xRead implicitly explores the reads from various parts of the donor genome and converts the construction task to a fast read-mapping, which substantially improves the overall performance. Moreover, the produced graph can be also well connected (i.e., the reads are comprehensively connected with few false-positive overlaps).

xRead is to some extent designed in a minimalist style that constructs a simplified, but not oversimplified, overlapping graph. The graph can support both of the strategies commonly used by the state-of-the-art assemblers: correction-then-assembly [14, 15, 32] and assembly-then-correction [17, 18, 51]. For correction-then-assembly, the approach can implicitly cluster the reads of the same genomic regions since all of them can be aligned to the seed read(s) from there. Therefore, the graph becomes a suitable input to read correction. For assembly-then-correction, xRead also paves the way to successful layout since the graph is highly connected with few false-positive edges. Further, the alignments between nonseed and seed reads can also be directly used to infer local consensus in the correction phase. With these features, xRead lays a foundation for downstream assembly steps. It is also worth noting that the seed reads are natively the representatives of various genome parts. Thus, they should be seen as the backbone of assembly and well handled by approaches specifically designed for either of the 2 strategies.

xRead has the potential to convert its produced graph to other styles, such as restricted to the best or containing all the significant overlaps, which are popularly used strategies by other overlapping tools. This is done by using transitive relationships. It is not difficult for a seed read to collect all the reads overlapping with it since such reads are either directly aligned to it or aligned to another seed read in the same connected component. For the latter case, the assumption also stands due to both the high connectivity (i.e., seed reads are well connected as mentioned) and precision (i.e., in most cases, all the transitive edges/overlaps are true positive) of the graph. Thus, the overlaps to the seed read can be thoroughly evaluated to find out the best or all the significant ones. It is similar to a nonseed read (i.e., the read is initially aligned to a seed read and then involved in a connected component). Further, the overlaps to the nonseed read can be inferred and evaluated through all the overlaps implied by the component. This feature is partially demonstrated by simulation and real benchmarks (Supplementary Tables S5 and S9; also refer to Supplementary Fig. S8A for schematic illustrations).

xRead still has some limitations to handle the reads from ultra-long repeats, which is also a common problem with all read overlapping tools. As the lengths are not enough to span such repeats, the reads from various copies are not distinguishable with the crosstalk of their intrinsic similarity and sequencing noise. Under such circumstances, xRead could align the seed reads from various copies together and map nonseed reads to some of them randomly. Then all the reads are connected as 1 component and the graph collapses, which is also a common case during read layout (refer to Supplementary Fig. S8B for schematic illustrations). A feasible solution is to use tailored approaches to precisely correct sequencing errors and recluster the reads. With the improved base quality, the reads would have a good chance to be distinguished and the graph can be corrected. xRead also makes its own efforts to reduce this effect by 2 means. One is the CRO-based alignment skeletons, which restrict highly confident overlaps and reduce the chance of mapping a read to a wrong copy. Another is that xRead outputs the inferred coverage for each of the reads (in its PAF format output, CV FLAG). This is a good repeat-indicating marker since the repetitive reads have obviously higher coverage; furthermore, they can be conveniently focused in later steps.

The use of xRead for whole-genome assembly was also evaluated, although it is hard to integrate a standalone overlapping tool into the state-of-the-art assemblers since most of their modules are coupled by various heuristics and do not allow third-party plug-ins. We tried our best to build a pipeline by borrowing the layout and consensus modules from NextDenovo and Wtdbg2, respectively. Considering both the time cost and RAM usage, the pipeline showed higher performance and scalability; meanwhile, it also achieved the same order of completeness, correctness, and contiguity as that of those state-of-the-art assemblers. The results suggest that xRead is promising to scalable assembly. Further, we also would like to claim that the ability of xRead is not fully exerted with the straightforward (to some extent rough) integration. For example, due to the lack of a readily made module, this pipeline can still not fully take advantage of precise read correction (which is critical to repeat-handling). Moreover, existing layout methods also could not fully consider the characteristics of the graphs produced by xRead. It is an important future work (has been ongoing) to develop novel tools based on xRead graph to achieve a high-quality, efficient, and scalable genome assembly. We realize that there could be 3 key points to the development as follows.

First, it is critical to develop a tailored read correction method for xRead. The seed reads can be directly used as anchors to cluster the reads from the same genomic regions. Thus, read correction can be straightforwardly implemented by similar approaches to that of state-of-the-art assemblers, such as multiple sequence alignment [52], pseudo-variant calling [53], and sequence graph analysis [14]. Moreover, it also needs to develop novel methods to use the correction information to distinguish and recluster the reads from various repeat copies to refine the graph.

Second, it also needs to design a specific read layout method with xRead graph. That is, various weights should be added to the edges and carefully considered since the connectivity of the CRO graph depends on the edges and short paths between seed reads, and they should be well handled. It is feasible to build an essential backbone by seed reads as they are well connected and implicitly distributed along the whole genome. Moreover, nonseed reads can be used as extra evidence to rescue unconnected contigs, prevent misassemblies, and support the consensus phase.

Third, it is still an open problem to implement an efficient haplotype assembly. For xRead, one feasible way is to expand the graph dynamically with the transitive relationships during layout and precisely analyze the alignments among reads to adaptively reconstruct haplotype-specific paths. Another one is to use the initially assembled genome as reference to implement realignment, variant calling, and phasing (i.e., achieve haplotype assembly in a “*de novo* and resequencing” way. This approach could be more suited to tasks having various types of reads (such as many T2T-assembly tasks) since the resequencing style postprocessing is more convenient to integrate the data and use various kinds of tools to correct misassemblies and construct haplotypes. Moreover, it is also feasible to keep low cost all the way and, even more, simultaneously handle many genomes in a step-parallel approach to achieve very high overall performance in large-scale genomics studies.

## Methods

### The selection and index of seed reads

xRead initially selects ${{\mathrm{P}}}_0{\mathrm{\% }}$ of longest reads (default value: 3%) as seed reads at first and assigns zero coverage to all the input reads. Other than the first iteration, xRead selects seed reads with an updated profile of read coverage—that is, ${{\mathrm{P}}}_{\mathrm{s}}{\mathrm{\% }}$ of the low-covered reads (default value: 10%) are randomly selected as seed reads, where the low-covered reads are defined by a threshold ${{\mathrm{T}}}_{{\mathrm{RC}}}$ derived from the average coverage of their read parts.

A minimizer-based index is then built for the seed reads. Given a seed read, a set of windows of size ${{\mathrm{W}}}_{{\mathrm{SR}}}$ (default value: 5 bp) starting at every single base is defined, and all the *k*-mers (default value: 15 bp) within the windows (for both of the strands) are input into a hash function. The *k*-mer with minimum hash value is chosen to define a quadruple minimizer $( {{V}_{SR},{R}_{SR},{P}_{SR},{S}_{SR}} )$, where ${V}_{SR}$, ${R}_{SR}$, ${P}_{SR}$, and ${S}_{SR}$ indicate the hash value, the read, the position, and the strand of the minimizer, respectively. All the minimizers of the seed reads are recorded and sorted by their hash values for indexing; moreover, a hash table of *l*-mers (default value: 11 bp) is also built as an auxiliary index data structure to accelerate the retrieval and matching of minimizers in the following step.

### Alignment skeleton-based read overlapping

xRead defines all the reads (all the low covered reads) as query reads in the first iteration (other iterations) and aligns them to the selected seed reads to discover new overlaps to construct (refine) the overlapping graph. The alignment is inspired by deSALT [45] and implemented in a modified minimizer-based approach, which is suited to the detection of read overlaps. Given a query read, xRead collects all its minimizers using the same hash function at first. The minimizers are matched to seed reads through the read index, and xRead separately merges colinear matches within the same seed reads to build a set of match blocks (MBs).

xRead uses the MBs as vertices to construct a direct acyclic graph (DAG). Two MBs from the same seed read define an edge if they meet the following conditions:


(1) \begin{eqnarray*}
{D}_q > - k,\,\,{D}_s > - k,\,\,\left| {{D}_q - {D}_s} \right| < \delta \times \min \left( {{D}_q,{D}_s} \right)
\end{eqnarray*}

where ${D}_q$ and ${D}_s$ are the distances between 2 MBs on the query and seed reads, respectively; *k* is the maximum allowed overlap length between MBs; and $\delta $ is a parameter to limit the length difference between ${D}_q$ and ${D}_s$. The weight and penalty are also assigned to each edge based on the number of covered bases and the distance between 2 nodes. The path with the highest score is then inferred in a sparse dynamic programming (SDP) approach by the following recursive equation and is considered the alignment skeleton.


(2) \begin{eqnarray*}
S\left( {M{B}_j} \right) &=& \max \left\{ {S\left( {M{B}_i} \right) + w\left( {M{B}_i \to M{B}_j} \right) - p\left( {M{B}_i \to M{B}_j} \right)} \right\},\\
&&\quad M{B}_i \in \textit{Precusor}\left\{ {M{B}_j} \right\}
\end{eqnarray*}

where $S( {M{B}_j} )$ is the score of the vertex $M{B}_j$, $w( {M{B}_i \to M{B}_j} )$ is the weight of the edge $M{B}_i \to M{B}_j$, and $p( {M{B}_i \to M{B}_j} )$ is the penalty of the edge $M{B}_i \to M{B}_j$.

It is also worth noting that multiple alignment skeletons could be built in practice since a query read usually has true positive overlaps to multiple seed reads. More precisely, xRead removes all the MBs along the path after an alignment skeleton is built. Another skeleton is then built with the updated DAG. The iterative process goes on until no alignment skeleton with high scores can be built.

### The construction and refinement of overlapping graph

xRead keeps a global graph data structure to record read overlaps during the iterative process. The produced alignment skeletons are converted to read overlapping information and supplied to the data structure incrementally. For a given query read, the produced alignment skeletons that meet 1 of the following 3 conditions are filtered out at first since they could be false positives caused by sequencing errors or repeats in local genomic regions: (i) the overlap length is shorter than ${{\mathrm{T}}}_{{\mathrm{OM}}}$ (default value: 500 bp), (ii) the total number of nonredundant bases of all the MBs is lower than ${{\mathrm{T}}}_{{\mathrm{NB}}}$ (default value: 100 bp), and (iii) the overhang length of either read is longer than ${{\mathrm{T}}}_{{\mathrm{OH}}}$ (default value: 2,000 bp). Further, xRead selects confident read overlaps (CROs), that is, the ${{\mathrm{N}}}_{{\mathrm{AS}}}$ (default value: 2) highest scored ones of the remaining alignment skeletons, and records their positions in the corresponding 2 reads.

xRead (re)estimates read coverage with the updated overlapping information. For a given read, its coverage is estimated by the numbers of the seed reads directly connected to it by the CROs and the reads having CROs to the same seed reads, which can be regarded as indirectly aligned to it. It is also worth noting that there could be a proportion of reads being partially overlapped (i.e., some of their read parts have a high number of CROs while other parts have few). Under this circumstance, xRead implements a more precise local estimation—that is, it splits the given read by ${{\mathrm{W}}}_{{\mathrm{RC}}}$ size nonoverlapping windows (default value: 1,000 bp) and separately estimates the coverage of various windows by the reads directly and indirectly connected to them. Further, xRead computes the average of the window coverage as the estimated coverage for a read.

The distribution of the coverage of various reads is then estimated, and the medium of read coverage ${{\mathrm{M}}}_{{\mathrm{RC}}}$ is computed. A threshold ${{\mathrm{T}}}_{{\mathrm{RC}}}$ is set as ${{\mathrm{M}}}_{{\mathrm{RC}}} \times {P}_{RC}$ for the selection of seed reads in the next iteration where ${P}_{RC}$ is a user-defined parameter (default value: 0.5). With given ${{\mathrm{T}}}_{{\mathrm{RC}}}$, xRead monitors the number of newly selected seed reads. If it is too low, xRead considers that there are few reads being lowly covered and outputs the resulting graph in PAF format.

### The inference of comprehensive overlaps

The graph produced by xRead can be regarded as a core graph consisting of the overlaps between the seed and query reads. As some of the *de novo* assembly approaches require comprehensive read overlapping information, xRead provides an additional function to infer the overlaps between nonseed reads and produce a more comprehensive graph. Mainly, it implements an iterative width-first searching approach based on the transitive relationships among the overlaps. That is, for each of the nonseed reads, xRead initially retrieves all the other reads connected to the same seed read(s) it attached and infers the overlaps via the transitive relationships. The length and placement of the inferred overlaps are investigated, and the ones meeting the conditions similar to that of CROs remain. Further, the remaining overlaps are added to the graph as virtual edges, and xRead further expands the graph through them in the following iterations. The transitive overlap-based inference continues until no new legal overlap is found or it reaches a predefined number of iterations.

### Assessment of the sensitivity and precision in produced graph

We use both simulated and real datasets in various read lengths and quality to evaluate the ability of xRead. The precision and sensitivity of the produced graph were assessed with the ground-truth edge set of the overlapping graph (short as ground-truth overlap set). The ground-truth overlap set was generated based on the genomic positions of the reads. For simulated datasets, the read positions are directly given by the output files of the simulator (PBSIM). For real datasets, due to the absence of ground truth, we take advantage of the high mappability of long reads to produce pseudo-ground truth. That is, the reads were aligned to the corresponding reference genome using Minimap2 with default settings. The reads being unaligned or in low mapping quality were marked as ambiguous reads and filtered out (i.e., unused in the benchmark). Further, the remaining reads as well as their mapping positions were used to compose the pseudo-ground-truth set. The overlaps between reads were then collected based on (pseudo-)-ground-truth read positions and used to produce the (pseudo-)-ground-truth overlap set. Since too short overlaps could be caused by coincidence and most of them could be directly removed in downstream assembly steps, herein, only the read overlaps longer than 500 bp were considered in the evaluation. This criterion also refers to previous studies [13].

The generated (pseudo-)-ground-truth overlap set was then used to evaluate the precision and sensitivity. Any reported overlap was considered a true positive only if it matched an overlap in the ground-truth overlap set. It is worth noting that a read from a real dataset may have multiple positions due to the ambiguity of alignment. In such cases, we consider an overlap to be true positive if it matches any of the overlaps derived from corresponding reads in the ground-truth set. Any non-true-positive overlap is seen as a false-positive overlap. The precision and sensitivity were then calculated as ${\mathrm{N}}_{\mathrm{O}}^{TP}/N_O^R$ and ${\mathrm{N}}_{\mathrm{O}}^{TP}/N_O^G$, where ${\mathrm{N}}_{\mathrm{O}}^{TP}$, $N_O^R$, and $N_O^G$ are the number of true-positive overlaps, reported overlaps, and overlaps in the ground-truth set, respectively.

### The implementation of benchmarks

We implemented benchmarks of read overlapping on simulated and real long-read datasets from 9 genomes: *E. coli* (ASM584v2), *S. cerevisiae* (R64), *C. elegans* (WBcel235), *A. thaliana* (TAIR10.1), *D. melanogaster* (Release 6 plus ISO1 MT), *Z. mays* (subsp. *mays* SK) [54], *M. musculus* (GRCm39), *H. sapiens* (T2T-CHM13v2.0), and *A. mexicanum* (AmbMex60DD) [7]. Most of the datasets are ONT or PacBio CLR reads, and xRead was compared with 5 state-of-the-art tools: MHAP (version 2.1.3), MECAT2 (v20190314), Minimap2 (version 2.24), wtdbg2 (version 2.5), and BLEND (version 1.0.0). Moreover, a couple of in-house Python scripts were used to interpret and evaluate the outputs of the tools in various formats. Other state-of-the-art assemblers like Canu, Shasta, NECAT, Flye, and NextDenovo were not included in the read overlapping benchmarks since they do not provide stand-alone modules to output interpretable results or employ generic alignment tools such as Minimap2 for read overlapping.

In addition, we implemented benchmarks of *de novo* assembly on 12 simulated and 4 real datasets. The 12 simulated datasets were from 4 genomes (*E. coli, A. thaliana, D. melanogaster*, and *H. sapiens*) in 3 error models (low-quality ONT, high-quality ONT, and PacBio HiFi sequencing). The 4 real datasets include 3 ONT datasets (*C. elegans, D. melanogaster*, and *H. sapiens*) and a human HiFi dataset. We built an in-house assembly pipeline that uses an overlapping graph of xRead, the layout module of NextDenovo (NextGraph), the consensus module of Wtdbg2 (wtpoa-cns), and an in-house correction script. The 3 pipelines (i.e., xRead-pipe, xRead-nd, and xRead-wtpoa) were compared with 5 state-of-the-art assemblers (i.e., NextDenovo, Wtdbg2, Flye version 2.9.4-b1799, Shasta version 0.12.0, and Hifiasm version 0.19.9) by the evaluation of QUAST.

All the benchmarks were implemented on a server with 4 Intel Xeon 5220R CPUs (96 CPU cores in total) and 1 Terabyte RAM running Linux Ubuntu 16.04. Refer to Supplementary Tables S3, S4, S6, and S7 (Parameter columns) and Supplementary Note 1 for the detailed settings of the tools used in the benchmark.

### Postprocessing of xRead-pipe to misassembly correction

To correct structural errors in assembly, we used an in-house postprocessing script developed in one of our T2T plant genome assembly studies [50]. The method is based on read-to-contig realignments, which work in 2 simple steps: (i) it calculates the per-base depth of contigs to detect candidate misjoin regions, and (ii) it detects highly plausible structural errors and directly eliminates them. A brief description of its implementation is as follows.

In the first step, the method initially realigns the reads to the contigs and calculates the per-base depth of the contigs. A 10-kb sliding window is used to scan the contigs to collect abnormally high and low coverages as well as read clipping signatures. It defines a high coverage region (HCR) as a set of consecutive windows whose average coverage is higher than 3 times the sequencing depth of the whole dataset, while a low coverage region (LCR) is defined as having average coverage lower than 15% of the sequencing depth. Clipping events (CLIPs) are also identified based on the large clippings implied by the realignments within windows. To avoid detecting heterozygous structural variants as false-positive misassemblies, a window is considered a CLIP only if over 60% of the reads in the window have large clippings. The HCR, LCR, and CLIP regions are then combined to form a total set of candidate misjoin regions.

In the second step, the method further verifies misassemblies by several heuristics. The candidate regions are sorted by their genomic positions, and adjacent regions within 20 kb are merged and treated as a single region. HCRs and LCRs shorter than 20 kb are filtered out to avoid local false-positive regions, such as HiFi coverage gaps. Meanwhile, CLIPs within 10 kb of contig ends are also filtered out to avoid the artifacts from contig breakpoints. After filtration, the method simply discards the sequences around the marked structural error regions and splits the contigs.

It is worth noting that the updated contigs could be shortened with the straightforward split operation. However, this restrict method is effective in preventing large misjoins, especially in highly repetitive regions. Moreover, a scaffolding method using long-range sequencing information (e.g., ultra-long reads or Hi-C data) could be a good supplement to rejoin the contigs to achieve highly continuous assembly without loss of correctness.

## Availability of Supporting Source Code and Requirements

Project name: xRead

Project homepage: https://github.com/tcKong47/xRead

Operating system(s): Linux

Programming language: C

Other requirements: None

License: MIT license

RRID:SCR_025372

## Supplementary Material

giaf007_xRead_Supplementary_Material

giaf007_GIGA-D-24-00195_Original_Submission

giaf007_GIGA-D-24-00195_Revision_1

giaf007_GIGA-D-24-00195_Revision_2

giaf007_Response_to_Reviewer_Comments_Original_Submission

giaf007_Response_to_Reviewer_Comments_Revision_1

giaf007_Reviewer_1_Report_Original_Submission Antoine Limasset, Ph.D -- 7/2/2024

giaf007_Reviewer_1_Report_Revision_1 Antoine Limasset, Ph.D -- 12/13/2024

giaf007_Reviewer_2_Report_Original_Submission Anuradha Wickramarachchi -- 8/13/2024

## Data Availability

The following reference genomes were used in this study: *Escherichia coli* (GCF_000005845.2), *Saccharomyces cerevisiae* (GCF_000146045.2), *Caenorhabditis elegans* (GCF_000002985.6), *Arabidopsis thaliana* (GCF_000001735.4), *Drosophila melanogaster* (GCF_000001215.4), *Zea mays* [55], *Mus musculus* (GCF_000001635.27), *Homo sapiens* CHM13 (GCF_009914755.1), *Homo sapiens* HG002 [56], and *Ambystoma mexicanum* (GCA_002915635.3). Real datasets used in this study can be accessed through the following accessions: *Escherichia coli* (SRR19746198), *Caenorhabditis elegans* (SRR19746198), *Drosophila melanogaster* (SRR13070625), *Homo sapiens* HG002 ONT datasets in fast base-calling mode [57], *Homo sapiens* HG002 ONT dataset in super-high-accuracy base-calling mode [58], *Homo sapiens* HG002 PacBio HiFi dataset [59], and *Ambystoma mexicanum* (SRR5349126-SRR5349175). Please refer to Supplementary Tables S1 and S2 for additional information. Additional codes underlying this study can be found in the GitHub repository [60]. An archival copy of the code is available via Software Heritage [61].

## Additional Files

**Supplementary Table S1**. Detailed information of reference genomes.

**Supplementary Table S2**. Availability information of real datasets.

**Supplementary Table S3**. The performance of various tools on simulated datasets.

**Supplementary Table S4**. The yields of various tools on simulated datasets.

**Supplementary Table S5**. The sensitivity of the expanded graphs of xRead on simulated datasets.

**Supplementary Table S6**. The performance of various tools on real sequencing datasets.

**Supplementary Table S7**. The yields of various tools on real sequencing datasets.

**Supplementary Table S8**. Percentages of the reads not correctly overlapped by xRead with various causes.

**Supplementary Table S9**. The sensitivity of the expanded graphs of xRead on real sequencing datasets.

**Supplementary Table S10**. The external memory of various tools on both simulated and real datasets.

**Supplementary Table S11**. The performance of *de novo* assembly on simulated datasets.

**Supplementary Table S12**. Statistics of assembly results on simulated datasets.

**Supplementary Fig. S1**. The performance and external memory usage on simulated and real datasets.

**Supplementary Fig. S2**. An example of the mistaken selection of edges at branching paths by NextGraph.

**Supplementary Fig. S3**. An example of the removal of critical edges of z-clip structure by NextGraph.

**Supplementary Fig. S4**. An example of the removal of critical edges of z-clip structure by NextGraph.

**Supplementary Fig. S5**. The number of misassemblies in contigs longer than N10/N50/N90 values of various assemblers.

**Supplementary Fig. S6**. An example of the structural error in a contig of Flye on the real human PacBio HiFi dataset.

**Supplementary Fig. S7**. An example of the structural error in a contig of NextDenovo on the real human ONT dataset.

**Supplementary Fig. S8**. The schematic illustrations for the features of xRead approach.

**Supplementary Note 1**. The command and parameters used for assembly benchmarks.

## Abbreviations

CLIP: clipping event; CPU: central processing unit; CRO: confident read overlap; CUDA: compute unified device architecture; DAG: direct acyclic graph; DDF: distance difference factor; FPR: false-positive overlap rate; GB: gigabyte; GIAB: Genome in a Bottle; HCR: high coverage region; LCR: low coverage region; LINE: long interspersed nuclear elements; MB: match block; OLC: overlap-layout-consensus; ONT: Oxford Nanopore Technologies; PacBio: Pacific Biosciences; PAF: pairwise alignment format; RAM: random access memory; SDP: sparse dynamic programming; SIMD: single instruction multiple data; SINE: short interspersed nuclear elements; SMRT: single molecule real time; T2T: telomere-to-telomere.
