# Supplementary material for: xRead: a coverage-guided approach for scalable construction of read overlapping graph
Source: Gigascience. 2025 Feb 17;14:giaf007. doi: 10.1093/gigascience/giaf007 (PMC11831799; doi:10.1093/gigascience/giaf007)
Supplement: giaf007_xRead_Supplementary_Material [file giaf007_xread_supplementary_material.docx]

**xRead: a coverage-guided approach for scalable construction of read overlapping graph**

**Supplementary Notes**

Tangchao Kong^1, 2^, Yadong Wang^1, 2, *^, Bo Liu^1, 2, *^

^1^Center for Bioinformatics, faculty of computing, Harbin Institute of Technology, Harbin, Heilongjiang 150001, China

^2^ Key Laboratory of Biological Bigdata, Ministry of Education, Harbin Institute of Technology, Harbin, Heilongjiang 150001, China

* Corresponding author

Contact: 21B903020@stu.hit.edu.cn, ydwang@hit.edu.cn, bo.liu@hit.edu.cn

**Contents**

[Supplementary Table 1. Detailed information of reference genomes 3](#_Toc183535009)

[Supplementary Table 2. Availability information of real datasets 4](#_Toc183535010)

[Supplementary Table 3. The performance of various tools on simulated datasets 5](#_Toc183535011)

[Supplementary Table 4. The yields of various tools on simulated datasets 8](#_Toc183535012)

[Supplementary Table 5. The sensitivity of the expanded graphs of xRead on simulated datasets 11](#_Toc183535013)

[Supplementary Table 6. The performance of various tools on real sequencing datasets 12](#_Toc183535014)

[Supplementary Table 7. The yields of various tools on real sequencing datasets 14](#_Toc183535015)

[Supplementary Table 8. Percentages of the reads not correctly overlapped by xRead with various causes 16](#_Toc183535016)

[Supplementary Table 9. The sensitivity of the expanded graphs of xRead on real sequencing datasets 17](#_Toc183535017)

[Supplementary Table 10. The external memory of various tools on both simulated and real datasets. 18](#_Toc183535018)

[Supplementary Table 11. The performance of de novo assembly on simulated datasets 19](#_Toc183535019)

[Supplementary Table 12. Statistics of assembly results on simulated datasets 21](#_Toc183535020)

[Supplementary Figure 1. The performance and external memory usage on simulated and real datasets. 24](#_Toc183535021)

[Supplementary Figure 2. An example of the mistaken selection of edges at branching paths by NextGraph. 25](#_Toc183535022)

[Supplementary Figure 3. An example of the removal of critical edges of z-clip structure by NextGraph. 26](#_Toc183535023)

[Supplementary Figure 4. An example of the removal of critical edges of z-clip structure by NextGraph. 27](#_Toc183535024)

[Supplementary Figure 5. The number of mis-assemblies in contigs longer than N10/N50/N90 values of various assemblers. 28](#_Toc183535025)

[Supplementary Figure 6. An example of the structural error in a contig of Flye on the real human PacBio HiFi dataset. 29](#_Toc183535026)

[Supplementary Figure 7. An example of the structural error in a contig of NextDenovo on the real human ONT dataset. 30](#_Toc183535027)

[Supplementary Figure 8. The schematic illustrations for the features of xRead approach. 31](#_Toc183535028)

[Supplementary Note 1. The command and parameters used for assembly benchmarks 32](#_Toc183535029)

# Supplementary Table 1. Detailed information of reference genomes

| **No.** | **Reference genome** | **Version** | **Genome size** | **Availability** |
| --- | --- | --- | --- | --- |
| 1 | *Escherichia coli* | ASM584v2 | 4.6 Mb | https://www.ncbi.nlm.nih.gov/datahub/genome/GCF_000005845.2/ |
| 2 | *Saccharomyces cerevisiae* | R64 | 12.1 Mb | https://www.ncbi.nlm.nih.gov/datahub/genome/GCF_000146045.2/ |
| 3 | *Caenorhabditis elegans* | WBcel235 | 100.3 Mb | https://www.ncbi.nlm.nih.gov/datahub/genome/GCF_000002985.6/ |
| 4 | *Arabidopsis thaliana* | TAIR10.1 | 119.1 Mb | https://www.ncbi.nlm.nih.gov/datahub/genome/GCF_000001735.4/ |
| 5 | *Drosophila melanogaster* | Release 6 plus ISO1 MT | 143.7 Mb | https://www.ncbi.nlm.nih.gov/datahub/genome/GCF_000001215.4/ |
| 6 | *Zea mays (SK)* | GWHAACS00000000 | 2.2 Gb | https://ngdc.cncb.ac.cn/gwh/Assembly/123/show |
| 7 | *Mus musculus* | GRCm39 | 2.7 Gb | https://www.ncbi.nlm.nih.gov/datahub/genome/GCF_000001635.27/ |
| 8 | *Homo sapiens* ^a^ | T2T-CHM13v2.0 | 3.1 Gb | https://www.ncbi.nlm.nih.gov/datasets/genome/GCF_009914755.1/ |
| 9 | *Homo sapiens* ^b^ | T2T-HG002v1.1-maternal | 3.1 Gb | https://s3-us-west-2.amazonaws.com/human-pangenomics/T2T/HG002/assemblies/hg002v1.1.mat.fasta.gz |
| 10 | *Ambystoma mexicanum* | AmbMex60DD | 28.2 Gb | https://www.ncbi.nlm.nih.gov/datahub/genome/GCA_002915635.3/ |

(a) The T2T-CHM13v2.0 genome was utilized as the reference for simulating data, as well as serving as the reference genome in all overlap and assembly benchmarks based on simulated datasets.

(b) For real datasets, the maternal haplotype of the T2T-HG002v1.1 genome was employed as the reference in all overlap and assembly benchmarks, as the real data originated from the HG002 sample.

# Supplementary Table 2. Availability information of real datasets

| **No.** | **Genome** | **Sample** | **Accession** |
| --- | --- | --- | --- |
| 1 | *Escherichia coli* | Isolate: EM130d | SRR19746198 |
| 2 | *Caenorhabditis elegans* | strain N2 | SRR10028111 |
| 3 | *Drosophila melanogaster* | BDGP genome strain | SRR13070625 |
| 4 | *Homo sapiens* | HG002 | https://s3-us-west-2.amazonaws.com/human-pangenomics/NHGRI_UCSC_panel/HG002/hpp_HG002_NA24385_son_v1/nanopore/HG002_ucsc_Oct_2018_Guppy_3.0.fastq.gz |
| 5 | *Homo sapiens* | HG002 | https://labs.epi2me.io/gm24385_q20_2021.10/ |
| 6 | *Homo sapiens* | HG002 | https://ftp-trace.ncbi.nlm.nih.gov/giab/ftp/data/AshkenazimTrio/HG002_NA24385_son/PacBio_CCS_15kb/alignment/HG002.Sequel.15kb.pbmm2.hs37d5.whatshap.haplotag.RTG.10x.trio.bam |
| 7 | *Ambystoma mexicanum* | PRJNA378970 | [SRR5349126](https://trace.ncbi.nlm.nih.gov/Traces/sra?run=SRR5349126)-[SRR5349175](https://trace.ncbi.nlm.nih.gov/Traces/sra?run=SRR5349175) |

# Supplementary Table 3. The performance of various tools on simulated datasets

| **Tool** | **Parameter ^a^** | **Thread ^b^** | **Real time ^c^** | **CPU time ^c^** | **Memory (GB) ^d^** |
| --- | --- | --- | --- | --- | --- |
| **Simulated ONT datasets with an average accuracy of 87%** | | | | | |
| ***Escherichia coli*** | | | | | |
| xRead | -k 15 -l 11 -w 5 -x 3 -X 10 -t 8 -M 16 | 8 | 7.96 | 15.27 | 0.35 |
| MHAP | --settings 2 --num-threads 8 | 8 | 51.18 | 440.98 | 16.39 |
| MECAT2 | -outfmt paf -num_threads 8 | 8 | 13.590 | 80.46 | 2.11 |
| minimap2 | -x ava-ont -t 8 | 8 | 18.84 | 112.22 | 3.31 |
| wtdbg2 | -p 0 -k 15 -AS 2 -s 0.05 -L 5000 -t 8 | 8 | 18.24 | 51.80 | 1.42 |
| BLEND | -x ava-ont -t 8 | 8 | 20.85 | 52.39 | 2.28 |
| ***Saccharomyces cerevisiae*** | | | | | |
| xRead | -k 15 -l 11 -w 5 -x 3 -X 10 -t 8 -M 16 | 8 | 19.11 | 47.72 | 0.88 |
| MHAP | --settings 2 --num-threads 8 | 8 | 137.37 | 1213.45 | 20.25 |
| MECAT2 | -outfmt paf -num_threads 8 | 8 | 55.47 | 325.66 | 5.11 |
| minimap2 | -x ava-ont -t 8 | 8 | 53.26 | 342.10 | 5.69 |
| wtdbg2 | -p 0 -k 15 -AS 2 -s 0.05 -L 5000 -t 8 | 8 | 71.10 | 518.35 | 2.71 |
| BLEND | -x ava-ont -t 8 | 8 | 50.77 | 152.16 | 5.06 |
| ***Caenorhabditis elegans*** | | | | | |
| xRead | -k 15 -l 11 -w 5 -x 3 -X 10 -t 8 -M 16 | 8 | 321.74 | 1906.02 | 4.83 |
| MHAP | --settings 2 --num-threads 8 | 8 | 1263.42 | 12322.48 | 77.56 |
| MECAT2 | -outfmt paf -num_threads 8 | 8 | 689.07 | 4681.95 | 34.64 |
| minimap2 | -x ava-ont -t 8 | 8 | 1650.88 | 12039.55 | 21.91 |
| wtdbg2 | -p 0 -k 15 -AS 2 -s 0.05 -L 5000 -t 8 | 8 | 1929.32 | 15271.74 | 18.03 |
| BLEND | -x ava-ont -t 8 | 8 | 498.63 | 2260.97 | 17.43 |
| ***Arabidopsis thaliana*** | | | | | |
| xRead | -k 15 -l 11 -w 5 -x 3 -X 10 -t 8 -M 16 | 8 | 329.20 | 1744.48 | 5.59 |
| MHAP | --settings 2 --num-threads 8 | 8 | 3449.58 | 21751.85 | 81.20 |
| MECAT2 | -outfmt paf -num_threads 8 | 8 | 739.72 | 4912.01 | 34.68 |
| minimap2 | -x ava-ont -t 8 | 8 | 1533.62 | 11737.85 | 27.46 |
| wtdbg2 | -p 0 -k 15 -AS 2 -s 0.05 -L 5000 -t 8 | 8 | 2019.11 | 16175.72 | 22.06 |
| BLEND | -x ava-ont -t 8 | 8 | 592.72 | 2637.43 | 23.55 |
| ***Drosophila melanogaster*** | | | | | |
| xRead | -k 15 -l 11 -w 5 -x 3 -X 10 -t 8 -M 16 | 8 | 402.30 | 2220.21 | 6.00 |
| MHAP | --settings 2 --num-threads 8 | 8 | 7325.11 | 50070.11 | 98.50 |
| MECAT2 | -outfmt paf -num_threads 8 | 8 | 909.53 | 6077.60 | 34.65 |
| minimap2 | -x ava-ont -t 8 | 8 | 1766.14 | 13441.95 | 37.48 |
| wtdbg2 | -p 0 -k 15 -AS 2 -s 0.05 -L 5000 -t 8 | 8 | 2218.79 | 17815.28 | 26.38 |
| BLEND | -x ava-ont -t 8 | 8 | 714.18 | 3446.04 | 30.65 |
| ***Zea mays (SK)*** | | | | | |
| xRead | -k 15 -l 11 -w 5 -x 3 -X 10 -t 24 -M 24 | 24 | 21.93 (h) | 500.24 (h) | 14.02 |
| MHAP | --settings 2 --num-threads 24 | 24 | - | - | - |
| MECAT2 | -outfmt paf -num_threads 24 | 24 | 35.30 (h) | 819.45 (h) | 73.00 |
| minimap2 | -x ava-ont -t 24 | 24 | 277.69 (h) | 1513.49 (h) | 64.34 |
| wtdbg2 | -p 19 -AS 2 -s 0.05 -L 5000 -t 24 | 24 | 26.38 (h) | 592.78 (h) | 336.19 |
| BLEND | -x ava-ont -t 24 | 24 | 30.81 (h) | 400.97 (h) | 40.35 |
| ***Mus musculus*** | | | | | |
| xRead | -k 15 -l 11 -w 5 -x 3 -X 10 -t 24 -M 24 | 24 | 12.94 (h) | 243.92 (h) | 14.89 |
| MHAP | -settings 2 --num-threads 24 | 24 | - | - | - |
| MECAT2 | -outfmt paf -num_threads 24 | 24 | 31.62 (h) | 720.70 (h) | 73.29 |
| minimap2 | -x ava-ont -t 24 | 24 | 42.07 (h) | 868.69 (h) | 51.95 |
| wtdbg2 | -p 19 -AS 2 -s 0.05 -L 5000 -t 24 | 24 | 35.26 (h) | 816.68 (h) | 292.80 |
| BLEND | -x ava-ont -t 24 | 24 | 40.38 (h) | 370.16 (h) | 36.97 |
| ***Homo sapiens*** | | | | | |
| xRead | -k 15 -l 11 -w 5 -x 3 -X 10 -t 24 -M 24 | 24 | 18.68 (h) | 398.05 (h) | 17.94 |
| MHAP | -settings 2 --num-threads 24 | 24 | - | - | - |
| MECAT2 | -outfmt paf -num_threads 24 | 24 | 59.57 (h) | 1362.27 (h) | 72.31 |
| minimap2 | -x ava-ont -t 24 | 24 | 50.15 (h) | 1132.15 (h) | 56.10 |
| wtdbg2 | -p 19 -AS 2 -s 0.05 -L 5000 -t 24 | 24 | 45.09 (h) | 1060.82 (h) | 321.74 |
| BLEND | -x ava-ont -t 24 | 24 | 48.79 (h) | 511.12 (h) | 32.62 |
| ***Ambystoma mexicanum* ^e^** | | | | | |
| xRead | -k 15 -l 11 -w 5 -x 3 -X 10 -t 64 -M 64 | 64 | 465.16 (h) | 24792.34 (h) | 59.446 |
| MHAP | -settings 2 --num_threads 64 | 64 | - | - | - |
| MECAT2 | -outfmt paf -num_threads 64 | 64 | 1005.52 (h) | 60233.28 (h) | 172.460 |
| minimap2 | -x ava-ont -t 64 | 64 | - | - | - |
| wtdbg2 | -p 19 -AS 2 -s 0.05 -L 5000 -t 64 | 64 | - | - | - |
| BLEND | -x ava-ont -t 64 | 64 | - | - | - |
| **Simulated ONT datasets with an average accuracy of 94%** | | | | | |
| ***Escherichia coli*** | | | | | |
| xRead | -k 15 -l 11 -w 13 -x 3 -X 10 -t 8 -M 16 | 8 | 9.13 | 18.25 | 0.34 |
| MHAP | --settings 2 --num-threads 8 | 8 | 127.20 | 819.53 | 32.38 |
| MECAT2 | -outfmt paf -num_threads 8 | 8 | 18.14 | 97.90 | 2.22 |
| minimap2 | -x ava-ont -w 13 -t 8 | 8 | 46.40 | 250.66 | 1.35 |
| wtdbg2 | -p 0 -k 15 -AS 2 -s 0.05 -L 5000 -t 8 | 8 | 59.00 | 395.86 | 1.01 |
| BLEND | -x ava-ont -t 8 | 8 | 57.14 | 316.69 | 2.08 |
| ***Arabidopsis thaliana*** | | | | | |
| xRead | -k 15 -l 11 -w 13 -x 3 -X 10 -t 8 -M 16 | 8 | 275.99 | 1115.97 | 4.24 |
| MHAP | --settings 2 --num-threads 8 | 8 | 4307.25 | 28996.55 | 80.36 |
| MECAT2 | -outfmt paf -num_threads 8 | 8 | 1006.88 | 6944.62 | 30.72 |
| minimap2 | -x ava-ont -w 13 -t 8 | 8 | 1846.11 | 11988.96 | 14.15 |
| wtdbg2 | -p 0 -k 15 -AS 2 -s 0.05 -L 5000 -t 8 | 8 | 3761.03 | 27368.13 | 21.78 |
| BLEND | -x ava-ont -t 8 | 8 | 1810.42 | 12011.89 | 19.07 |
| ***Drosophila melanogaster*** | | | | | |
| xRead | -k 15 -l 11 -w 13 -x 3 -X 10 -t 8 -M 16 | 8 | 395.48 | 1844.23 | 4.57 |
| MHAP | --settings 2 --num-threads 8 | 8 | 11696.79 | 85215.97 | 96.05 |
| MECAT2 | -outfmt paf -num_threads 8 | 8 | 1235.75 | 8688.84 | 30.65 |
| minimap2 | -x ava-ont -w 13 -t 8 | 8 | 1995.05 | 12786.13 | 18.58 |
| wtdbg2 | -p 0 -k 15 -AS 2 -s 0.05 -L 5000 -t 8 | 8 | 5289.25 | 39490.57 | 25.48 |
| BLEND | -x ava-ont -t 8 | 8 | 1779.90 | 11445.69 | 27.03 |
| ***Homo sapiens*** | | | | | |
| xRead | -k 15 -l 11 -w 13 -x 3 -X 10 -t 24 -M 24 | 24 | 9.42 (h) | 180.97 (h) | 14.63 |
| MHAP | -settings 2 --num-threads 24 | 24 | - | - | - |
| MECAT2 | -outfmt paf -num_threads 24 | 24 | 50.19 (h) | 1161.82 (h) | 73.24 |
| minimap2 | -x ava-ont -w 13 -t 24 | 24 | 68.65 (h) | 1069.82 (h) | 24.37 |
| wtdbg2 | -p 19 -AS 2 -s 0.05 -L 5000 -t 24 | 24 | 71.39 (h) | 1656.81 (h) | 336.11 |
| BLEND | -x ava-ont -t 24 | 24 | 55.65 (h) | 674.17 (h) | 41.85 |

(a) The parameters of the tools used for benchmarking on the simulated datasets.

(b) The number of CPU threads used for benchmarking on the simulated datasets.

(c) The real-time and CPU time of the tools cost on the simulated datasets, the results marked by “h” indicates CPU hours, otherwise, CPU seconds.

(d) The memory footprints of the tools (in GB) on the simulated datasets.

(e) Only xRead and MECAT2 finished the *A. mexicanum* dataset. Other tools failed due to being out-of-memory or very large time costs.

‘-’ indicates that the result of the tool is not available for the dataset.

The average accuracy of the first nine simulated datasets is 87%, and that of the last four is 94%.

# Supplementary Table 4. The yields of various tools on simulated datasets

| **Tool** | **Parameter ^a^** | **Preci-sion ^b^** | **Sensiti-vity ^b^** | **R% ^c^** | **C% ^d^** | **Gap Num. ^e^** | **Con.**  **Num. ^f^** |
| --- | --- | --- | --- | --- | --- | --- | --- |
| **Simulated ONT datasets with an average accuracy of 87%** | | | | | | | |
|  | ***Escherichia coli*** |  |  |  |  |  |  |
| xRead | -k 15 -l 11 -w 5 -x 3 -X 10 -t 8 -M 16 | 99.939 | 4.339 | 99.668 | 99.989 | 0 | 1 |
| MHAP | --settings 2 --num-threads 8 | 94.437 | 52.282 | 95.981 | 99.989 | 0 | 1 |
| MECAT2 | -outfmt paf -num_threads 8 | 99.760 | 5.697 | 97.022 | 99.989 | 0 | 1 |
| minimap2 | -x ava-ont -t 8 | 97.160 | 94.787 | 99.974 | 99.989 | 0 | 1 |
| wtdbg2 | -p 0 -k 15 -AS 2 -s 0.05 -L 5000 -t 8 | 91.585 | 79.824 | 96.118 | 99.989 | 0 | 1 |
| BLEND | -x ava-ont -t 8 | 99.414 | 83.152 | 98.550 | 99.989 | 0 | 1 |
|  | ***Saccharomyces cerevisiae*** |  |  |  |  |  |  |
| xRead | -k 15 -l 11 -w 5 -x 3 -X 10 -t 8 -M 16 | 99.219 | 4.177 | 99.448 | 99.875 | 1 | 2 |
| MHAP | --settings 2 --num-threads 8 | 68.494 | 54.348 | 96.218 | 99.875 | 1 | 1 |
| MECAT2 | -outfmt paf -num_threads 8 | 99.151 | 5.641 | 97.347 | 99.872 | 2 | 4 |
| minimap2 | -x ava-ont -t 8 | 89.861 | 94.681 | 99.958 | 99.875 | 1 | 1 |
| wtdbg2 | -p 0 -k 15 -AS 2 -s 0.05 -L 5000 -t 8 | 54.065 | 78.933 | 96.100 | 99.872 | 2 | 2 |
| BLEND | -x ava-ont -t 8 | 96.811 | 83.397 | 98.570 | 99.872 | 0 | 2 |
|  | ***Caenorhabditis elegans*** |  |  |  |  |  |  |
| xRead | -k 15 -l 11 -w 5 -x 3 -X 10 -t 8 -M 16 | 99.709 | 4.295 | 99.645 | 99.993 | 0 | 3 |
| MHAP | --settings 2 --num-threads 8 | 88.509 | 56.490 | 96.599 | 99.993 | 0 | 2 |
| MECAT2 | -outfmt paf -num_threads 8 | 96.017 | 5.865 | 97.812 | 99.993 | 0 | 4 |
| minimap2 | -x ava-ont -t 8 | 46.376 | 95.402 | 99.978 | 99.993 | 0 | 2 |
| wtdbg2 | -p 0 -k 15 -AS 2 -s 0.05 -L 5000 -t 8 | 81.821 | 80.379 | 96.198 | 99.993 | 0 | 2 |
| BLEND | -x ava-ont -t 8 | 87.200 | 84.419 | 98.848 | 99.993 | 0 | 2 |
|  | ***Arabidopsis thaliana*** |  |  |  |  |  |  |
| xRead | -k 15 -l 11 -w 5 -x 3 -X 10 -t 8 -M 16 | 99.709 | 4.295 | 99.645 | 99.993 | 0 | 1 |
| MHAP | --settings 2 --num-threads 8 | 88.509 | 56.490 | 96.599 | 99.993 | 0 | 1 |
| MECAT2 | -outfmt paf -num_threads 8 | 96.017 | 5.865 | 97.812 | 99.993 | 0 | 10 |
| minimap2 | -x ava-ont -t 8 | 46.376 | 95.402 | 99.978 | 99.993 | 0 | 1 |
| wtdbg2 | -p 0 -k 15 -AS 2 -s 0.05 -L 5000 -t 8 | 81.821 | 80.379 | 96.198 | 99.993 | 2 | 1 |
| BLEND | -x ava-ont -t 8 | 81.952 | 83.135 | 98.656 | 99.815 | 139 | 9 |
|  | ***Drosophila melanogaster*** |  |  |  |  |  |  |
| xRead | -k 15 -l 11 -w 5 -x 3 -X 10 -t 8 -M 16 | 91.126 | 4.095 | 89.684 | 99.993 | 1 | 861 |
| MHAP | --settings 2 --num-threads 8 | 6.585 | 58.543 | 96.567 | 99.994 | 0 | 2 |
| MECAT2 | -outfmt paf -num_threads 8 | 80.760 | 6.498 | 95.006 | 99.993 | 1 | 39 |
| minimap2 | -x ava-ont -t 8 | 22.938 | 90.940 | 96.926 | 99.994 | 0 | 477 |
| wtdbg2 | -p 0 -k 15 -AS 2 -s 0.05 -L 5000 -t 8 | 34.405 | 68.934 | 80.747 | 99.992 | 3 | 9 |
| BLEND | -x ava-ont -t 8 | 61.278 | 75.649 | 91.368 | 99.666 | 350 | 422 |
|  | ***Zea mays (SK)*** |  |  |  |  |  |  |
| xRead | -k 15 -l 11 -w 5 -x 3 -X 10 -t 24 -M 24 | 87.828 | 3.091 | 95.855 | 100.0 | 0 | 20 |
| MHAP | -settings 2 --num-threads 24 | - | - | - | - | - | - |
| MECAT2 | -outfmt paf -num_threads 24 | 15.161 | 11.835 | 96.052 | 100.0 | 0 | 5 |
| minimap2 | -x ava-ont -t 24 | 1.516 | 92.374 | 99.876 | 100.0 | 0 | 1 |
| wtdbg2 | -p 19 -AS 2 -s 0.05 -L 5000 -t 24 | 4.446 | 36.434 | 92.978 | 100.0 | 4 | 16 |
| BLEND | -x ava-ont -t 24 | 1.440 | 76.921 | 97.466 | 99.988 | 763 | 4 |
|  | ***Mus musculus*** |  |  |  |  |  |  |
| xRead | -k 15 -l 11 -w 5 -x 3 -X 10 -t 24 -M 24 | 94.948 | 3.952 | 96.210 | 100.0 | 2 | 47 |
| MHAP | -settings 2 --num-threads 24 | - | - | - | - | - | - |
| MECAT2 | -outfmt paf -num_threads 24 | 32.779 | 9.072 | 88.857 | 100.0 | 0 | 405 |
| minimap2 | -x ava-ont -t 24 | 4.482 | 90.418 | 99.913 | 100.0 | 0 | 6 |
| wtdbg2 | -p 19 -AS 2 -s 0.05 -L 5000 -t 24 | 44.502 | 68.809 | 94.710 | 100.0 | 1 | 6 |
| BLEND | -x ava-ont -t 24 | 26.335 | 77.802 | 98.278 | 99.992 | 356 | 50 |
|  | ***Homo sapiens*** |  |  |  |  |  |  |
| xRead | -k 15 -l 11 -w 5 -x 3 -X 10 -t 24 -M 24 | 92.360 | 3.907 | 95.070 | 100.0 | 0 | 18 |
| MHAP | -settings 2 --num-threads 24 | - | - | - | - | - | - |
| MECAT2 | -outfmt paf -num_threads 24 | 40.118 | 9.705 | 95.657 | 100.0 | 0 | 28 |
| minimap2 | -x ava-ont -t 24 | 9.355 | 91.781 | 98.548 | 99.998 | 7 | 562 |
| wtdbg2 | -p 19 -AS 2 -s 0.05 -L 5000 -t 24 | 62.008 | 65.694 | 88.711 | 100.0 | 0 | 284 |
| BLEND | -x ava-ont -t 24 | 49.584 | 79.403 | 96.675 | 99.930 | 246 | 258 |
|  | ***Ambystoma mexicanum* ^g^** |  |  |  |  |  |  |
| xRead | -k 15 -l 11 -w 5 -x 3 -X 10 -t 64 -M 64 | 91.848 | 5.690 | 98.407 | 99.999 | 1 | 862 |
| MHAP | -settings 2 --num_threads 64 | - | - | - | - | - | - |
| MECAT2 | -outfmt paf -num_threads 64 | 4.879 | 26.944 | 97.334 | 99.999 | 206 | 22 |
| minimap2 | -x ava-ont -t 64 | - | - | - | - | - | - |
| wtdbg2 | -p 19 -AS 2 -s 0.05 -L 5000 -t 64 | - | - | - | - | - | - |
| BLEND | -x ava-ont -t 64 | - | - | - | - | - | - |
| **Simulated ONT datasets with an average accuracy of 94%** | | | | | | | |
|  | ***Escherichia coli*** |  |  |  |  |  |  |
| xRead | -k 15 -l 11 -w 13 -x 3 -X 10 -t 8 -M 16 | 99.895 | 4.279 | 99.910 | 99.995 | 0 | 1 |
| MHAP | --settings 2 --num-threads 8 | 73.146 | 93.317 | 99.917 | 99.995 | 0 | 1 |
| MECAT2 | -outfmt paf -num_threads 8 | 99.694 | 6.001 | 99.936 | 99.995 | 0 | 1 |
| minimap2 | -x ava-ont -w 13 -t 8 | 92.055 | 99.191 | 99.981 | 99.995 | 0 | 1 |
| wtdbg2 | -p 0 -k 15 -AS 2 -s 0.05 -L 5000 -t 8 | 89.289 | 85.867 | 96.676 | 99.995 | 0 | 1 |
| BLEND | -x ava-ont -t 8 | 94.340 | 98.909 | 99.923 | 99.995 | 0 | 1 |
|  | ***Arabidopsis thaliana*** |  |  |  |  |  |  |
| xRead | -k 15 -l 11 -w 13 -x 3 -X 10 -t 8 -M 16 | 98.870 | 4.240 | 99.685 | 99.831 | 3 | 3 |
| MHAP | --settings 2 --num-threads 8 | 23.673 | 93.602 | 99.939 | 99.831 | 3 | 1 |
| MECAT2 | -outfmt paf -num_threads 8 | 89.041 | 6.580 | 99.863 | 99.831 | 3 | 2 |
| minimap2 | -x ava-ont -w 13 -t 8 | 23.758 | 99.243 | 99.994 | 99.831 | 3 | 1 |
| wtdbg2 | -p 0 -k 15 -AS 2 -s 0.05 -L 5000 -t 8 | 43.167 | 83.188 | 96.457 | 99.828 | 4 | 1 |
| BLEND | -x ava-ont -t 24 | 35.384 | 98.286 | 99.965 | 99.831 | 3 | 3 |
|  | ***Drosophila melanogaster*** |  |  |  |  |  |  |
| xRead | -k 15 -l 11 -w 13 -x 3 -X 10 -t 8 -M 16 | 89.508 | 4.128 | 90.791 | 99.673 | 0 | 476 |
| MHAP | --settings 2 --num-threads 8 | 4.033 | 94.143 | 99.923 | 99.673 | 0 | 1 |
| MECAT2 | -outfmt paf -num_threads 8 | 77.673 | 7.113 | 98.857 | 99.673 | 0 | 3 |
| minimap2 | -x ava-ont -w 13 -t 8 | 15.192 | 97.196 | 98.970 | 99.673 | 0 | 284 |
| wtdbg2 | -p 0 -k 15 -AS 2 -s 0.05 -L 5000 -t 8 | 35.062 | 73.673 | 81.656 | 99.661 | 1 | 4 |
| BLEND | -x ava-ont -t 8 | 21.976 | 95.428 | 99.223 | 99.673 | 0 | 259 |
|  | ***Homo sapiens*** |  |  |  |  |  |  |
| xRead | -k 15 -l 11 -w 13 -x 3 -X 10 -t 24 -M 24 | 94.323 | 4.075 | 96.655 | 100.0 | 0 | 88 |
| MHAP | -settings 2 --num-threads 24 | - | - | - | - | - | - |
| MECAT2 | -outfmt paf -num_threads 24 | 22.889 | 10345 | 98.717 | 100.0 | 0 | 8 |
| minimap2 | -x ava-ont -w 13 -t 24 | 4.943 | 96.023 | 99.068 | 100.0 | 1 | 22 |
| wtdbg2 | -p 19 -AS 2 -s 0.05 -L 5000 -t 24 | 38.957 | 79.166 | 96.182 | 100.0 | 1 | 16 |
| BLEND | -x ava-ont -t 24 | 8.860 | 95.874 | 99.026 | 99.999 | 2 | 68 |

(a) The parameters of the tools used for benchmarking on the simulated datasets.

(b) The precision and sensitivity of the tools on the simulated datasets.

(c) The proportion of the reads having at least one ground truth overlap being recovered.

(d) The percentage of the donor genome being covered by the connected reads.

(e) The number of gaps in the donor genome.

(f) The number of connected components of produced graphs.

(g) Only xRead and MECAT2 finished the *A. mexicanum* dataset. Other tools failed due to out-of-memory or very large time costs.

‘-’ indicates that the result of the tool is not available for the dataset.

The average accuracy of the first nine simulated datasets is 87%, and that of the last four is 94%.

# Supplementary Table 5. The sensitivity of the expanded graphs of xRead on simulated datasets

| **Datasets** | **Outputs of xRead** | | **1 Iteration ^a^** | | **3 Iterations ^a^** | | **5 Iterations ^a^** | |
| --- | --- | --- | --- | --- | --- | --- | --- | --- |
|  | **Sensitivity** | **R%** | **Sensitivity** | **R%** | **Sensitivity** | **R%** | **Sensitivity** | **R%** |
| ***E. coli* ^b^** | 4.339 | 99.668 | 87.301 | 99.668 | 98.689 | 99.668 | 98.723 | 99.668 |
| ***S. cerevisiae* ^b^** | 4.177 | 99.448 | 86.211 | 99.628 | 98.263 | 99.660 | 98.373 | 99.670 |
| ***C. elegans* ^b^** | 4.295 | 99.645 | 85.536 | 99.738 | 98.767 | 99.743 | 98.833 | 99.743 |
| ***A. thaliana* ^b^** | 4.287 | 99.472 | 85.841 | 99.681 | 98.365 | 99.690 | 98.468 | 99.691 |
| ***D. melanogaster* ^b^** | 4.095 | 89.684 | 77.921 | 95.974 | 93.377 | 96.287 | 93.962 | 96.337 |
| ***Z. mays (SK)* ^b^** | 3.091 | 95.855 | 78.712 | 98.037 | 92.887 | 98.794 | 94.472 | 98.922 |
| ***M. musculus* ^b^** | 3.952 | 96.210 | 79.968 | 99.238 | 93.608 | 99.412 | 94.903 | 99.490 |
| ***H. sapiens* ^b^** | 3.907 | 95.070 | 81.293 | 98.667 | 96.137 | 99.585 | 97.859 | 99.673 |
| ***A. mexicanum* ^b^** | 5.690 | 98.407 | 83.322 | 99.045 | 87.926 | 99.588 | 88.747 | 99.713 |
| ***E. coli* ^c^** | 4.279 | 99.910 | 89.691 | 99.923 | 99.145 | 99.930 | 99.200 | 99.930 |
| ***A. thaliana* ^c^** | 4.240 | 99.685 | 99.338 | 99.954 | 99.080 | 99.962 | 99.239 | 99.964 |
| ***D. melanogaster* ^c^** | 4.128 | 90.791 | 82.014 | 98.980 | 95.824 | 99.174 | 96.476 | 99.217 |
| ***H. sapiens* ^c^** | 4.075 | 96.655 | 84.309 | 99.448 | 94.470 | 99.628 | 98.312 | 99.668 |

(a) The results of the graphs expanded by 1, 3 and 5 iterations, respectively.

(b) The simulated ONT datasets with average accuracy of 87%.

(c) The simulated high quality ONT datasets with average accuracy of 94%.

# Supplementary Table 6. The performance of various tools on real sequencing datasets

| **Tool** | **Parameter ^a^** | **Thread ^b^** | **Real time ^c^** | **CPU time ^c^** | **Memory ^d^** |
| --- | --- | --- | --- | --- | --- |
| ***Escherichia coli*** | | | | | |
| xRead | -k 15 -l 11 -w 5 -x 3 -X 10 -t 8 -M 16 | 8 | 14.59 | 53.391 | 0.61 |
| MHAP | --setting 2 --num-threads 8 | 8 | 214.92 | 1546.38 | 14.11 |
| MECAT2 | -outfmt paf -num_threads 8 | 8 | 46.58 | 275.91 | 3.28 |
| minimap2 | -x ava-ont -t 8 | 8 | 194.39 | 1171.73 | 5.43 |
| wtdbg2 | -p 0 -k 15 -AS 2 -s 0.05 -L 5000 -t 8 | 8 | 142.33 | 1009.05 | 2.51 |
| BLEND | -x ava-ont -t 8 | 8 | 64.79 | 355.28 | 3.81 |
| ***Caenorhabditis elegans*** | | | | | |
| xRead | -k 15 -l 11 -w 5 -x 3 -X 10 -t 8 -M 16 | 8 | 1531.94 | 10880.96 | 6.08 |
| MHAP | --setting 2 --num-threads 8 | 8 | 22368.01 | 174366.67 | 112.15 |
| MECAT2 | -outfmt paf -num_threads 8 | 8 | 3632.01 | 26397.74 | 30.88 |
| minimap2 | -x ava-ont -t 8 | 8 | 17944.82 | 125949.93 | 47.66 |
| wtdbg2 | -p 0 -k 15 -AS 2 -s 0.05 -L 5000 -t 8 | 8 | 19511.99 | 151825.74 | 36.43 |
| BLEND | -x ava-ont -t 8 | 8 | 2668.17 | 19432.11 | 32.96 |
| ***Drosophila melanogaster*** | | | | | |
| xRead | -k 15 -l 11 -w 5 -x 3 -X 10 -t 8 -M 16 | 8 | 1324.97 | 9421.72 | 8.22 |
| MHAP | --setting 2 --num-threads 8 | 8 | 29380.11 | 202662.45 | 102.28 |
| MECAT2 | -outfmt paf -num_threads 8 | 8 | 1680.91 | 11798.05 | 35.11 |
| minimap2 | -x ava-ont -t 8 | 8 | 5995.24 | 43179.38 | 39.45 |
| wtdbg2 | -p 0 -k 15 -AS 2 -s 0.05 -L 5000 -t 8 | 8 | 5469.43 | 43568.98 | 25.67 |
| BLEND | -x ava-ont -t 8 | 8 | 1248.93 | 8624.59 | 29.34 |
| ***Homo sapiens (ONT fast mode)*** | | | | | |
| xRead | -k 15 -l 11 -w 5 -x 5 -X 10 -t 24 -M 24 | 24 | 23.79 (h) | 459.06 (h) | 16.95 |
| MHAP | -settings 2 --num-threads 24 | 24 | - | - | - |
| MECAT2 | -outfmt paf -num_threads 24 | 24 | 20.03 (h) | 434.23 (h) | 76.38 |
| minimap2 | -x ava-ont -t 24 | 24 | 43.90 (h) | 878.73 (h) | 62.74 |
| wtdbg2 | -p 19 -AS 2 -s 0.05 -L 5000 -t 24 | 24 | 26.35 (h) | 611.63 (h) | 157.78 |
| BLEND | -x ava-ont -t 24 | 24 | 9.79 (h) | 198.91 (h) | 39.17 |
| ***Homo sapiens (PacBio HiFi)*** | | | | | |
| xRead | -k 19 -l 11 -w 40 -x 5 -X 10 -t 24 -M 24 | 24 | 2.97 (h) | 13.53 (h) | 9.27 |
| MHAP | -settings 2 --num-threads 24 | 24 | - | - | - |
| MECAT2 | -outfmt paf -num_threads 24 | 24 | 27.92 (h) | 632.57 (h) | 68.47 |
| minimap2 | -x ava-pb -t 24 | 24 | 75.76 (h) | 1101.28 (h) | 33.21 |
| wtdbg2 | -p 21 -k 0 -AS 4 -K 0.05 -s 0.5 -t 24 | 24 | 12.48 (h) | 281.72 (h) | 110.75 |
| BLEND | -x ava-hifi -t 24 | 24 | 10.27 (h) | 17.69 (h) | 3.98 |
| ***Homo sapiens (ONT super high accuracy mode)*** | | | | | |
| xRead | -k 19 -l 11 -w 40 -x 3 -X 10 -t 24 -M 24 | 24 | 14.03 (h) | 107.25 (h) | 11.64 |
| MHAP | -settings 2 --num-threads 24 | 24 | - | - | - |
| MECAT2 | -outfmt paf -num_threads 24 | 24 | 134.55 (h) | 3139.62 (h) | 70.12 |
| minimap2 | -x ava-ont -t 24 | 24 | 416.37 (h) | 74571.88 (h) | 53.47 |
| wtdbg2 | -p 21 -k 0 -AS 4 -K 0.05 -s 0.5 -t 24 | 24 | 42.72 (h) | 969.02 (h) | 316.75 |
| BLEND | -x ava-ont -t 24 | 24 | 102.70 (h) | 2165.52 (h) | 38.96 |
| ***Ambystoma mexicanum* ^e^** | | | | | |
| xRead | -k 15 -l 11 -w 5 -x 5 -X 10 -t 64 -M 64 | 64 | 385.92 (h) | 14731.77 (h) | 57.12 |
| MHAP | -settings 2 --num-threads 64 | 64 | - | - | - |
| MECAT2 | -outfmt paf -num_threads 64 | 64 | - | - | - |
| minimap2 | -x ava-pb -t 64 | 64 | - | - | - |
| wtdbg2 | -p 19 -AS 2 -s 0.05 -L 5000 -t 64 | 64 | - | - | - |
| BLEND | -x ava-pb -t 64 | 64 | - | - | - |

(a) The parameters of the tools used for benchmarking on the real datasets.

(b) The number of CPU threads used for benchmarking on the real datasets.

(c) The real-time and CPU time of the tools cost on the real datasets, the results marked by “h” indicates CPU hours, otherwise, CPU seconds.

(d) The memory footprints of the tools (in GB) on the real datasets.

(e) Only xRead finished the *A. mexicanum* dataset. Other tools failed due to out-of-memory, segmentation fault or very large time cost.

‘-’ indicates that the result of the tool is not available for the dataset.

# Supplementary Table 7. The yields of various tools on real sequencing datasets

| **Tool** | **Parameter ^a^** | **Preci-sion ^b^** | **Sensiti-vity ^b^** | **R% ^c^** | **C% ^d^** | **Gap Num. ^e^** | **Con.**  **Num. ^f^** |
| --- | --- | --- | --- | --- | --- | --- | --- |
|  | ***E. coli*** |  |  |  |  |  |  |
| xRead | -k 15 -l 11 -w 5 -x 3 -X 10 -t 8 -M 16 | 99.795 | 2.935 | 98.810 | 100.0 | 0 | 55 |
| MHAP | --setting 2 --num-threads 8 | 74.929 | 91.140 | 99.907 | 100.0 | 0 | 65 |
| MECAT2 | -outfmt paf -num_threads 8 | 99.811 | 3.706 | 99.764 | 100.0 | 0 | 87 |
| minimap2 | -x ava-ont -t 8 | 93.641 | 96.968 | 99.997 | 100.0 | 0 | 17 |
| wtdbg2 | -p 0 -k 15 -AS 2 -s 0.05 -L 5000 -t 8 | 91.774 | 76.700 | 91.252 | 100.0 | 0 | 60 |
| BLEND | -x ava-ont -t 8 | 97.362 | 97.315 | 99.900 | 100.0 | 0 | 31 |
|  | ***C. elegans*** |  |  |  |  |  |  |
| xRead | -k 15 -l 11 -w 5 -x 3 -X 10 -t 8 -M 16 | 99.603 | 2.412 | 98.913 | 100.0 | 0 | 227 |
| MHAP | --setting 2 --num-threads 8 | 57.981 | 85.074 | 98.947 | 100.0 | 0 | 271 |
| MECAT2 | -outfmt paf -num_threads 8 | 99.553 | 5.458 | 98.289 | 100.0 | 0 | 54 |
| minimap2 | -x ava-ont -t 8 | 36.595 | 95.287 | 99.987 | 100.0 | 0 | 636 |
| wtdbg2 | -p 0 -k 15 -AS 2 -s 0.05 -L 5000 -t 8 | 77.976 | 69.450 | 83.782 | 100.0 | 0 | 72 |
| BLEND | -x ava-ont -t 8 | 61.294 | 92.228 | 99.583 | 100.0 | 0 | 19 |
|  | ***D. melanogaster*** |  |  |  |  |  |  |
| xRead | -k 15 -l 11 -w 5 -x 3 -X 10 -t 8 -M 16 | 98.183 | 4.217 | 98.226 | 100.0 | 0 | 145 |
| MHAP | --setting 2 --num-threads 8 | 23.767 | 81.457 | 99.922 | 100.0 | 0 | 9 |
| MECAT2 | -outfmt paf -num_threads 8 | 97.482 | 6.816 | 99.012 | 100.0 | 0 | 40 |
| minimap2 | -x ava-ont -t 8 | 23.052 | 78.785 | 99.854 | 100.0 | 0 | 41 |
| wtdbg2 | -p 0 -k 15 -AS 2 -s 0.05 -L 5000 -t 8 | 35.041 | 39.034 | 69.496 | 99.998 | 3 | 11 |
| BLEND | -x ava-ont -t 8 | 46.944 | 73.980 | 98.491 | 100.0 | 0 | 15 |
|  | ***H. sapiens (ONT fast mode)*** |  |  |  |  |  |  |
| xRead | -k 15 -l 11 -w 5 -x 5 -X 10 -t 24 -M 24 | 99.264 | 7.214 | 97.642 | 99.995 | 20 | 2530 |
| MHAP | -settings 2 --num-threads 24 | - | - | - | - | - | - |
| MECAT2 | -outfmt paf -num_threads 24 | 90.847 | 55.528 | 98.820 | 99.991 | 26 | 1182 |
| minimap2 | -x ava-ont -t 8 | 7.013 | 90.565 | 99.728 | 100.0 | 2 | 471 |
| wtdbg2 | -p 19 -AS 2 -s 0.05 -L 5000 -t 24 | 54.880 | 26.899 | 43.057 | 99.974 | 197 | 44 |
| BLEND | -x ava-ont -t 24 | 49.069 | 82.793 | 99.320 | 99.994 | 41 | 18 |
|  | ***H. sapiens (PacBio HiFi)*** |  |  |  |  |  |  |
| xRead | -k 19 -l 11 -w 40 -x 5 -X 10 -t 24 -M 24 | 99.061 | 7.426 | 99.589 | 99.999 | 3 | 58 |
| MHAP | -settings 2 --num-threads 24 | - | - | - |  | - | - |
| MECAT2 | -outfmt paf -num_threads 24 | 85.673 | 70.471 | 99.982 | 100.0 | 0 | 1 |
| minimap2 | -x ava-pb -t 24 | 1.703 | 95.709 | 99.988 | 100.0 | 0 | 2 |
| wtdbg2 | -p 21 -k 0 -AS 4 -K 0.05 -s 0.5 -t 24 | 96.208 | 77.871 | 99.885 | 99.999 | 2 | 861 |
| BLEND | -x ava-hifi -t 24 | 98.902 | 62.516 | 99.131 | 99.989 | 45 | 9 |
|  | ***H. sapiens (ONT super high accuracy mode)*** | |  |  |  |  |  |
| xRead | -k 19 -l 11 -w 40 -x 3 -X 10 -t 24 -M 24 | 97.532 | 3.085 | 97.887 | 99.996 | 72 | 2248 |
| MHAP | -settings 2 --num-threads 24 | - | - | - | - | - | - |
| MECAT2 | -outfmt paf -num_threads 24 | 85.836 | 65.911 | 99.955 | 100.0 | 1 | 120 |
| minimap2 | -x ava-ont -t 24 | 1.353 | 94.067 | 99.850 | 99.999 | 26 | 269 |
| wtdbg2 | -p 21 -k 0 -AS 4 -K 0.05 -s 0.5 -t 24 | 95.093 | 64.328 | 89.841 | 99.979 | 161 | 1450 |
| BLEND | -x ava-ont -t 24 | 9.417 | 89.817 | 99.997 | 99.999 | 68 | 175 |
|  | ***A. mexicanum ^g^*** |  |  |  |  |  |  |
| xRead | -k 15 -l 11 -w 5 -x 5 -X 10 -t 64 -M 64 | 86.286 | 2.193 | 92.080 | 99.992 | 1 | 28777 |
| MHAP | -settings 2 --num-threads 64 | - | - | - | - | - | - |
| MECAT2 | -outfmt paf -num_threads 64 | - | - | - | - | - | - |
| minimap2 | -x ava-pb -t 64 | - | - | - | - | - | - |
| wtdbg2 | -p 19 -AS 2 -s 0.05 -L 5000 -t 64 | - | - | - | - | - | - |
| BLEND | -x ava-pb -t 64 | - | - | - | - | - | - |

(a) The parameters of the tools used for benchmarking on the simulated datasets.

(b) The precision and sensitivity of the tools on the simulated datasets.

(c) The proportion of the reads having at least one ground truth overlap being recovered.

(d) The percentage of the donor genome being covered by the connected reads.

(e) The number of gaps in the donor genome.

(f) The number of connected components of produced graphs.

(g) Only xRead finished the *A. mexicanum* dataset. Other tools failed due to out-of-memory, segmentation fault or very large time cost.

‘-’ indicates that the result of the tool is not available for the dataset.

# Supplementary Table 8. Percentages of the reads not correctly overlapped by xRead with various causes

| **Datasets** | **R% of xRead** | **R_1_% ^d^** | **R_2_% ^e^** | **R_3_% ^f^** |
| --- | --- | --- | --- | --- |
| ***E. coli*** | 98.810 | 0.000 | 0.028 | 1.162 |
| ***C. elegans*** | 98.913 | 0.014 | 0.052 | 1.021 |
| ***D. melanogaster*** | 98.226 | 0.040 | 1.004 | 0.730 |
| ***H. sapiens ^a^*** | 97.642 | 0.077 | 0.474 | 1.807 |
| ***H. sapiens ^b^*** | 99.589 | 0.179 | 0.193 | 0.039 |
| ***H. sapiens ^c^*** | 97.887 | 0.354 | 0.788 | 0.971 |
| ***A. mexicanum*** | 92.080 | 2.516 | 1.522 | 3.882 |

(a) The human datasets in ONT fast base-calling modes.

(b) The PacBio HiFi human dataset.

(c) The human datasets in ONT super high accuracy base-calling modes.

(d) The percentage of the reads having not been overlapped.

(e) The percentage of the reads having only false positive overlaps.

(f) The percentage of the reads being overlapped with other reads not included in the pseudo-ground truth set.

# Supplementary Table 9. The sensitivity of the expanded graphs of xRead on real sequencing datasets

| **Datasets** | **Outputs of xRead** | | **1 Iteration ^d^** | | **3 Iterations ^d^** | | **5 Iterations ^d^** | |
| --- | --- | --- | --- | --- | --- | --- | --- | --- |
|  | **Sensitivity** | **R%** | **Sensitivity** | **R%** | **Sensitivity** | **R%** | **Sensitivity** | **R%** |
| ***E. coli*** | 2.935 | 98.810 | 78.177 | 99.015 | 95.625 | 99.018 | 96.067 | 99.018 |
| ***C. elegans*** | 2.412 | 98.913 | 65.082 | 99.039 | 95.284 | 99.052 | 96.360 | 99.056 |
| ***D. melanogaster*** | 4.217 | 98.226 | 71.773 | 98.956 | 95.833 | 99.202 | 96.799 | 99.301 |
| ***H. sapiens ^a^*** | 7.214 | 97.642 | 71.776 | 98.114 | 88.651 | 98.192 | 92.293 | 98.193 |
| ***H. sapiens ^b^*** | 7.426 | 99.589 | 68.439 | 99.915 | 87.833 | 99.926 | 93.073 | 99.929 |
| ***H. sapiens ^c^*** | 3.085 | 97.887 | 49.947 | 98.480 | 86.241 | 98.542 | 88.710 | 98.554 |
| ***A. mexicanum*** | 2.193 | 92.080 | 40.705 | 92.974 | 65.296 | 93.650 | 87.110 | 94.021 |

(a) The human datasets in ONT fast base-calling modes.

(b) The PacBio HiFi human dataset.

(c) The human datasets in ONT super high accuracy base-calling modes.

(d) The results of the graphs expanded by 1, 3 and 5 iterations, respectively.

# Supplementary Table 10. The external memory of various tools on both simulated and real datasets.

| **Dataset** | **xRead** | **MHAP** | **MECAT2** | **Minimap2** | **Wtdbg2** | **BLEND** |
| --- | --- | --- | --- | --- | --- | --- |
| **Output Format ^a^** | **PAF** | **M4 format** | **PAF** | **PAF** | **alignment.gz** | **PAF** |
| **Simulated ONT datasets with an average accuracy of 87%** | | | | | | |
| ***E. coli*** | 854.0 (KB) | 9.2 (MB) | 1.3 (MB) | 22.0 (MB) | 16.0 (MB) | 19.0 (MB) |
| ***S. cerevisiae*** | 2.1 (MB) | 36.0 (MB) | 3.3 (MB) | 63.0 (MB) | 67.0 (MB) | 51.0 (MB) |
| ***C. elegans*** | 1.7 (MB) | 243.0 (MB) | 33.0 (MB) | 1.2 | 413.0 (MB) | 489.0 (MB) |
| ***A. thaliana*** | 23.0 (MB) | 339.0 (MB) | 42.0 (MB) | 1.7 | 746.0 (MB) | 609.0 (MB) |
| ***D. melanogaster*** | 30.0 (MB) | 5.0 | 64.0 (MB) | 3.5 | 1.3 | 974.0 (MB) |
| ***Z. mays (SK)*** | 470.0 (MB) | - | 9.9 | 4.5 (TB) | 75.0 | 783.5 |
| ***M. musculus*** | 533.0 (MB) | - | 4.1 | 261.0 | 19.0 | 39.0 |
| ***H. sapiens*** | 622.0 (MB) | - | 4.1 | 174.0 | 18.0 | 29.0 |
| ***A. mexicanum*** | 9.2 | - | 869.0 | - | - | - |
| **Simulated ONT datasets with an average accuracy of 94%** | | | | | | |
| ***E. coli*** | 744.0 (KB) | 22.0 (MB) | 1.4 (MB) | 25.0 (MB) | 16.0 (MB) | 25.0 (MB) |
| ***A. thaliana*** | 21.0 (MB) | 1.7 | 50.0 (MB) | 2.9 | 896.0 (MB) | 1.9 |
| ***D. melanogaster*** | 27.0 (MB) | 12.0 | 75.0 (MB) | 5.5 | 1.2 | 3.6 |
| ***H. sapiens*** | 555.0 (MB) | - | 7.4 | 304.0 | 25.0 | 168.0 |
| ***Real datasets*** | | | | | | |
| ***E. coli*** | 1.5 (MB) | 74.0 (MB) | 3.2 (MB) | 90.0 (MB) | 48.0 (MB) | 82.0 (MB) |
| ***C. elegans*** | 44.0 (MB) | 2.8 | 159.0 (MB) | 13.0 | 1.9 | 4.6 |
| ***D. melanogaster*** | 31.0 (MB) | 43.0 | 113.0 (MB) | 8.2 | 1.2 | 3.8 |
| ***H. sapiens ^b^*** | 447.0 (MB) | - | 12.0 | 396.0 | 9.6 | 78.0 |
| ***H. sapiens ^c^*** | 942.0 (MB) | - | 76.0 | 7.6 (TB) | 41.0 | 1.7 (TB) |
| ***H. sapiens ^d^*** | 388.0 (MB) | - | 7.3 | 374.0 | 6.2 | 212.0 |
| ***A. mexicanum*** | 4.9 | - | - | - | - | - |

(a) The format of various tools. Specifically, xRead, MECAT2, Minimap2 and BLEND use PAF format to store the overlap information in external memory. MHAP employs the BLASR’s M4 format, and Wtdbg2 generates files in its own alignment.gz format. All output files were zipped using gzip. It is worthnoting that tools, such as Wtdbg2, generate other files to store necessary information for downstream assembly. The external memory consumption of these extra information was excluded.

(b) The human datasets in ONT fast base-calling modes.

(c) The PacBio HiFi human dataset.

(d) The human datasets in ONT super high accuracy base-calling modes.

# Supplementary Table 11. The performance of de novo assembly on simulated datasets

| **Datasets** | **Tool** | **Thread ^b^** | **Real time ^c^** | **Peak Memory ^d^** |
| --- | --- | --- | --- | --- |
| **Simulated ONT datasets with an average accuracy of 87%** | | | | |
| ***E. coli*** | xRead-nd | 30 | 7.07 | 0.69 |
|  | xRead-wtpoa | 30 | 22.06 | 3.73 |
|  | NextDenovo | 30 | 30.95 | 1.62 |
|  | Wtdbg2 | 30 | 37.83 | 1.85 |
|  | Flye | 30 | 826.51 | 8.99 |
| ***A. thaliana*** | xRead-nd | 30 | 189.07 | 9.21 |
|  | xRead-wtpoa | 30 | 509.67 | 9.81 |
|  | NextDenovo | 30 | 536.75 | 10.05 |
|  | Wtdbg2 | 30 | 1283.40 | 22.74 |
|  | Flye | 30 | 2537.85 | 36.03 |
| ***D. melanogaster*** | xRead-nd | 30 | 235.10 | 9.89 |
|  | xRead-wtpoa | 30 | 681.41 | 11.54 |
|  | NextDenovo | 30 | 694.69 | 10.32 |
|  | Wtdbg2 | 30 | 1384.46 | 26.67 |
|  | Flye | 30 | 3126.81 | 51.97 |
| ***H. sapiens*** | xRead-nd | 30 | 10.89 (h) | 19.88 |
|  | xRead-wtpoa | 30 | 15.81 (h) | 30.18 |
|  | NextDenovo | 30 | 16.75 (h) | 225.26 |
|  | Wtdbg2 | 30 | 37.19 (h) | 321.34 |
|  | Flye | 30 | 50.34 (h) | 382.21 |
| **Simulated ONT datasets with an average accuracy of 94%** | | | | |
| ***E. coli*** | xRead-nd | 30 | 6.12 | 0.68 |
|  | xRead-wtpoa | 30 | 17.74 | 2.11 |
|  | NextDenovo | 30 | 38.49 | 1.66 |
|  | Wtdbg2 | 30 | 35.29 | 1.85 |
|  | Flye | 30 | 774.58 | 2.23 |
|  | Shasta | 30 | 23.85 | 1.23 |
| ***A. thaliana*** | xRead-nd | 30 | 153.66 | 7.95 |
|  | xRead-wtpoa | 30 | 436.48 | 9.42 |
|  | NextDenovo | 30 | 889.31 | 9.04 |
|  | Wtdbg2 | 30 | 1375.68 | 23.03 |
|  | Flye | 30 | 2419.66 | 21.64 |
|  | Shasta | 30 | 396.44 | 27.64 |
| ***D. melanogaster*** | xRead-nd | 30 | 203.36 | 9.03 |
|  | xRead-wtpoa | 30 | 603.97 | 16.00 |
|  | NextDenovo | 30 | 2884.57 | 9.91 |
|  | Wtdbg2 | 30 | 1533.69 | 26.85 |
|  | Flye | 30 | 3610.60 | 31.85 |
|  | Shasta | 30 | 455.08 | 31.47 |
| ***H. sapiens*** | xRead-nd | 30 | 12.32 (h) | 19.13 |
|  | xRead-wtpoa | 30 | 15.76 (h) | 25.04 |
|  | NextDenovo | 30 | 25.24 (h) | 273.97 |
|  | Wtdbg2 | 30 | 48.10 (h) | 330.46 |
|  | Flye | 30 | 27.18 (h) | 341.89 |
|  | Shasta | 30 | 3.54 (h) | 692.78 |
| **Simulated PacBio HiFi datasets with an average accuracy > 99.5%** | | | | |
| ***E. coli*** | xRead-nd | 30 | 6.83 | 0.69 |
|  | xRead-wtpoa | 30 | 17.95 | 1.91 |
|  | NextDenovo | 30 | 37.34 | 1.43 |
|  | Wtdbg2 | 30 | 23.669 | 1.79 |
|  | Flye | 30 | 296.90 | 1.05 |
|  | Shasta | 30 | 256.32 | 1.62 |
|  | Hifiasm | 30 | 4.72 | 0.12 |
| ***A. thaliana*** | xRead-nd | 30 | 142.73 | 6.89 |
|  | xRead-wtpoa | 30 | 398.73 | 12.74 |
|  | NextDenovo | 30 | 794.00 | 5.24 |
|  | Wtdbg2 | 30 | 568.97 | 8.16 |
|  | Flye | 30 | 5007.74 | 25.91 |
|  | Shasta | 30 | 916.23 | 46.85 |
|  | Hifiasm | 30 | - | - |
| ***D. melanogaster*** | xRead-nd | 30 | 184.73 | 7.58 |
|  | xRead-wtpoa | 30 | 477.13 | 21.19 |
|  | NextDenovo | 30 | 1981.81 | 6.07 |
|  | Wtdbg2 | 30 | 716.90 | 10.12 |
|  | Flye | 30 | 12331.66 | 29.09 |
|  | Shasta | 30 | 1884.69 | 49.04 |
|  | Hifiasm | 30 | 4439.20 | 17.74 |
| ***H. sapiens*** | xRead-nd | 30 | 12.70 (h) | 17.35 |
|  | xRead-wtpoa | 30 | 14.95 (h) | 37.27 |
|  | NextDenovo | 30 | 36.63 (h) | 101.06 |
|  | Wtdbg2 | 30 | 19.70 (h) | 189.90 |
|  | Flye | 30 | 46.90 (h) | 249.01 |
|  | Shasta | 30 | 7.13 (h) | 776.61 |
|  | Hifiasm | 30 | 16.41 (h) | 169.99 |

(a) The assemblies were generated using three pipelines. NextDenovo: default pipeline of NextDenovo version 2.5.1. xRead-nd: xRead version 1.0.0, itergrated with the nextgraph of NextDenovo version 2.5.1. xRead-wtpoa: xRead-nd, integrated with the wtpoa-cns of Wtdbg2 version 2.5. Wtdbg2: version 2.5. Flye: version 2.9.4. Shasta: version Linux-0.12.0. Hifiasm: version 0.19.9.

(b) The number of CPU threads used for benchmarking on simulated datasets.

(c) The overall real-time of the tools cost on simulated datasets, the results marked by “h” indicates CPU hours, otherwise, CPU seconds.

(d) The peak memory of the tools (in GB) on simulated datasets.

# Supplementary Table 12. Statistics of assembly results on simulated datasets

| **Datasets** | **Tool ^a^** | | **Assembly**  **Length (bp) ^b^** | | **#Contigs ^c^** | | **N10**  **(kb) ^d^** | | **N50**  **(kb) ^d^** | | **N90**  **(kb) ^d^** | | **Genome fraction (%) ^e^** | | **#Misassemblies ^f^** | | **#SEs ^g^** |
| --- | --- | --- | --- | --- | --- | --- | --- | --- | --- | --- | --- | --- | --- | --- | --- | --- | --- |
| **Simulated ONT datasets with an average accuracy of 87%** | | | | | | | | | | | | | | | | | |
| ***E. coli*** | xRead-nd | 4573814 | | 1 | | 4573.81 | | 4573.81 | | 4573.81 | | 98.537 | | 0 | | 0 | |
|  | xRead-wtpoa | 4638314 | | 1 | | 4638.31 | | 4638.31 | | 4638.31 | | 99.957 | | 0 | | 0 | |
|  | xRead-pipe | 4638314 | | 1 | | 4638.31 | | 4638.31 | | 4638.31 | | 99.957 | | 0 | | 0 | |
|  | NextDenovo | 4635638 | | 1 | | 4635.64 | | 4635.64 | | 4635.64 | | 99.870 | | 0 | | 0 | |
|  | Wtdbg2 | 4638136 | | 1 | | 4638.14 | | 4638.14 | | 4638.14 | | 99.942 | | 0 | | 0 | |
|  | Flye | 4641415 | | 1 | | 4641.42 | | 4641.42 | | 4641.42 | | 99.994 | | 0 | | 0 | |
| ***A. thaliana*** | xRead-nd | 117319092 | | 15 | | 18324.06 | | 14221.29 | | 4297.88 | | 99.033 | | 25 | | 4 | |
|  | xRead-wtpoa | 118820332 | | 15 | | 18550.87 | | 14400.92 | | 4340.45 | | 99.325 | | 13 | | 3 | |
|  | xRead-pipe | 118765481 | | 18 | | 17500.48 | | 12989.19 | | 4340.45 | | 99.272 | | 8 | | 0 | |
|  | NextDenovo | 118672238 | | 12 | | 26849.62 | | 18505.42 | | 14449.55 | | 99.109 | | 13 | | 0 | |
|  | Wtdbg2 | 113377694 | | 100 | | 11561.15 | | 4808.57 | | 713.18 | | 94.825 | | 54 | | 3 | |
|  | Flye | 118894813 | | 44 | | 16207.16 | | 9255.43 | | 2386.07 | | 99.370 | | 14 | | 0 | |
| ***D. melanogaster*** | xRead-nd | 135976802 | | 31 | | 24697.44 | | 14936.88 | | 2637.39 | | 95.531 | | 102 | | 3 | |
|  | xRead-wtpoa | 137472546 | | 31 | | 25001.84 | | 15120.54 | | 2670.25 | | 95.583 | | 40 | | 3 | |
|  | xRead-pipe | 137382673 | | 34 | | 21358.28 | | 13904.87 | | 2670.25 | | 95.546 | | 33 | | 0 | |
|  | NextDenovo | 136208158 | | 24 | | 28823.12 | | 23283.37 | | 21269.54 | | 95.454 | | 37 | | 1 | |
|  | Wtdbg2 | 132079831 | | 133 | | 21466.01 | | 12108.28 | | 1103.49 | | 92.381 | | 131 | | 9 | |
|  | Flye | 136564595 | | 109 | | 18699.27 | | 7356.89 | | 1076.20 | | 95.599 | | 32 | | 0 | |
| ***H. sapiens*** | xRead-nd | 3006132246 | | 1008 | | 52837.94 | | 16587.64 | | 1863.45 | | 94.836 | | 2201 | | 323 | |
|  | xRead-wtpoa | 3043144197 | | 1008 | | 53488.37 | | 16792.31 | | 1886.41 | | 95.479 | | 657 | | 244 | |
|  | xRead-pipe | 3029245379 | | 1286 | | 27763.23 | | 9008.31 | | 1112.31 | | 95.238 | | 312 | | 14 | |
|  | NextDenovo | 2824960124 | | 537 | | 78352.55 | | 32222.91 | | 5171.77 | | 91.227 | | 261 | | 33 | |
|  | Wtdbg2 | 2827893704 | | 5122 | | 62574.62 | | 15586.46 | | 1986.11 | | 92.869 | | 1226 | | 103 | |
|  | Flye | 2835378909 | | 1638 | | 75611.48 | | 24292.89 | | 3249.17 | | 92.604 | | 113 | | 2 | |
| **Simulated ONT datasets with an average accuracy of 94%** | | | | | | | | | | | | | | | | | |
| ***E. coli*** | xRead-nd | 4613021 | | 1 | | 4613.02 | | 4613.02 | | 4613.02 | | 99.382 | | 0 | | 0 | |
|  | xRead-wtpoa | 4637408 | | 1 | | 4637.41 | | 4637.41 | | 4637.41 | | 99.909 | | 0 | | 0 | |
|  | xRead-pipe | 4637408 | | 1 | | 4637.41 | | 4637.41 | | 4637.41 | | 99.909 | | 0 | | 0 | |
|  | NextDenovo | 4638319 | | 1 | | 4638.32 | | 4638.32 | | 4638.32 | | 99.928 | | 0 | | 0 | |
|  | Wtdbg2 | 4639219 | | 1 | | 4639.22 | | 4639.22 | | 4639.22 | | 99.928 | | 0 | | 0 | |
|  | Flye | 4641415 | | 1 | | 4641.42 | | 4641.42 | | 4641.42 | | 99.994 | | 0 | | 0 | |
|  | Shasta | 4635037 | | 1 | | 4635.04 | | 4635.04 | | 4635.04 | | 99.856 | | 0 | | 0 | |
| ***A. thaliana*** | xRead-nd | 118503456 | | 17 | | 23843.61 | | 10894.73 | | 3414.08 | | 99.138 | | 21 | | 6 | |
|  | xRead-wtpoa | 119007665 | | 17 | | 23999.88 | | 10966.31 | | 3436.32 | | 99.539 | | 17 | | 4 | |
|  | xRead-pipe | 118756243 | | 21 | | 16477.07 | | 9540.80 | | 3393.56 | | 99.246 | | 11 | | 0 | |
|  | NextDenovo | 118518803 | | 13 | | 18529.59 | | 14970.75 | | 9272.22 | | 99.069 | | 12 | | 0 | |
|  | Wtdbg2 | 110523086 | | 199 | | 5837.85 | | 1759.35 | | 316.97 | | 92.407 | | 225 | | 5 | |
|  | Flye | 118932644 | | 46 | | 16237.16 | | 8594.20 | | 3962.79 | | 99.547 | | 8 | | 0 | |
|  | Shasta | 118021571 | | 73 | | 10127.22 | | 5526.83 | | 1421.22 | | 99.041 | | 9 | | 0 | |
| ***D. melanogaster*** | xRead-nd | 137109662 | | 42 | | 37568.26 | | 11734.63 | | 1341.79 | | 95.872 | | 83 | | 22 | |
|  | xRead-wtpoa | 138044534 | | 42 | | 37814.01 | | 11814.49 | | 1350.04 | | 96.012 | | 53 | | 16 | |
|  | xRead-pipe | 137213557 | | 57 | | 29498.50 | | 8676.69 | | 1112.43 | | 95.992 | | 32 | | 1 | |
|  | NextDenovo | 136345804 | | 31 | | 32050.17 | | 24405.68 | | 19991.45 | | 95.521 | | 31 | | 1 | |
|  | Wtdbg2 | 132632320 | | 186 | | 21818.61 | | 13860.82 | | 832.27 | | 92.291 | | 168 | | 17 | |
|  | Flye | 138251889 | | 148 | | 28536.76 | | 19750.73 | | 1293.19 | | 96.677 | | 79 | | 4 | |
|  | Shasta | 135039274 | | 170 | | 27899.55 | | 11185.26 | | 609.88 | | 94.946 | | 18 | | 0 | |
| ***H. sapiens*** | xRead-nd | 2999038656 | | 795 | | 60534.21 | | 17937.72 | | 2543.40 | | 95.532 | | 858 | | 189 | |
|  | xRead-wtpoa | 3018821214 | | 795 | | 60445.29 | | 18054.46 | | 2559.75 | | 96.012 | | 463 | | 136 | |
|  | xRead-pipe | 3011702354 | | 937 | | 28964.21 | | 10336.64 | | 1088.76 | | 95.897 | | 187 | | 5 | |
|  | NextDenovo | 2826366146 | | 548 | | 98900.09 | | 33377.41 | | 5272.52 | | 91.539 | | 329 | | 120 | |
|  | Wtdbg2 | 2779752148 | | 4263 | | 35460.62 | | 12943.85 | | 1398.36 | | 91.069 | | 1743 | | 202 | |
|  | Flye | 2880044671 | | 2011 | | 90530.43 | | 23996.78 | | 3140.97 | | 94.486 | | 111 | | 9 | |
|  | Shasta | 2872617389 | | 2047 | | 50155.95 | | 22858.11 | | 3654.82 | | 93.451 | | 31 | | 13 | |
| **Simulated PacBio HiFi datasets with an average accuracy > 99.5%** | | | | | | | | | | | | | | | | | |
| ***E. coli*** | xRead-nd | 4642218 | | 1 | | 4642.22 | | 4642.22 | | 4642.22 | | 99.900 | | 0 | | 0 | |
|  | xRead-wtpoa | 4637086 | | 1 | | 4637.09 | | 4637.09 | | 4637.09 | | 99.902 | | 0 | | 0 | |
|  | xRead-pipe | 4637086 | | 1 | | 4637.09 | | 4637.09 | | 4637.09 | | 99.902 | | 0 | | 0 | |
|  | NextDenovo | 4638774 | | 1 | | 4638.77 | | 4638.77 | | 4638.77 | | 99.938 | | 0 | | 0 | |
|  | Wtdbg2 | 4638735 | | 1 | | 4638.74 | | 4638.74 | | 4638.74 | | 99.937 | | 0 | | 0 | |
|  | Flye | 4637366 | | 1 | | 4637.37 | | 4637.37 | | 4637.37 | | 99.908 | | 0 | | 0 | |
|  | Shasta | 4635644 | | 1 | | 4635.64 | | 4635.64 | | 4635.64 | | 99.871 | | 0 | | 0 | |
|  | Hifiasm | 4640995 | | 1 | | 4641.00 | | 4641.00 | | 4641.00 | | 99.986 | | 0 | | 0 | |
| ***A. thaliana*** | xRead-nd | 119429257 | | 17 | | 18630.77 | | 15412.70 | | 6567.76 | | 99.530 | | 16 | | 9 | |
|  | xRead-wtpoa | 119217868 | | 17 | | 18602.79 | | 15381.17 | | 6560.43 | | 99.509 | | 15 | | 7 | |
|  | xRead-pipe | 118823457 | | 24 | | 16387.41 | | 13133.50 | | 4218.61 | | 99.384 | | 6 | | 0 | |
|  | NextDenovo | 118654105 | | 12 | | 18543.19 | | 16390.61 | | 9271.27 | | 99.247 | | 20 | | 0 | |
|  | Wtdbg2 | 116588264 | | 118 | | 13099.77 | | 5948.05 | | 554.08 | | 97.599 | | 38 | | 1 | |
|  | Flye | 119295921 | | 46 | | 16228.81 | | 9284.11 | | 6102.58 | | 99.512 | | 7 | | 1 | |
|  | Shasta | 118499642 | | 71 | | 8785.49 | | 5684.97 | | 1556.97 | | 99.200 | | 4 | | 0 | |
|  | Hifiasm | - | | - | | - | | - | | - | | - | | - | | - | |
| ***D. melanogaster*** | xRead-nd | 136918774 | | 28 | | 32088.80 | | 24422.49 | | 3309.09 | | 95.587 | | 77 | | 29 | |
|  | xRead-wtpoa | 136707179 | | 28 | | 32041.34 | | 24391.79 | | 3304.19 | | 95.132 | | 62 | | 21 | |
|  | xRead-pipe | 135723952 | | 42 | | 26731.36 | | 21573.39 | | 1505.37 | | 95.023 | | 33 | | 0 | |
|  | NextDenovo | 136497766 | | 30 | | 31999.51 | | 24377.56 | | 22641.20 | | 95.492 | | 36 | | 0 | |
|  | Wtdbg2 | 122229732 | | 159 | | 17929.53 | | 13268.60 | | 610.51 | | 85.900 | | 138 | | 12 | |
|  | Flye | 136972048 | | 107 | | 18503.00 | | 5403.21 | | 945.54 | | 95.726 | | 20 | | 2 | |
|  | Shasta | 135330328 | | 103 | | 27900.35 | | 15662.11 | | 1037.83 | | 94.935 | | 4 | | 0 | |
|  | Hifiasm | 135877135 | | 65 | | 27904.64 | | 21478.66 | | 1156.78 | | 95.269 | | 4 | | 0 | |
| ***H. sapiens*** | xRead-nd | 3034139693 | | 987 | | 56721.34 | | 18110.77 | | 2328.49 | | 96.024 | | 716 | | 126 | |
|  | xRead-wtpoa | 3029456194 | | 987 | | 56626.01 | | 18085.61 | | 2324.83 | | 95.653 | | 547 | | 121 | |
|  | xRead-pipe | 3020806604 | | 1106 | | 33246.74 | | 9672.90 | | 1815.73 | | 95.153 | | 226 | | 11 | |
|  | NextDenovo | 2868299407 | | 810 | | 81515.65 | | 28688.81 | | 4616.58 | | 93.234 | | 191 | | 25 | |
|  | Wtdbg2 | 2769197307 | | 1711 | | 66592.33 | | 19514.81 | | 2675.58 | | 89.800 | | 201 | | 95 | |
|  | Flye | 3043060109 | | 1950 | | 110620.19 | | 40901.96 | | 5086.58 | | 98.548 | | 92 | | 7 | |
|  | Shasta | 3073577436 | | 3308 | | 110670.46 | | 45039.61 | | 4966.01 | | 98.887 | | 5 | | 8 | |
|  | Hifiasm | 2852490207 | | 101 | | 287400.38 | | 141036.2 | | 61706.97 | | 91.689 | | 64 | | 260 | |

(a) The assemblies were generated using eight pipelines and benchmarked using QUAST and an in-house assessment script to assess the misassemblies.

(b) The total number of bases in all contigs.

(c) The total number of contigs.

(d) N10/N50/N90: The length of the shortest contig at 10%/50%/90% of the assembly.

(e) The percentage of aligned bases of the reference genome.

(f) The number of misassemblies defined by QUAST. In short, the number of alignment breakpoints of all contigs.

(g) The number of structural errors.


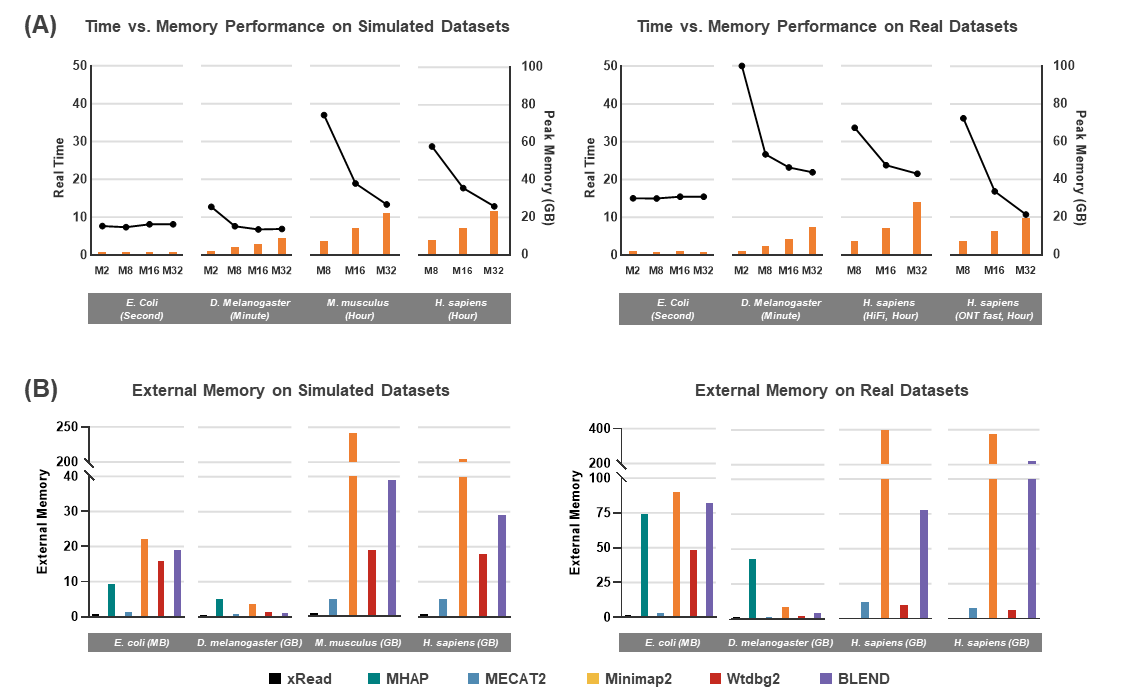


# Supplementary Figure 1. The performance and external memory usage on simulated and real datasets.

(A) The performance of xRead with various RAM configurations across 4 simulated and 4 real datasets. The memory configurations are respectively set to 2GB, 8GB, 16GB and 32GB (labeled as M2, M8, M16 and M32 correspondingly). The runtime and memory footprints are respectively shown by the left and right y-axis. Worthnoting that the unit of real-time varies and is indicated in the gray blocks below the plot as seconds, minutes or hours.

(B) The external memory usage of xRead, MHAP, MECAT2, Minimap2, Wtdbg2 and BLEND on 4 simulated and 4 real datasets. The tools have various output formats, i.e., MHAP employs the M4 format from BLASR, Wtdbg2 uses its own custom zip format (alignment.gz) and xRead, MECAT2, Minimap2 and BLEND use PAF format. All uncompressed output files are compressed by gzip for consistency. The unit of external memory usage varies and is marked in the gray blocks below the plot as MB or GB. Note that assemblers like Wtdbg2 generate supplementary layout information for the subsequent assembly step, which is excluded from the comparison.


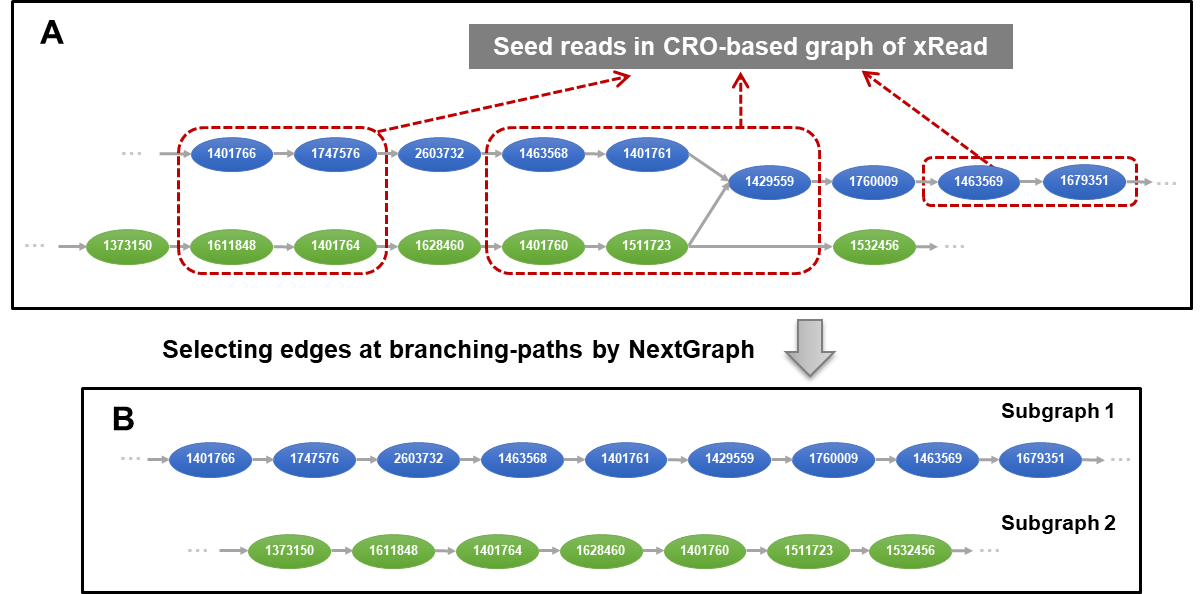


# Supplementary Figure 2. An example of the mistaken selection of edges at branching paths by NextGraph.

(A) A local representation of the graph produced by xRead-nd. The seed reads are outlined in red dashed boxes. The nodes comprising the final assembly path are marked in blue and green corresponding to distinct contigs.

(B) After selecting and removing edges at branching paths by NextGraph, two new subgraphs were produced. The critical edge between node 1511723 and 1429559 was mistakenly removed, leading to a gap and a mis-assembly.


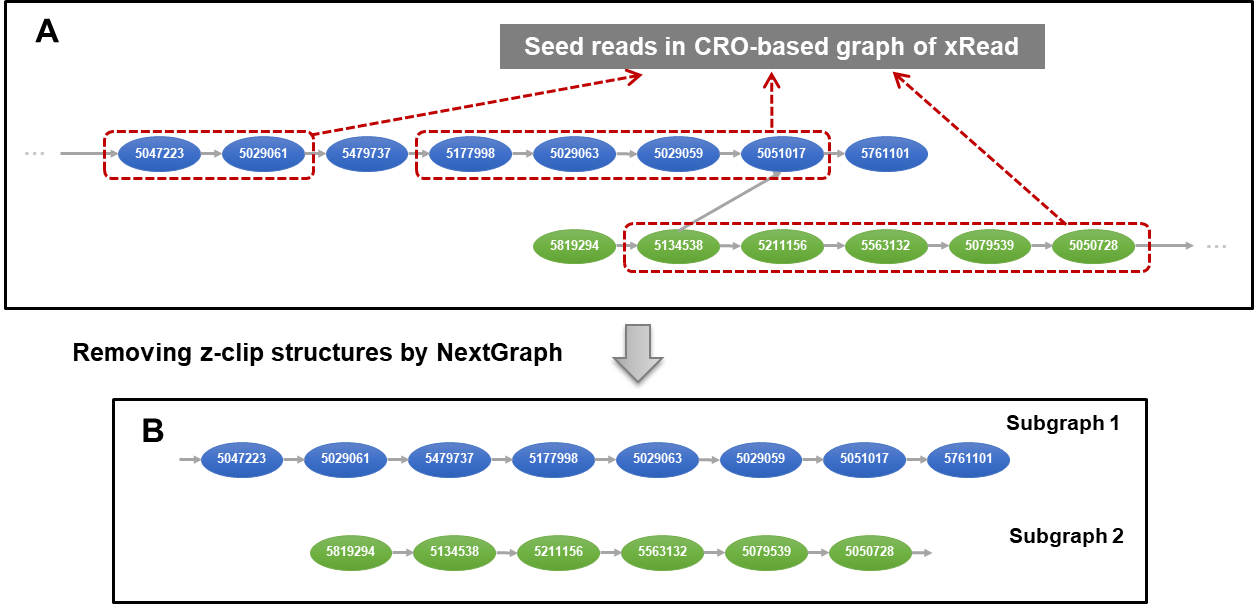


# Supplementary Figure 3. An example of the removal of critical edges of z-clip structure by NextGraph.

(A) A local representation of the graph produced by xRead-nd. The seed reads are outlined in red dashed boxes. The nodes comprising the final assembly path are marked in blue and green corresponding to distinct contigs.

(B) After removing z-clip structures by NextGraph, two new subgraphs were produced. The critical edge in the graph between node 5134538 and 5051007 was removed, leading to the introduction of a gap in the assembly results.


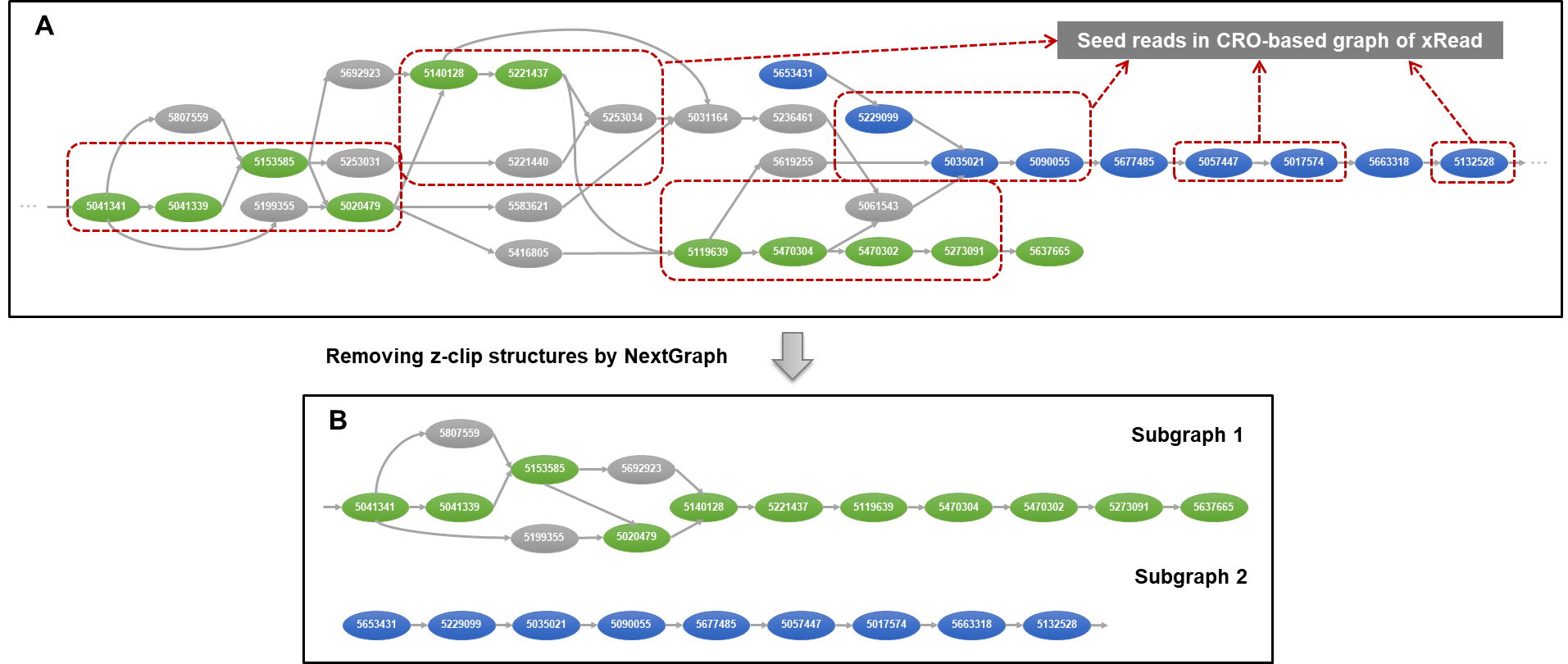


# Supplementary Figure 4. An example of the removal of critical edges of z-clip structure by NextGraph.

(A) A local representation of the graph produced by xRead-nd. The seed reads are outlined in red dashed boxes. The nodes comprising the final assembly path are marked in blue and green corresponding to distinct contigs, other related nodes are marked in gray.

(B) After removing z-clip structures by NextGraph, two new subgraphs were produced. The critical edges between the blue node (5035021) and green nodes (5119639 and 5470304) connected by gray nodes (5619255 and 5061543) were removed, leading to the introduction of a gap in the assembly results.


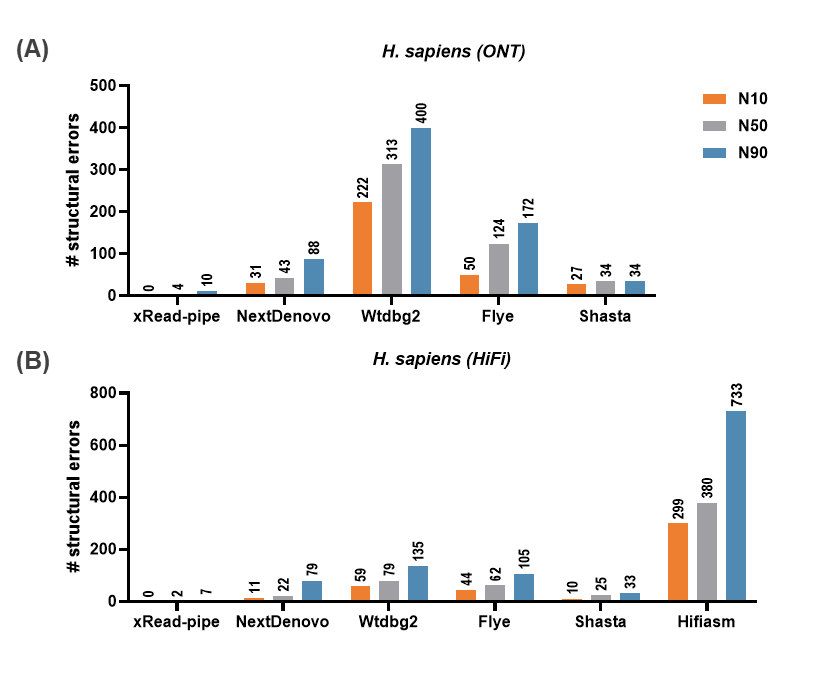


# Supplementary Figure 5. The number of mis-assemblies in contigs longer than N10/N50/N90 values of various assemblers.

(A) The number of structural errors in the assemblies from xRead-pipe, NextDenovo, Wtdbg2, Flye and Shasta on a real human ONT dataset. The number of structural errors is shown for contigs with lengths greater than the N10, N50, and N90 thresholds, represented by orange, gray, and blue bars, respectively.

(B) The number of structural errors in the assemblies from xRead-pipe, NextDenovo, Wtdbg2, Flye, Shasta and Hifiasm on a real human HiFi dataset. The number of structural errors is shown for contigs with lengths greater than the N10, N50, and N90 thresholds, represented by orange, gray, and blue bars, respectively.


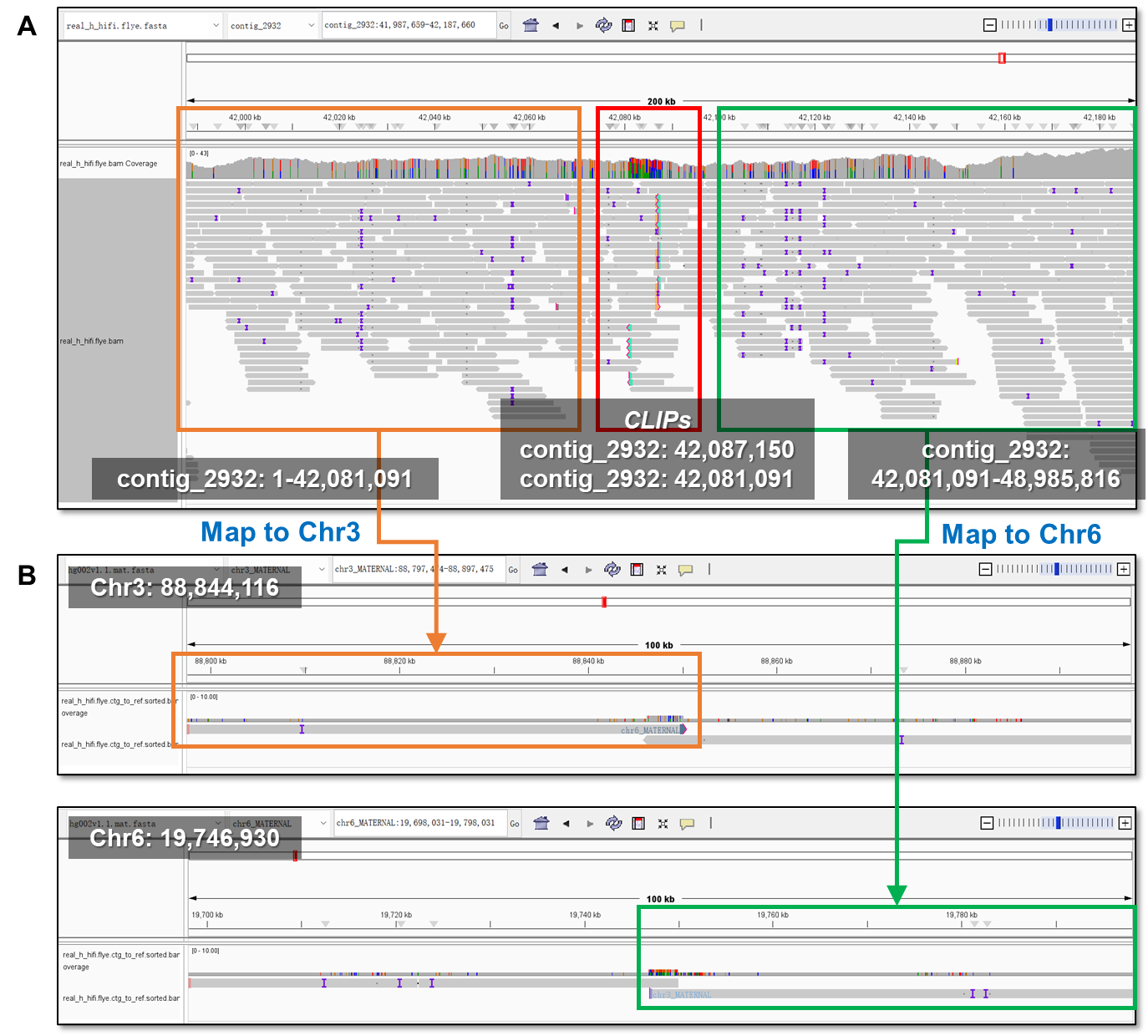


# Supplementary Figure 6. An example of the structural error in a contig of Flye on the real human PacBio HiFi dataset.

(A) The IGV snapshot of HiFi read alignment against the contig contig_2932 produced by Flye. Two CLIPs were observed (red box) on contig_2932: 42,087,150 and contig_2932: 42,081,091, respectively.

(B) The IGV snapshot of aligning contigs produced by Flye against the reference chromosomes Chr3 and Chr6, respectively. Split alignments were observed: the region to the left of CLIP (contig_2932: 1-42,081,091, orange box) maps to Chr3: 88,844,116, while the region to the right of CLIP (contig_2932: 42,081,092-48,985,816, green box) maps to Chr6: 19,746,930.


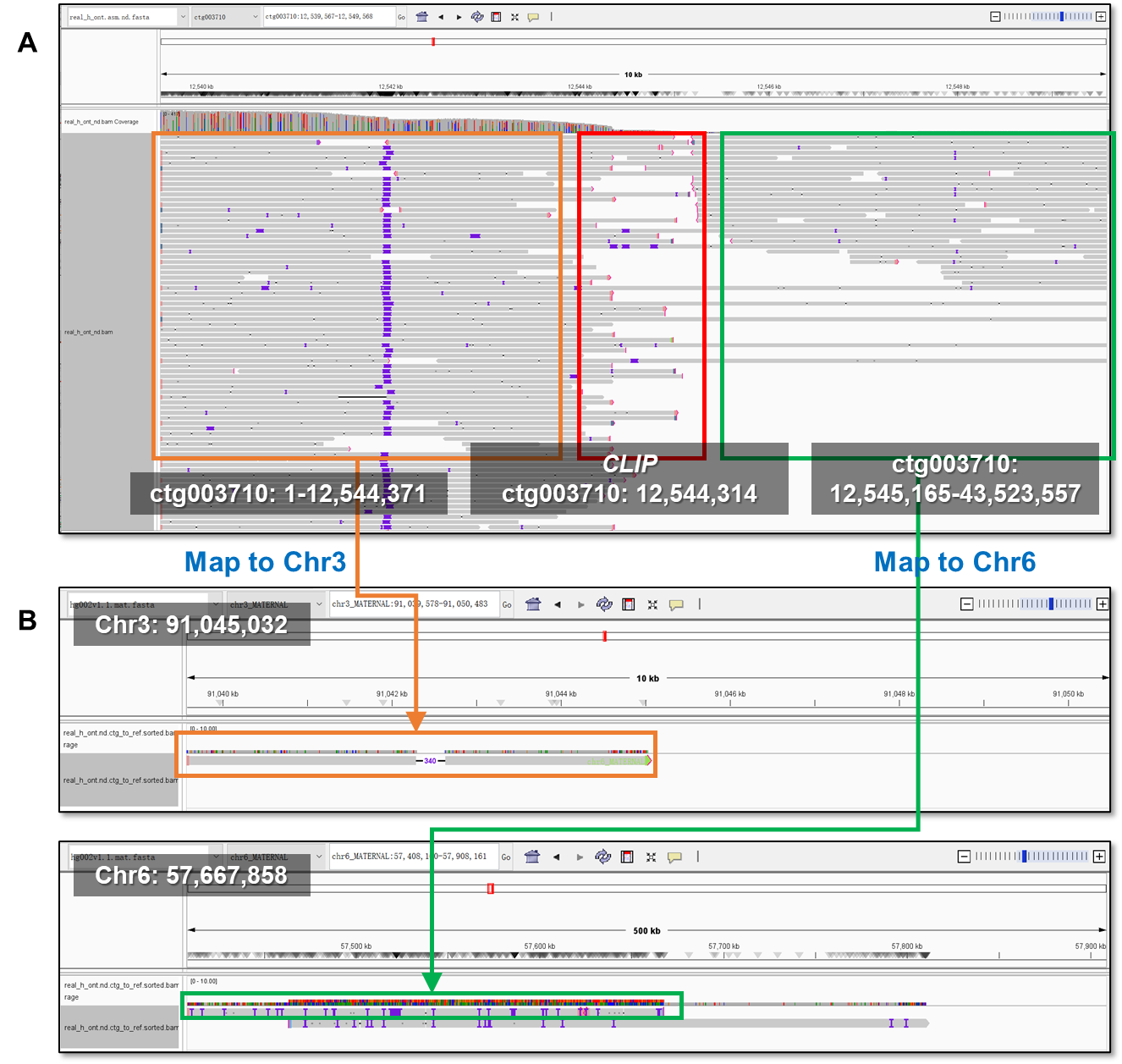


# Supplementary Figure 7. An example of the structural error in a contig of NextDenovo on the real human ONT dataset.

(A) The IGV snapshot of ONT read alignment against the contig ctg003710 produced by NextDenovo. A CLIP was observed (red box) on ctg003710: 12,544,314.

(B) The IGV snapshot of aligning contigs produced by NextDenovo against the reference chromosomes Chr3 and Chr6. Split alignments caused by this structural error were observed. The segment to the left of CLIP (ctg003710: 1-12,544,371, orange box) was mapped to the left of Chr3: 91,045,032, while the segment to the right of CLIP (ctg003710: 12,545,165-43,523,557, green box) was mapped to the left of Chr6: 57,667,858.


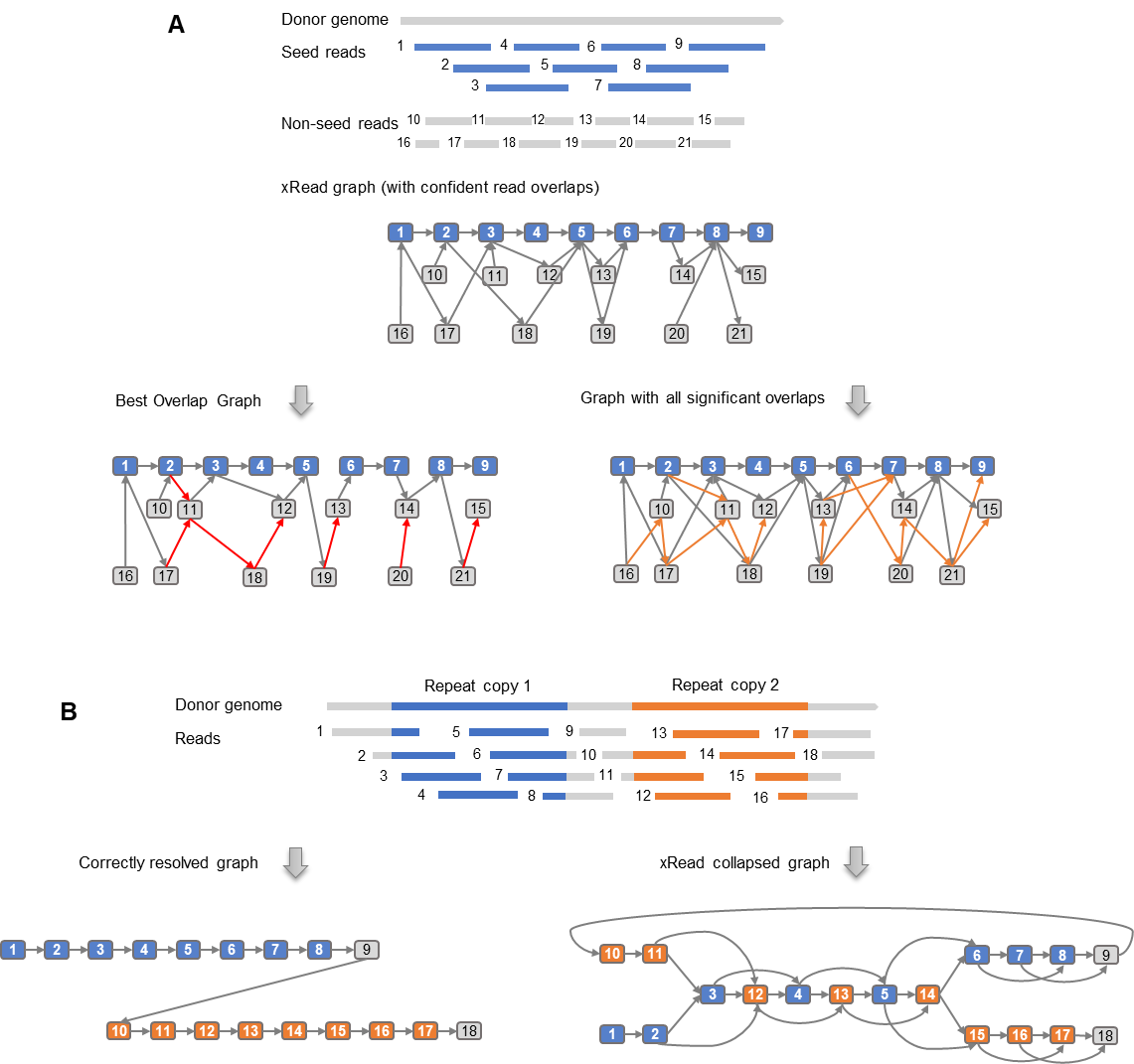


# Supplementary Figure 8. The schematic illustrations for the features of xRead approach.

(A) Two different graph styles converted from xRead’s overlapping graph. (Top) Given a specific genomic region, xRead implements coverage-guided overlapping among seed reads (marked as blue bars) and non-seed reads (marked as gray bars) which produces a connected component for the seed- and non-seed-reads. (Bottom left) xRead converts the produced graph to the best overlap graph based on transitive relationships implied by the connected component. The edges (represented by red lines) inferred by transitive operations correspond to the best overlap for the read end. Edges representing non-best overlaps are eliminated. (Bottom right) xRead converts the produced graph to a comprehensive overlapping graph containing all significant overlaps based on transitive relationships implied by the connected component. The edges (represented by orange lines) are inferred by transitive operations.

(B) The repeat handling of xRead. (Top) Two highly similar repeat copies (marked in blue and orange) that no read is long enough to span. (Bottom left) Ideally, the reads from the two repeat copies are distinguishable, forming a correctly resolved graph as illustrated. (Bottom right) The overlapping graph produced by xRead. The reads from repeat copy 1 and copy 2 are connected to form a single component, i.e., the graph collapses for the various copies, due to the crosstalk between their high similarity and sequencing errors. Such a case also usually happens to other read-overlapping tools, which need more advanced methods to resolve in downstream assembly steps, such as read correction or heuristic layout.

# Supplementary Note 1. The command and parameters used for assembly benchmarks

**1. The commands and parameters used for assembly pipelines on simulated datasets**

**(1) For simulated ONT datasets with an average accuracy of 87%:**

**xRead**

$xRead -k 15 -l 11 -w 5 -x 5 -X 10 -a 2000 -n 3 -m 500 -b 100 -t 30 -M 24 {sim_dataset} -f {out_file}

**NextDenovo**

[General]

job_type = local

job_prefix = nextDenovo

task = all # 'all', 'correct', 'assemble'

rewrite = yes # yes/no

deltmp = yes

rerun = 3

parallel_jobs = 2

input_type = raw

read_type = ont

input_fofn = {input_file_dir}

workdir = {out_dir}

[correct_option]

read_cutoff = 1k

genome_size = {genome_size}

pa_correction = 2

sort_options = -m 170g -t 30

minimap2_options_raw = -t 30

correction_options = -p 15

[assemble_option]

minimap2_options_cns = -t 30

nextgraph_options = -a 1

**Note:** The {genome_size} option was assigned as 4.6m, 120m, 140m and 3.1g for *E. coli, A. thaliana, D. melanogaster* and *H. sapiens*, respectively.

**Wtdbg2**

For nanopore data and genome size < 1G:

$wtdbg2 -x preset2 -g {genome_size} -i {sim_dataset} -t 30 -fo {out_file1}

$wtpoa_cns -t 30 -i{out_file1}.ctg.lay.gz -fo {out_file2}

For nanopore data and genome size >= 1G:

$wtdbg2 -x preset3 -g {genome_size} -i {sim_dataset} -t 30 -fo {out_file1}

$wtpoa_cns -t 30 -i{out_file1}.ctg.lay.gz -fo {out_file2}

**Flye**

$flye --nano-raw {sim_dataset} --out-dir {out_dir} --threads 30

**Shasta**

$shasta --input {sim_dataset} --config Nanopore-OldGuppy-Seq2020 --assemblyDirectory {out_dir} --threads 30

**(2) For simulated ONT datasets with an average accuracy of 94%:**

**xRead**

$xRead -k 15 -l 11 -w 13 -x 5 -X 10 -a 1500 -n 3 -m 800 -b 300 -t 30 -M 24 {sim_dataset} -f {out_file}

**NextDenovo**

[General]

read_type = ont

**Wtdbg2**

For nanopore data and genome size < 1G:

$wtdbg2 -x preset2 -g {genome_size} -i {sim_dataset} -t 30 -fo {out_file1}

$wtpoa_cns -t 30 -i{out_file1}.ctg.lay.gz -fo {out_file2}

For nanopore data and genome size >= 1G:

$wtdbg2 -x preset3 -g {genome_size} -i {sim_dataset} -t 30 -fo {out_file1}

$wtpoa_cns -t 30 -i{out_file1}.ctg.lay.gz -fo {out_file2}

**Flye**

$flye --nano-hq {sim_dataset} --out-dir {out_dir} --threads 30

**Shasta**

$shasta --input {sim_dataset} --config Nanopore-May2022 --assemblyDirectory {out_dir} --threads 30

**(3) For simulated PacBio HiFi datasets with average accuracy > 99.5%:**

**xRead**

$xRead -k 15 -l 11 -w 17 -x 5 -X 10 -a 1000 -n 3 -m 1000 -b 600 -t 30 -M 24 {sim_dataset} -f {out_file}

**NextDenovo**

[General]

read_type = hifi

**Wtdbg2**

$wtdbg2 -x preset4 -g {genome_size} -i {sim_dataset} -t 30 -fo {out_file1}

$wtpoa_cns -t 30 -i{out_file1}.ctg.lay.gz -fo {out_file2}

**Flye**

$flye --pacbio-hifi {sim_dataset} --out-dir {out_dir} --threads 30

**Shasta**

$shasta --input {sim_dataset} --config HiFi-Oct2021 --assemblyDirectory {out_dir} --threads 30

**2. The commands and parameters used for assembly pipelines on real datasets**

**(1) For ONT datasets in fast base-calling mode:**

**xRead**

$xRead -k 15 -l 11 -w 5 -x 5 -X 10 -a 2000 -n 3 -m 500 -b 100 -t 30 -M 24 {real_dataset} -f {out_file}

**NextDenovo**

[General]

read_type = ont

**Wtdbg2**

For nanopore data and genome size < 1G:

$wtdbg2 -x preset2 -g {genome_size} -i {real_dataset} -t 30 -fo {out_file1}

$wtpoa_cns -t 30 -i{out_file1}.ctg.lay.gz -fo {out_file2}

For nanopore data and genome size >= 1G:

$wtdbg2 -x preset3 -g {genome_size} -i {real_dataset} -t 30 -fo {out_file1}

$wtpoa_cns -t 30 -i{out_file1}.ctg.lay.gz -fo {out_file2}

**Flye**

$flye --nano-raw {real_dataset} --out-dir {out_dir} --threads 30

**Shasta**

$shasta --input {real_dataset} --config Nanopore-OldGuppy-Seq2020 --assemblyDirectory {out_dir} --threads 30

**(2) For human PacBio HiFi datasets:**

**xRead**

$xRead -k 15 -l 11 -w 17 -x 5 -X 10 -a 1000 -n 3 -m 1000 -b 600 -t 30 -M 24 {real_dataset} -f {out_file}

**NextDenovo**

[General]

read_type = hifi

**Wtdbg2**

$wtdbg2 -x preset4 -g {genome_size} -i {real_dataset} -t 30 -fo {out_file1}

$wtpoa_cns -t 30 -i{out_file1}.ctg.lay.gz -fo {out_file2}

**Flye**

$flye --pacbio-corr {real_dataset} --out-dir {out_dir} --threads 30

**Shasta**

$shasta --input {real_dataset} --config HiFi-Oct2021 --assemblyDirectory {out_dir} --threads 30
