# Supplementary material for: xRead: a coverage-guided approach for scalable construction of read overlapping graph
Source: Gigascience. 2025 Feb 17;14:giaf007. doi: 10.1093/gigascience/giaf007 (PMC11831799; doi:10.1093/gigascience/giaf007)
Supplement: giaf007_GIGA-D-24-00195_Revision_2 [file giaf007_giga-d-24-00195_revision_2.pdf]

GigaScience

xRead: a coverage-guided approach for scalable construction of read overlapping graph

--Manuscript Draft--

**Manuscript Number:**	GIGA-D-24-00195R2
**Full Title:**	xRead: a coverage-guided approach for scalable construction of read overlapping graph
**Article Type:**	Technical Note
**Funding Information:**	National Key Research and Development Program of China (2021YFF1200105) — Prof. Bo Liu<br>National Natural Science Foundation of China (62172125) — Prof. Bo Liu
**Abstract:**	Background: The development of long-read sequencing is promising for the high-quality and comprehensive de novo assembly for various species around the world. However, it is still challenging for assemblers to handle thousands of genomes, tens of gigabase-level assembly sizes and terabase-level datasets efficiently, which is a bottleneck to large-scale de novo sequencing studies. A major cause is the read overlapping graph construction that state-of-the-art tools usually have to cost terabyte-level RAM space and tens of days for large genomes. Such lower performance and scalability are not suited to handle the numerous samples being sequenced.<br><br>Findings: Herein, we propose xRead, a novel iterative overlapping graph construction approach that achieves high performance, scalability and yield simultaneously. Under the guidance of its coverage-based model, xRead converts read-overlapping to heuristic read-mapping and incremental graph construction tasks with highly controllable RAM space and faster speed. It enables to process very large datasets (such as the 1.28 Tb A. mexicanum dataset) with less than 64GB RAM and obviously lower time costs. Moreover, benchmarks suggest that it can produce highly accurate and well-connected overlapping graphs, which are also supportive to various kinds of downstream assembly strategies.<br><br>Conclusions: xRead is able to break through the major bottleneck to graph construction and lays a new foundation for de novo assembly. This tool is suited to handle a large number of datasets from large genomes and may play important roles in many de novo sequencing studies.
**Corresponding Author:**	Bo Liu<br>Harbin Institute of Technology<br>Harbin, Heilongjiang CHINA
**Corresponding Author Secondary Information:**	
**Corresponding Author's Institution:**	Harbin Institute of Technology
**Corresponding Author's Secondary Institution:**	
**First Author:**	Tangchao Kong
**First Author Secondary Information:**	
**Order of Authors:**	Tangchao Kong<br>Yadong Wang<br>Bo Liu
**Order of Authors Secondary Information:**	
**Response to Reviewers:**	Dear Hans,<br>Thank you for your email and for accepting our manuscript “xRead: a coverage-guided

	approach for scalable construction of read overlapping graph" (GIGA-D-24-00195R1) for publication in GigaScience.<br>We have addressed all the editorial points as requested:<br>1. The Software Heritage link has been added to the references and cited in the Data Availability section as instructed.<br>2. All other URLs have been incorporated into the references and appropriately cited in the Data Availability section.<br>3. ORCIDs for all authors have been added to the title page, following their names.<br>4. We have checked the grammar throughout the manuscript, with particular attention to the abstract.<br>5. We have reviewed the journal's requirements for each section of the manuscript to ensure that every section complies accordingly.<br>The revised manuscript has been uploaded to the Editorial Manager system for your review. Thank you for your guidance throughout the process. We look forward to the next steps in the publication of our work.<br>Best wishes,<br>Bo Liu
Additional Information:	
Question	Response
Are you submitting this manuscript to a special series or article collection?	No
**Experimental design and statistics**<br><br>Full details of the experimental design and statistical methods used should be given in the Methods section, as detailed in our Minimum Standards Reporting Checklist. Information essential to interpreting the data presented should be made available in the figure legends.<br><br>Have you included all the information requested in your manuscript?	Yes
**Resources**<br><br>A description of all resources used, including antibodies, cell lines, animals and software tools, with enough information to allow them to be uniquely identified, should be included in the Methods section. Authors are strongly encouraged to cite Research Resource Identifiers (RRIDs) for antibodies, model organisms and tools, where possible.<br><br>Have you included the information requested as detailed in our Minimum Standards Reporting Checklist?	Yes

**Availability of data and materials**<br><br>All datasets and code on which the conclusions of the paper rely must be either included in your submission or deposited in publicly available repositories (where available and ethically appropriate), referencing such data using a unique identifier in the references and in the "Availability of Data and Materials" section of your manuscript.<br><br>Have you have met the above requirement as detailed in our Minimum Standards Reporting Checklist?	Yes

Manuscript

Click here to access/download;Manuscript;xRead Manuscript.docx

xRead: a coverage-guided approach for scalable construction of read overlapping graph

Tangchao Kong [0009-0007-5948-5670][1, 2], Yadong Wang [0000-0001-6500-6217][1, 2, *], Bo Liu [0000-0002-4414-4001][1, 2, *]

[1]Center for Bioinformatics, Faculty of Computing, Harbin Institute of Technology, Harbin, Heilongjiang 150001, China

[2] Key Laboratory of Biological Bigdata, Ministry of Education, Harbin Institute of Technology, Harbin, Heilongjiang 150001, China

* Corresponding author. Yadong Wang, E-mail: ydwang@hit.edu.cn; Bo Liu, E-mail: bo.liu@hit.edu.cn

Abstract

Background: The development of long-read sequencing is promising for the high-quality and comprehensive de novo assembly for various species around the world. However, it is still challenging for assemblers to handle thousands of genomes, tens of gigabase-level assembly sizes and terabase-level datasets efficiently, which is a bottleneck to large-scale de novo sequencing studies. A major cause is the read overlapping graph construction that state-of-the-art tools usually have to cost terabyte-level RAM space and tens of days for large genomes. Such lower performance and scalability are not suited to handle the numerous samples being sequenced.

Findings: Herein, we propose xRead, a novel iterative overlapping graph construction approach that achieves high performance, scalability and yield simultaneously. Under the guidance of its coverage-based model, xRead converts read-overlapping to heuristic read-mapping and incremental graph construction tasks with highly controllable RAM space and faster speed. It enables to process very large datasets (such as the 1.28 Tb *A. mexicanum* dataset) with less than 64GB RAM and obviously lower time costs. Moreover, benchmarks suggest that it can produce highly accurate and well-connected overlapping graphs, which are also supportive to various kinds of downstream assembly strategies.

Conclusions: xRead is able to break through the major bottleneck to graph construction and lays a new foundation for de novo assembly. This tool is suited to handle a large number of datasets from large genomes and may play important roles in many de novo sequencing studies.

Keywords: read overlapping graph, de novo assembly, long-read sequencing, long-read alignment

Introduction

De novo assembly is to reconstruct donor genome sequence from reads without reference, which is fundamental to genomics studies. The rapid advances in long-read sequencing technologies, such as Single Molecule Real Time (SMRT) sequencing [1] and nanopore sequencing [2], have been able to produce reads having >10kbp median length and >100kbp maximum length [3]. They have superior repeat-spanning ability to resolve complex repetitive regions, which greatly helps to achieve high-quality assemblies such as telomere-to-telomere [4] and haplotype assembly [5]. However, the assembly of large genomes is still non-trivial (such as *P. taeda* [6], *A. mexicanum* [7], *E. superba* [8], etc.). One of the bottlenecks is the computation intensity, i.e., most of the state-of-the-art assemblers require terabytes of RAM space and cost thousands of CPU hours for such tasks [9, 10]. In this situation, employed tools are not scalable enough to handle many large genomes with commonly used computational environments since the costs of time and internal memory are prohibitive. Thus, it becomes a major bottleneck to large-scale de novo sequencing studies like the Vertebrate Genomes Project [11] and Earth Biogenome Project [12].

A primary cause of the bottleneck is the all-against-all read alignments to construct initial read overlapping graph which is a fundamental step in Overlap-Layout-Consensus (OLC) approach. OLC is one of the most commonly used approaches adopted by state-of-the-art long read-based assemblers [13-18] (and de Bruijn graph-based approaches as well [19-21]). However, in theory, the upper limit of time complexity of all-against-all read alignment can be very high, i.e., $O(m^2n^2)$, where m and n are the number and length of the reads, respectively. Many efforts have been made to effectively lower this complexity (see below), however, the time cost is still non-neglectable in absolute terms and thus still needs further improvements. Meanwhile, the RAM usage is also high, especially due to all the read information being kept in memory. This is usually a bottleneck for large-scale studies due to the lack of computers with high RAM configuration. Moreover, the crosstalk of genome repeats and sequencing noise also affect the quality of the graph.

State-of-the-art tools use various heuristics to reduce the use of computational resources while improving the yield of read overlapping. Seed-and-extension is one of the most commonly used heuristics. Such approaches retrieve short matches (i.e., seeds) between various reads (usually through indexing data structures) and conduct extended alignments around them to confirm the actual overlapped parts of the reads. The time cost can be significantly reduced with them, as the alignments focus on some pairs of reads being matched whose number is much lower than $O(m^2)$. However, the real cost depends on the various adopted strategies as well. HGAP [22] is one of the earliest tools tailored to the assembly of noisy long reads. It heuristically indexes a proportion of the longest read and employs a typical seed-and-extension alignment tool (BLASR [23]) to align them with other reads.

FALCON assembler [14] employs DALIGNER [24] which partitions reads into blocks and uses sorted k-mers within them as the index, further, the blocks are merged to discover read overlaps. Wtdbg2 [17] indexes a quarter of k-mers as seeds and takes each tiling 256 bp subsequence as a bin for each read. Further, it employs 256 bp bin-based dynamic programming for extension instead of base-level alignment. Flye [25] collects frequent k-mers in reads as seeds and estimates the overlaps by finding the longest common sub-path with a fast dynamic programming algorithm. Shasta [18] randomly selects k-mers (seeds) to find candidate overlaps with the LowHash algorithm and performs a tailored marker alignment approach for extension. Minimap2 [26] is a minimizer-based generic aligner suited to find the overlaps of long reads in various lengths and error rates which is also employed by several state-of-the-art assemblers such as Raven [27], PECAT [28] and Nextdenovo [29]. It essentially uses minimizer-based [30, 31] seeding and chaining to detect read overlaps and also supports the base-level alignment of anchored reads if necessary. Similar to Minimap2, Hifiasm [32] uses a minimizer-based approach tailored for HiFi reads to implement read overlap alignment as the initial step and further achieve haplotype-resolved assembly. BLEND [33] uses SimHash [34, 35] to generate the same hash value for both identical and similar k-mers (seeds) to find fuzzy matches and detect read overlaps.

Some of the previous studies also focus on the acceleration of the base-level alignment to reduce the cost of extension. Most of them take advantage of Single Instruction Multiple Data (SIMD) instructions, such as Intel AVX instructions or Compute Unified Device Architecture (CUDA) in Nvidia GPU. Manavski and Valle proposed an implementation of Smith-Waterman algorithm [36] under CUDA framework. Libssa [37] uses AVX2 instructions to accelerate the classical Smith-Waterman and Needleman-Wunsch algorithms. Parasail [38] is a SIMD-based implementation of global, semi-global and local alignments that supports a couple of instruction sets such as SSE2, SSE4.1, AVX2, AltiVec and NEON. Suzuki and Kasahara developed a fast SIMD-based alignment algorithm named libgaba [39], and it was further improved in KSW2 [26] and employed by Minimap2.

The overall cost of seed-and-extension approaches is still high due to many issues such as the large number of reads, high sequencing errors and ubiquitous repeats. Alignment-free approaches are also proposed. Most of them use compact sequence representations (usually termed sketches [40]) to directly measure read similarities. MHAP [13] uses MinHash technique which employs 256-1512 hash functions to construct sketches and use them to estimate the Jaccard similarity of the reads. Canu [15] uses adaptive k-mer weighting to improve MinHash which reduces the effect of repetitive k-mers. MECAT [16] splits reads into blocks and finds candidate overlaps with at least one matched block. Low-similarity overlaps are then filtered based on distance difference factor (DDF) scores. NECAT [41] extends DDF scoring by sorting all k-mer pairs and chaining them together to remove false positive k-mers. This is more suited to the sequencing errors of ONT reads. Such approaches avoid full alignment, however, their computational cost is also non-neglectable since many query and merging operations

are usually needed to make a number of sketches to achieve high sensitivity.

More efficient and scalable long-read assembly approaches are in wide demand to deal with the ever-increasing sizes and numbers of de novo sequencing genomes. Moreover, there are still a number of false positives/negatives in overlapping graphs caused by the various tradeoffs on sensitivity, precision and performance. Herein, we propose xRead, an incremental overlapping graph construction approach that enables to achieve high scalability, performance and yields simultaneously. Guided by a novel read-coverage-based objective function, xRead iteratively builds the overlapping graph with heuristic read indexing and lightweight alignment skeletons. The approach has three major contributions to breakthrough the bottleneck to the high-performance genome assembly. Firstly, it has outstanding scalability for memory usage which enables to build the overlapping graphs for the datasets of large genomes with low and controllable RAM space cost. For example, it can build an overlapping graph for the 32X PacBio sequencing dataset (1.9 Terabyte) of the Axolotl genome with 64GB or lower RAM. Secondly, it has high speed for various-sized genomes, e.g., several times faster on average than that of Minimap2 on the long-read datasets from small bacteria to large mammal genomes. Thirdly, it is able to achieve high precision and connectivity in graph construction.

Findings

Overview of the xRead approach

Unlike state-of-the-art overlapping tools that in essence push each of the reads to explore all the other ones with heuristics, xRead adopts a “mimicking-and-mapping” design. Mainly, it is motivated by that each read can be seen as a representative of some part of the donor genome. A selected set of seed-reads covering the whole genome can mimic a “virtual reference” to reveal read-overlaps through a read-to-reference mapping, i.e., the reads from the same region can be implicitly aligned to the corresponding seed-read(s) in the virtual reference. So, all the overlaps between seed and non-seed-reads can be detected and the various parts of the genome can also be connected by such alignments. Guided by a novel coverage-based strategy of seed-read selection (“the completeness of virtual reference”, see below), xRead constructs the overlapping graph in an iterative process (Fig. 1). In each iteration, it implements graph construction and refinement in three major steps as follows (also refer to Methods section for more detailed information).

Step 1: xRead selects a proportion of reads with relatively low coverage and high length as seed-reads and builds a partial read index for them.

Step 2: xRead employs a lightweight alignment skeleton approach to discover new read overlaps between the seed-reads and other less covered reads (also termed as query reads).

Step 3: xRead constructs/refines the overlapping graph based on the produced alignment

skeletons. Further, it (re-)estimates the read coverage and stops the process if most reads have high enough coverage, otherwise, turns to step 1 for a new iteration.

This approach lays a new foundation for genome assembly in two aspects. Firstly, it is apparent that the cost can be substantially reduced by converting all-against-all read alignment to a read-to-reference mapping task. Secondly, the produced graph can also be correctly connected, i.e., achieve high precision and connectivity simultaneously, which is supportive to both of the popular strategies in long read assembly (i.e., correction-then-assembly and assembly-then-correction, also refer to Discussion section for a more detailed explanation). These are done by some tailored implementations for several critical issues as follows.

1) The completeness of virtual reference. To achieve the best performance, it is fundamental to select a set of seed-reads covering the whole genome and having as few as possible redundant reads, but non-trivial. xRead achieves this goal by using a fact of the read mapping. That is, given an arbitrary set of seed-reads, relatively high coverage (i.e., times of being aligned) can be derived for all the reads belonging to the virtual-reference covered regions, which is done by exploring the produced alignments and their transitive relationships. Meanwhile, the reads from uncovered regions have much lower coverage. Thus, the coverage becomes a useful indicator to iteratively select the reads from those still uncovered regions and the whole genome can be implicitly and progressively explored. This assumption could be over-simplified for the reads from ultralong repeats, however, it does not affect the overlapping task much (refer to Discussion section for more details).

2) The mappability of virtual reference. A high mappability of virtual reference (seed-reads) is useful to achieve confident read mapping. Thanks to the randomness and less bias of long-read sequencing [42-44], there is no obvious correlation between the read lengths and positions. Thus, with the arbitrary selection assumption mentioned above, it is feasible to straightforwardly select the longest ones from the remaining reads to maximize the mappability of the virtual reference.

3) The correctness of read overlapping. xRead is more precision-oriented in the precision-sensitivity tradeoff during overlapping detection. The virtual reference helps to reduce false positive overlaps systematically with its high mappability. Moreover, xRead also employs a conservative alignment-scoring approach to construct an accurate overlapping graph. Following this strategy, xRead only keeps the most confident read overlaps (CROs, see Methods) with high alignment scores. Motivated by that successful assembly may come from a correctly connected graph, the high-precision design of xRead is useful, especially with good graph connectivity (see below).

4) The connectivity of the produced graph. Although not implementing a comprehensive overlap directly, xRead also produces highly connected graphs. The key point is the completeness of virtual

reference. From a donor genome point of view, the seed-reads are ubiquitously placed and no large gap exists between two nearby seed-reads. Thus, each non-seed-read can be aligned (i.e., connected) to one or more seed-reads, and then the non-seed-reads aligned to the same seed-read compose a connected component. Further, these components can be connected comprehensively since any pair of nearby seed-reads can be either directly aligned (if they are overlapped) or connected via one or more non-seed-reads having overlaps to both of them.

5) The optimization of performance. xRead optimizes speed and RAM usage by several tailored implementations to achieve outstanding performance and scalability. One is the use of the lightweight alignment skeleton (Step 2) to speedup each iteration, referring to previous studies [45-47]. Another is that, followed by the coverage-guided overlapping strategy, the batch size of the seed-reads is highly tunable so that the whole process can be run with controllable RAM usage.

The datasets used in benchmarks

We simulated 17 datasets by PBSIM [48, 49] and also employed 7 real datasets (Table 1) for the benchmark of read overlapping and genome assembly. The datasets are from nine genomes (Supplementary Table 1) having small- (<1Gbp, i.e., *E. coli*, *S. cerevisiae*, *C. elegans*, *A. thaliana* and *D. melanogaster)*, large- (>1Gbp, i.e., *Z. mays*, *M. musculus* and *H. sapiens)* and very large- (>10Gbp, i.e., *A. mexicanum*) sizes. It is also worth noting that we partitioned the chromosomes of the *A. mexicanum* genome in the simulation due to the limit of PBSIM (15 of the chromosomes were divided into 30 <1Gbp ones in advance).

To assess the performance of xRead in various error rates, we respectively simulated nine low-quality, four high-quality ONT-like and four HiFi-like datasets (50x coverage each) as follows.

1) The nine low-quality ONT datasets were simulated for all nine genomes with the pre-trained R103 chemistry model of PBSIM. The mean read length and total error rate were 13 kbp and 13%, respectively, referring to a previous study [3]. These datasets are highly noisy like the fast base-calling mode of ONT platforms and employed to assess the robustness of xRead to sequencing errors for various-sized genomes.

2) The four high-quality ONT datasets were also simulated from four genomes (*E. coli*, *A. thaliana*, *D. melanogaster* and *H. sapiens*). The QSHMM-ONT-HQ model was used (the mean read length and average accuracy were 15 kbp and 94%, respectively) to mimic the data produced by mostly used ONT platforms (especially for its HAC mode).

3) The four HiFi-like datasets were produced by sample-based simulation of PBSIM with a real sequencing dataset (mean read length: 16.6 kbp and average base accuracy: 99.6%) from four genomes (*E. coli*, *A. thaliana*, *D. melanogaster* and *H. sapiens*). These datasets mimic the data

produced by currently used PacBio platforms.

Moreover, the 7 real datasets are as follows. Three of them are from smaller genomes (*E. coli*, *C. elegans* and *D. melanogaster)* and produced by ONT platforms. Three are real human datasets from the well-studied GIAB sample HG002 (NA24385), two of them are ONT datasets in fast and super high accuracy base-calling modes, respectively, and the other one is a PacBio HiFi dataset. The ONT super high accuracy and PacBio HiFi datasets were employed to assess the ability of xRead on high-quality long-read datasets. Furthermore, a PacBio CLR dataset from *A. mexicanum* genome was also used to assess the ability of the tools on very large genomes.

Simulation benchmark for read-overlapping

We implemented benchmarks on the nine low-quality and four high-quality ONT-like simulated datasets to assess the baseline performance of xRead. Five state-of-the-art tools (MHAP, MECAT2, Minimap2, wtdbg2 and BLEND) were employed for comparison. Their runtimes, memory footprints, precisions and sensitivities are in Fig. 2 (refer to Supplementary Tables 3-4 for numerical information). Mainly, three key-points were observed from the results as follows.

1) xRead has outstanding scalability for various-sized genomes.

xRead allows controllable RAM usage and we limited it to 16GB, 24GB and 64GB for the datasets of small, large and very large genomes, respectively. A larger RAM space is used for *A. mexicanum* to reduce I/O operations although it is still much lower than that of comparing tools. xRead accomplishes all the tasks successfully (Fig. 2a and Supplementary Table 3), suggesting that the coverage-guided design is able to well-handle large datasets with medium-sized workstations. Meanwhile, the speed of xRead is about 1.2-18 times (4 times on average) faster in real-time than that of other tools for the low-quality datasets and about 2-29 times (8.5 times on average) for the high-quality datasets, respectively. Overall, the performance suggests that xRead has good scalability to various-sized genomes.

Other tools were run without any limitation on RAM space and their memory footprints are 3-47 times higher than that of xRead with relatively lower speed on all simulated datasets. This could be caused by the following two kinds of read overlapping strategies. One is to load all the reads and process them in memory, like MHAP and wtdbg2, whose RAM usage is quite high. The other one is to divide the whole datasets into batches of reads and separately handle each of them, like MECAT2, Minimap2 and BLEND. For each batch, the involved reads are indexed in time and other reads are aligned to them for overlaps. This is more similar to xRead, however, they also required significantly higher RAM space, e.g., MECAT2 used over 172GB RAM to process the *A. mexicanum* dataset with 64 CPU threads. Moreover, the lower speed of MECAT2, Minimap2 and BLEND could be also due to their own designs, i.e., they straightforwardly divide the datasets and align all the reads to a specific

batch of reads in each iteration. It is also worth noting that only xRead and MECAT2 finished the *A. mexicanum* task, and the real-time of xRead is 60% off compared to that of MECAT2 which saved about 25 days. For this dataset, MHAP and wtdbg2 ran out of memory (over 1 Terabyte). Minimap2 and BLEND showed even lower speeds, i.e., Minimap2 cost about 178 hours (with 64 CPU threads) to process only 11 read batches (about 3.29% of the dataset) and BLEND cost about 175 hours to process 28 read batches (about 8.38% of the dataset). Considering the high time cost (estimated over 225 and 87 days, respectively), we early stopped the programs.

To further investigate the effect of RAM usage on the speed of xRead, we evaluated four other configurations (2 GB, 8 GB, 16 GB and 32 GB) using four low-quality simulated datasets and four real datasets (*E. coli*, *D. melanogaster*, *M. musculus* and *H. sapiens*, Supplementary Figure 1A). The results showed that xRead still kept relatively high speeds even with very low RAM usage (e.g., 2GB) and the runtime gain lowered with larger RAM space (e.g., 32GB), suggesting that a small RAM space is enough for xRead. Mainly, the time-space tradeoff comes from the number of reads being indexed. That is, xRead has to load fewer reads at one time with less RAM and this leads to not only more iterations but also a higher number of index-query operations in each iteration, due to that more matching-failures happened during the generation of the alignment skeletons.

We also assessed the usage of external memory. xRead showed a 50%-99% off compared to other tools (Supplementary Figure 1B and Supplementary Table 10). It is also worth noting that xRead showed superior scalability with large-scale datasets. For example, xRead only required 9.2 GB of external memory on the 2 TB simulated *A. mexicanum* dataset, while MECAT2 consumed 869 GB. This advantage comes from that xRead only records the confident overlaps with high scores, meanwhile, it also avoids transitive overlaps between non-seed-reads which reduces redundancy.

2) xRead produces accurate read overlaps and has the potential to achieve high sensitivity.

We evaluated the overall precisions and sensitivities of the tools (Fig. 2b and Supplementary Table 4, Precision and Sensitivity columns, also refer to Methods section for more details about assessment, including the definitions of ground truth, true positive and false positive overlaps, as well as the computation of precision and sensitivity). The results of the tools varied, indicating their different strategies and tradeoffs. With the relatively conservative CRO strategy, xRead outputs a set of “core overlaps” which connect seed-reads and all the other reads with high scores, achieving the highest precisions on all the datasets. MHAP, MECAT2 and wtdbg2 are more likely to pursue the balance between precision and sensitivity, so that their sensitivities are higher than that of xRead but precisions are lowered. Minimap2 and BLEND try to recover read overlaps comprehensively. Minimap2 outputs the highest number of read overlaps and achieves the highest overall sensitivities on all the datasets, while BLEND achieves higher precision at the cost of the sensitivities, possibly due to its ability to find

both fuzzy and exact seed matches. However, the precision of Minimap2 is lowest, i.e., there are lots of false positives in the graphs, not only for the large but also the relatively small genomes, such as *A. thaliana* and *D. melanogaster*.

We further investigated more detailed information of the produced graphs with three additional metrics, R%, C% and Con. Num. The results indicate that the xRead graphs are connective and also have the potential to achieve high sensitivity through additional transitive operations if required.

R% indicates the proportion of the reads having at least one ground truth overlap being recovered. This metric indicates the proportion of the reads meeting the least requirement to correctly connect to the graph. The result (Fig. 2c and Supplementary Table 4, R% column) suggests that Minimap2 has the highest R% due to its sensitivity. For other tools, their R% is also very high and comparable to that of Minimap2, indicating that most of the reads have one or more correct overlaps being recovered. For xRead, this indicates that most of the reads can be correctly aligned to seed-reads. Therefore, it also holds the possibility of comprehensive graphs with post-processing. Moreover, for all the tools, R% is improved on high-quality datasets, suggesting that the true positive overlaps are more distinguishable from those false positives with the improved data quality.

C% indicates the percentage of the donor genome being covered by the connected reads. Herein, connected reads indicate the reads having at least one edge (overlap) in the produced graph. This metric reflects the coverage of the graph to the donor genome which is fundamental to complete assembly. It was observed that nearly all the tools achieved very high C% (close to 100%) for various datasets, indicating that the produced graphs had non- or very few uncovered regions (the C% and Gap Num. columns of Supplementary Table 4). For xRead, this result suggests that the seeds and their connected reads can cover the whole genome, which is helpful to fully recover the genome sequence. Only the Gap Num. of BLEND becomes obviously higher on low-quality datasets, indicating that it could not handle low-quality reads in some genomic regions.

Con. Num. indicates the number of connected components in produced graphs which measures their connectivity. It was observed (Con. Num. column of Supplementary Table 4) that on various datasets nearly all the produced graphs had relatively few connected components. This is partially due to the superior read lengths, meanwhile, it also indicates that tools enable the effective alignment and connection of the reads. Moreover, the Con. Num. of all tools decreased on high-quality datasets, suggesting that more potential connections can be revealed with improved sequencing quality. With the highly connected graphs, all the tools have the chance to indirectly infer all the read overlaps in the same components via transitive relationships and achieve high sensitivity. For xRead, the number of connected components is comparable to that of other tools on various datasets, however, it slightly increased for some of the genomes. We investigated the graphs and found that most of the reads were

connected and clustered in a few components, and other components only had very few (mostly two or three) reads. This is mainly due to that a small proportion of selected seed-reads are error-prone and some of the reads were aligned to them by accident. It usually happened in large datasets, since more outliers having serious sequencing errors occurred with increased read numbers. Other tools also had such a trend, meanwhile, by filtering out those extremely small ones, all the tools had very similar numbers of connected components.

Considering the precision, coverage and connectivity, we realized that the graphs produced by xRead are suited to function as a core graph to guide successful read assembly or error correction, referring to previous studies [22]. Furthermore, xRead also provides an additional tool to optionally expand the core graphs to meet the requirements of various genome assembly approaches. Mainly, it is implemented by a transitive rule-based width-first searching approach to iteratively recover the overlaps among non-seed-reads (refer to Methods section for more details). The tool enables to recover as many overlaps as possible. Meanwhile, it also supports fine-tuning the number of iterations for various tradeoffs between sensitivity and precision. The overall sensitivities of the graphs expanded by 1, 3 and 5 iterations are in Supplementary Table 5. It is observed that the sensitivity of the produced graphs greatly improved by even only one iteration. Moreover, the number of overlaps saturated after 5 iterations for most of the reads and the sensitivity metrics (overall sensitivity and R%) can be higher or close to that of Minimap2.

3) xRead keeps high yields along the whole genomes.

We assessed the read overlaps in various local regions to track the behaviors of xRead under various genomic contexts. Mainly, the reference genome was split into non-overlapping blocks (size: 1kbp and 10kbp for small and large/very large genomes, respectively) and the reads were assigned to corresponding blocks according to the ground truth. Further, the false positive overlap rates (FPR) and R% in various blocks were separately calculated. Herein, for a given block, FPR is defined as the proportion of false positives against all overlaps that at least one of the involved reads belongs to the block, and R% is the proportion of the reads belonging to the block that has at least one ground truth overlap recalled. The violin plots of FPR and R% for various datasets are in Fig. 2d and e.

The FPR plots show that xRead is able to keep relatively low FPR along the whole genome and has a significantly higher number of zero FPR blocks. We further investigated the high FPR blocks of xRead and found that most of them are of complex repetitive regions. Such cases are quite complicated and challenging due to the combination of high similarity of repeat copies and sequencing noise. An example is the *Z. mays* dataset where the genome is highly repetitive and all of the tools cannot maintain very low FPR. However, with the relatively conservative CRO strategy, xRead still maintained relatively high precision, i.e., for most of the blocks it had <20% FPR while other tools produced ubiquitous high

FPR (>80%) blocks.

The R% plots show that all of the tools have high R% along the whole genome, i.e., they have the ability to recover true positive overlaps in various regions. For xRead, this derives from the nearly non-gap coverage of the whole genome by the seed-reads and the high ability of alignment skeletons to capture the non-seed-reads from the same local regions. We further investigated those rare low R% blocks of xRead and observed that they also concentrated in highly repetitive regions. This is mainly due to that the reads from such regions, especially the ones having relatively short lengths and lower repeat spanning ability, are more likely to be aligned to the seed-reads from other copies of the same repeats and the incorrect CROs mislead the calculation of read coverage. Thus, such reads may have high coverage in the first several iterations of graph construction and lead to a collapsed graph. However, this issue does not affect the connectivity of the graph and the missing overlaps in the same repeat copy can still be recovered by transitive relationships. Thus, it keeps the chance for further steps (read correction and/or layout) to well-handle them and produce correct assemblies. Also refer to Discussion section for more detailed explanations.

Real-data benchmark for read-overlapping

The tools were further benchmarked with 7 real datasets from ONT and PacBio platforms (Table 1). The same limitation on RAM space was applied for xRead and no limitation for other tools. The performance, precision and sensitivity were assessed and similar trends were observed.

xRead still had high performance (Fig. 3a and Supplementary Table 6), i.e., overall faster speed with lower memory footprints than all of the other tools. It is also worth noting that xRead had higher performance on the ONT super high accuracy and PacBio HiFi datasets (several to tens of times faster than other tools), indicating that it is also suited to high-quality long reads. An exception occurred on the ONT fast mode human dataset that the speed of xRead is slower than that of MECAT2 and BLEND. It is mainly caused by the repeats of the human genome, i.e., xRead spent much time on the sorting and linking of the numerous minimizers from highly repetitive regions. Although higher, the time cost is still acceptable in absolute terms. The performance also shows that xRead is more suited to handle very large genomes. It is the only tool that accomplished the real *A. mexicanum* dataset successfully on the employed server. MHAP and wtdbg2 were out of memory (>1 Terabytes). MECAT2 raised an error signal and terminated. The speed of Minimap2 and BLEND was still very low like that of the simulated datasets and was early-stopped considering its very high estimated time cost.

The precision of xRead was still higher than that of other tools on the real datasets (Fig. 3b and Supplementary Table 7, Precision column). MECAT2 also achieved similar precision on smaller genomes, however, it decreased on the three human datasets, potentially due to the characteristics of

its DDF score model. The precision of wtdbg2 and BLEND was relatively high on PacBio datasets while significantly lowered on ONT datasets, indicating that they could be not suited to more error-prone reads. The precisions of MHAP and Minimap2 were significantly lower.

The overall sensitivities of the tools still varied due to their different designs, while Minimap2 was the highest one in contrast to its precision. On the R% metric (Fig. 3c and Supplementary Table 7, R% column), the tools were high and close to each other (except for wtdbg2 on ONT datasets), indicating that they potentially had similar abilities to detect the overlaps of real sequencing reads. Mainly, the reads having no true positive overlap recalled were from highly repetitive regions. In this situation, all of the tools were likely to be affected by the reads from various copies of the repeats and were hard to distinguish between true- and false positive overlaps effectively.

In addition, we also found another two issues that slightly lowered the R% of xRead. One is the crosstalk of the genomic repeats and the sequencing errors in some parts of the reads (see more detailed discussion below). The other is the lack of ground truth. That is, most of the ambiguous reads are potentially from the incomplete regions (i.e., the regions marked as "N" bases) of reference genome and cannot be mapped. The overlaps with such reads were discarded in the evaluation since their correctness cannot be determined. Thus, some of the reads were recognized as non-overlapped since the ambiguous reads were the seed-reads they overlapped with. We checked a portion of such overlaps and realized that they were also reasonable. That is, most of them were from ambiguous reads to the reads that can be mapped around the incomplete regions with high scores. Supplementary Table 8 gives more detailed information about the proportions of the reads having no correct overlap caused by various issues. Overall, considering that the R% of xRead is still high in absolute terms, it could be not problematic for genome assembly.

The metrics about the coverage and connectivity of the produced graphs were further assessed (C%, Gap Num. and Con. Num. columns of Supplementary Table 7). Similarly, the graphs are highly connected (i.e., relatively low numbers of connected components) and able to cover nearly the whole genomes with few gaps. It is also worth noting that for all of the tools the number of connected components slightly increased. This is also due to that real sequencing reads are more error-prone and likely to cause small components consisting of a few accidentally aligned reads. Partial evidence is that all the tools have obviously lower numbers of connected components on the human PacBio HiFi dataset than that of the ONT fast mode dataset. However, such small and noisy components could also not seriously affect genome assembly since it is not difficult to implement filtration by the size and quality of the overlaps. Furthermore, we also implemented graph expansion for the graphs produced by xRead (Supplementary Table 9). Consistent with that of simulated datasets, i.e., the overall sensitivity of the graphs obviously improved and nearly saturated after 5 iterations.

The read overlaps in local regions were also investigated. The violin plots of FPRs and R% of various genomic blocks are in Fig. 3d and e. xRead had the highest number of blocks with zero FPR and the distributions of the R% of various tools were quite similar. Moreover, it was observed that most of the reads having false positive and missing overlaps concentrated in a few blocks, leading to local high FPRs and low R%. We further investigated those blocks and found that the false positives mainly derived from highly repetitive regions. However, by separately investigating low R% blocks, we observed that the overlaps missed by xRead were caused by more complicated interactions of sequencing errors and repeats as follows.

Firstly, most of the missing overlaps were caused by error-prone read parts. In real data, some parts of the long reads have very high errors (especially ONT reads), so there is a lack of matches in those local regions. If the error-prone part is very long and in the inner region of the read, a lowly scored skeleton could be produced and filtered out by xRead. Another case is as mentioned above that the low-quality part is near either of the ends of the read where xRead could skip the skeleton due to the placement of the overlap.

Secondly, a small proportion of the missing overlaps were due to repeats as well as the design of xRead. During the construction of alignment skeletons, xRead initially does not use highly repetitive minimizers which occur more than a threshold of times in the index since most of them bring false positive matches which may affect both precision and performance. In most cases, the remaining minimizers are enough to build correct alignment skeletons. However, some of the reads from repetitive regions could lack matches and xRead employs an additional approach to solve the problem. That is, for the low-scored skeletons having a high number of repetitive minimizers, xRead uses the initial matches of the less repetitive minimizers as anchors and re-searches the matches between repetitive minimizers around the anchors to refine the skeletons. This helps to resolve most of the repetitive reads. However, in very rare cases the reads are short and enclosed in long repeats. Their skeletons are of low specificity and could bring many false positives so that xRead discards them.

The use of xRead for de novo assembly

The effect of xRead on genome assembly was further assessed. We tried to integrate xRead into a couple of state-of-the-art assemblers, such as NextDenovo [29], Wtdbg2 [17], Hifiasm [32], Shasta [18], Flye [25] and Canu [15]. This is non-trivial since for most of them the various modules (such as overlapping, layout and consensus) are coupled with specific in-memory data structures (also lack of published details) which do not allow flexible plug-ins. Under such circumstances, we made efforts to build an in-house assembly pipeline that uses the xRead graph as input and borrows two open-source modules from other assemblers, i.e., the layout module of NextDenovo (NextGraph) and the consensus module of Wtdbg2 (wtpoa-cns). Further, an in-house post-processing script developed in one of our

previous T2T-assembly studies [50] was used to reduce the structural mis-assemblies caused by the incompatibility of the modules. In the benchmark, we assessed the final assemblies (termed as xRead-pipe) as well as the intermediate outputs through NextGraph and wtpoa-cns modules (termed as xRead-nd and xRead-wtpoa respectively).

Twelve simulated datasets (Table 1) from four genomes (*E. coli, A. thaliana, D. melanogaster* and *H. sapiens*) in three error models (low-quality ONT, high-quality ONT and PacBio HiFi sequencing) were used for benchmarks. Moreover, three ONT real datasets (*C. elegans, D. melanogaster* and *H. sapiens*) and a human HiFi dataset (Table 1) were also employed. We tried to implement six pipelines (i.e., xRead-pipe, NextDenovo, Wtdbg2, Flye, Shasta and Hifiasm) on all the 12 (simulated) + 4 (real) datasets for comparison. Since Shasta is not suited to low-quality ONT datasets and Hifiasm is specifically designed for HiFi data, their corresponding results were excluded. QUAST [42] (version 5.2.0) was employed to evaluate the assemblies by the following metrics: assembly length, the number of contigs, N10/N50/N90 statistics, genome fraction, the number of all- and structural mis-assemblies. It is worth noting that we distinguished the assembly errors by local sequence errors (small-scale sequence expansions and collapses) or structural errors (large-scale misjoins) since the latter are usually much more difficult to resolve and should be more focused.

The results are in Table 2 (real) and Supplementary Tables 11-12 (simulated), respectively. It indicates that the completeness, correctness and contiguity of xRead-pipe are close to that of state-of-the-art assemblers, while its performance is obviously higher. Some intermediate results also suggested that some features of xRead were also not being fully utilized by this straightforward integration and it also has the potential to further improve assembly quality by more tailored layout and consensus modules. Mainly, three issues were observed as follows.

1) xRead-pipe reduces the overall computational cost of genome assembly.

For nearly all the datasets, both the runtime and memory usage of xRead-pipe is lower than most of the other assemblers (Table 2 and Supplementary Table 11, Real time and Peak Memory columns), suggesting that xRead is beneficial to the overall performance of assembly. Especially, xRead-pipe outperforms the original NextDenovo and Wtdbg2 pipelines by on average 2.5 times speedup and 4.2 times lower memory footprints. It is also worth noting that Shasta is the fastest one on human datasets while it also has the highest memory footprints, indicating a tradeoff between time- and memory cost. One example is that Shasta has a 776 GB peak memory on the simulated human HiFi dataset (17 and 37 GB for xRead-nd and xRead-wtpoa, respectively) which is a non-neglectable requirement for computational resource and could affect its scalability due to hardware limitation.

2) xRead-pipe is able to produce correct assemblies.

The QUAST evaluation (Table 2, Supplementary Table 12) indicates that overall xRead-pipe made comparable or lower numbers of mistakes than that of other pipelines, with quite similar assembly completeness (assembly lengths and genome fractions). This suggests that, using xRead graph, the assembly quality of the pipeline can reach the same level of state-of-the-art tools. We further investigated the types of mis-assemblies and found that, for all the assemblers, most of the mis-assemblies were local sequence errors and the numbers of structural errors were much lower. Especially, the assemblies of xRead-pipe had the lowest numbers of structural errors among all the assemblers. This should be praised since the small errors are easier to fix by advanced error correction and consensus sequence generation approaches. However, nearly all the large misjoins are related to complex repeats and are non-trivial to deal with.

The results of xRead-nd, xRead-wtpoa and xRead-pipe showed decreasing numbers of mis-assemblies, indicating the improvement in correctness by various modules. We further tracked their behaviors and found that a primary cause of the errors was the incompatibility of the layout module. That is, NextDenovo is in a correction-then-assembly design that takes advantage of a tailored correction module to reduce sequencing errors and re-cluster reads in advance. This is critical to read layout, however, it does not allow xRead graphs as input. Under such circumstances, the forced layout module (NextGraph) produced more errors. Further, a large proportion of the errors were amended by wtpoa and the in-house correction script. Wtpoa mainly improved the quality of consensus sequence and reduced the number of sequence errors, especially the ones caused by ONT sequencing noise. It also rescued a proportion of structural error. Further, by contig realignment (refer to Methods section), the in-house script more effectively detected misjoin regions and largely eliminated the structural errors. With the two modules, xRead-pipe is able to catch up or outperform the original NextDenovo pipeline (and other assemblers as well) at assembly correctness.

There are still errors caused by the crosstalk of xRead and NextGraph that cannot be eliminated. One is due to the very long repeats such as segmental duplications that the read length is still not enough to solve them thoroughly. Other assemblers also have such problems as well. On the other hand, it is also non-neglectable that NextGraph may not fully take advantage of xRead. In the xRead graphs, seed-reads are critical to connect the various parts of the donor genome. They should be given higher weights and used as the backbone during assembly. However, NextGraph does not have such a weight-tuning function. It implemented graph simplification by iteratively resolving nonlinear structures using some heuristics based on read overlap lengths, identities and depths. With its own heuristics, some of the overlaps between seed-reads were mistakenly discarded and leaving structural errors. A typical case is the removal of the z-clip structure (an example is shown in Supplementary Figure 2), i.e., due to the lack of adaptive weighting, some critical edges in nonlinear structures like z-clip structures were mistakenly removed and resulted in the misjoins between non-successive genomic parts.

3) xRead-pipe has comparable continuity to state-of-the-art approaches.

The results (Table 2 and Supplementary Table 12, N10/N50/N90 columns) suggest that the continuity of xRead-pipe is comparable to or slightly lower than that of other assemblers. We investigated the intermediate results of xRead-pipe and found that the decreased continuity is caused by the removal of some critical edges by NextGraph which made assembly gaps. For most of the assembly gaps in the results of xRead-pipe, the successive genome parts were initially connected in the graphs of xRead. The primary cause is still the heuristics of NextGraph to handle the z-clip structures. As mentioned above, the seed-reads in z-clip structures should serve as the backbone of the xRead graph. However, NextGraph selected the branches by its own rules and did not consider the importance of seed-reads, thus mistakes occurred (an example is in Supplementary Figure 3, and a more complicated one is in Supplementary Figure 4). Therefore, to further improve assembly continuity, a tailored layout method should be developed for xRead to better use the topology as well as other evidence provided by the graph.

Moreover, it is also non-neglectable that a tradeoff can be observed between the continuity and correctness of assembly. That is, the assemblers producing longer contigs also made higher numbers of structural errors. Such errors have no correlation with the lengths of contigs (Supplementary Figure 5) but are related to genomic contexts such as repeats, indicating that they could be partially caused by some relatively aggressive repeat-handling strategies of the assemblers. We realigned the error-containing contigs and investigated the mapping positions of their various parts. With the annotation of RepeatMasker, we found that the errors mostly happened around those well-known repetitive elements such as SINE, LINE, simple tandem repeats and centromeric repeat patterns.

It is worth noting that the errors may also occur in very long contigs which are usually produced with high confidence. An example is in Supplementary Figure 6. This is a contig of Flye whose length is 50.0 Mb, close to its N10 statistics. A clipping was detected around contig_2932: 42,085,000 which indicates a translocation. The contig was then split into two segments (42.1 Mb and 6.9 Mb, respectively) which can be aligned to reference at the positions Chr3: 88,844,116 and Chr6: 19,746,930, respectively. Another example is in Supplementary Figure 7. This is a contig of NextDenovo whose length is 43.5 Mb, also close to its N10 statistics. A clipping event was observed at ctg003710: 12,544,314, indicating a translocation error in the contig. The contig then can be split aligned to reference at two positions, i.e., Chr3: 91,045,032 and Chr6: 57,667,858.

Discussion

Long-read sequencing technologies are promising for the high-quality genome assembly of various species while they also make new requests to efficiently process thousands of genomes, tens of

gigabase-level assembly sizes and terabase-level datasets. Read-overlapping graph construction is the most computationally intensive step in this task and especially gets challenged. Herein, we propose xRead, a highly scalable graph construction approach. With its novel coverage-guided model and lightweight alignment skeletons, xRead implicitly explores the reads from various parts of the donor genome and converts the construction task to a fast read-mapping which substantially improves the overall performance. Moreover, the produced graph can be also well-connected, i.e., the reads are comprehensively connected with few false positive overlaps.

xRead is to some extent designed in a minimalist style that constructs a simplified, but not over-simplified, overlapping graph. The graph can support both of the two strategies commonly used by the state-of-the-art assemblers, i.e., correction-then-assembly [14, 15, 32] and assembly-then-correction [17, 18, 51]. For correction-then-assembly, the approach can implicitly cluster the reads of the same genomic regions since all of them can be aligned to the seed-read(s) from there. Therefore, the graph becomes a suitable input to read correction. For assembly-then-correction, xRead also paves the way to successful layout since the graph is highly connected with few false positive edges. Further, the alignments between non-seed- to seed-reads can also be directly used to infer local consensus in the correction phase. With these features, xRead lays a foundation for downstream assembly steps. It is also worth noting that, the seed-reads are natively the representatives of various genome parts. Thus, they should be seen as the backbone of assembly and well-handled by approaches specifically designed for either of the two strategies.

xRead has the potential to convert its produced graph to other styles, such as restricted to the best- or containing all the significant overlaps, which are popularly used strategies by other overlapping tools. This is done by using transitive relationships. It is not difficult for a seed-read to collect all the reads overlapping with it since such reads are either directly aligned to it, or aligned to another seed-read in the same connected component. For the latter case, the assumption also stands due to both the high connectivity (i.e., seed-reads are well-connected as mentioned) and precision (i.e., in most cases, all the transitive edges/overlaps are true positive) of the graph. Thus, the overlaps to the seed-read can be thoroughly evaluated to find out the best or all the significant ones. It is similar to a non-seed-read, i.e., the read is initially aligned to a seed-read and then involved in a connected component. Further, the overlaps to the non-seed-read can be inferred and evaluated through all the overlaps implied by the component. This feature is partially demonstrated by simulation and real benchmarks (Supplementary Tables 5 and 9), also refer to Supplementary Figure 8A for schematic illustrations.

xRead still has some limitations to handle the reads from ultralong repeats which is also a common problem to all read overlapping tools. As the lengths are not enough to span such repeats, the reads from various copies are not distinguishable with the crosstalk of their intrinsic similarity and sequencing

noise. Under such circumstances, xRead could align the seed-reads from various copies together and map non-seed-reads to some of them randomly. Then all the reads are connected as one component and the graph collapses, which is also a common case during read layout (refer to Supplementary Figure 8B for schematic illustrations). A feasible solution is to use tailored approaches to precisely correct sequencing errors and re-cluster the reads. With the improved base quality, the reads would have a good chance to be distinguished and the graph can be corrected. xRead also makes its own efforts to reduce this effect by two means. One is the CRO-based alignment skeletons which restricts highly confident overlaps and reduces the chance of mapping a read to a wrong copy. Another is that xRead outputs the inferred coverage for each of the reads (in its PAF format output, CV FLAG). This is a good repeat-indicating marker since the repetitive reads have obviously higher coverage, furthermore, they can be conveniently focused in later steps.

The use of xRead for whole genome assembly was also evaluated although it is hard to integrate a standalone overlapping tool into the state-of-the-art assemblers since most of their modules are coupled by various heuristics and do not allow third-party plug-ins. We tried our best to build a pipeline by borrowing the layout and consensus modules from NextDenovo and Wtdbg2, respectively. Considering both the time cost and RAM usage, the pipeline showed higher performance and scalability, meanwhile, it also achieved the same order of completeness, correctness and contiguity as that of those state-of-the-art assemblers. The results suggest that xRead is promising to scalable assembly. Further, we also would like to claim that the ability of xRead is not fully exerted with the straightforward (to some extent rough) integration. For example, due to the lack of a readily made module, this pipeline can still not fully take advantage of precise read correction (which is critical to repeat-handling). Moreover, existing layout methods also could not fully consider the characteristics of the graphs produced by xRead. It is an important future work (has been ongoing) to develop novel tools based on xRead graph to achieve high-quality, efficient and scalable genome assembly. We realize that there could be three key points to the development as follows.

Firstly, it is critical to develop a tailored read correction method for xRead. The seed-reads can be directly used as anchors to cluster the reads from the same genomic regions. Thus, read correction can be straightforwardly implemented by similar approaches to that of state-of-the-art assemblers, such as multiple sequence alignment [52], pseudo variant calling [53], and sequence graph analysis [14]. Moreover, it also needs to develop novel methods to use the correction information to distinguish and re-cluster the reads from various repeat copies to refine the graph.

Secondly, it also needs to design a specific read layout method with xRead graph. That is, various weights should be added to the edges and carefully considered since the connectivity of the CRO graph depends on the edges and short-paths between seed-reads and they should be well-handled. It is

feasible to build an essential backbone by seed-reads as they are well connected and implicitly distributed along the whole genome. Moreover, non-seed-reads can be used as extra evidence to rescue unconnected contigs, prevent mis-assemblies, and support the consensus phase.

Thirdly, it is still an open problem to implement efficient haplotype assembly. For xRead, one feasible way is to expand the graph dynamically with the transitive relationships during layout and precisely analyze the alignments among reads to adaptively reconstruct haplotype-specific paths. Another one is to use the initially assembled genome as reference to implement realignment, variant calling, and phasing, i.e., achieve haplotype assembly in a “de novo- and re-sequencing” way. This approach could be more suited to tasks having various types of reads (such as many T2T-assembly tasks) since the resequencing-style post-processing is more convenient to integrate the data and use various kinds of tools to correct mis-assemblies and construct haplotypes. Moreover, it is also feasible to keep low cost all the way, and even more, simultaneously handle many genomes in a step-parallel approach to achieve very high overall performance in large-scale genomics studies.

Methods

The selection and index of seed-reads

xRead initially selects P_0% of longest reads (default value: 3%) as seed-reads at first and assigns zero coverage to all the input reads. Other than the first iteration, xRead selects seed-reads with an updated profile of read coverage, i.e., P_s% of the low-covered reads (default value: 10%) are randomly selected as seed-reads, where the low-covered reads are defined by a threshold T_{RC} derived from the average coverage of their read parts.

A minimizer-based index is then built for the seed-reads. Given a seed-read, a set of windows of size W_{SR} (default value: 5bp) starting at every single base are defined and all the *k*-mers (default value: 15bp) within the windows (for both of the strands) are input into a hash function. The *k*-mer with minimum hash value is chosen to define a quadruple minimizer $(V_{SR}, R_{SR}, P_{SR}, S_{SR})$, where V_{SR}, R_{SR}, P_{SR} and S_{SR} indicate the hash value, the read, the position, and the strand of the minimizer. All the minimizers of the seed-reads are recorded and sorted by their hash values for indexing, moreover, a hash table of *l*-mers (default value: 11bp) is also built as an auxiliary index data structure to accelerate the retrieval and matching of minimizers in the following step.

Alignment skeleton-based read overlapping

xRead defines all the reads (all the low covered reads) as query reads in the first iteration (other iterations), and aligns them to the selected seed-reads to discover new overlaps to construct (refine) the overlapping graph. The alignment is inspired by deSALT [45] and implemented in a modified minimizer-based approach which is suited to the detection of read overlaps. Given a query read, xRead

collects all its minimizers using the same hash function at first. The minimizers are matched to seed-reads through the read index and xRead separately merges co-linear matches within the same seed-reads to build a set of match blocks (MBs).

xRead uses the MBs as vertices to construct a direct acyclic graph (DAG). Two MBs from the same seed-read define an edge if they meet the following conditions:

$$D_q > -k, D_s > -k, \left|D_q - D_s\right| < \delta \times \min\left(D_q, D_s\right) \quad (1)$$

where D_q and D_s are the distances between two MBs on the query and seed-reads, respectively, k is the maximum allowed overlap length between MBs, and δ is a parameter to limit the length difference between D_q and D_s. The weight and penalty are also assigned to each edge based on the number of covered bases and the distance between two nodes. The path with the highest score is then inferred in a sparse dynamic programming (SDP) approach by the following recursive equation and is considered the alignment skeleton.

$$S\left(MB_j\right) = \max\left\{S(MB_i) + w\left(MB_i \to MB_j\right) - p\left(MB_i \to MB_j\right)\right\}, MB_i \in Precusor\left\{MB_j\right\} \quad (2)$$

where $S\left(MB_j\right)$ is the score of the vertex MB_j, $w\left(MB_i \to MB_j\right)$ is the weight of the edge $MB_i \to MB_j$, and $p\left(MB_i \to MB_j\right)$ is the penalty of the edge $MB_i \to MB_j$.

It is also worth noting that multiple alignment skeletons could be built in practice since a query read usually has true positive overlaps to multiple seed-reads. More precisely, xRead removes all the MBs along the path after an alignment skeleton is built. Another skeleton is then built with the updated DAG. The iterative process goes on until no alignment skeleton with high scores can be built.

The construction and refinement of overlapping graph

xRead keeps a global graph data structure to record read overlaps during the iterative process. The produced alignment skeletons are converted to read overlapping information and supplied to the data structure incrementally. For a given query read, the produced alignment skeletons that meet one of the following three conditions are filtered out at first since they could be false positives caused by sequencing errors or repeats in local genomic regions: 1) the overlap length is shorter than $\mathrm{T_{OM}}$ (default value: 500bp); 2) the total number of non-redundant bases of all the MBs is lower than $\mathrm{T_{NB}}$ (default value: 100bp); 3) the overhang length of either read is longer than $\mathrm{T_{OH}}$ (default value: 2000bp). Further, xRead selects confident read overlaps (CROs), i.e., the $\mathrm{N_{AS}}$ (default value: 2) highest scored ones of the remaining alignment skeletons, and records their positions in the corresponding two reads.

xRead (re-)estimates read coverage with the updated overlapping information. For a given read, its coverage is estimated by the numbers of the seed-reads directly connected to it by the CROs and the reads having CROs to the same seed-reads which can be regarded as indirectly aligned to it. It is

also worth noting that there could be a proportion of reads being partially overlapped, i.e., some of their read parts have a high number of CROs while other parts have few. Under this circumstance, xRead implements a more precise local estimation, i.e., it splits the given read by $\mathrm{W_{RC}}$ size non-overlapping windows (default value: 1000bp) and separately estimates the coverage of various windows by the reads directly and indirectly connected to them. Further, xRead computes the average of the window coverage as the estimated coverage for a read.

The distribution of the coverage of various reads is then estimated and the medium of read coverage $\mathrm{M_{RC}}$ is computed. A threshold $\mathrm{T_{RC}}$ is set as $\mathrm{M_{RC}} \times P_{RC}$ for the selection of seed-reads in the next iteration where P_{RC} is a user-defined parameter (default value: 0.5). With given $\mathrm{T_{RC}}$, xRead monitors the number of newly selected seed-reads. If it is too low, xRead considers that there are few reads being lowly covered and outputs the resulting graph in PAF format.

The inference of comprehensive overlaps

The graph produced by xRead can be regarded as a core graph consisting of the overlaps between the seed and query reads. As some of the de novo assembly approaches require comprehensive read overlapping information, xRead provides an additional function to infer the overlaps between non-seed-reads and produce a more comprehensive graph. Mainly, it implements an iterative width-first searching approach based on the transitive relationships among the overlaps. That is, for each of the non-seed-reads, xRead initially retrieves all the other reads connected to the same seed-read(s) it attached and infers the overlaps via the transitive relationships. The length and placement of the inferred overlaps are investigated and the ones meeting the conditions similar to that of CROs are remained. Further, the remained overlaps are added to the graph as virtual edges and xRead further expands the graph through them in the following iterations. The transitive-overlap-based inference continues until no new legal overlap is found or it reaches a pre-defined number of iterations.

Assessment of the sensitivity and precision in produced graph

We use both simulated and real datasets in various read lengths and quality to evaluate the ability of xRead. The precision and sensitivity of the produced graph were assessed with the ground truth edge set of the overlapping graph (short as ground truth overlap set). The ground truth overlap set was generated based on the genomic positions of the reads. For simulated datasets, the read positions are directly given by the output files of the simulator (PBSIM). For real datasets, due to the absence of ground truth, we take advantage of the high mappability of long reads to produce pseudo-ground truth. That is, the reads were aligned to the corresponding reference genome using Minimap2 with default settings. The reads being unaligned or in low mapping quality were marked as ambiguous reads and filtered out, i.e., unused in the benchmark. Further, the remaining reads as well as their mapping

positions were used to compose the pseudo-ground truth set. The overlaps between reads were then collected based on (pseudo-) ground truth read positions and used to produce the (pseudo-) ground truth overlap set. Since too short overlaps could be caused by coincidence and most of them could be directly removed in downstream assembly steps, herein, only the read overlaps longer than 500bp were considered in the evaluation. This criteria also refers to previous studies [13].

The generated (pseudo-) ground truth overlap set was then used to evaluate the precision and sensitivity. Any reported overlap was considered a true positive only if it matched an overlap in the ground truth overlap set. It is worth noting that a read from a real dataset may have multiple positions due to the ambiguity of alignment. In such cases, we consider an overlap to be true positive if it matches any of the overlaps derived from corresponding reads in the ground truth set. Any non-true-positive overlap is seen as a false positive overlap. The precision and sensitivity were then calculated as $\mathrm{N}_{\mathrm{O}}^{TP}/N_{O}^{R}$ and $\mathrm{N}_{\mathrm{O}}^{TP}/N_{O}^{G}$, where $\mathrm{N}_{\mathrm{O}}^{TP}$, N_{O}^{R} and N_{O}^{G} are the number of true positive overlaps, reported overlaps, and overlaps in the ground truth set, respectively.

The implementation of benchmarks

We implemented benchmarks of read overlapping on simulated and real long-read datasets from nine genomes, i.e., *E. coli* (ASM584v2), *S. cerevisiae* (R64), *C. elegans* (WBcel235), *A. thaliana* (TAIR10.1), *D. melanogaster* (Release 6 plus ISO1 MT), *Z. mays* (subsp. *mays* SK) [54], *M. musculus* (GRCm39), *H. sapiens* (T2T-CHM13v2.0) and *A. mexicanum* (AmbMex60DD) [7]. Most of the datasets are ONT or PacBio CLR reads, and xRead was compared with five state-of-the-art tools, i.e., MHAP (version 2.1.3), MECAT2 (v20190314), Minimap2 (version 2.24), wtdbg2 (version 2.5) and BLEND (version 1.0.0). Moreover, a couple of in-house Python scripts were used to interpret and evaluate the outputs of the tools in various formats. Other state-of-the-art assemblers like Canu, Shasta, NECAT, Flye, and NextDenovo were not included in the read overlapping benchmarks since they do not provide stand-alone modules to output interpretable results, or employ generic alignment tools such as Minimap2 for read overlapping.

In addition, we implemented benchmarks of de novo assembly on 12 simulated and 4 real datasets. The twelve simulated datasets were from four genomes (*E. coli*, *A. thaliana*, *D. melanogaster* and *H. sapiens*) in three error models (low-quality ONT, high-quality ONT and PacBio HiFi sequencing). The four real datasets include three ONT datasets (*C. elegans, D. melanogaster* and *H. sapiens*) and a human HiFi dataset. We build an in-house assembly pipeline that uses overlapping graph of xRead, the layout module of NextDenovo (NextGraph), the consensus module of Wtdbg2 (wtpoa-cns) and an in-house correction script. The three pipelines (i.e., xRead-pipe, xRead-nd and xRead-wtpoa) are compared with five state-of-the-art assemblers (i.e., NextDenovo, Wtdbg2, Flye version 2.9.4-b1799, Shasta version 0.12.0 and Hifiasm version 0.19.9) by the evaluation of QUAST.

All the benchmarks were implemented on a server with 4 Intel Xeon 5220R CPUs (96 CPU cores in total) and 1 Terabyte RAM running Linux Ubuntu 16.04. Refer to Supplementary Tables 3, 4, 6, and 7 (Parameter columns) and Supplementary Note 1 for the detailed settings of the tools used in the benchmark.

Post-processing of xRead-pipe to mis-assembly correction

To correct structural errors in assembly, we used an in-house post-processing script developed in one of our T2T plant genome assembly studies [50]. The method is based on read-to-contig realignments which work in two simple steps, 1) it calculates the per-base depth of contigs to detect candidate misjoin regions; 2) it detects highly plausible structural errors and directly eliminates them. A brief description of its implementation is as follows.

In the first step, the method initially realigns the reads to the contigs and calculates the per-base depth of the contigs. A 10 kb sliding window is used to scan the contigs to collect abnormally high- and low coverages as well as read clipping signatures. It defines a high coverage region (HCR) as a set of consecutive windows whose average coverage is higher than three times the sequencing depth of the whole dataset, while a low coverage region (LCR) is defined as having average coverage lower than 15% of the sequencing depth. Clipping events (CLIPs) are also identified based on the large clippings implied by the realignments within windows. To avoid detecting heterozygous structural variants as false positive mis-assemblies, a window is considered as a CLIP only if over 60% of the reads in the window have large clippings. The HCR, LCR and CLIP regions are then combined to form a total set of candidates misjoin regions.

In the second step, the method further verifies mis-assemblies by several heuristics. The candidate regions are sorted by their genomic positions, and adjacent regions within 20 kb are merged and treated as a single region. HCRs and LCRs shorter than 20kb are filtered out to avoid local false positive regions, such as HiFi coverage gaps. Meanwhile, CLIPs within 10 kb of contig ends are also filtered out to avoid the artifacts from contig breakpoints. After filtration, the method simply discards the sequences around the marked structural-error regions and splits the contigs.

It is worth noting that the updated contigs could be shortened with the straightforward split operation. However, this restrict method is effective in preventing large misjoins, especially in highly repetitive regions. Moreover, a scaffolding method using long-range sequencing information (e.g., ultralong reads or Hi-C data) could be a good supplement to rejoin the contigs to achieve highly continuous assembly without loss of correctness.

Availability of Supporting Source Code and Requirements

Project name: xRead

Project home page: https://github.com/tcKong47/xRead

Operating system(s): Linux

Programming language: C

Other requirements: None

License: MIT license

RRID: SCR_025372

Additional Files

Supplementary Table S1. Detailed information of reference genomes.

Supplementary Table S2. Availability information of real datasets.

Supplementary Table S3. The performance of various tools on simulated datasets.

Supplementary Table S4. The yields of various tools on simulated datasets.

Supplementary Table S5. The sensitivity of the expanded graphs of xRead on simulated datasets.

Supplementary Table S6. The performance of various tools on real sequencing datasets.

Supplementary Table S7. The yields of various tools on real sequencing datasets.

Supplementary Table S8. Percentages of the reads not correctly overlapped by xRead with various causes.

Supplementary Table S9. The sensitivity of the expanded graphs of xRead on real sequencing datasets.

Supplementary Table S10. The external memory of various tools on both simulated and real datasets.

Supplementary Table S11. The performance of de novo assembly on simulated datasets.

Supplementary Table S12. Statistics of assembly results on simulated datasets.

Supplementary Figure S1. The performance and external memory usage on simulated and real datasets.

Supplementary Figure S2. An example of the mistaken selection of edges at branching paths by NextGraph.

Supplementary Figure S3. An example of the removal of critical edges of z-clip structure by NextGraph.

Supplementary Figure S4. An example of the removal of critical edges of z-clip structure by NextGraph.

Supplementary Figure S5. The number of mis-assemblies in contigs longer than N10/N50/N90 values of various assemblers.

Supplementary Figure S6. An example of the structural error in a contig of Flye on the real human PacBio HiFi dataset.

Supplementary Figure S7. An example of the structural error in a contig of NextDenovo on the real human ONT dataset.

Supplementary Figure S8. The schematic illustrations for the features of xRead approach.

Supplementary Note 1. The command and parameters used for assembly benchmarks.

Data Availability

The following reference genomes were used in this study: *Escherichia coli* (GCF_000005845.2), *Saccharomyces cerevisiae* (GCF_000146045.2), *Caenorhabditis elegans* (GCF_000002985.6), *Arabidopsis thaliana* (GCF_000001735.4), *Drosophila melanogaster* (GCF_000001215.4), Zea mays [55], *Mus musculus* (GCF_000001635.27), *Homo sapiens* CHM13 (GCF_009914755.1), *Homo sapiens* HG002 [56] and *Ambystoma mexicanum* (GCA_002915635.3). Real datasets used in this study can be accessed through the following accessions: *Escherichia coli* (SRR19746198), *Caenorhabditis elegans* (SRR19746198), *Drosophila melanogaster* (SRR13070625), *Homo sapiens* HG002 ONT datasets in fast base-calling mode [57], *Homo sapiens* HG002 ONT dataset in super high accuracy base-calling mode [58], *Homo sapiens* HG002 PacBio HiFi dataset [59] and *Ambystoma mexicanum* (SRR5349126-SRR5349175). Please refer to Supplementary Tables S1 and S2 for additional information. Additional codes underlying this study can be found in the GitHub repository [60]. An archival copy of the code is available via Software Heritage [61].

Abbreviations

SMRT: Single Molecule Real Time; CPU: Central Processing Unit; RAM: Random Access Memory; OLC: Overlap-Layout-Consensus; SIMD: Single Instruction Multiple Data; CUDA: Compute Unified Device Architecture; DDF: Distance Difference Factor; PacBio: Pacific Biosciences; ONT: Oxford Nanopore Technologies; GB: Giga Byte; CRO: Confident Read Overlap; GIAB: Genome In A Bottle; FPR: false positive overlap rates; MB: Match block; DAG: Direct Acyclic Graph; SDP: Sparse Dynamic Programming; PAF: Pairwise Alignment Format. HCR: High Coverage Region; LCR: Low Coverage Region; CLIP: Clipping event.

Consent for publication

Not applicable.

Competing interests

The authors declare that they have no competing interests.

Funding

This work has been supported by the National Key Research and Development Program of China (No: 2021YFF1200105) and the National Natural Science Foundation of China (No: 62172125).

Author contributions

TK implemented the method, BL designed the method, and TK, BL, and YW performed the analysis. All of the authors wrote the manuscript.

References

1. Eid J, Fehr A, Gray J, Luong K, Lyle J, Otto G, et al. Real-Time DNA Sequencing from Single Polymerase Molecules. Science. 2009;323 5910:133-8. doi:10.1126/science.1162986.
2. Mikheyev AS and Tin MMY. A first look at the Oxford Nanopore MinION sequencer. Molecular Ecology Resources. 2014;14 6:1097-102. doi:10.1111/1755-0998.12324.
3. Logsdon GA, Vollger MR and Eichler EE. Long-read human genome sequencing and its applications. Nature Reviews Genetics. 2020;21 10:597-614. doi:10.1038/s41576-020-0236-x.
4. Nurk S, Koren S, Rhie A, Rautiainen M, Bzikadze AV, Mikheenko A, et al. The complete sequence of a human genome. Science. 2022;376 6588:44-+. doi:10.1126/science.abj6987.
5. Garg S, Fungtammasan A, Carroll A, Chou M, Schmitt A, Zhou X, et al. Chromosome-scale, haplotype-resolved assembly of human genomes. Nature Biotechnology. 2021;39 3:309-12. doi:10.1038/s41587-020-0711-0.
6. Neale DB, Wegrzyn JL, Stevens KA, Zimin AV, Puiu D, Crepeau MW, et al. Decoding the massive genome of loblolly pine using haploid DNA and novel assembly strategies. Genome Biology. 2014;15 3 doi:10.1186/gb-2014-15-3-r59.
7. Nowoshilow S, Schloissnig S, Fei JF, Dahl A, Pang AWC, Pippel M, et al. The axolotl genome and the evolution of key tissue formation regulators. Nature. 2018;554 7690:50-+. doi:10.1038/nature25458.
8. Shao C, Sun S, Liu K, Wang J, Li S, Liu Q, et al. The enormous repetitive Antarctic krill genome reveals environmental adaptations and population insights. Cell. 2023; doi:10.1016/j.cell.2023.02.005.
9. Sovic I, Krizanovic K, Skala K and Sikic M. Evaluation of hybrid and non-hybrid methods for de novo assembly of nanopore reads. Bioinformatics. 2016;32 17:2582-9. doi:10.1093/bioinformatics/btw237.
10. Jayakumar V and Sakakibara Y. Comprehensive evaluation of non-hybrid genome assembly tools for third-generation PacBio long-read sequence data. Briefings in Bioinformatics. 2019;20 3:866-76. doi:10.1093/bib/bbx147.
11. Rhie A, McCarthy SA, Fedrigo O, Damas J, Formenti G, Koren S, et al. Towards complete and error-free genome assemblies of all vertebrate species. Nature. 2021;592 7856:737-+. doi:10.1038/s41586-021-03451-0.
12. Lewin HA, Robinson GE, Kress WJ, Baker WJ, Coddington J, Crandall KA, et al. Earth BioGenome Project: Sequencing life for the future of life. Proceedings of the National Academy of Sciences of the

United States of America. 2018;115 17:4325-33. doi:10.1073/pnas.1720115115.

13. Berlin K, Koren S, Chin CS, Drake JP, Landolin JM and Phillippy AM. Assembling large genomes with single-molecule sequencing and locality-sensitive hashing. Nature Biotechnology. 2015;33 6:623-+. doi:10.1038/nbt.3238.

14. Chin CS, Peluso P, Sedlazeck FJ, Nattestad M, Concepcion GT, Clum A, et al. Phased diploid genome assembly with single-molecule real-time sequencing. Nature Methods. 2016;13 12:1050-+. doi:10.1038/nmeth.4035.

15. Koren S, Walenz BP, Berlin K, Miller JR, Bergman NH and Phillippy AM. Canu: scalable and accurate long-read assembly via adaptive k-mer weighting and repeat separation. Genome Research. 2017;27 5:722-36. doi:10.1101/gr.215087.116.

16. Xiao CL, Chen Y, Xie SQ, Chen KN, Wang Y, Han Y, et al. MECAT : fast mapping, error correction, and de novo assembly for single-molecule sequencing reads. Nature Methods. 2017;14 11:1072-+. doi:10.1038/nmeth.4432.

17. Ruan J and Li H. Fast and accurate long-read assembly with wtdbg2. Nature Methods. 2020;17 2:155-+. doi:10.1038/s41592-019-0669-3.

18. Shafin K, Pesout T, Lorig-Roach R, Haukness M, Olsen HE, Bosworth C, et al. Nanopore sequencing and the Shasta toolkit enable efficient de novo assembly of eleven human genomes. Nature Biotechnology. 2020;38 9:1044-+. doi:10.1038/s41587-020-0503-6.

19. Bankevich A, Nurk S, Antipov D, Gurevich AA, Dvorkin M, Kulikov AS, et al. SPAdes: A New Genome Assembly Algorithm and Its Applications to Single-Cell Sequencing. Journal of Computational Biology. 2012;19 5:455-77. doi:10.1089/cmb.2012.0021.

20. Rautiainen M and Marschall T. MBG: Minimizer-based sparse de Bruijn Graph construction. Bioinformatics. 2021;37 16:2476-8. doi:10.1093/bioinformatics/btab004.

21. Bankevich A, Bzikadze AV, Kolmogorov M, Antipov D and Pevzner PA. Multiplex de Bruijn graphs enable genome assembly from long, high-fidelity reads. Nature Biotechnology. 2022;40 7:1075-81. doi:10.1038/s41587-022-01220-6.

22. Chin CS, Alexander DH, Marks P, Klammer AA, Drake J, Heiner C, et al. Nonhybrid, finished microbial genome assemblies from long-read SMRT sequencing data. Nature Methods. 2013;10 6:563-+. doi:10.1038/nmeth.2474.

23. Chaisson MJ and Tesler G. Mapping single molecule sequencing reads using basic local alignment with successive refinement (BLASR): application and theory. Bmc Bioinformatics. 2012;13 doi:10.1186/1471-2105-13-238.

24. Myers G. Efficient Local Alignment Discovery amongst Noisy Long Reads. In: *14th International Workshop on Algorithms in Bioinformatics (WABI)* Wroclaw, POLAND, Sep 08-10 2014, Algorithms in bioinformatics, pp.52-67.

25. Kolmogorov M, Yuan J, Lin Y and Pevzner PA. Assembly of long, error-prone reads using repeat graphs. Nature Biotechnology. 2019;37 5:540-+. doi:10.1038/s41587-019-0072-8.

26. Li H. Minimap2: pairwise alignment for nucleotide sequences. Bioinformatics. 2018;34 18:3094-100. doi:10.1093/bioinformatics/bty191.

27. Vaser R and Šikić M. Time- and memory-efficient genome assembly with Raven. Nature Computational Science. 2021;1 5:332-6. doi:10.1038/s43588-021-00073-4.

28. Nie F, Ni P, Huang N, Zhang J, Wang Z, Xiao C, et al. De novo diploid genome assembly using long noisy reads. Nature Communications. 2024;15 1:2964. doi:10.1038/s41467-024-47349-7.

29. Hu J, Wang Z, Sun Z, Hu B, Ayoola AO, Liang F, et al. NextDenovo: an efficient error correction

and accurate assembly tool for noisy long reads. Genome Biology. 2024;25 1:107. doi:10.1186/s13059-024-03252-4.

30. Schleimer S, Wilkerson DS and Aiken A. Winnowing: local algorithms for document fingerprinting. *Proceedings of the 2003 ACM SIGMOD international conference on Management of data*. San Diego, California: Association for Computing Machinery, 2003, p. 76–85.

31. Roberts M, Hayes W, Hunt BR, Mount SM and Yorke JA. Reducing storage requirements for biological sequence comparison. Bioinformatics. 2004;20 18:3363-9. doi:10.1093/bioinformatics/bth408.

32. Cheng H, Concepcion GT, Feng X, Zhang H and Li H. Haplotype-resolved de novo assembly using phased assembly graphs with hifiasm. Nature Methods. 2021;18 2:170-5. doi:10.1038/s41592-020-01056-5.

33. Firtina C, Park J, Alser M, Kim JS, Cali DS, Shahroodi T, et al. BLEND: a fast, memory-efficient and accurate mechanism to find fuzzy seed matches in genome analysis. Nar Genomics and Bioinformatics. 2023;5 1 doi:10.1093/nargab/lqad004.

34. Charikar MS. Similarity estimation techniques from rounding algorithms. *Proceedings of the thiry-fourth annual ACM symposium on Theory of computing*. Montreal, Quebec, Canada: Association for Computing Machinery, 2002, p. 380–8.

35. Manku GS, Jain A and Sarma AD. Detecting near-duplicates for web crawling. *Proceedings of the 16th international conference on World Wide Web*. Banff, Alberta, Canada: Association for Computing Machinery, 2007, p. 141–50.

36. Manavski SA and Valle G. CUDA compatible GPU cards as efficient hardware accelerators for Smith-Waterman sequence alignment. Bmc Bioinformatics. 2008;9 doi:10.1186/1471-2105-9-s2-s10.

37. Rognes T. Faster Smith-Waterman database searches with inter-sequence SIMD parallelisation. Bmc Bioinformatics. 2011;12 doi:10.1186/1471-2105-12-221.

38. Daily J. Parasail: SIMD C library for global, semi-global, and local pairwise sequence alignments. Bmc Bioinformatics. 2016;16 doi:10.1186/s12859-016-0930-z.

39. Suzuki H and Kasahara M. Introducing difference recurrence relations for faster semi-global alignment of long sequences. Bmc Bioinformatics. 2018;19 doi:10.1186/s12859-018-2014-8.

40. Rowe WPM. When the levee breaks: a practical guide to sketching algorithms for processing the flood of genomic data. Genome Biology. 2019;20 1 doi:10.1186/s13059-019-1809-x.

41. Chen Y, Nie F, Xie SQ, Zheng YF, Dai Q, Bray T, et al. Efficient assembly of nanopore reads via highly accurate and intact error correction. Nature Communications. 2021;12 1 doi:10.1038/s41467-020-20236-7.

42. Amarasinghe SL, Su S, Dong XY, Zappia L, Ritchie ME and Gouil Q. Opportunities and challenges in long-read sequencing data analysis. Genome Biology. 2020;21 1 doi:10.1186/s13059-020-1935-5.

43. Magi A, Semeraro R, Mingrino A, Giusti B and D'Aurizio R. Nanopore sequencing data analysis: state of the art, applications and challenges. Briefings in Bioinformatics. 2018;19 6:1256-72. doi:10.1093/bib/bbx062.

44. Carneiro MO, Russ C, Ross MG, Gabriel SB, Nusbaum C and DePristo MA. Pacific biosciences sequencing technology for genotyping and variation discovery in human data. Bmc Genomics. 2012;13 doi:10.1186/1471-2164-13-375.

45. Liu B, Liu Y, Li J, Guo H, Zang T and Wang Y. deSALT: fast and accurate long transcriptomic read alignment with de Bruijn graph-based index. Genome Biology. 2019;20 1:274. doi:10.1186/s13059-019-1895-9.

46. Liu Y, Jiang T, Su J, Liu B, Zang T and Wang Y. SKSV: ultrafast structural variation detection from circular consensus sequencing reads. Bioinformatics. 2021;37 20:3647-9. doi:10.1093/bioinformatics/btab341.

47. Ekim B, Sahlin K, Medvedev P, Berger B and Chikhi R. Efficient mapping of accurate long reads in minimizer space with mapquik. Genome research. 2023;33 7:1188-97.

48. Ono Y, Asai K and Hamada M. PBSIM2: a simulator for long-read sequencers with a novel generative model of quality scores. Bioinformatics. 2021;37 5:589-95. doi:10.1093/bioinformatics/btaa835.

49. Ono Y, Hamada M and Asai K. PBSIM3: a simulator for all types of PacBio and ONT long reads. Nar Genomics and Bioinformatics. 2022;4 4 doi:10.1093/nargab/lqac092.

50. Zhang A, Kong T, Sun B, Qiu S, Guo J, Ruan S, et al. A telomere-to-telomere genome assembly of Zhonghuang 13, a widely-grown soybean variety from the original center of Glycine max. The Crop Journal. 2024;12 1:142-53. doi:https://doi.org/10.1016/j.cj.2023.10.003.

51. Li H. Minimap and miniasm: fast mapping and de novo assembly for noisy long sequences. Bioinformatics. 2016;32 14:2103-10. doi:10.1093/bioinformatics/btw152.

52. Vaser R, Sovic I, Nagarajan N and Sikic M. Fast and accurate de novo genome assembly from long uncorrected reads. Genome Research. 2017;27 5:737-46. doi:10.1101/gr.214270.116.

53. Zheng ZX, Li SM, Su JH, Leung AWS, Lam TW and Luo RB. Symphonizing pileup and full-alignment for deep learning-based long-read variant calling. Nature Computational Science. 2022;2 12:797-+. doi:10.1038/s43588-022-00387-x.

54. Yang N, Liu J, Gao Q, Gui ST, Chen L, Yang LF, et al. Genome assembly of a tropical maize inbred line provides insights into structural variation and crop improvement. Nature Genetics. 2019;51 6:1052-+. doi:10.1038/s41588-019-0427-6.

55. Yang N. The Genome of Zea mays (SK) [Dataset]. China National Center for Bioinformatics. https://ngdc.cncb.ac.cn/gwh/Assembly/123/show. Accessed 6 Jan 2025.

56. Human Pangenome Reference Consortium. HG002 T2T assemblies (Version 1.1) [Dataset]. Amazon Web Services. https://s3-us-west-2.amazonaws.com/human-pangenomics/T2T/HG002/assemblies/hg002v1.1.mat.fasta.gz. Accessed 6 Jan 2025.

57. Human Pangenome Reference Consortium. ONT dataset in fast base-calling mode for HG002 [Dataset]. Amazon Web Services. https://s3-us-west-2.amazonaws.com/human-pangenomics/NHGRI_UCSC_panel/HG002/hpp_HG002_NA24385_son_v1/nanopore/HG002_ucsc_Oct_2018_Guppy_3.0.fastq.gz. Accessed 6 Jan 2025.

58. Oxford Nanopore Technologies. ONT dataset in super high accuracy base-calling mode for HG002 [Dataset]. Amazon Web Services https://labs.epi2me.io/gm24385_q20_2021.10/. Accessed 6 Jan 20

59. Genome in a Bottle Consortium. PacBio HiFi dataset for HG002 [Dataset]. NCBI Trace Archive. https://ftp-trace.ncbi.nlm.nih.gov/giab/ftp/data/AshkenazimTrio/HG002_NA24385_son/PacBio_CCS_15kb/alignment/HG002.Sequel.15kb.pbmm2.hs37d5.whatshap.haplotag.RTG.10x.trio.bam. Accessed 6 Jan 2025.

60. Kong T, Wang Y, Liu B. The source code files for xRead. GitHub. 2024. https://github.com/tcKong47/xRead. Accessed 6 Jan 2025.

61. Kong T, Wang Y, Liu B. xRead: A coverage-guided approach for scalable construction of read overlapping graph (Version 1.0.0.2) [Computer software]. Software Heritage. 2024. https://archive.softwareheritage.org/swh:1:dir:80e9649ddc30d2ba1171b75e9e125dbdad3dcef0;origin=https://github.com/tcKong47/xRead;visit=swh:1:snp:46fec48573910edfc6cb61f3597c55b20c2931de;anchor=swh:1:rev:b72eb036fd1c924bf641186d896e5b911ee74b13. Accessed 6 Jan 2025.

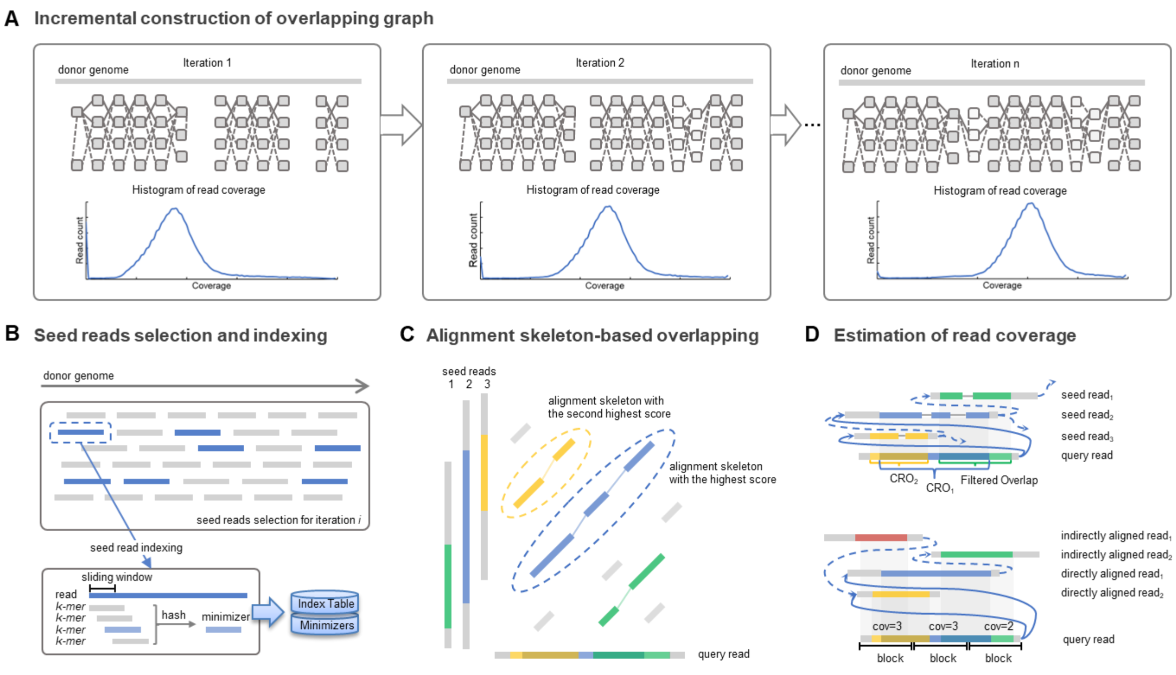

Fig. 1 A schematic illustration of xRead. **a.** The incremental construction of the overlapping graph. The subplots represent the connections among reads being incrementally recovered within various iterations. The gray blocks indicate the sequencing reads. The dashed and solid lines respectively indicate the overlaps between the seed and the query reads, and the seed-reads themselves. The histograms in the lower part indicate the distributions of read coverage which are updated in various iterations. **b.** The selection and indexing of seed-reads. xRead selects a portion of lowly covered reads as seed-reads (marked as blue bars) and a list of minimizers is extracted using a hash function. A minimizer-based index is then built by a hash table-based data structure. Meanwhile, the same hash function is also used to generate minimizers for query reads. **c.** Alignment skeleton-based read overlapping. For a given query read, xRead finds the MBs (marked as colored bars) between it and all the seed-reads via the index. Further, it uses the SDP approach to generate one or more alignment skeletons (the dashed ovals indicate the skeletons with the first and second highest scores). **d.** Estimation of read coverage. The upper subplot represents the selection of CROs. The first two highest-scored skeletons are selected as CROs which bring new edges (represented by solid lines) to the overlapping graph. Other skeletons with lower scores are filtered out. The lower subplot represents the (re-)estimation of read coverage. xRead splits the reads into non-overlapping blocks and then counts the reads directly (marked as solid lines) and indirectly (marked as dashed lines) being aligned to it.

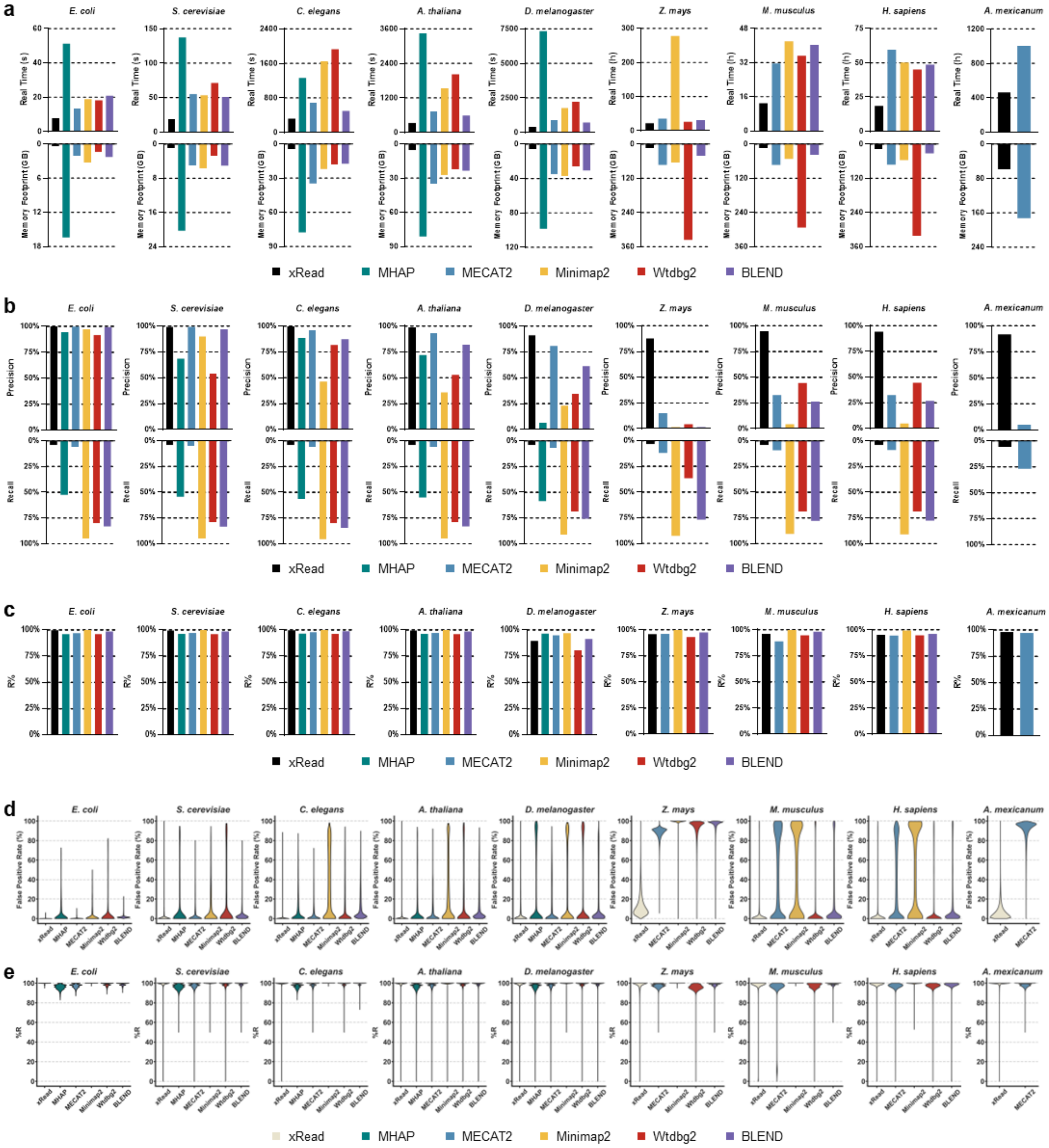

Fig 2. Results on simulated datasets. The figure depicts the real-time, peak memory (**a**), precision, sensitivity (**b**), R% (**c**), and violin plots of FPR (**d**) and R% (**e**) of each overlapper on nine simulated datasets of various-sized genomes (from *E. coli* to *A. mexicanum*). **a-c.** In each subplot of (**a**), (**b**), and (**c**), the colors black, green, blue, orange, and red respectively represent the overlapper xRead, MHAP, MECAT2, Minimap2, wtdbg2, and BLEND. **d-e.** The violin plot of FPR and R% for various datasets. For a given tool, the absence of the results for some datasets is due to its failure during benchmarking. Refer to supplementary material for detailed information on datasets and results.

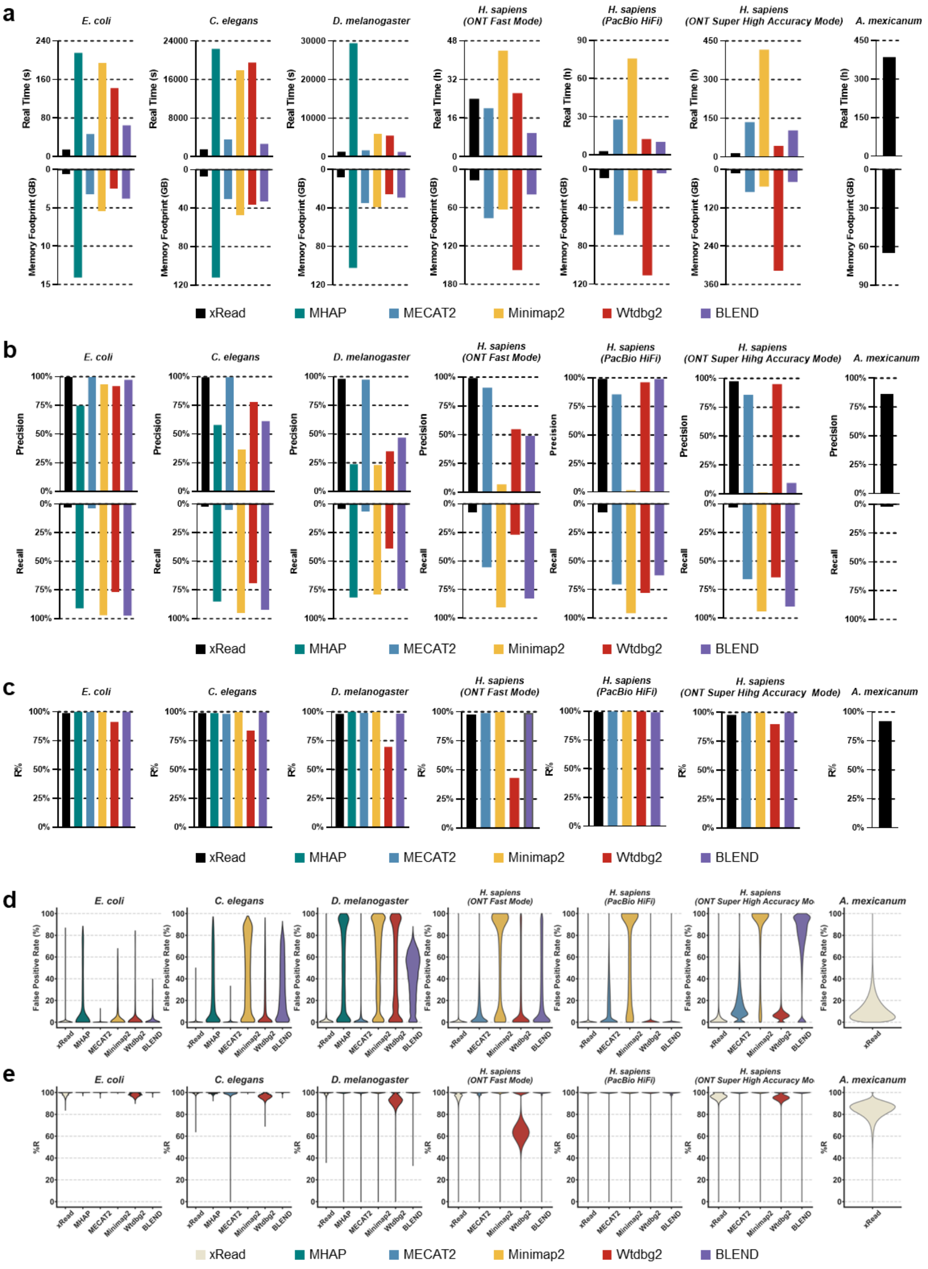

Fig 3. Results on real datasets. The figure depicts the real-time, peak memory (**a**), precision, sensitivity (**b**), R% (**c**), and violin plots of FPR (**d**) and R% (**e**) of each overlapper on seven real datasets of various-sized genomes (*E. coli, C. elegans, D. melanogaster,* three *H. sapiens* dataset from different platforms, and *A. mexicanum*). **a-c.** In each subplot of (**a**), (**b**), and (**c**), the colors black, green, blue, orange, and red respectively represent the overlapper xRead, MHAP, MECAT2, Minimap2, and wtdbg2, and BLEND. **d-e.** The violin plot of FPR and R% for various datasets. For a given tool, the absence of the results for some datasets is due to its failure during benchmarking. Refer to supplementary material for detailed information on datasets and results.

Table 1. Detailed information of simulated and real datasets

No.	Reference genome	Error model / Platform	File size	Num. of reads	Error rate	Ave. Read length	Coverage
**Simulated ONT datasets with an average accuracy of 87% [a]**							
1	*Escherichia coli*	R103	443 MB	15378	13.0%	13000	50x
2	*Saccharomyces cerevisiae*	R103	1.2 GB	40562	13.0%	13000	50x
3	*Caenorhabditis elegans*	R103	9.4 GB	334603	13.0%	13000	50x
4	*Arabidopsis thaliana*	R103	12 GB	398748	13.0%	13000	50x
5	*Drosophila melanogaster*	R103	14 GB	561293	13.0%	13000	50x
6	*Zea mays (SK)*	R103	202 GB	7724943	13.0%	13000	50x
7	*Mus musculus*	R103	255 GB	9376380	13.0%	13000	50x
8	*Homo sapiens*	R103	291 GB	12215872	13.0%	13000	50x
9	*Ambystoma mexicanum*	R103	2 TB	95447945	13.0%	13000	50x
**Simulated ONT datasets with an average accuracy of 94% [b]**							
10	*Escherichia coli*	QSHMM-ONT-HQ	443 MB	15613	6.0%	15000	50x
11	*Arabidopsis thaliana*	QSHMM-ONT-HQ	12 GB	402487	6.0%	15000	50x
12	*Drosophila melanogaster*	QSHMM-ONT-HQ	14 GB	561999	6.0%	15000	50x
13	*Homo sapiens*	QSHMM-ONT-HQ	291 GB	10475665	6.0%	15000	50x
**Simulated HiFi datasets with an average accuracy > 99.5% [c]**							
14	*Escherichia coli*	-	443 MB	13939	< 0.5%	16606	50x
15	*Arabidopsis thaliana*	-	12 GB	360265	< 0.5%	16606	50x
16	*Drosophila melanogaster*	-	13 GB	414157	< 0.5%	16606	50x
17	*Homo sapiens*	-	291 GB	9385394	< 0.5%	16606	50x
**Real datasets**							
18	*Escherichia coli*	ONT R9.3	775 MB	34389	-	11766	86x
19	*Caenorhabditis elegans*	ONT R9.3	21 GB	789871	-	13724	108x
20	*Drosophila melanogaster*	ONT R9.3	14 GB	640215	-	11142	50x
21	*Homo sapiens*	ONT R9.3	162 GB	10836442	-	7903	28x
22	*Homo sapiens*	ONT R10.4	419 GB	18125024	-	12360	80x
23	*Homo sapiens*	PacBio HiFi	166 GB	6596012	-	13478	28x
24	*Ambystoma mexicanum*	PacBio RS II	1.9 TB	105847426	-	9576	32x

(a) The datasets were simulated by PBSIM2 using the pre-trained R103 chemistry model based on the reference genomes in Table 1. The read-depth, ratio of sequencing errors (mismatches: insertions: deletions), mean length, and total error rate were configured as 50, 23:31:46, 13000, and 13% respectively. **(b)** Four datasets were simulated by PBSIM3 using the provided QSHMM-ONT-

HQ quality score model. The read-depth, ratio of sequencing errors (mismatches: insertions: deletions), mean length, and total error rate were configured as 50, 39:24:36, 15000, and 6% respectively. **(c)** Four PacBio HiFi datasets were simulated using PBSIM3 sampling-based simulation. The read length and quality score of simulated datasets are sampled from a real HiFi dataset with mean read length and accuracy of 16606 and 99.6%, respectively.

Table 2. Statistics of assembly results on real datasets.

Datasets	Tool [a]	Assembly Length (bp) [b]	#Ctgs [c]	N10 (kb) [d]	N50 (kb) [d]	N90 (kb) [d]	Genome fraction (%) [e]	#Mis-assemblies [f]	#SEs [g]	Real time [h]	Peak Memory (GB) [i]
C. elegans	xRead-nd	106383147	50	6183.59	3561.75	970.09	97.989	186	18	360.4 (s)	7.97
	xRead-wtpoa	102908753	50	7218.37	3645.98	948.68	98.893	45	11	753.8 (s)	18.93
	xRead-pipe	102454687	98	4753.84	2176.12	637.66	99.155	39	0	-	-
	NextDenovo	103679213	32	15672.21	5776.25	2136.32	99.547	76	2	7979.5 (s)	12.94
	Wtdbg2	99706610	82	10659.58	3841.09	941.37	97.711	62	11	1591.5 (s)	20.47
	Flye	102735830	57	6598.99	3310.01	1190.73	99.745	81	3	9046.0 (s)	45.57
	Shasta	99144317	66	5428.85	2831.68	950.70	97.369	38	1	611.5 (s)	40.47
	Hifiasm	-	-	-	-	-	-	-	-	-	-
D. melanogaster	xRead-nd	145917905	64	21360.15	14378.63	1523.78	89.955	598	82	719.2 (s)	12.56
	xRead-wtpoa	146420770	64	21611.07	14367.83	1508.29	90.973	393	77	1210.0 (s)	20.42
	xRead-pipe	142950657	151	15209.22	10453.23	532.13	90.979	333	1	-	-
	NextDenovo	136332796	31	27936.35	22718.49	2307.26	92.572	205	13	7444.5 (s)	19.45
	Wtdbg2	155277279	933	21514.16	7152.20	66.96	91.496	336	31	1753.0 (s)	27.92
	Flye	139561820	165	27939.08	21917.50	950.81	93.763	301	8	7841.6 (s)	50.84
	Shasta	133568381	152	27932.29	21763.69	944.65	91.181	242	6	457.5 (s)	27.07
	Hifiasm	-	-	-	-	-	-	-	-	-	-
***H. sapiens* (ONT fast mode)**	xRead-nd	2858898337	971	27779.62	6055.25	1386.12	90.401	3018	358	20.89 (h)	22.76
	xRead-wtpoa	2856254648	971	27779.99	6216.56	1554.92	92.459	686	261	24.18 (h)	37.05
	xRead-pipe	2850159937	1410	21912.39	4486.01	1155.18	92.405	372	29	-	-
	NextDenovo	2782862247	638	57559.56	25059.84	3780.30	91.614	329	108	71.89 (h)	168.75
	Wtdbg2	2729955397	4257	31781.95	11412.92	1354.65	88.623	522	400	20.12 (h)	158.35
	Flye	2847205020	3048	59028.68	21863.18	2830.99	93.101	964	177	32.39 (h)	217.85
	Shasta	2768613021	5627	4426.75	1775.63	340.55	90.738	366	34	1.45 (h)	293.24
	Hifiasm	-	-		-	-	-	-	-	-	-
***H. sapiens* (PacBio HiFi)**	xRead-nd	2972271147	2478	31619.87	12035.12	662.49	95.209	3269	253	7.16 (h)	20.61
	xRead-wtpoa	2957818291	2478	32009.50	12031.25	688.13	94.894	2975	157	9.44 (h)	22.88
	xRead-pipe	2893264635	2607	26185.02	8411.10	509.92	93.775	1583	12	-	-
	NextDenovo	2852886493	1689	77210.10	20583.16	1351.32	93.392	769	79	16.42 (h)	86.67
	Wtdbg2	2769287788	2295	44134.56	14168.42	2005.26	90.804	619	135	10.32 (h)	110.71
	Flye	2918011841	3121	59296.15	24686.38	2047.19	94.656	2678	107	25.88 (h)	151.04
	Shasta	3060152361	11261	90749.00	31895.10	878.29	97.218	3357	53	3.84 (h)	547.01
	Hifiasm	3089368991	664	139105.11	87025.29	9357.14	98.353	3693	854	6.07 (h)	93.43

(a) The assemblies were generated using eight pipelines and benchmarked using QUAST and an in-house assessment script to assess the mis-assemblies. **(b)** The total number of bases in all contigs. **(c)** The total number of contigs. **(d)** N10/N50/N90: The length of the shortest contig at 10%/50%/90% of the assembly. **(e)** The percentage of aligned bases of the reference genome. **(f)** The number

of all mis-assemblies, including both local sequence errors and structural errors. **(g)** The number of structural errors. **(h)** The overall real-time of the tools cost on simulated datasets using 30 threads, the results marked by “s” and “h” indicate CPU hours and CPU seconds, respectively. **(i)** The peak memory of the tools (in GB) on simulated datasets.

Response to the editor

Dear Prof. Liu,

Your manuscript "xRead: a coverage-guided approach for scalable construction of read overlapping graph" (GIGA-D-24-00195) has been assessed by our reviewers. Although it is potentially of interest, we are unable to consider it for publication in its current form. The reviewers have raised a number of points which we believe would improve the manuscript and may allow a substantially revised version to be published in GigaScience.

In particular, reviewer#1 raised fundamental concerns regarding the method and feels the algorithm may be overly greedy. We have discussed this point with a member of our editorial board and decided to give you a chance to revised the paper; however, if you wish to proceed with the submission to GigaScience, we insist on the points below, in addition to addressing all other comments of the two reviewers.

According to our editorial board, "the danger of the approach is the coverage-based algorithm could get confused when there are errors in repetitive sequence: these errors will reduce the read coverage, so that an overlap looks like it comes from unique sequence that is reliable to overlap; but it actually comes from a high copy repeat leading to assembly errors. So it is greedy in that as soon as it looks for a reliable overlap it quits too early; but it is hard to tell from the metrics they present how often this really occurs."

To address this,

- we insist on using CHM13 instead of GRCh38 for the baseline for simulation since this will have a complete catalog of human repeats present.
- we also insist on newer data models (13% error for ONT is too extreme).
- we also insist on comparison to newer assemblers (as Reviewer #2 suggests), and on assembly benchmarks as well as overlap-only benchmarks.
- Please also include the source code (not only compiled files) in your software repo.

If you are able to fully address these points and the other reviewers' comments, we would encourage you to submit a revised manuscript to GigaScience. Once you have made the necessary corrections, please submit online at:

https://www.editorialmanager.com/giga/

If you have forgotten your username or password please use the "Send Login Details" link to get your login information. For security reasons, your password will be reset.

Please include a point-by-point within the 'Response to Reviewers' box in the submission system. Please ensure you describe additional experiments that were carried out and include a detailed rebuttal of any criticisms or requested revisions that you disagreed with. Please also ensure that your revised manuscript conforms to the journal style, which can be found in the Instructions for Authors on the journal homepage. If the data and code has been modified in the revision process please be sure to update the public versions of this too.

The due date for submitting the revised version of your article is 14 Nov 2024.

I look forward to receiving your revised manuscript soon.

Best wishes,

Hans

Dr Hans Zauner
Editor, GigaScience
www.gigasciencejournal.com

--Response:

Thanks very much for the handling of our manuscript. We appreciate the editor's and the reviewers' comments and suggestions and have addressed all the raised points.

To address the concerns raised by the editorial board and Reviewer #1 about the "greedy strategy" of xRead and its effect on repeat handling, we largely re-write "Overview of the xRead approach" and "Discussion". In the current version, these sections precisely describe the theoretical designs and features of xRead and thoroughly explain its applicability to major strategies of long read-based assembly as well as its potential equivalence to the graphs produced by other state-of-the-art read overlapping tools.

Following the suggestions of the editorial board member, we have simulated new datasets from the T2T-CHM13v2.0 reference genome and added new benchmarks, the results are in Fig.2 and Supplementary Tables 3-4. Moreover, we have provided the results on ONT-like datasets with higher quality (6% error rate, simulated by PBSIM) in the previous version of the manuscript, but could not describe them clearly. To improve the readability, we added a new section "The datasets used in benchmark" and a table (Table 1) to more clearly describe the detailed information of the datasets used in the benchmark.

To more thoroughly assess the use of xRead to genome assembly, we build a whole genome assembly pipeline (termed as xRead-pipe) based on the overlapping graphs produced by xRead. This is non-trivial since most of the state-of-the-art assemblers have various heuristics in their modules and do not allow a third-party plug-in (this is also mentioned by Reviewer #2). We tried our best to build the pipeline by borrowing the layout and consensus modules from NextDenovo and Wtdbg2 as they are open-source and more integration-friendly. We also used a post-processing script developed in one of the previous T2T genome assembly studies to reduce the incompatibility of the modules. We benchmarked xRead-pipe by 16 datasets (12 simulated plus 4 real) from various-sized genomes and compared the results to that of five state-of-the-art assemblers including NextDenovo, Flye, Wtdbg2, Shasta and Hifiasm. Overall, xRead-pipe showed higher performance and comparable assembly quality (i.e., completeness, correctness and continuity) to that of the state-of-the-art assemblers. Moreover, we also realized from the behaviors of xRead-pipe that this straightforwardly integrated pipeline is still far from well-tailored to release all the potentials of xRead. We give a detailed discussion about this fact (in Discussion section), furthermore, bring the key points of our future work (which has been ongoing) to extend xRead to a full genome assembler at the end of the manuscript.

A point-to-point response is as follows.

- According to our editorial board, "the danger of the approach is the coverage-based algorithm could get confused when there are errors in repetitive sequence: these errors will reduce the read coverage, so that an overlap looks like it comes from unique sequence that is reliable to overlap; but it actually comes from a high copy repeat leading to assembly errors. So it is greedy in that as soon as it looks for a reliable overlap it quits too early; but it is hard to tell from the metrics they present how often this really occurs."

--Response:

Thanks for the comments from editorial board members. We agree that xRead could make false positive overlaps for the reads from repetitive regions. However, we also believe that the xRead approach is not over-greedy and these mistakes may be not caused by its coverage-guided iterative processing. In fact, the graph produced by xRead is to some extent equivalent to the ones produced by other state-of-the-art read overlapping tools. They also meet very similar problems of handling repeats, moreover, this issue can be addressed in downstream steps of assembly. A more precise explanation is as follows.

First of all, the graph produced by xRead is seed-read centered. It is also possible to convert the graph to other styles, such as the best (restricted to the best overlaps) or all significant (containing all the significant overlaps) overlapping graphs produced by other tools. This is in essence supported by the high precision and connectivity of xRead that the potential overlaps can be confidently inferred from the connected components of the graph by transitive relationships. In this situation, it is hard to say that xRead is an over-greedy approach, although with the "mimicking-and-mapping" design it seems simply converting read overlapping to a read mapping-like task. A more detailed description about the design of xRead approach has been added in overview subsection ("Overview of the xRead approach", Findings section), moreover, a detailed discussion about its features has also been supplied in Discussion section, please also refer to it (or see below).

It is a common problem for read-overlapping tools that false positive overlaps could be produced for the reads from repetitive regions, since they are highly similar and cannot be confidently distinguished by the alignment (or other measures of read similarity). This problem also happens to xRead. That is, for the reads from various repeat copies, xRead could align all the seed-reads together and randomly map other non-seed-reads to some of the seed-reads depending on the scores of the alignment skeletons, thus some false positive overlaps are produced. Such errors are apparently caused by the intrinsic sequence similarity, but not the coverage-guided heuristics of xRead. Moreover, the consequence is quite similar to that of other overlapping tools, i.e., the repetitive reads form a connected component and the produced graph collapses for the multiple copies of repeats (also refer to Supplementary Figure 8 for schematic illustrations). Such a case is frequently met in the layout phase of assembly. And this issue can also be addressed since the reads are well-connected and easy to cluster via the connected component. As many advanced read correction, re-clustering and heuristic layout methods have been proposed, many of them can be potential solutions to the graph of xRead, like what they did for the graphs produced by other tools.

It is also worthnoting that, in this situation, the derived coverage for a repetitive read would be not reduced, but increased, since with the graph topology more reads can directly or indirectly connect to it than that of non-repeat reads. We also guess that the comment of the editorial board member could point to the case that xRead selects a read from a repetitive region as seed-read, meanwhile, no read from other copies is selected. For this case, all the reads from other copies would be aligned to the seed-read. And then the consequence is also similar, i.e., all the reads from the various copies are still concentrated in the same connected component, moreover, each of the reads can infer its best or all the significant overlaps from the transitive relationships implied by the component (as mentioned above). In this situation, we believe that xRead does not quit too early during read overlapping.

Although cannot be fully prevented, xRead also makes its own efforts to reduce the effects of such errors by two means. One is the CRO-based alignment skeletons which restricts highly confident overlaps and reduces the chance of mapping a read to a wrong copy. Another is that xRead outputs the inferred coverage for each of the reads (in its PAF format output, CV FLAG). This is a good repeat-indicating marker since the repetitive reads have obviously higher coverages, moreover, they can be conveniently focused in later steps.

Furthermore, in the current version of the manuscript, we also implemented a thorough benchmark to assess the effect of xRead on genome assembly. The results demonstrate that the ability of the xRead-based assembly pipeline is close to that of other state-of-the-art assemblers (with higher performance). This also indicates that the false positives do not affect too much to the whole procedure and the overall quality of genome assembly. Also see below (the response for the third issue raised by the editorial board) for more detailed information about the benchmark.

For the description on the design of xRead, we have mentioned in the current version of the manuscript:

"Unlike state-of-the-art overlapping tools that in essence push each of the reads to explore all the other ones with heuristics, xRead adopts a "mimicking-and-mapping" design. Mainly, it is motivated by that each read can be seen as a representative of some part of the donor genome. A selected set of seed-reads covering the whole genome can mimic a "virtual reference" to reveal read-overlaps through a read-to-reference mapping, i.e., the reads from the same region can be implicitly aligned to the corresponding seed-read(s) in the virtual reference. So, all the overlaps between seed and non-seed-reads can be detected and the various parts of the genome can also be connected by such alignments. Guided by a novel coverage-based strategy of seed-read selection ("the completeness of virtual reference", see below), xRead constructs the overlapping graph in an iterative process (Fig. 1)." (line 18, page 4 of main text)

"This approach lays a new foundation for genome assembly in two aspects. Firstly, it is apparent that the cost can be substantially reduced by converting all-against-all read alignment to a read-to-reference mapping task. Secondly, the produced graph can also be correctly connected, i.e., achieve high precision and connectivity simultaneously, which is supportive to both of the popular strategies in long read assembly (i.e., correction-then-assembly and assembly-then-correction, also refer to Discussion section for a more detailed explanation). These are done by some tailored implementations for several critical issues as follows.

1) The completeness of virtual reference. To achieve the best performance, it is fundamental to select a set of seed-reads covering the whole genome and having as few as possible redundant reads, but non-trivial. xRead achieves this goal by using a fact of the read mapping. That is, given an arbitrary set of seed-reads, relatively high coverage (i.e., times of being aligned) can be derived for all the reads belonging to the virtual-reference covered regions, which is done by exploring the produced alignments and their transitive relationships. Meanwhile, the reads from uncovered regions have much lower coverage. Thus, the coverage becomes a useful indicator to iteratively select the reads from those still uncovered regions and the whole genome can be implicitly and progressively explored. This assumption could be over-simplified for the reads from ultralong repeats, however, it does not affect the overlapping task

much (refer to Discussion section for more details).

2) The mappability of virtual reference. A high mappability of virtual reference (seed-reads) is useful to achieve confident read mapping. Thanks to the randomness and less bias of long-read sequencing [42-44], there is no obvious correlation between the read lengths and positions. Thus, with the arbitrary selection assumption mentioned above, it is feasible to straightforwardly select the longest ones from the remaining reads to maximize the mappability of the virtual reference.

3) The correctness of read overlapping. xRead is more precision-oriented in the precision-sensitivity tradeoff during overlapping detection. The virtual reference helps to reduce false positive overlaps systematically with its high mappability. Moreover, xRead also employs a conservative alignment-scoring approach to construct an accurate overlapping graph. Following this strategy, xRead only keeps the most confident read overlaps (CROs, see Methods) with high alignment scores. Motivated by that successful assembly may come from a correctly connected graph, the high precision design of xRead is useful, especially with good graph connectivity (see below).

4) The connectivity of the produced graph. Although not implementing a comprehensive overlap directly, xRead also produces highly connected graphs. The key point is the completeness of virtual reference. From a donor genome point of view, the seed-reads are ubiquitously placed and no large gap exists between two nearby seed-reads. Thus, each non-seed-read can be aligned (i.e., connected) to one or more seed-reads, and then the non-seed-reads aligned to the same seed-read compose a connected component. Further, these components can be connected comprehensively since any pair of nearby seed-reads can be either directly aligned (if they are overlapped) or connected via one or more non-seed-reads having overlaps to both of them.

5) The optimization of performance. xRead optimizes speed and RAM usage by several tailored implementations to achieve outstanding performance and scalability. One is the use of the lightweight alignment skeleton (Step 2) to speedup each iteration, referring to previous studies [45-47]. Another is that, followed by the coverage-guided overlapping strategy, the batch size of the seed-reads is highly tunable so that the whole process can be run with controllable RAM usage." (line 3, page 5 of main text)

For the discussion on the produced graph and repeat handling of xRead, we have mentioned in the current version of the manuscript as follows:

"xRead has the potential to convert its produced graph to other styles, such as restricted to the best- or containing all the significant overlaps, which are popularly used strategies by other overlapping tools. This is done by using transitive relationships. It is not difficult for a seed-read to collect all the reads overlapping with it since such reads are either directly aligned to it, or aligned to another seed-read in the same connected component. For the latter case, the assumption also stands due to both of the high connectivity (i.e., seed-reads are well-connected as mentioned) and precision (i.e., in most cases all the transitive edges/overlaps are true positive) of the graph. Thus, the overlaps to the seed-read can be thoroughly evaluated to find out the best or all the significant ones. It is similar to a non-seed-read, i.e., the read is initially aligned to a seed-read and then involved in a connected component. Further, the overlaps to it can be inferred and evaluated through all the overlaps implied by the component. This feature

is partially demonstrated by simulation and real benchmarks (Supplementary Tables 5 and 9), also refer to Supplementary Figure 8A for schematic illustrations." (line 20, page 17 of main text)

"xRead still has some limitations to handle the reads from ultralong repeats which is also a common problem to all read overlapping tools. As the lengths are not enough to span such repeats, the reads from various copies are not distinguishable with the crosstalk of their intrinsic similarity and sequencing noise. Under such circumstances, xRead could align the seed-reads from various copies together and map non-seed-reads to some of them randomly. Then all the reads are connected as one component and the graph collapses, which is also a common case during read layout (refer to Supplementary Figure 8B for schematic illustrations). A feasible solution is to use tailored approaches to precisely correct sequencing errors and re-cluster the reads. With the improved base quality, the reads would have a good chance to be distinguished and the graph can be corrected. xRead also makes its own efforts to reduce this effect by two means. One is the CRO-based alignment skeletons which restricts highly confident overlaps and reduces the chance of mapping a read to a wrong copy. Another is that xRead outputs the inferred coverage for each of the reads (in its PAF format output, CV FLAG). This is a good repeat-indicating marker since the repetitive reads have obviously higher coverage, furthermore, they can be conveniently focused in later steps." (line 32, page 17 of main text)

- we insist on using CHM13 instead of GRCh38 for the baseline for simulation since this will have a complete catalog of human repeats present.

--Response:

We have followed this suggestion. In the previous version of the manuscript, we simulated a low-quality ONT-like dataset (87%) using the GRCh38 reference genome in the benchmark, which was done at the beginning of the development of xRead. However, since the publication of T2T-CHM13v2.0, we have used it to simulate the high-quality ONT-like (94%) and HiFi-like datasets. We are sorry for not updating the early-used low-quality ONT datasets, as well as the inconsistency in the information of reference genome.

In the current version of the manuscript, we re-simulated the low-quality ONT-like dataset (87%) using PBSIM with the T2T-CHM13v2.0 reference genome, which was previously generated based on GRCh38. We also revised Supplementary Table 1 to update the information on reference genomes used in our benchmarks. Furthermore, we confirmed that all the simulated human genome datasets used in the overlapping and assembly benchmarks are based on T2T-CHM13v2.0 now.

The results of the low-quality ONT-like dataset (87%) have also been updated in the corresponding figure (Fig. 2) and tables (Supplementary Table 3-5). Highly similar trends were observed from the new results, i.e., the graphs produced by xRead still have much fewer false positive overlaps (i.e., high precision) with good connectivity. Meanwhile, xRead also achieved relatively high speed (approximately three times faster than other tools) with quite low memory footprint (5%-55% compared to that of other tools). It is also worthnoting that the numbers of connected components (Con. Num. statistics) of the graphs produced by xRead, MECAT2 and wtdbg2 were significantly reduced, indicating the improvements on graph connectivity. We further checked the detailed information and found that this derives from the gapless assembly

and better repeat representation of CHM13, i.e., the simulated reads are natively more connectable, compared to that of GRCh38.

- we also insist on newer data models (13% error for ONT is too extreme).

--Response:

We agree with this suggestion that newer data models are more suited to be used in the benchmark. In the previous version of the manuscript, we used a low-quality (13% error rate) ONT data model to assess the ability of xRead to handle the noisy long reads from various-sized genomes.

We also simulated high-quality ONT-like datasets (6% error rate) and employed them in both the overlap and assembly benchmarks. These datasets were simulated from four genomes (*E. coli*, *A. thaliana*, *D. melanogaster* and *H. sapiens*), using the QSHMM-ONT-HQ data model provided by PBSIM. Their mean read length and average accuracy were about 15 kbp and 94%, respectively.

We have realized the unclear description of the detailed information about the employed datasets, which is also hinted by Reviewer #2. Therefore, we add a new subsection "The datasets used in benchmark" to clarify the datasets used in overlap and assembly benchmarks. Moreover, a new table (Table 1) is also added to describe the detailed information of the datasets to further improve the readability.

We have revised the manuscript as follows:

"We simulated 17 datasets by PBSIM [48, 49] and also employed 7 real datasets (Table 1) for the benchmark of read overlapping and genome assembly. The datasets are from nine genomes (Supplementary Table 1) having small- (<1Gbp, i.e., *E. coli, S. cerevisiae, C. elegans, A. thaliana* and *D. melanogaster*), large- (>1Gbp, i.e., *Z. mays, M. musculus* and *H. sapiens*) and very large- (>10Gbp, i.e., *A. mexicanum*) sizes. It is also worthnoting that we partitioned the chromosomes of the A. mexicanum genome in the simulation due to the limit of PBSIM (15 of the chromosomes were divided into 30 <1Gbp ones in advance).

To assess the performance of xRead in various error rates, we respectively simulated nine low-quality, four high-quality ONT-like and four HiFi-like datasets (50x coverage each) as follows.

1) The nine low-quality ONT datasets were simulated for all nine genomes with the pre-trained R103 chemistry model of PBSIM. The mean read length and total error rate were 13 kbp and 13%, respectively, referring to a previous study [3]. These datasets are highly noisy like the fast base-calling mode of ONT platforms and employed to assess the robustness of xRead to sequencing errors for various-sized genomes.

2) The four high-quality ONT datasets were also simulated from four genomes (*E. coli, A. thaliana, D. melanogaster* and *H. sapiens*). The QSHMM-ONT-HQ model was used (the mean read length and average accuracy were 15 kbp and 94%, respectively) to mimic the data produced by mostly used ONT platforms (especially for its HAC mode).

3) The four HiFi-like datasets were produced by sample-based simulation of PBSIM with a real sequencing dataset (mean read length: 16.6 kbp and average base accuracy: 99.6%) from four genomes (*E. coli, A. thaliana, D. melanogaster* and *H. sapiens*). These datasets mimic the data produced by currently used PacBio platforms." (line 13, page 6 of main text)

- we also insist on comparison to newer assemblers (as Reviewer #2 suggests), and on assembly benchmarks as well as overlap-only benchmarks.

--Response:

We fully agreed with the editorial board member and the reviewers that it is important to evaluate the effect of xRead on genome assembly and compare it to newer assemblers. In the current version, we implemented a more thorough genome assembly benchmark. Mainly, we used in total 16 datasets (12 simulated plus 4 real) having various sequencing qualities. In detail, the 12 simulated datasets are in three error models (low-quality ONT, high-quality ONT, and PacBio HiFi) from four genomes (*E. coli, A. thaliana, D. melanogaster* and *H. sapiens*). For the 4 real datasets, 3 of them are produced by ONT platforms from *C. elegans, D. melanogaster,* and *H. sapiens*, respectively, and the other one is also a human dataset but produced by PacBio Hifi sequencing. Five state-of-the-art assemblers (NextDenovo, Flye, Wtdbg2, Shasta and Hifiasm) were employed for comparison.

We used xRead as the overlapping module and built an in-house assembly pipeline (termed xRead-pipe). This is non-trivial since most state-of-the-art assemblers do not allow third-party plug-ins. We tried our best to integrate the layout and consensus modules of NextDenovo and Wtdbg2 (NextGraph and wtpoa-cns, respectively) to achieve this goal as they are open-source and more integration-friendly. Furthermore, we also used an in-house assembly post-processing script developed in one of our previous studies to reduce assembly errors caused by the incompatibility of various modules. All the assemblers were implemented on the simulated and real datasets, please refer to Table 2, Supplementary Tables 11-12 and Supplementary Figures 2-7 for detailed results. Moreover, we also have added a new subsection ("The use of xRead for de novo assembly") in Findings section to describe the results of whole genome assembly with xRead and also give detailed discussions and explanations on critical issues.

The results indicate that, overall, xRead-pipe is able to achieve comparable completeness, correctness and contiguity to that of state-of-the-art assembles and its performance is obviously higher. Mainly, three key points were observed.

1) Compared to other assemblers, xRead-pipe significantly reduced the time cost and memory usage of genome assembly which indicates outstanding performance and scalability.

2) xRead-pipe achieved similar assembly completeness (assembly length and genome fraction) to that of other assemblers while it had the lowest numbers of structural errors, indicating the high correctness of the assembly.

3) The continuity (N10/N50/N90 statistics) of xRead-pipe was lower (but comparable) to some of the other assemblers. Moreover, it is also observed that the assemblers producing longer contigs usually had more misjoin errors, which indicates a tradeoff between correctness and continuity by various assembly strategies.

We carefully tracked the behaviors of xRead-pipe and found that, the pipeline still has not released all the potentials of xRead through this straightforward and forced integration of various modules yet, although it has achieved considerable quality and performance. This is mainly due to the following two issues caused by the incompatibility of NextGraph.

1) The read correction module of NextDenovo is not compatible with xRead. Thus, NextGraph is forced to directly process the graph of xRead without the critical read correction step and its ability is suppressed.

2) NextGraph handles the graph by its own heuristics and does not fully consider the characteristics of xRead, such as the higher importance of the seed-reads to graph topology. Thus, some of the critical edges were wrongly handled which led to misjoins and gaps.

Such incompatibility affected both the correctness and continuity of assembly. With the consensus and post-processing modules, xRead-pipe corrected a large proportion of the misassemblies, however, a more tailored approach is still needed. We added detailed discussions about this issue in Discussion section. Further, at the end of the manuscript, we brought the key points to our future works by summing up all the results and analysis. The work has been ongoing to develop xRead into a well-tailored and more advanced genome assembly tool under the guidance of the key points.

For the implementation of assembly benchmark, we have mentioned in the current version of the manuscript ("The use of xRead for de novo assembly", Findings section) as follows:

"The effect of xRead on genome assembly was further assessed. We tried to integrate xRead into a couple of state-of-the-art assemblers, such as NextDenovo [29], Wtdbg2 [17], Hifiasm [32], Shasta [18], Flye [25] and Canu [15]. This is non-trivial since for most of them the various modules (such as overlapping, layout and consensus) are coupled with specific in-memory data structures (also lack of published details) which do not allow flexible plug-ins. Under such circumstances, we made efforts to build an in-house assembly pipeline that uses the xRead graph as input and borrows two open-source modules from other assemblers, i.e., the layout module of NextDenovo (NextGraph) and the consensus module of Wtdbg2 (wtpoa-cns). Further, an in-house post-processing script developed in one of our previous T2T-assembly studies [50] was used to reduce the structural mis-assemblies caused by the incompatibility of the modules. In the benchmark, we assessed the final assemblies (termed as xRead-pipe) as well as the intermediate outputs through NextGraph and wtpoa-cns modules (termed as xRead-nd and xRead-wtpoa respectively).

Twelve simulated datasets (Table 1) from four genomes (*E. coli, A. thaliana, D. melanogaster* and *H. sapiens*) in three error models (low-quality ONT, high-quality ONT and PacBio HiFi sequencing) were used for benchmark. Moreover, three ONT real datasets (*C. elegans, D. melanogaster* and *H. sapiens*) and a human HiFi dataset (Table 1) were also employed. We tried to implement six pipelines (i.e., xRead-pipe, NextDenovo, Wtdbg2, Flye, Shasta and Hifiasm) on all the 12 (simulated) + 4 (real) datasets for comparison. Since Shasta is not suited to low-quality ONT datasets and Hifiasm is specifically designed for HiFi data, their corresponding results were excluded. QUAST [42] (version 5.2.0) was employed to evaluate the assemblies by the following metrics: assembly length, the number of contigs, N10/N50/N90 statistics, genome fraction, the number of all- and structural mis-assemblies. It is worthnoting that we distinguished the assembly errors by local sequence errors (small-scale sequence expansions and collapses) or structural errors (large-scale misjoins) since the latter are usually much more difficult to resolve and should be more focused." (line 27, page 13 of main text)

For the results of assembly benchmark, we have mentioned in the current version of the manuscript ("The use of xRead for de novo assembly", Findings section) as follows:

"The results are in Table 2 (real) and Supplementary Tables 11-12 (simulated), respectively. It indicates that the completeness, correctness and contiguity of xRead-pipe are

close to that of state-of-the-art assemblers, while its performance is obviously higher. Some intermediate results also suggested that some features of xRead were also not being fully utilized by this straightforward integration and it also has the potential to further improve assembly quality by more tailored layout and consensus modules. Mainly, three issues were observed as follows.

1) xRead-pipe reduces the overall computational cost of genome assembly.

For nearly all the datasets, both the runtime and memory usage of xRead-pipe is lower than most of the other assemblers (Table 2 and Supplementary Table 11, Real time and Peak Memory columns), suggesting that xRead is beneficial to the overall performance of assembly. Especially, xRead-pipe outperforms the original NextDenovo and Wtdbg2 pipelines by on average 2.5 times speedup and 4.2 times lower memory footprints. It is also worthnoting that Shasta is the fastest one on human datasets while it also has the highest memory footprints, indicating a tradeoff between time- and memory cost. One example is that Shasta has a 776 GB peak memory on the simulated human HiFi dataset (17 and 37 GB for xRead-nd and xRead-wtpoa, respectively) which is a non-neglectable requirement for computational resource and could affect its scalability due to hardware limitation.

2) xRead-pipe is able to produce correct assemblies.

The QUAST evaluation (Table 2, Supplementary Table 12) indicates that overall xRead-pipe made comparable or lower numbers of mistakes than that of other pipelines, with quite similar assembly completeness (assembly lengths and genome fractions). This suggests that, using xRead graph, the assembly quality of the pipeline can reach the same level of state-of-the-art tools. We further investigated the types of mis-assemblies and found that, for all the assemblers, most of the mis-assemblies were local sequence errors and the numbers of structural errors were much lower. Especially, the assemblies of xRead-pipe had the lowest numbers of structural errors among all the assemblers. This should be praised since the small errors are easier to fix by advanced error correction and consensus sequence generation approaches. However, nearly all the large misjoins are related to complex repeats and are non-trivial to deal with.

The results of xRead-nd, xRead-wtpoa and xRead-pipe showed decreasing numbers of mis-assemblies, indicating the improvement on correctness by various modules. We further tracked their behaviors and found that a primary cause of the errors was the incompatibility of the layout module. That is, NextDenovo is in a correction-then-assembly design that takes advantage of a tailored correction module to reduce sequencing errors and re-cluster reads in advance. This is critical to read layout, however, it does not allow xRead graphs as input. Under such circumstances, the forced layout module (NextGraph) produced more errors. Further, a large proportion of the errors were amended by wtpoa and the in-house correction script. Wtpoa mainly improved the quality of consensus sequence and reduced a number of sequence errors, especially the ones caused by ONT sequencing noise. It also rescued a proportion of structural errors. Further, by contig realignment (refer to Methods section), the in-house script more effectively detected misjoin regions and largely eliminated the structural errors. With the two modules, xRead-pipe is able to catch up or outperform the original NextDenovo pipeline (and other assemblers as well) at assembly correctness.

There are still errors caused by the crosstalk of xRead and NextGraph that cannot be eliminated. One is due to the very long repeats such as segmental duplications that the read

length is still not enough to solve them thoroughly. Other assemblers also have such problems as well. On the other hand, it is also non-neglectable that NextGraph may not fully take advantage of xRead. In the xRead graphs, seed-reads are critical to connect the various parts of the donor genome. They should be given higher weights and used as the backbone during assembly. However, NextGraph does not have such a weight-tuning function. It implemented graph simplification by iteratively resolving nonlinear structures using some heuristics based on read overlap lengths, identities and depths. With its own heuristics, some of the overlaps between seed-reads were mistakenly discarded and leaving structural errors. A typical case is the removal of the z-clip structure (an example is shown in Supplementary Figure 2), i.e., due to the lack of adaptive weighting, some critical edges in nonlinear structures like z-clip structures were mistakenly removed and resulted in the misjoins between non-successive genomic parts.

3) xRead-pipe has comparable continuity to state-of-the-art approaches.

The results (Table 2 and Supplementary Table 12, N10/N50/N90 columns) suggest that the continuity of xRead-pipe is comparable to or slightly lower than that of other assemblers. We investigated the intermediate results of xRead-pipe and found that the decreased continuity is caused by the removal of some critical edges by NextGraph which made assembly gaps. For most of the assembly gaps in the results of xRead-pipe, the successive genome parts were initially connected in the graphs of xRead. The primary cause is still the heuristics of NextGraph to handle the z-clip structures. As mentioned above, the seed-reads in z-clip structures should serve as the backbone of the xRead graph. However, NextGraph selected the branches by its own rules and did not consider the importance of seed-reads, thus mistakes occurred (an example is in Supplementary Figure 3, and a more complicated one is in Supplementary Figure 4). Therefore, to further improve assembly continuity, a tailored layout method should be developed for xRead to better use the topology as well as other evidence provided by the graph.

Moreover, it is also non-neglectable that a tradeoff can be observed between the continuity and correctness of assembly. That is, the assemblers producing longer contigs also made higher numbers of structural errors. Such errors have no correlation with the lengths of contigs (Supplementary Figure 5), but are related to genomic contexts such as repeats, indicating that they could be partially caused by some relatively aggressive repeat-handling strategies of the assemblers. We realigned the error-containing contigs and investigated the mapping positions of their various parts. With the annotation of RepeatMasker, we found that the errors mostly happened around those well-known repetitive elements such as SINE, LINE, simple tandem repeats and centromeric repeat patterns.

It is worthnoting that the errors may also occur in very long contigs which are usually produced with high confidence. An example is in Supplementary Figure 6. This is a contig of Flye whose length is 50.0 Mb, close to its N10 statistics. A clipping was detected around contig_2932: 42,085,000 which indicates a translocation. The contig was then split into two segments (42.1 Mb and 6.9 Mb, respectively) which can be aligned to reference at the positions Chr3: 88,844,116 and Chr6: 19,746,930, respectively. Another example is in Supplementary Figure 7. This is a contig of NextDenovo whose length is 43.5 Mb, also close to its N10 statistics. A clipping event was observed at ctg003710: 12,544,314, indicating a translocation error in the contig. The contig then can be split aligned to reference at two positions, i.e., Chr3: 91,045,032 and Chr6: 57,667,858.” (line 17, page 14 of main text)

For the key points of our future work, we have mentioned in the current version of the manuscript:

“The use of xRead to whole genome assembly was also evaluated although it is hard to integrate a standalone overlapping tool to the state-of-the-art assemblers since most of their modules are coupled by various heuristics and do not allow third-party plug-ins. We tried our best to build a pipeline by borrowing the layout and consensus modules from NextDenovo and Wtdbg2, respectively. Considering both the time cost and RAM usage, the pipeline showed higher performance and scalability, meanwhile, it also achieved the same order of completeness, correctness and contiguity as that of those state-of-the-art assemblers. The results suggest that xRead is promising to scalable assembly. Further, we also would like to claim that the ability of xRead is not fully exerted with the straightforward (to some extent rough) integration. For example, due to the lack of a readily made module, this pipeline can still not fully take advantage of precise read correction (which is critical to repeat-handling). Moreover, existing layout methods also could not fully consider the characteristics of the graphs produced by xRead. It is an important future work (has been ongoing) to develop novel tools based on xRead graph to achieve high-quality, efficient and scalable genome assembly. We realize that there could be three key points to the development as follows.

Firstly, it is critical to develop a tailored read correction method for xRead. The seed-reads can be directly used as anchors to cluster the reads from the same genomic regions. Thus, read correction can be straightforwardly implemented by similar approaches to that of state-of-the-art assemblers, such as multiple sequence alignment [52], pseudo variant calling [53], and sequence graph analysis [14]. Moreover, it also needs to develop novel methods to use the correction information to distinguish and re-cluster the reads from various repeat copies to refine the graph.

Secondly, it also needs to design a specific read layout method with xRead graph. That is, various weights should be added to the edges and carefully considered since the connectivity of the CRO graph depends on the edges and short-paths between seed-reads and they should be well-handled. It is feasible to build an essential backbone by seed-reads as they are well connected and implicitly distributed along the whole genome. Moreover, non-seed-reads can be used as extra evidence to rescue unconnected contigs, prevent mis-assemblies, and support the consensus phase.

Thirdly, it is still an open problem to implement efficient haplotype assembly. For xRead, one feasible way is to expand the graph dynamically with the transitive relationships during layout and precisely analyze the alignments among reads to adaptively reconstruct haplotype-specific paths. Another one is to use the initially assembled genome as reference to implement realignment, variant calling, and phasing, i.e., achieve haplotype assembly in a “de novo- and re-sequencing” way. This approach could be more suited to tasks having various types of reads (such as many T2T-assembly tasks) since the resequencing-style post-processing is more convenient to integrate the data and use various kinds of tools to correct mis-assemblies and construct haplotypes. Moreover, it is also feasible to keep low cost all the way, and even more, simultaneously handle many genomes in a step-parallel approach to achieve very high overall performance in large-scale genomics studies.” (line 12, page 18 of main text)

- Please also include the source code (not only compiled files) in your software repo.

--Response:

Thanks for pointing this out. We checked and updated the source code URL on GitHub, as well as the "Instruction" section of README.md. We also verified the availability of all URLs. All of them have been ensured to be correct and available now.

The new URL including the source code is as follows:

https://github.com/tcKong47/xRead/releases/download/v1.0.0/xRead-v1.0.0.tar.gz

The updated compiling instructions are as follows:

```
wget https://github.com/tcKong47/xRead/releases/download/v1.0.0/xRead-v1.0.0.tar.gz
tar -zxvf xRead-v1.0.0.tar.gz
cd xRead-v1.0.0; make
```

Response to reviewer #1

Reviewer #1: The manuscript describes xreads, a novel method that enables resource-efficient overlap graph computation based on new strategies to compute it quickly and with controlled memory usage. The authors introduce several quality metrics to assess the quality of the overlap graph and integrate their tool into NextDenovo, improving its resource usage.

--Response:

Thanks for the comments and suggestions of the reviewer. We have supplied new benchmark results and revised the manuscript according to the reviewer's comments. A point-by-point response has been made according to the comments as follows.

The manuscript is overall clear, although the section order can make it hard to read as concepts are defined backward. Some typos and minor phrasing issues should be corrected.

--Response:

We agree with the reviewer's comments. We have checked all proposed concepts and linguistic issues throughout the entire manuscript, and also have adjusted the positions of some concept definitions. Moreover, for those paragraphs not suited to re-position, we have added references to give kindly guidance to the reader.

We realized that the motivations, concepts and designs of the xRead approach are not clearly described at the beginning of the manuscript which does affect the readability. In the current version of the manuscript, we largely re-write the subsection "Overview of the xRead approach" to precisely describe the design of xRead and more thoroughly explain its features to help the readers to more easily understand the approach.

Some important concepts are also given in advance. For example, the Confident Read Overlaps (CROs) strategy is a concept frequently used in Findings section but defined later in Methods section. In the current version, we introduce the CRO strategy in the "Overview of the xRead approach" section, which let it appear at the beginning of Findings section. Moreover, some important definitions for the assessment, such as ground truth, true positive and false positive overlaps, as well as the computation of precision and sensitivity, are given in an independent subsection of Methods (titled "Assessment of the sensitivity and precision in produced graph"). We realized that this subsection could be not very suited to move forward, so that we added references when using them in Findings section to guide readers to the corresponding detailed information.

We have revised the manuscript as follows:

"Moreover, xRead also employs a conservative alignment-scoring approach to construct an accurate overlapping graph. Following this strategy, xRead only keeps the most confident read overlaps (CROs, see Methods) with high alignment scores." (line 27, page 5 of main text)

"We evaluated the overall precisions and sensitivities of the tools (Fig. 2b and Supplementary Table 4, Precision and Sensitivity columns, also refer to Methods section for more details about assessment, including the definitions of ground truth, true positive and false positive overlaps, as well as the computation of precision and sensitivity)." (line 24, page 8 of main text)

We also corrected the typos and writings mistakes. Some representative examples are listed below. We have revised the manuscript as follows:

"Step 1: xRead selects a proportion of reads with relatively low coverage and high length as seed-reads and builds a partial read index for them." (line 29, page 4 of main text)

"Therefore, it also holds the possibility of comprehensive graphs with post-processing." (line 12, page 9 of main text)

"R% is the proportion of the reads belonging to the block that has at least one ground truth overlap recalled." (line 26, page 10 of main text)

"Partial evidence is that all the tools have obviously lower numbers of connected components on the human PacBio HiFi dataset than that of the ONT fast mode dataset." (line 28, page 12 of main text)

"Further, xRead computes the average of the window coverage as the estimated coverage for a read." (line 5, page 21 of main text)

For a thorough explanation of the xRead's designs, we have mentioned in the current version of the manuscript ("Overview of the xRead approach", Findings) as follows:

"Unlike state-of-the-art overlapping tools that in essence push each of the reads to explore all the other ones with heuristics, xRead adopts a "mimicking-and-mapping" design. Mainly, it is motivated by that each read can be seen as a representative of some part of the donor genome. A selected set of seed-reads covering the whole genome can mimic a "virtual reference" to reveal read-overlaps through a read-to-reference mapping, i.e., the reads from the same region can be implicitly aligned to the corresponding seed-read(s) in the virtual reference. So, all the overlaps between seed and non-seed-reads can be detected and the various parts of the genome can also be connected by such alignments. Guided by a novel coverage-based strategy of seed-read selection ("the completeness of virtual reference", see below), xRead constructs the overlapping graph in an iterative process (Fig. 1)." (line 18, page 4 of main text)

"This approach lays a new foundation for genome assembly in two aspects. Firstly, it is apparent that the cost can be substantially reduced by converting all-against-all read alignment to a read-to-reference mapping task. Secondly, the produced graph can also be correctly connected, i.e., achieve high precision and connectivity simultaneously, which is supportive to both of the popular strategies in long read assembly (i.e., correction-then-assembly and assembly-then-correction, also refer to Discussion section for a more detailed explanation). These are done by some tailored implementations for several critical issues as follows.

1) The completeness of virtual reference. To achieve the best performance, it is fundamental to select a set of seed-reads covering the whole genome and having as few as possible redundant reads, but non-trivial. xRead achieves this goal by using a fact of the read mapping. That is, given an arbitrary set of seed-reads, relatively high coverage (i.e., times of being aligned) can be derived for all the reads belonging to the virtual-reference covered regions, which is done by exploring the produced alignments and their transitive relationships. Meanwhile, the reads from uncovered regions have much lower coverage. Thus, the coverage becomes a useful indicator to iteratively select the reads from those still uncovered regions and the whole genome can be implicitly and progressively explored. This assumption could be over-

simplified for the reads from ultralong repeats, however, it does not affect the overlapping task much (refer to Discussion section for more details).

2) The mappability of virtual reference. A high mappability of virtual reference (seed-reads) is useful to achieve confident read mapping. Thanks to the randomness and less bias of long-read sequencing [42-44], there is no obvious correlation between the read lengths and positions. Thus, with the arbitrary selection assumption mentioned above, it is feasible to straightforwardly select the longest ones from the remaining reads to maximize the mappability of the virtual reference.

3) The correctness of read overlapping. xRead is more precision-oriented in the precision-sensitivity tradeoff during overlapping detection. The virtual reference helps to reduce false positive overlaps systematically with its high mappability. Moreover, xRead also employs a conservative alignment-scoring approach to construct an accurate overlapping graph. Following this strategy, xRead only keeps the most confident read overlaps (CROs, see Methods) with high alignment scores. Motivated by that successful assembly may come from a correctly connected graph, the high precision design of xRead is useful, especially with good graph connectivity (see below).

4) The connectivity of the produced graph. Although not implementing a comprehensive overlap directly, xRead also produces highly connected graphs. The key point is the completeness of virtual reference. From a donor genome point of view, the seed-reads are ubiquitously placed and no large gap exists between two nearby seed-reads. Thus, each non-seed-read can be aligned (i.e., connected) to one or more seed-reads, and then the non-seed-reads aligned to the same seed-read compose a connected component. Further, these components can be connected comprehensively since any pair of nearby seed-reads can be either directly aligned (if they are overlapped) or connected via one or more non-seed-reads having overlaps to both of them.

5) The optimization of performance. xRead optimizes speed and RAM usage by several tailored implementations to achieve outstanding performance and scalability. One is the use of the lightweight alignment skeleton (Step 2) to speedup each iteration, referring to previous studies [45-47]. Another is that, followed by the coverage-guided overlapping strategy, the batch size of the seed-reads is highly tunable so that the whole process can be run with controllable RAM usage." (line 3, page 5 of main text)

Remarks:

The manuscript spends a lot of time evaluating the quality of the overlap graph, which is a very commendable approach and is often overlooked. I thank the authors for this contribution. However, I have issues with the definition of ground truth overlap. Even if two reads do not come from successive parts of the genome, if they share, let's say, a very large perfect overlap, they should indeed overlap in the graph. Considering that the actual biological overlap is necessarily the best one found in the reads is a greedy strategy that could harm the final assembly. Because of this definition, I am not fully convinced by xreads' performance, which seems to employ an overall very greedy strategy.

--Response:

We agree with the reviewer that it could be important for some of the state-of-the-art assemblers to detect all the significant (say, large and perfect) overlaps since their assembly

strategies and heuristics are designed based on such a prior. However, we would also like to claim that a perfect overlapping graph should contain only the overlaps of the reads from successive parts of the genome, as they imply the correct connections of various genomic parts. Other overlaps are in theory false positives and in practice could affect read layout. Especially, the overlaps from various copies of repeats can be very significant, but also misleading. Moreover, to prevent or reduce the misjoins and/or shortened contigs caused by them, many advanced methods have to be used, such as read correction, re-clustering and heuristic layout. This has become a core task for state-of-the-art assemblers, but still non-trivial. In practice, such overlaps usually affect the correctness and continuity of assembly.

xRead is motivated by that each read is a representative of some part of the donor genome. Using this fact, it selects a set of seed-reads covering the whole genome to mimic a “virtual reference” and implements read alignment against the reference to reveal the overlaps to the seed-reads. By this “mimicking-and-mapping” design (as well as the tailored alignment skeletons), the produced graph can simultaneously achieve both high precision (i.e., fewer false positive overlaps) and high connectivity. Such graphs can well-support both of the two strategies commonly used by the state-of-the-art assemblers, i.e., correction-then-assembly and assembly-then-correction. Moreover, it is also possible to convert the graph to other styles, such as a graph restricted to the best or containing all the significant overlaps, which are also popularly used strategies by other overlapping tools. With these features, we believe that xRead is not an over-greedy approach, although it seems simply done. We have added a detailed discussion about the feature of xRead in the Discussion section (also see the quote below).

For the discussion on the features of the xRead approach, we have mentioned in the current version of the manuscript:

“xRead is to some extent designed in a minimalist style that constructs a simplified, but not over-simplified, overlapping graph. The graph can support both of the two strategies commonly used by the state-of-the-art assemblers, i.e., correction-then-assembly [14, 15, 32] and assembly-then-correction [17, 18, 51]. For correction-then-assembly, the approach can implicitly cluster the reads of the same genomic regions since all of them can be aligned to the seed-read(s) from there. Therefore, the graph becomes a suitable input to read correction. For assembly-then-correction, xRead also paves the way to successful layout since the graph is highly connected with few false positive edges. Further, the alignments between non-seed- to seed-reads can also be directly used to infer local consensus in the correction phase. With these features, xRead lays a foundation for downstream assembly steps. It is also worthnoting that, the seed-reads are natively the representatives of various genome parts. Thus, for either of the two strategies, they should be seen as the backbone of assembly and well-handled by specifically designed approaches.

xRead has the potential to convert its produced graph to other styles, such as restricted to the best- or containing all the significant overlaps, which are popularly used strategies by other overlapping tools. This is done by using transitive relationships. It is not difficult for a seed-read to collect all the reads overlapping with it since such reads are either directly aligned to it, or aligned to another seed-read in the same connected component. For the latter case, the assumption also stands due to both of the high connectivity (i.e., seed-reads are well-connected as mentioned) and precision (i.e., in most cases all the transitive edges/overlaps are true

positive) of the graph. Thus, the overlaps to the seed-read can be thoroughly evaluated to find out the best or all the significant ones. It is similar to a non-seed-read, i.e., the read is initially aligned to a seed-read and then involved in a connected component. Further, the overlaps to it can be inferred and evaluated through all the overlaps implied by the component. This feature is partially demonstrated by simulation and real benchmarks (Supplementary Tables 5 and 9), also refer to Supplementary Figure 8A for schematic illustrations." (line 8, page 17 of main text)

We also agree with the reviewer that xRead could have limitations in handling the reads from ultralong repeats, like that of other state-of-the-art approaches. This is a common problem to read overlapping in theory (especially under the crosstalk of repeat and sequencing noise). However, this issue can be addressed in the layout phase during assembly by the many proposed read correction, re-clustering and heuristic layout methods. Moreover, xRead also makes its own efforts to reduce the effects. A discussion has also been added.

For the discussion on the repeat handling of xRead, we have mentioned in the current version of the manuscript:

"xRead still has some limitations to handle the reads from ultralong repeats which is also a common problem to all read overlapping tools. As the lengths are not enough to span such repeats, the reads from various copies are not distinguishable with the crosstalk of their intrinsic similarity and sequencing noise. Under such circumstances, xRead could align the seed-reads from various copies together and map non-seed-reads to some of them randomly. Then all the reads are connected as one component and the graph collapses, which is also a common case during read layout (refer to Supplementary Figure 8B for schematic illustrations). A feasible solution is to use tailored approaches to precisely correct sequencing errors and re-cluster the reads. With the improved base quality, the reads would have a good chance to be distinguished and the graph can be corrected. xRead also makes its own efforts to reduce this effect by two means. One is the CRO-based alignment skeletons which restricts highly confident overlaps and reduces the chance of mapping a read to a wrong copy. Another is that xRead outputs the inferred coverage for each of the reads (in its PAF format output, CV FLAG). This is a good repeat-indicating marker since the repetitive reads have obviously higher coverage, furthermore, they can be conveniently focused in later steps." (line 32, page 17 of main text)

According to the comments and suggestions of the reviewers and editorial board, we also implemented a thorough benchmark to assess the effect of xRead on genome assembly. The results demonstrate that the assembly quality of the xRead-based pipeline can be similar to that of the state-of-the-art assemblers (with higher performance), which also suggests the usability of xRead (see below, the response to the assembly benchmark). Meanwhile, to help the readers more easily understand, we also largely rewrite the overview subsection ("Overview of the xRead approach", Findings section) which gives a thorough explanation of the motivation, design and high-level implementation of xRead approach.

A key selling point of the abstract is the ability of xreads to work with controlled memory usage at the expense of time and external memory usage. Showing some results on this feature would be very interesting, such as a plot showing the time performance depending on memory usage, for example. Also, the amount of external memory used should be discussed.

--Response:

We have followed this suggestion. New results have been added in the manuscript to describe the speed and external memory usage of xRead with various RAM configurations (Supplementary Figure 1 and Supplementary Table 10). In detail, we evaluated the effects of four RAM space configurations (2 GB, 8 GB, 16 GB and 32 GB) across four simulated datasets and four real datasets from various-sized genomes *(E. coli, D. melanogaster, M. musculus* and *H. sapiens*). Overall, the results suggested that xRead kept high speed even if the allowed RAM space was very small (e.g., 2GB). Speedup was also observed with higher RAM space, however, the gain saturated at 16 to 32GB (Supplementary Figure 1A). This is also the reason that xRead uses 16GB as the default setting (which is also available in most modern computers). Moreover, xRead also had lower external memory usage, i.e., about 1% to 50% of that of other tools (Supplementary Figure 1B and Supplementary Table 10). This is helpful for large-scale datasets since such storage costs are also non-neglectable in practice.

We have mentioned in the current version of the manuscript:

"To further investigate the effect of RAM usage on the speed of xRead, we evaluated four other configurations (2 GB, 8 GB, 16 GB and 32 GB) using four low-quality simulated datasets and four real datasets (*E. coli, D. melanogaster, M. musculus* and *H. sapiens*, Supplementary Figure 1A). The results showed that xRead still kept relatively high speeds even with very low RAM usage (e.g., 2GB) and the runtime gain lowered with larger RAM space (e.g., 32GB), suggesting that a small RAM space is enough for xRead. Mainly, the time-space tradeoff comes from the number of reads being indexed. That is, xRead has to load fewer reads at one time with less RAM and this leads to not only more iterations but also a higher number of index-query operations in each iteration, due to that more matching-failures happened during the generation of the alignment skeletons." (line 8, page 8 of main text)

"We also assessed the usage of external memory. xRead showed a 50%-99% off compared to other tools (Supplementary Figure 1B and Supplementary Table 10). It is also worthnoting that xRead showed superior scalability with large-scale datasets. For example, xRead only required 9.2 GB of external memory on the 2 TB simulated A. mexicanum dataset, while MECAT2 consumed 869 GB. This advantage comes from that xRead only records the confident overlaps with high scores, meanwhile, it also avoids transitive overlaps between non-seed-reads which reduces redundancy." (line 17, page 8 of main text)

As far as I understand, the end goal of xreads is to perform efficient de novo assembly. The assembly results should be the primary results of the manuscript and not relegated to the supplementary section. The assembly benchmark should include other assemblers and not only NextDenovo. The assembly results and justification are not quite convincing since the proposed assembler is slightly more resource-efficient at the cost of degraded assembly quality. While the case studies are interesting, it is hard to avoid concluding that the overall quality is degraded compared to regular NextDenovo.

--Response:

We agree with the reviewer that the end goal of xRead is to perform efficient de novo assembly, although currently the tool focuses on efficient overlapping graph construction, as this is one of the major bottlenecks to high performance and scalable assembly. We also

realized the necessity of assembly results and implemented a more thorough benchmark to assess the overall effect of xRead on genome assembly.

Mainly, we used 16 datasets (12 simulated plus 4 real) in total with various sequencing quality for this benchmark. In detail, the 12 simulated datasets are in three error models (low-quality ONT, high-quality ONT and PacBio HiFi) from four genomes (*E. coli, A. thaliana, D. melanogaster* and *H. sapiens*), and for the 4 real datasets, 3 of them are produced by ONT platforms from *C. elegans, D. melanogaster, and H. sapiens* respectively and the other one is also a human dataset but produced by PacBio Hifi sequencing. Moreover, five state-of-the-art assemblers (NextDenovo, Flye, Wtdbg2, Shasta, and Hifiasm) were compared.

For assessing xRead, we tried our best to build an in-house assembly pipeline (termed as xRead-pipe) by using various methods to integrate xRead into a number of assembly pipelines, such as NextDenovo, Wtdbg2, Hifiasm, Shasta, Flye and Canu. It is a pity that most of the state-of-the-art assembly pipelines do not allow a third-party plug-in, partially due to their tailored heuristics which need a tightly coupled implementation for the various modules. Finally, we built the pipeline by borrowing the layout module (NextGraph) from NextDenovo and the consensus module (wtpoa-cns) from Wtdbg2. The selection of these modules is primarily due to their open-source and integration-friendly. However, by this straightforward integration, these two modules also have their own incompatibility (especially NextGraph) which affected the correctness and continuity of the assembly. We also employed an in-house error correction script for post-processing. This script was developed in one of our previous T2T assembly studies and it uses the read realignments to contigs to detect and correct structural errors (large misjoins) in the assembly.

All the assemblers were implemented on the simulated and real datasets, please refer to Table 2, Supplementary Tables 11-12, and Supplementary Figures 2-7 for detailed information about the results. Moreover, we also have added a new subsection ("The use of xRead for de novo assembly") in the Findings section to precisely describe and discuss these results.

For the implementation of assembly benchmark, we have mentioned in the current version of the manuscript ("The use of xRead for de novo assembly", Findings section) as follows:

"The effect of xRead on genome assembly was further assessed. We tried to integrate xRead into a couple of state-of-the-art assemblers, such as NextDenovo [29], Wtdbg2 [17], Hifiasm [32], Shasta [18], Flye [25] and Canu [15]. This is non-trivial since for most of them the various modules (such as overlapping, layout and consensus) are coupled with specific in-memory data structures (also lack of published details) which do not allow flexible plug-ins. Under such circumstances, we made efforts to build an in-house assembly pipeline that uses the xRead graph as input and borrows two open-source modules from other assemblers, i.e., the layout module of NextDenovo (NextGraph) and the consensus module of Wtdbg2 (wtpoa-cns). Further, an in-house post-processing script developed in one of our previous T2T-assembly studies [50] was used to reduce the structural mis-assemblies caused by the incompatibility of the modules. In the benchmark, we assessed the final assemblies (termed as xRead-pipe) as well as the intermediate outputs through NextGraph and wtpoa-cns modules (termed as xRead-nd and xRead-wtpoa respectively).

Twelve simulated datasets (Table 1) from four genomes (*E. coli, A. thaliana, D. melanogaster* and *H. sapiens*) in three error models (low-quality ONT, high-quality ONT and

PacBio HiFi sequencing) were used for benchmark. Moreover, three ONT real datasets (*C. elegans, D. melanogaster* and *H. sapiens*) and a human HiFi dataset (Table 1) were also employed. We tried to implement six pipelines (i.e., xRead-pipe, NextDenovo, Wtdbg2, Flye, Shasta and Hifiasm) on all the 12 (simulated) + 4 (real) datasets for comparison. Since Shasta is not suited to low-quality ONT datasets and Hifiasm is specifically designed for HiFi data, their corresponding results were excluded. QUAST [42] (version 5.2.0) was employed to evaluate the assemblies by the following metrics: assembly length, the number of contigs, N10/N50/N90 statistics, genome fraction, the number of all- and structural mis-assemblies. It is worthnoting that we distinguished the assembly errors by local sequence errors (small-scale sequence expansions and collapses) or structural errors (large-scale misjoins) since the latter are usually much more difficult to resolve and should be more focused.” (line 27, page 13 of main text)

The results indicate that, overall, xRead-pipe achieves comparable completeness, correctness and contiguity to that of state-of-the-art assembles and its performance is obviously higher. Mainly, three key points were observed.

1) Compared to other assemblers, xRead-pipe significantly reduced the time cost and memory usage of genome assembly which indicates outstanding performance and scalability.

2) xRead-pipe achieved similar assembly completeness (assembly length and genome fraction) to that of other assemblers while it had the lowest numbers of structural errors, indicating the high correctness of the assembly.

3) The continuity (N10/N50/N90 statistics) of xRead-pipe was lower (but comparable) to some of the other assemblers. Moreover, it is also observed that the assemblers producing longer contigs usually had more misjoin errors, which indicates a tradeoff between correctness and continuity by various assembly strategies.

For the performance issue (the first point mentioned above), we would also like to claim that the speed and memory improvement of xRead-pipe is non-neglectable. This is not limited to that of the regular NextDenovo (which xRead-pipe has on average 3 times speedup and 29% memory footprint on the simulated and real datasets compared to it). Both the reductions in runtime and RAM usage are meaningful to the whole assembly procedure, especially for those very large genomes, in the following two aspects.

1) For runtime, the cost of the datasets from large genomes could be huge, e.g., Minimap2 (which is the overlapping tool employed by regular NextDenovo) showed nearly prohibitive time cost for the *A. mexicanum* datasets. However, the speed of xRead is not only faster in relative terms, but also scalable to such datasets in absolute terms, which leads to a feasible solution to the assembly task.

2) For RAM usage, the upper limit of xRead is highly controllable (we also showed the results of xRead with very low RAM such as 2GB, as mentioned above). This also makes many large-scale tasks feasible. Shasta is the only pipeline having an obvious faster speed than xRead-pipe, however, its memory footprints are tens of folds higher, e.g., many hundreds of GB for human datasets. This could be also prohibitive to larger genomes. We tried to implement it to handle *A. mexicanum* datasets, however, it ran out of memory on our server (1TB RAM configuration). Thus, the frugal use of computational resources by xRead is commendable, especially coupled with its speed.

For the performance issue, we have mentioned in the current version of the manuscript ("The use of xRead for de novo assembly", Findings section) as follows:

"1) xRead-pipe reduces the overall computational cost of genome assembly.

For nearly all the datasets, both the runtime and memory usage of xRead-pipe is lower than most of the other assemblers (Table 2 and Supplementary Table 11, Real time and Peak Memory columns), suggesting that xRead is beneficial to the overall performance of assembly. Especially, xRead-pipe outperforms the original NextDenovo and Wtdbg2 pipelines by on average 2.5 times speedup and 4.2 times lower memory footprints. It is also worthnoting that Shasta is the fastest one on human datasets while it also has the highest memory footprints, indicating a tradeoff between time- and memory cost. One example is that Shasta has a 776 GB peak memory on the simulated human HiFi dataset (17 and 37 GB for xRead-nd and xRead-wtpoa, respectively) which is a non-neglectable requirement for computational resource and could affect its scalability due to hardware limitation." (line 23, page 14 of main text)

For the correctness and the continuity issues (the second and third points mentioned above), we carefully tracked the behaviors of xRead-pipe and found that the degraded assembly quality shown in the previous version of the manuscript was primarily caused by the incompatibility of the employed layout module (NextGraph). This is mainly due to the following two issues.

1) NextDenovo is in a correction-and-assembly design that uses a read-correction module to correct sequencing errors in advance of the layout. However, the read correction module does not allow the graph produced by xRead as input. Thus, in our pipeline, we forced NextGraph to directly handle the xRead graph and its ability was suppressed.

2) NextGraph handles the graph by its own heuristics and does not fully consider the characteristics of xRead, such as the higher importance of the seed-reads to graph topology, so some of the critical edges in the xRead graph were wrongly handled which led to misjoins and gaps.

These two issues affected both the correctness and continuity of assembly. In the current version, we added wtpoa and the post-processing correction in the pipeline and they rescued a large proportion of the errors caused by NextGraph. Mainly, Wtpoa improved the quality of consensus sequence and reduced a number of sequence errors, especially the ones caused by ONT sequencing noise. It also rescued a proportion of structural errors. Further, our realignment-based correction script effectively detected misjoin regions and eliminated most of the structural errors. Please also refer to Table 2 and Supplementary Table 12 (the rows marked by xRead-nd, xRead-wtpoa, and xRead-pipe, respectively) for the results of various steps and their improvements in assembly quality. With the two modules, xRead-pipe is able to catch up or outperform the regular NextDenovo pipeline (and other assemblers as well) at assembly correctness. Especially, it achieved the lowest number of structural assembly errors.

For the correctness and the continuity issues, we have mentioned in the current version of the manuscript ("The use of xRead for de novo assembly", Findings section) as follows:

"2) xRead-pipe is able to produce correct assemblies.

The QUAST evaluation (Table 2, Supplementary Table 12) indicates that overall xRead-pipe made comparable or lower numbers of mistakes than that of other pipelines, with quite

similar assembly completeness (assembly lengths and genome fractions). This suggests that, using xRead graph, the assembly quality of the pipeline can reach the same level of state-of-the-art tools. We further investigated the types of mis-assemblies and found that, for all the assemblers, most of the mis-assemblies were local sequence errors and the numbers of structural errors were much lower. Especially, the assemblies of xRead-pipe had the lowest numbers of structural errors among all the assemblers. This should be praised since the small errors are easier to fix by advanced error correction and consensus sequence generation approaches. However, nearly all the large misjoins are related to complex repeats and are non-trivial to deal with.

The results of xRead-nd, xRead-wtpoa and xRead-pipe showed decreasing numbers of mis-assemblies, indicating the improvement on correctness by various modules. We further tracked their behaviors and found that a primary cause of the errors was the incompatibility of the layout module. That is, NextDenovo is in a correction-then-assembly design that takes advantage of a tailored correction module to reduce sequencing errors and re-cluster reads in advance. This is critical to read layout, however, it does not allow xRead graphs as input. Under such circumstances, the forced layout module (NextGraph) produced more errors. Further, a large proportion of the errors were amended by wtpoa and the in-house correction script. Wtpoa mainly improved the quality of consensus sequence and reduced a number of sequence errors, especially the ones caused by ONT sequencing noise. It also rescued a proportion of structural errors. Further, by contig realignment (refer to Methods section), the in-house script more effectively detected misjoin regions and largely eliminated the structural errors. With the two modules, xRead-pipe is able to catch up or outperform the original NextDenovo pipeline (and other assemblers as well) at assembly correctness.

There are still errors caused by the crosstalk of xRead and NextGraph that cannot be eliminated. One is due to the very long repeats such as segmental duplications that the read length is still not enough to solve them thoroughly. Other assemblers also have such problems as well. On the other hand, it is also non-neglectable that NextGraph may not fully take advantage of xRead. In the xRead graphs, seed-reads are critical to connect the various parts of the donor genome. They should be given higher weights and used as the backbone during assembly. However, NextGraph does not have such a weight-tuning function. It implemented graph simplification by iteratively resolving nonlinear structures using some heuristics based on read overlap lengths, identities and depths. With its own heuristics, some of the overlaps between seed-reads were mistakenly discarded and leaving structural errors. A typical case is the removal of the z-clip structure (an example is shown in Supplementary Figure 2), i.e., due to the lack of adaptive weighting, some critical edges in nonlinear structures like z-clip structures were mistakenly removed and resulted in the misjoins between non-successive genomic parts.

3) xRead-pipe has comparable continuity to state-of-the-art approaches.

The results (Table 2 and Supplementary Table 12, N10/N50/N90 columns) suggest that the continuity of xRead-pipe is comparable to or slightly lower than that of other assemblers. We investigated the intermediate results of xRead-pipe and found that the decreased continuity is caused by the removal of some critical edges by NextGraph which made assembly gaps. For most of the assembly gaps in the results of xRead-pipe, the successive genome parts were initially connected in the graphs of xRead. The primary cause is still the heuristics of NextGraph

to handle the z-clip structures. As mentioned above, the seed-reads in z-clip structures should serve as the backbone of the xRead graph. However, NextGraph selected the branches by its own rules and did not consider the importance of seed-reads, thus mistakes occurred (an example is in Supplementary Figure 3, and a more complicated one is in Supplementary Figure 4). Therefore, to further improve assembly continuity, a tailored layout method should be developed for xRead to better use the topology as well as other evidence provided by the graph.

Moreover, it is also non-neglectable that a tradeoff can be observed between the continuity and correctness of assembly. That is, the assemblers producing longer contigs also made higher numbers of structural errors. Such errors have no correlation with the lengths of contigs (Supplementary Figure 5), but are related to genomic contexts such as repeats, indicating that they could be partially caused by some relatively aggressive repeat-handling strategies of the assemblers. We realigned the error-containing contigs and investigated the mapping positions of their various parts. With the annotation of RepeatMasker, we found that the errors mostly happened around those well-known repetitive elements such as SINE, LINE, simple tandem repeats and centromeric repeat patterns.

It is worthnoting that the errors may also occur in very long contigs which are usually produced with high confidence. An example is in Supplementary Figure 6. This is a contig of Flye whose length is 50.0 Mb, close to its N10 statistics. A clipping was detected around contig_2932: 42,085,000 which indicates a translocation. The contig was then split into two segments (42.1 Mb and 6.9 Mb, respectively) which can be aligned to reference at the positions Chr3: 88,844,116 and Chr6: 19,746,930, respectively. Another example is in Supplementary Figure 7. This is a contig of NextDenovo whose length is 43.5 Mb, also close to its N10 statistics. A clipping event was observed at ctg003710: 12,544,314, indicating a translocation error in the contig. The contig then can be split aligned to reference at two positions, i.e., Chr3: 91,045,032 and Chr6: 57,667,858." (line 33, page 14 of main text)

Efforts have been made, however, we also realized from the behaviors of xRead-pipe that this straightforwardly integrated pipeline is still far from well-tailored to release all the potentials of xRead. We give a detailed discussion about this fact in the Discussion section. Furthermore, it has been our ongoing work to develop xRead into a novel full assembler to help large-scale de novo sequencing studies. We have mentioned this and described the technical key points at the end of the manuscript.

For the discussions on xRead approach, we have mentioned in the current version of the manuscript:

"The use of xRead to whole genome assembly was also evaluated although it is hard to integrate a standalone overlapping tool to the state-of-the-art assemblers since most of their modules are coupled by various heuristics and do not allow third-party plug-ins. We tried our best to build a pipeline by borrowing the layout and consensus modules from NextDenovo and Wtdbg2, respectively. Considering both the time cost and RAM usage, the pipeline showed higher performance and scalability, meanwhile, it also achieved the same order of completeness, correctness and contiguity as that of those state-of-the-art assemblers. The results suggest that xRead is promising to scalable assembly. Further, we also would like to claim that the ability of xRead is not fully exerted with the straightforward (to some extent rough)

integration. For example, due to the lack of a readily made module, this pipeline can still not fully take advantage of precise read correction (which is critical to repeat-handling). Moreover, existing layout methods also could not fully consider the characteristics of the graphs produced by xRead. It is an important future work (has been ongoing) to develop novel tools based on xRead graph to achieve high-quality, efficient and scalable genome assembly. We realize that there could be three key points to the development as follows.

Firstly, it is critical to develop a tailored read correction method for xRead. The seed-reads can be directly used as anchors to cluster the reads from the same genomic regions. Thus, read correction can be straightforwardly implemented by similar approaches to that of state-of-the-art assemblers, such as multiple sequence alignment [52], pseudo variant calling [53], and sequence graph analysis [14]. Moreover, it also needs to develop novel methods to use the correction information to distinguish and re-cluster the reads from various repeat copies to refine the graph.

Secondly, it also needs to design a specific read layout method with xRead graph. That is, various weights should be added to the edges and carefully considered since the connectivity of the CRO graph depends on the edges and short-paths between seed-reads and they should be well-handled. It is feasible to build an essential backbone by seed-reads as they are well connected and implicitly distributed along the whole genome. Moreover, non-seed-reads can be used as extra evidence to rescue unconnected contigs, prevent mis-assemblies, and support the consensus phase.

Thirdly, it is still an open problem to implement efficient haplotype assembly. For xRead, one feasible way is to expand the graph dynamically with the transitive relationships during layout and precisely analyze the alignments among reads to adaptively reconstruct haplotype-specific paths. Another one is to use the initially assembled genome as reference to implement realignment, variant calling, and phasing, i.e., achieve haplotype assembly in a "de novo- and re-sequencing" way. This approach could be more suited to tasks having various types of reads (such as many T2T-assembly tasks) since the resequencing-style post-processing is more convenient to integrate the data and use various kinds of tools to correct mis-assemblies and construct haplotypes. Moreover, it is also feasible to keep low cost all the way, and even more, simultaneously handle many genomes in a step-parallel approach to achieve very high overall performance in large-scale genomics studies." (line 12, page 18 of main text)

Response to reviewer #2

Reviewer #2: Overall comments.

Authors of the manuscript have developed an iterative overlap graph construction algorithm to support genome assembly. This is both an interesting and a demanding area of research due to very recent advancements in sequencing technologies.

--Response:

Thanks for the comments and suggestions of the reviewer. We have supplied new benchmark results and revised the manuscript according to the reviewer's comments. A point-by-point response has been made according to the comments as follows.

Although the text in the manuscript is interesting, grammar must be rechecked and revised. At some point it is difficult to keep track of the content and references to supplementary to make sense out of the content.

--Response:

We have carefully re-checked the grammar along the whole manuscript (also with the help of the Grammarly tool) and revised the corresponding sentences. Some representative examples are listed below. We have revised the manuscript as follows:

"Step 1: xRead selects a proportion of reads with relatively low coverage and high length as seed-reads and builds a partial read index for them." (line 29, page 4 of main text)

"Therefore, it also holds the possibility of comprehensive graphs with post-processing." (line 12, page 9 of main text)

"R% is the proportion of the reads belonging to the block that has at least one ground truth overlap recalled." (line 26, page 10 of main text)

"Partial evidence is that all the tools have obviously lower numbers of connected components on the human PacBio HiFi dataset than that of the ONT fast mode dataset." (line 28, page 12 of main text)

"Further, xRead computes the average of the window coverage as the estimated coverage for a read." (line 5, page 21 of main text)

Moreover, we have replaced the old supplementary table with the main text (Table 1) to provide detailed information about the datasets used in benchmarks in a more convenient way. We also added the results of the newly implemented assembly benchmark in the main text. The performance and quality of assembly on real datasets are given in Table 2, while those on simulated datasets are in Supplementary Tables 11-12. Other main results regarding read overlap benchmarks are still in Figures 2-3 of the manuscript, with detailed numerical information provided in Supplementary Tables 3-9.

Specific comments

Page 1 Line 13: I believe the authors are talking about assembly sizes and not genome sizes. The sentences here could be a bit short to make them easy to understand.

--Response:

We would like to describe here that the sizes of the donor genomes being sequenced and assembled are at tens of gigabase level, so we used the word “genome size” in the previous version. However, we agree with the reviewer that this word is to some extent unclear, and “assembly size” could be clearer to readers. We have revised the sentence in a shorter and more concise style.

The sentence has been revised as follows:

“However, it is still challenging for assemblers to handle thousands of genomes, tens of gigabase-level assembly sizes and terabase-level datasets efficiently, which is a bottleneck to large-scale de novo sequencing studies.” (line 12, page 1 of main text)

Page 2 Line 19: Theoretical time complexity O(m2n2) is bit of an overstatement due to the heuristics employed by most assemblers. For example, mash distance, minimisers and k-mer bins are there to prevent this explosion of complexity. Either acknowledge such methods or provide a range for the time complexity. I would be interesting to know the time complexities of the methods expressed in sentence starting Line 15.

--Response:

We agree with the reviewer that the $O(m^2n^2)$ time complexity is to some extent an overstatement here. In fact, we would like to claim the theoretical upper limit of the time complexity of all-against-all alignment is $O(m^2n^2)$, i.e., there are *m* reads having *n* bp length so that the time complexity of implementing pairwise alignment for each pair of the reads is $O(m^2n^2)$ in total. We fully agree with the reviewer that the cost should be much lower in practice since many heuristics have been adopted by various tools. We are sorry for the misleading.

To acknowledge this, we have revised the corresponding sentences and mentioned the efforts made by state-of-the-art approaches. Moreover, we also would like to claim in the text that, although many efforts have been made, the time cost is still non-neglectable in absolute terms and still needs further improvements.

We have revised the manuscript as follows:

“However, in theory, the upper limit of time complexity of all-against-all read alignment can be very high, i.e., $O(m^2n^2)$, where m and n are the number and length of the reads, respectively. Many efforts have been made to effectively lower this complexity (see below), however, the time cost is still non-neglectable in absolute terms thus still needs further improvements.” (line 18, page 2 of main text)

“Such approaches retrieve short matches (i.e., seeds) between various reads (usually through indexing data structures) and conduct extended alignments around them to confirm the actual overlapped parts of the reads. The time cost can be significantly reduced with them, as the alignments focus on some pairs of reads being matched whose number is much lower than $O(m^2)$. However, the real cost depends on the various adopted strategies as well.” (line 28, page 2 of main text)

Page 5 Line 11: Was this performed with overlapping windows of 1gb? Otherwise, simulations may not have reads spanning across such regions.

--Response:

The *A. mexicanum* dataset was generated by non-overlapping windows. We are sorry for the unclear description. In detail, due to the chromosome length limitations of PBSIM, we split the 15 chromosomes of the *A. mexicanum* genome into 30 chromosomes with length < 1Gbp, to generate a converted reference file. In the overlap benchmark, we used the converted reference to simulate the dataset to evaluate the ability of various tools. We agree with the reviewer that there is no read spanning the partitioned chromosomes. In our opinion, these partitioned chromosomes were used as distinct chromosomes in the benchmark and this conversion did not affect the benchmark too much.

To more clearly describe this issue, we revised the manuscript as follows:

"It is also worthnoting that we partitioned the chromosomes of the A. mexicanum genome in the simulation due to the limit of PBSIM (15 of the chromosomes were divided into 30 <1Gbp ones in advance)." (line 17, page 6 of main text)

Page 5 Line 14: It seems you are simulating 9 + 4 + 4 datasets. This is unclear, please make this into bullet points or separate paragraphs and explain clearly. Include simulator information in the table itself by may be making it landscape (in supplementary).

--Response:

We agree that in the previous version the description about the datasets is not clear enough to read. We have added a new subsection in the current version of the manuscript (titled "The datasets used in benchmark") to clearly describe the 17 simulated datasets, i.e., 9 + 4 + 4 datasets in three error models (low-quality ONT, high-quality ONT and PacBio HiFi sequencing, respectively). We replaced the old supplementary table with Table 1 in the manuscript to show detailed information more clearly about the configurations of the simulator for these datasets, including the error model, error rate, read length and read depth.

We have revised the manuscript as follows:

"The datasets used in benchmark

We simulated 17 datasets by PBSIM [48, 49] and also employed 7 real datasets (Table 1) for the benchmarks of read overlapping and genome assembly. The datasets are from nine genomes (Supplementary Table 1) having small- (<1Gbp, i.e., *E. coli, S. cerevisiae, C. elegans, A. thaliana,* and *D. melanogaster*), large- (>1Gbp, i.e., *Z. mays, M. musculus,* and *H. sapiens*) and very large- (>10Gbp, i.e., *A. mexicanum*) sizes. It is also worthnoting that we partitioned the chromosomes of the *A. mexicanum* genome in the simulation due to the limit of PBSIM (15 of the chromosomes were divided into 30 <1Gbp ones in advance).

To assess the performance of xRead in various error rates, we respectively simulated nine low-quality, four high-quality ONT-like and four HiFi-like datasets (50x coverage each) as follows.

1) The nine low-quality ONT datasets were simulated for all nine genomes with the pre-trained R103 chemistry model of PBSIM. The mean read length and total error rate were 13 kbp and 13%, respectively, referring to a previous study [3]. These datasets are highly noisy

like the fast base-calling mode of ONT platforms and employed to assess the robustness of xRead to sequencing errors for various-sized genomes.

2) The four high-quality ONT datasets were also simulated from four genomes (*E. coli, A. thaliana, D. melanogaster* and *H. sapiens*). The QSHMM-ONT-HQ model was used (the mean read length and average accuracy were 15 kbp and 94%, respectively) to mimic the data produced by mostly used ONT platforms (especially for its HAC mode).

3) The four HiFi-like datasets were produced by sample-based simulation of PBSIM with a real sequencing dataset (mean read length: 16.6 kbp and average base accuracy: 99.6%) from four genomes (*E. coli, A. thaliana, D. melanogaster* and *H. sapiens*). These datasets mimic the data produced by currently used PacBio platforms." (line 12, page 6 of main text)

Fig 2: I believe authors should expand their analysis to more recent and popular assemblers. For example, wtdbg2 is designed for noisy reads and not specifically for more accurate R10/ HiFi reads. So please include, HiFi-asm, Flye where appropriate. Flye supports ONT out of the box and in my experience does produce good assemblies.
Although, you are evaluating read overlaps, it is hard to ignore assemblers themselves just because they do not produce intermediate overlaps graphs.

--Response:

We followed the suggestion from the reviewer. In the current version of the manuscript, we implemented a more thorough benchmark to assess the effect of xRead on genome assembly. Mainly, we used in total of 16 datasets (12 simulated plus 4 real) with various sequencing qualities for this benchmark. In detail, the 12 simulated datasets are in three error models (low-quality ONT, high-quality ONT, and PacBio HiFi) from four genomes (*E. coli, A. thaliana, D. melanogaster* and *H. sapiens*), and for the 4 real datasets, 3 of them are produced by ONT platforms from *C. elegans, D. melanogaster,* and *H. sapiens* respectively and the other one is also a human dataset but produced by PacBio Hifi sequencing. Moreover, five state-of-the-art assemblers (NextDenovo, Flye, Wtdbg2, Shasta, and Hifiasm) were compared.

For assessing xRead, we tried our best to build an in-house assembly pipeline (termed as xRead-pipe) by using various methods to integrate xRead into a number of assembly pipelines, such as NextDenovo, Wtdbg2, Hifiasm, Shasta, Flye and Canu. It is a pity that most of the state-of-the-art assembly pipelines do not allow a third-party plug-in, partially due to their tailored heuristics which need a highly coupled design for the various modules. Finally, we built the pipeline by borrowing the layout module (NextGraph) from NextDenovo and the consensus module (wtpoa-cns) from Wtdbg2. The selection of these modules is primarily due to their open-source and integration-friendly. However, by this straightforward integration, these two modules also had their own incompatibility (especially NextGraph) which affected the correctness and continuity of the assembly. We also employed an in-house error correction script for post-processing. This script was developed in one of our previous T2T genome assembly studies and it uses the read realignments to contigs to detect and correct structural errors (large misjoins) in the assembly.

All the assemblers were implemented on the simulated and real datasets, please refer to Table 2, Supplementary Tables 11-12, and Supplementary Figures 2-7 for detailed information about the results. Moreover, we also have added a new subsection ("The use of xRead for de novo assembly") in the Findings section to precisely describe and discuss these results.

For the implementation of assembly benchmark, we have mentioned in the current version of the manuscript ("The use of xRead for de novo assembly", Findings section) as follows:

"The effect of xRead on genome assembly was further assessed. We tried to integrate xRead into a couple of state-of-the-art assemblers, such as NextDenovo [29], Wtdbg2 [17], Hifiasm [32], Shasta [18], Flye [25] and Canu [15]. This is non-trivial since for most of them the various modules (such as overlapping, layout and consensus) are coupled with specific in-memory data structures (also lack of published details) which do not allow flexible plug-ins. Under such circumstances, we made efforts to build an in-house assembly pipeline that uses the xRead graph as input and borrows two open-source modules from other assemblers, i.e., the layout module of NextDenovo (NextGraph) and the consensus module of Wtdbg2 (wtpoa-cns). Further, an in-house post-processing script developed in one of our previous T2T-assembly studies [50] was used to reduce the structural mis-assemblies caused by the incompatibility of the modules. In the benchmark, we assessed the final assemblies (termed as xRead-pipe) as well as the intermediate outputs through NextGraph and wtpoa-cns modules (termed as xRead-nd and xRead-wtpoa respectively).

Twelve simulated datasets (Table 1) from four genomes (*E. coli, A. thaliana, D. melanogaster* and *H. sapiens*) in three error models (low-quality ONT, high-quality ONT and PacBio HiFi sequencing) were used for benchmark. Moreover, three ONT real datasets (*C. elegans, D. melanogaster* and *H. sapiens*) and a human HiFi dataset (Table 1) were also employed. We tried to implement six pipelines (i.e., xRead-pipe, NextDenovo, Wtdbg2, Flye, Shasta and Hifiasm) on all the 12 (simulated) + 4 (real) datasets for comparison. Since Shasta is not suited to low-quality ONT datasets and Hifiasm is specifically designed for HiFi data, their corresponding results were excluded. QUAST [42] (version 5.2.0) was employed to evaluate the assemblies by the following metrics: assembly length, the number of contigs, N10/N50/N90 statistics, genome fraction, the number of all- and structural mis-assemblies. It is worthnoting that we distinguished the assembly errors by local sequence errors (small-scale sequence expansions and collapses) or structural errors (large-scale misjoins) since the latter are usually much more difficult to resolve and should be more focused." (line 27, page 13 of main text)

Overall, compared to all the other assemblers in the benchmark, xRead-pipe achieved significantly higher performance and comparable assembly quality (completeness, correctness and contiguity). Mainly, three key points were observed from the results as follows.

1) Compared to other assemblers, xRead-pipe significantly reduced the time cost and memory usage of genome assembly which indicates outstanding performance and scalability.

2) xRead-pipe achieved similar assembly completeness (assembly length and genome fraction) to that of other assemblers while it has the lowest numbers of structural errors, indicating the high correctness of the assembly.

3) The continuity (N10/N50/N90 statistics) of xRead-pipe is lower (but comparable) to some of the other assemblers. Moreover, it is also observed that the assemblers producing longer contigs usually have more misjoin errors, which indicates a tradeoff between correctness and continuity by various assembly strategies.

We carefully tracked the behaviors of xRead-pipe and found that, the pipeline still has not released all the potentials of xRead through this straightforward and forced integration of various modules yet, although it has achieved considerable quality and performance. This is

mainly due to the following two issues caused by the incompatibility of NextGraph.

1) NextDenovo is in a correction-and-assembly design that uses a read correction module to correct sequencing errors in advance of the layout. However, the read correction module does not allow the graph produced by xRead as input. Thus, in our pipeline, we forced NextGraph to directly handle the graph and its ability was suppressed.

2) NextGraph handles the graph by its own heuristics and does not fully consider the characteristics of xRead, such as the higher importance of the seed-reads to graph topology, so that some of the critical edges in the xRead graph were wrongly handled which led to misjoins and gaps.

Such incompatibility affected both the correctness and continuity of assembly. In the current version, the errors caused by NextGraph were largely amended by the newly added wtpoa and the post-processing module. Mainly, Wtpoa improved the quality of consensus sequence and reduced a number of sequence errors, especially the ones caused by ONT sequencing noise. It also rescued a proportion of structural errors. Further, our realignment-based correction script effectively detected misjoin regions and eliminated most of the structural errors. Please also refer to Table 2 and Supplementary Table 12 (the lines marked by xRead-nd, xRead-wtpoa, and xRead-pipe, respectively) for the results of various steps and their improvements in assembly quality. With the two modules, xRead-pipe is able to catch up or outperform the regular NextDenovo pipeline (and other assemblers as well) at assembly correctness. Especially, it achieved the lowest number of structural assembly errors.

For the results of assembly benchmarks, we have mentioned in the current version of the manuscript ("The use of xRead for de novo assembly", Findings section) as follows:

"The results are in Table 2 (real) and Supplementary Tables 11-12 (simulated), respectively. It indicates that the completeness, correctness and contiguity of xRead-pipe are close to that of state-of-the-art assemblers, while its performance is obviously higher. Some intermediate results also suggested that some features of xRead were also not being fully utilized by this straightforward integration and it also has the potential to further improve assembly quality by more tailored layout and consensus modules. Mainly, three issues were observed as follows.

1) xRead-pipe reduces the overall computational cost of genome assembly.

For nearly all the datasets, both the runtime and memory usage of xRead-pipe is lower than most of the other assemblers (Table 2 and Supplementary Table 11, Real time and Peak Memory columns), suggesting that xRead is beneficial to the overall performance of assembly. Especially, xRead-pipe outperforms the original NextDenovo and Wtdbg2 pipelines by on average 2.5 times speedup and 4.2 times lower memory footprints. It is also worthnoting that Shasta is the fastest one on human datasets while it also has the highest memory footprints, indicating a tradeoff between time- and memory cost. One example is that Shasta has a 776 GB peak memory on the simulated human HiFi dataset (17 and 37 GB for xRead-nd and xRead-wtpoa, respectively) which is a non-neglectable requirement for computational resource and could affect its scalability due to hardware limitation.

2) xRead-pipe is able to produce correct assemblies.

The QUAST evaluation (Table 2, Supplementary Table 12) indicates that overall xRead-pipe made comparable or lower numbers of mistakes than that of other pipelines, with quite

similar assembly completeness (assembly lengths and genome fractions). This suggests that, using xRead graph, the assembly quality of the pipeline can reach the same level of state-of-the-art tools. We further investigated the types of mis-assemblies and found that, for all the assemblers, most of the mis-assemblies were local sequence errors and the numbers of structural errors were much lower. Especially, the assemblies of xRead-pipe had the lowest numbers of structural errors among all the assemblers. This should be praised since the small errors are easier to fix by advanced error correction and consensus sequence generation approaches. However, nearly all the large misjoins are related to complex repeats and are non-trivial to deal with.

The results of xRead-nd, xRead-wtpoa and xRead-pipe showed decreasing numbers of mis-assemblies, indicating the improvement on correctness by various modules. We further tracked their behaviors and found that a primary cause of the errors was the incompatibility of the layout module. That is, NextDenovo is in a correction-then-assembly design that takes advantage of a tailored correction module to reduce sequencing errors and re-cluster reads in advance. This is critical to read layout, however, it does not allow xRead graphs as input. Under such circumstances, the forced layout module (NextGraph) produced more errors. Further, a large proportion of the errors were amended by wtpoa and the in-house correction script. Wtpoa mainly improved the quality of consensus sequence and reduced a number of sequence errors, especially the ones caused by ONT sequencing noise. It also rescued a proportion of structural errors. Further, by contig realignment (refer to Methods section), the in-house script more effectively detected misjoin regions and largely eliminated the structural errors. With the two modules, xRead-pipe is able to catch up or outperform the original NextDenovo pipeline (and other assemblers as well) at assembly correctness.

There are still errors caused by the crosstalk of xRead and NextGraph that cannot be eliminated. One is due to the very long repeats such as segmental duplications that the read length is still not enough to solve them thoroughly. Other assemblers also have such problems as well. On the other hand, it is also non-neglectable that NextGraph may not fully take advantage of xRead. In the xRead graphs, seed-reads are critical to connect the various parts of the donor genome. They should be given higher weights and used as the backbone during assembly. However, NextGraph does not have such a weight-tuning function. It implemented graph simplification by iteratively resolving nonlinear structures using some heuristics based on read overlap lengths, identities and depths. With its own heuristics, some of the overlaps between seed-reads were mistakenly discarded and leaving structural errors. A typical case is the removal of the z-clip structure (an example is shown in Supplementary Figure 2), i.e., due to the lack of adaptive weighting, some critical edges in nonlinear structures like z-clip structures were mistakenly removed and resulted in the misjoins between non-successive genomic parts.

3) xRead-pipe has comparable continuity to state-of-the-art approaches.

The results (Table 2 and Supplementary Table 12, N10/N50/N90 columns) suggest that the continuity of xRead-pipe is comparable to or slightly lower than that of other assemblers. We investigated the intermediate results of xRead-pipe and found that the decreased continuity is caused by the removal of some critical edges by NextGraph which made assembly gaps. For most of the assembly gaps in the results of xRead-pipe, the successive genome parts were initially connected in the graphs of xRead. The primary cause is still the heuristics of NextGraph

to handle the z-clip structures. As mentioned above, the seed-reads in z-clip structures should serve as the backbone of the xRead graph. However, NextGraph selected the branches by its own rules and did not consider the importance of seed-reads, thus mistakes occurred (an example is in Supplementary Figure 3, and a more complicated one is in Supplementary Figure 4). Therefore, to further improve assembly continuity, a tailored layout method should be developed for xRead to better use the topology as well as other evidence provided by the graph.

Moreover, it is also non-neglectable that a tradeoff can be observed between the continuity and correctness of assembly. That is, the assemblers producing longer contigs also made higher numbers of structural errors. Such errors have no correlation with the lengths of contigs (Supplementary Figure 5), but are related to genomic contexts such as repeats, indicating that they could be partially caused by some relatively aggressive repeat-handling strategies of the assemblers. We realigned the error-containing contigs and investigated the mapping positions of their various parts. With the annotation of RepeatMasker, we found that the errors mostly happened around those well-known repetitive elements such as SINE, LINE, simple tandem repeats and centromeric repeat patterns.

It is worthnoting that the errors may also occur in very long contigs which are usually produced with high confidence. An example is in Supplementary Figure 6. This is a contig of Flye whose length is 50.0 Mb, close to its N10 statistics. A clipping was detected around contig_2932: 42,085,000 which indicates a translocation. The contig was then split into two segments (42.1 Mb and 6.9 Mb, respectively) which can be aligned to reference at the positions Chr3: 88,844,116 and Chr6: 19,746,930, respectively. Another example is in Supplementary Figure 7. This is a contig of NextDenovo whose length is 43.5 Mb, also close to its N10 statistics. A clipping event was observed at ctg003710: 12,544,314, indicating a translocation error in the contig. The contig then can be split aligned to reference at two positions, i.e., Chr3: 91,045,032 and Chr6: 57,667,858." (line 17, page 14 of main text)

Efforts have been made, however, we also realized that it is important to develop xRead into a tailored full genome assembly tool to help large-scale de novo sequencing studies. This has been an ongoing work for us. Moreover, by summing up all the results and analysis, we also give the technical key points to develop the novel assembler at the end of the manuscript.

For the key points of our ongoing work to develop xRead, we have mentioned in the current version of the manuscript:

"The use of xRead to whole genome assembly was also evaluated although it is hard to integrate a standalone overlapping tool to the state-of-the-art assemblers since most of their modules are coupled by various heuristics and do not allow third-party plug-ins. We tried our best to build a pipeline by borrowing the layout and consensus modules from NextDenovo and Wtdbg2, respectively. Considering both the time cost and RAM usage, the pipeline showed higher performance and scalability, meanwhile, it also achieved the same order of completeness, correctness and contiguity as that of those state-of-the-art assemblers. The results suggest that xRead is promising to scalable assembly. Further, we also would like to claim that the ability of xRead is not fully exerted with the straightforward (to some extent rough) integration. For example, due to the lack of a readily made module, this pipeline can still not fully take advantage of precise read correction (which is critical to repeat-handling). Moreover,

existing layout methods also could not fully consider the characteristics of the graphs produced by xRead. It is an important future work (has been ongoing) to develop novel tools based on xRead graph to achieve high-quality, efficient and scalable genome assembly. We realize that there could be three key points to the development as follows.

Firstly, it is critical to develop a tailored read correction method for xRead. The seed-reads can be directly used as anchors to cluster the reads from the same genomic regions. Thus, read correction can be straightforwardly implemented by similar approaches to that of state-of-the-art assemblers, such as multiple sequence alignment [52], pseudo variant calling [53], and sequence graph analysis [14]. Moreover, it also needs to develop novel methods to use the correction information to distinguish and re-cluster the reads from various repeat copies to refine the graph.

Secondly, it also needs to design a specific read layout method with xRead graph. That is, various weights should be added to the edges and carefully considered since the connectivity of the CRO graph depends on the edges and short-paths between seed-reads and they should be well-handled. It is feasible to build an essential backbone by seed-reads as they are well connected and implicitly distributed along the whole genome. Moreover, non-seed-reads can be used as extra evidence to rescue unconnected contigs, prevent mis-assemblies, and support the consensus phase.

Thirdly, it is still an open problem to implement efficient haplotype assembly. For xRead, one feasible way is to expand the graph dynamically with the transitive relationships during layout and precisely analyze the alignments among reads to adaptively reconstruct haplotype-specific paths. Another one is to use the initially assembled genome as reference to implement realignment, variant calling, and phasing, i.e., achieve haplotype assembly in a "de novo- and re-sequencing" way. This approach could be more suited to tasks having various types of reads (such as many T2T-assembly tasks) since the resequencing-style post-processing is more convenient to integrate the data and use various kinds of tools to correct mis-assemblies and construct haplotypes. Moreover, it is also feasible to keep low cost all the way, and even more, simultaneously handle many genomes in a step-parallel approach to achieve very high overall performance in large-scale genomics studies." (line 12, page 18 of main text)

Page 5-9: In the benchmarks section, please include how True Positives and False Positives were labelled. Was this from simulation data?

--Response:

We revised the sentences about the definition of True Positives and False Positives overlaps, along with the computation of sensitivity and precision ("Assessment of Sensitivity and Precision in Produced Graphs", Methods section). Since the definitions of True Positives and False Positives are critical to the assessment, we precisely explained their definitions. Meanwhile, in our opinion, it is better to put this paragraph in the Methods section since it seems a little verbose in the Findings section and affects the readability. We have also added a note in the Findings section to guide readers to the detailed information.

The True Positives and False Positives overlaps are defined for both simulation and real data benchmark, respectively using the different methods as follows.

For simulated datasets, the ground truth positions of reads in the donor genome are directly provided by the PBSIM output files, so that the ground truth overlap set can be established by

the overlaps (longer than 500 bp) of the reads at successive genomic positions. Then in the outputs of a read overlapping tool, the overlaps within the ground truth set are considered true positives, and others are as false positives.

For real sequencing datasets, we defined pseudo-ground truth overlaps. That is, we aligned the reads to the reference genome at first. With the high mappability of long reads, their mapping positions are used as ground truth read positions, and pseudo-ground truth is built by the relative positions of the reads. Further, the same criteria are applied with pseudo-ground truth overlap set to determine the true- and false positives of various tools.

The note in the Findings section of the manuscript that guides readers to the Methods is written as follows:

"We evaluated the overall precisions and sensitivities of the tools (Fig. 2b and Supplementary Table 4, Precision and Sensitivity columns, also refer to Methods section for more details about assessment, including the definitions of ground truth, true positive and false positive overlaps, as well as the computation of precision and sensitivity)." (line 25, page 8 of main text)

The definitions of true positives and false positives overlaps are provided as follows:

"We use both of simulated and real datasets in various read lengths and quality to evaluate the ability of xRead. The precision and sensitivity of the produced graph were assessed with the ground truth edge set of the overlapping graph (short as ground truth overlap set). The ground truth overlap set was generated based on the genomic positions of the reads. For simulated datasets, the read positions are directly given by the output files of the simulator (PBSIM). For real datasets, due to the absence of ground truth, we take advantage of the high mappability of long reads to produce pseudo-ground truth. That is, the reads were aligned to the corresponding reference genome using Minimap2 with default settings. The reads being unaligned or in low mapping quality were marked as ambiguous reads and filtered out, i.e., unused in the benchmark. Further, the remaining reads as well as their mapping positions were used to compose the pseudo-ground truth set. The overlaps between reads were then collected based on (pseudo-) ground truth read positions and used to produce the (pseudo-) ground truth overlap set. Since too short overlaps could be caused by coincidence and most of them could be directly removed in downstream assembly steps, herein, only the read overlaps longer than 500bp were considered in the evaluation. This criteria also refers to previous studies [13].

The generated (pseudo-) ground truth overlap set was then used to evaluate the precision and sensitivity. Any reported overlap was considered as a true positive only if it matched an overlap in the ground truth overlap set. It is worthnoting that a read from a real dataset may have multiple positions due to the ambiguity of alignment. In such cases, we consider an overlap to be true positive if it matches any of the overlaps derived from corresponding reads in the ground truth set. Any non-true-positive overlap is seen as a false positive overlap. The precision and sensitivity were then calculated as $\mathrm{N}_{\mathrm{O}}^{TP}/N_O^R$ and $\mathrm{N}_{\mathrm{O}}^{TP}/N_O^G$, where $\mathrm{N}_{\mathrm{O}}^{TP}$, N_O^R and N_O^G are the number of true positive overlaps, reported overlaps, and overlaps in the ground truth set, respectively." (line 25, page 21 of main text)

Page 11: Use of xRead has been evaluated on genome assemblies. This is a very important and it is a bit unfortunate that existing assemblers are not very flexible in terms of plugging in new intermediate steps. It might be worth exploring into creating a new assembler using the wtpoa2 cli command of wtdbg2.

--Response:

Most of the state-of-the-art assemblers indeed do not allow a third-party plug-in. However, the wtpoa-cns module of Wtdbg2 is friendly to use as a standalone module for polishing the draft assembly. We appreciate the suggestion of the reviewer.

It has been used as the consensus module of the newly built pipeline (xRead-pipe) in the current version of the manuscript. In xRead-pipe, wtpoa-cns helped to improve the quality of assembly and reduce a number of misassemblies caused by the layout module (NextGraph, borrowed from NextDenovo) and sequencing errors, especially for noisy ONT datasets. The results of wtpoa-cns step are shown in all the genome assembly benchmarks (Table 2 and Supplementary Table 12, termed as xRead-wtpoa).

For building the xRead-wtpoa pipeline, we have mentioned in the current version of the manuscript as follows:

"This is non-trivial since for most of them the various modules (such as overlapping, layout and consensus) are coupled with specific in-memory data structures (also lack of published details) which do not allow flexible plug-ins. Under such circumstances, we made efforts to build an in-house assembly pipeline that uses the xRead graph as input and borrows two open-source modules from other assemblers, i.e., the layout module of NextDenovo (NextGraph) and the consensus module of Wtdbg2 (wtpoa-cns). Further, an in-house post-processing script developed in one of our previous T2T-assembly studies [50] was used to reduce the structural mis-assemblies caused by the incompatibility of the modules. In the benchmark, we assessed the final assemblies (termed as xRead-pipe) as well as the intermediate outputs through NextGraph and wtpoa-cns modules (termed as xRead-nd and xRead-wtpoa respectively)." (line 29, page 13 of main text)

For the corresponding results of xRead-wtpoa, we have mentioned in the current version of the manuscript as follows:

"The results of xRead-nd, xRead-wtpoa and xRead-pipe showed decreasing numbers of mis-assemblies, indicating the improvement on correctness by various modules. We further tracked their behaviors and found that a primary cause of the errors was the incompatibility of the layout module. That is, NextDenovo is in a correction-then-assembly design that takes advantage of a tailored correction module to reduce sequencing errors and re-cluster reads in advance. This is critical to read layout, however, it does not allow xRead graphs as input. Under such circumstances, the forced layout module (NextGraph) produced more errors. Further, a large proportion of the errors were amended by wtpoa and the in-house correction script. Wtpoa mainly improved the quality of consensus sequence and reduced a number of sequence errors, especially the ones caused by ONT sequencing noise. It also rescued a proportion of structural errors. Further, by contig realignment (refer to Methods section), the in-house script more effectively detected misjoin regions and largely eliminated the structural errors. With the two

modules, xRead-pipe is able to catch up or outperform the original NextDenovo pipeline (and other assemblers as well) at assembly correctness." (line 11, page 15 of main text)

Page 16: What will happen if you only capture reads from a single chromosome due to longer length? I believe the objective is to gather longest reads capturing as much as possible covering the whole genome. Please comment on this.

--Response:

Due to the randomness of long read sequencing data, in practice it is not very possible that all the reads have the largest lengths coming from some specific genomic regions like a single chromosome. However, we agree with the reviewer that this is an interesting case for xRead approach. In fact, the seed-read selection of xRead is not only guided by the read length, but more importantly, the coverage of the read from other reads. That is, in each iteration, the selected reads are the longest ones that still have not been mapped to previously selected seed-reads. A more detailed explanation is below.

Overall, the whole process of xRead is in a "mimicking-and-mapping" design. It is motivated by that each read is a representative of some part of the donor genome and a selected set of seed-reads covering the whole genome can mimic a "virtual reference" to reveal the read overlaps through a read-to-reference mapping. The seed-reads are regarded as the "virtual reference", and to achieve the goal, xRead uses the tailored read-coverage-based heuristics to ensure two critical features, i.e., the completeness of virtual reference and the mappability of virtual reference.

To achieve the completeness of virtual reference, xRead uses a fact of read mapping, i.e., given an arbitrary set of seed-reads, relatively high coverages (i.e., times of being aligned) can be derived for all the reads belonging to the virtual-reference covered regions. Meanwhile, the reads from uncovered regions have much lower coverage. Thus, the coverage becomes an indicator to select the representative reads from those uncovered regions. Furthermore, the reads from various genomic regions can be progressively explored, selected (as seed-reads), and connected (aligned together) by the iterations, even if the seed-reads initially come from a single chromosome or some specific regions.

To achieve the mappability of virtual reference, xRead further selects the longest reads from the set of unmapped reads. This is also based on the fact of arbitrary selection, i.e., with the coverage-guided heuristics, the whole donor genome and all of the reads can be fully explored by even arbitrary selection of seed-reads. Under such circumstances, it is feasible to directly select the longest low-covered read in each iteration, to improve the mappability of the virtual reference.

In the current version of the manuscript, we have largely rewritten the subsection "Overview of the xRead approach" to add more precise descriptions and explanations about xRead approach.

We have mentioned in the manuscript as follows:

"Unlike state-of-the-art overlapping tools that in essence push each of the reads to explore all the other ones with heuristics, xRead adopts a "mimicking-and-mapping" design. Mainly, it is motivated by that each read can be seen as a representative of some part of the donor genome. A selected set of seed-reads covering the whole genome can mimic a "virtual

reference” to reveal read-overlaps through a read-to-reference mapping, i.e., the reads from the same region can be implicitly aligned to the corresponding seed-read(s) in the virtual reference. So, all the overlaps between seed and non-seed-reads can be detected and the various parts of the genome can also be connected by such alignments. Guided by a novel coverage-based strategy of seed-read selection (“the completeness of virtual reference”, see below), xRead constructs the overlapping graph in an iterative process (Fig. 1).” (line 18, page 4 of main text)

“This approach lays a new foundation for genome assembly in two aspects. Firstly, it is apparent that the cost can be substantially reduced by converting all-against-all read alignment to a read-to-reference mapping task. Secondly, the produced graph can also be correctly connected, i.e., achieve high precision and connectivity simultaneously, which is supportive to both of the popular strategies in long read assembly (i.e., correction-then-assembly and assembly-then-correction, also refer to Discussion section for a more detailed explanation). These are done by some tailored implementations for several critical issues as follows.

1) The completeness of virtual reference. To achieve the best performance, it is fundamental to select a set of seed-reads covering the whole genome and having as few as possible redundant reads, but non-trivial. xRead achieves this goal by using a fact of the read mapping. That is, given an arbitrary set of seed-reads, relatively high coverage (i.e., times of being aligned) can be derived for all the reads belonging to the virtual-reference covered regions, which is done by exploring the produced alignments and their transitive relationships. Meanwhile, the reads from uncovered regions have much lower coverage. Thus, the coverage becomes a useful indicator to iteratively select the reads from those still uncovered regions and the whole genome can be implicitly and progressively explored. This assumption could be over-simplified for the reads from ultralong repeats, however, it does not affect the overlapping task much (refer to Discussion section for more details).

2) The mappability of virtual reference. A high mappability of virtual reference (seed-reads) is useful to achieve confident read mapping. Thanks to the randomness and less bias of long-read sequencing [42-44], there is no obvious correlation between the read lengths and positions. Thus, with the arbitrary selection assumption mentioned above, it is feasible to straightforwardly select the longest ones from the remaining reads to maximize the mappability of the virtual reference.” (line 3, page 5 of main text)

Page 19: In the Github Readme the download URL was wrong. Please correct it to the latest release

Correct: https://github.com/tcKong47/xRead/releases/download/xRead-v1.0.0.1/xRead-v1.0.0.tar.gz

Existing: https://github.com/tcKong47/xRead/releases/download/v1.0.0/xRead-v1.0.0.tar.gz

Make command failed with

make: *** No rule to make target `main.h', needed by `main.o'. Stop.

It seems the release does not have source code, but rather the compiled version. Please update github instructing how to compile code properly with a git clone.

--Response:

We are very sorry for the inconvenience caused by the incorrect URL.

We checked and updated the source code URL on GitHub, as well as the “Instruction” section of README.md. We also verified the availability of all URLs. All of them have been ensured to be correct and available now.

The new URL including the source code is as follows:

https://github.com/tcKong47/xRead/releases/download/v1.0.0/xRead-v1.0.0.tar.gz

The updated compiling instructions are as follows:

```
wget https://github.com/tcKong47/xRead/releases/download/v1.0.0/xRead-v1.0.0.tar.gz
tar -zxvf xRead-v1.0.0.tar.gz
cd xRead-v1.0.0; make
```

Figure 1

Click here to access/download;Figure;fig1.pdf

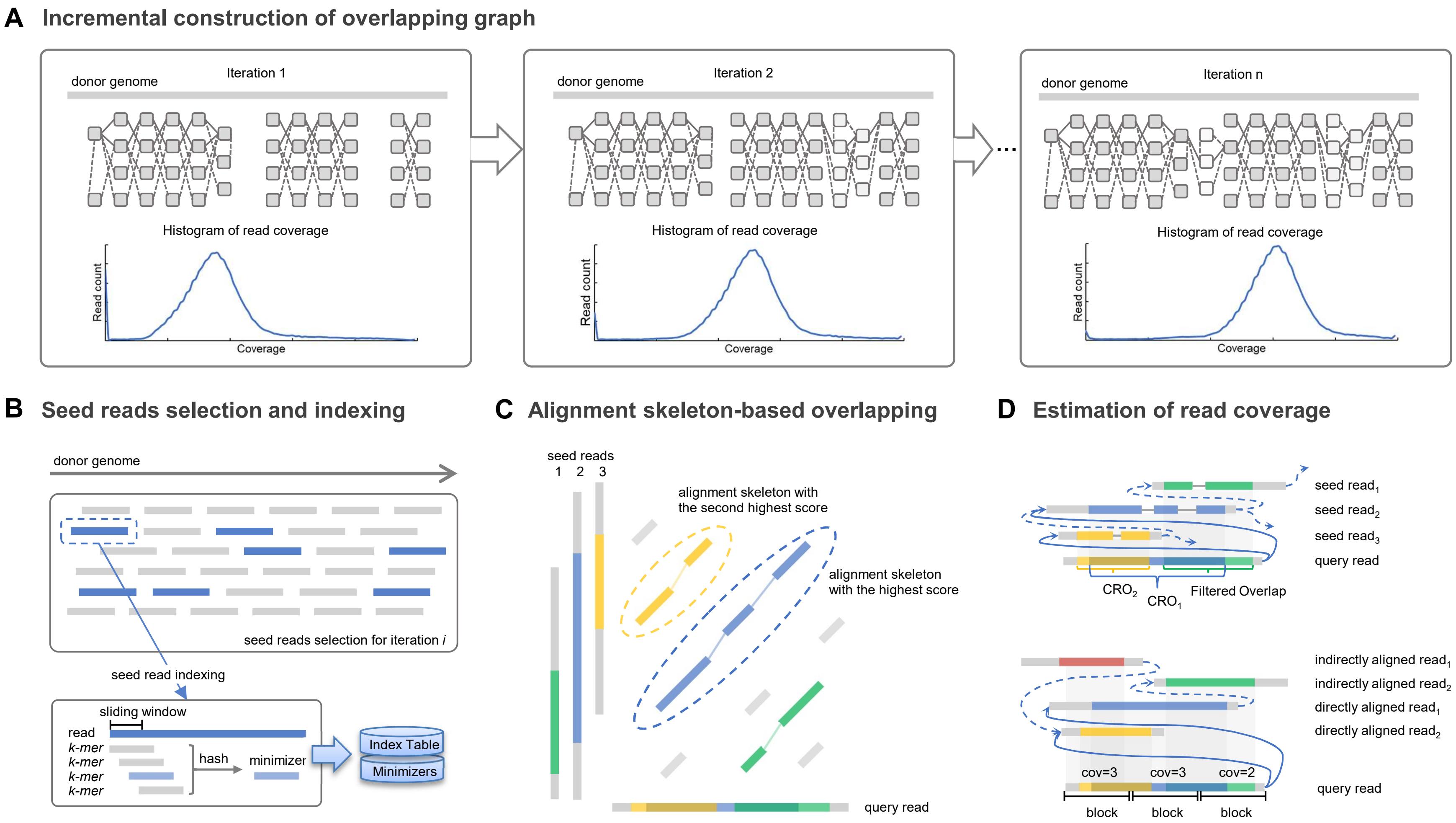

Figure 2

Click here to access/download;Figure;fig2.pdf

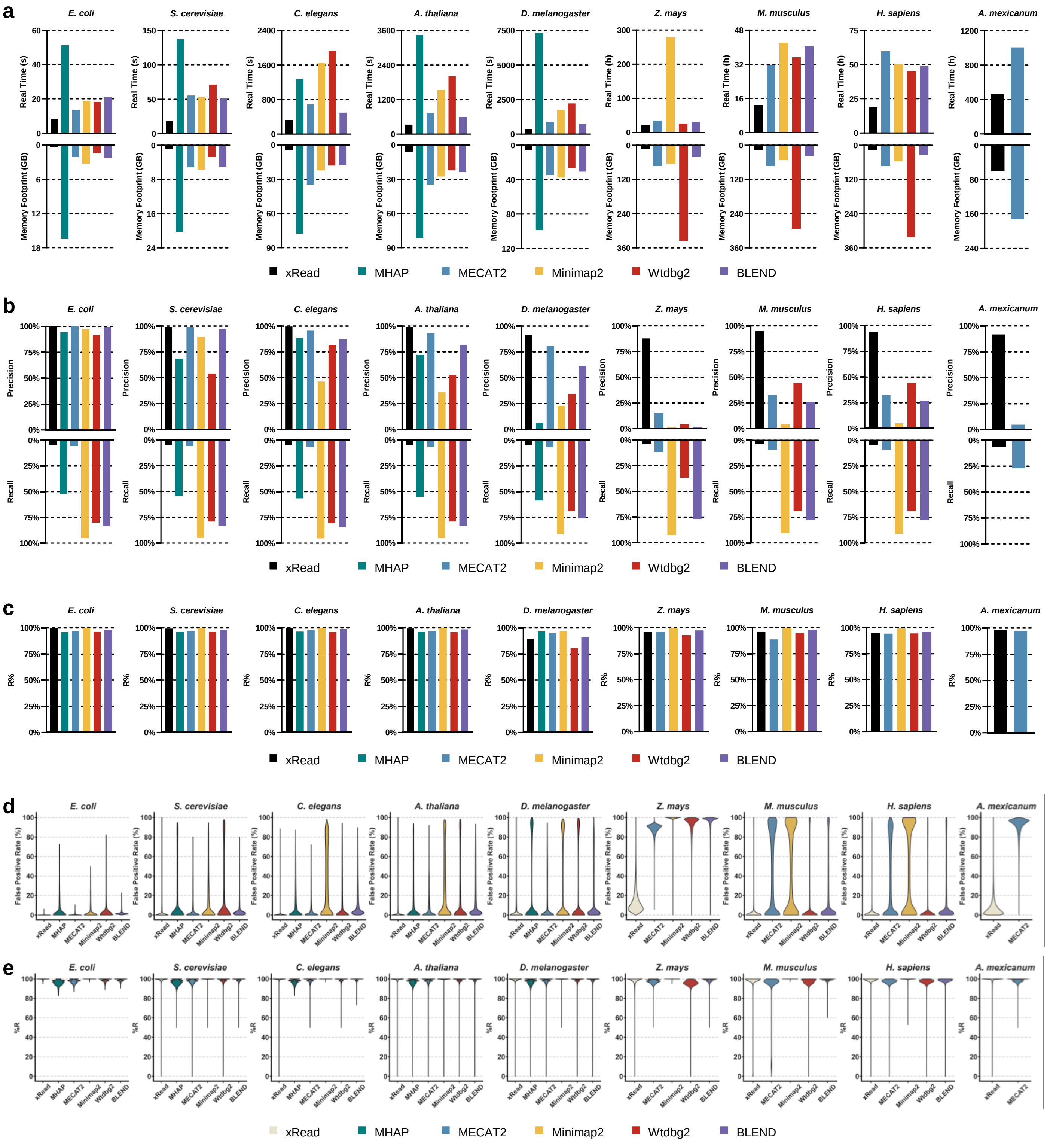

Figure 3 Click here to access/download;Figure;fig3.pdf

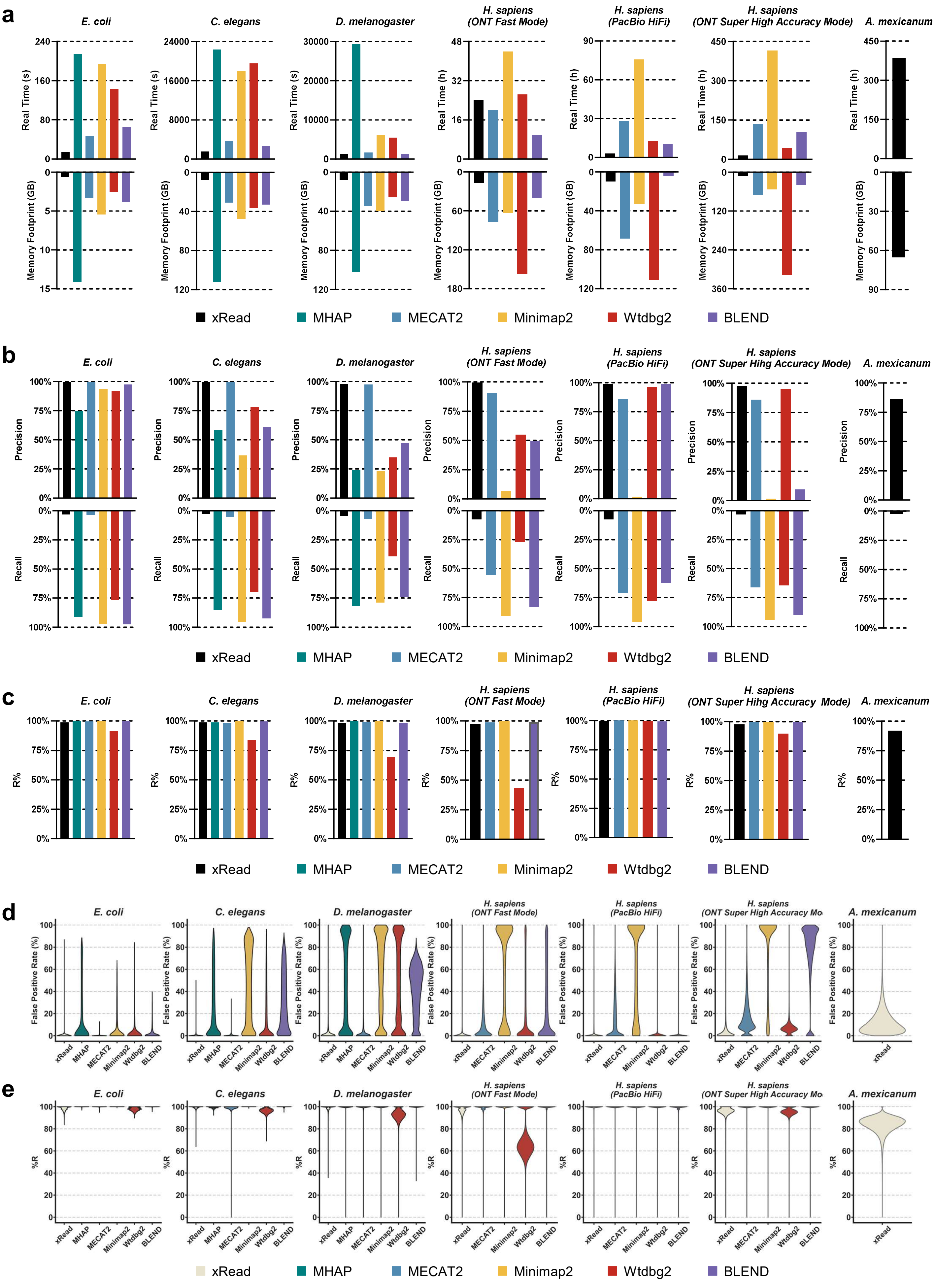

Click here to access/download
Supplementary Material
xRead Supplementary Material.docx
