# Supplementary material for: xRead: a coverage-guided approach for scalable construction of read overlapping graph
Source: Gigascience. 2025 Feb 17;14:giaf007. doi: 10.1093/gigascience/giaf007 (PMC11831799; doi:10.1093/gigascience/giaf007)
Supplement: giaf007_Response_to_Reviewer_Comments_Original_Submission [file giaf007_response_to_reviewer_comments_original_submission.pdf]

Author's Response To Reviewer Comments

We appreciate the valuable comments and suggestions from the editorial board members and the reviewers. We have made a precise point-to-point response to all the raised comments. Due to the lack of enough space here, we have added the response letter in the attachment. Please refer to it (see below, before the main text of the revised manuscript).
