# Supplementary material for: xRead: a coverage-guided approach for scalable construction of read overlapping graph
Source: Gigascience. 2025 Feb 17;14:giaf007. doi: 10.1093/gigascience/giaf007 (PMC11831799; doi:10.1093/gigascience/giaf007)
Supplement: giaf007_Response_to_Reviewer_Comments_Revision_1 [file giaf007_response_to_reviewer_comments_revision_1.pdf]

Author's Response To Reviewer Comments

Dear Hans,
Thank you for your email and for accepting our manuscript “xRead: a coverage-guided approach for scalable construction of read overlapping graph" (GIGA-D-24-00195R1) for publication in GigaScience.
We have addressed all the editorial points as requested:
1. The Software Heritage link has been added to the references and cited in the Data Availability section as instructed.
2. All other URLs have been incorporated into the references and appropriately cited in the Data Availability section.
3. ORCIDs for all authors have been added to the title page, following their names.
4. We have checked the grammar throughout the manuscript, with particular attention to the abstract.
5. We have reviewed the journal's requirements for each section of the manuscript to ensure that every section complies accordingly.
The revised manuscript has been uploaded to the Editorial Manager system for your review. Thank you for your guidance throughout the process. We look forward to the next steps in the publication of our work.
Best wishes,
Bo Liu
