# Supplementary material for: xRead: a coverage-guided approach for scalable construction of read overlapping graph
Source: Gigascience. 2025 Feb 17;14:giaf007. doi: 10.1093/gigascience/giaf007 (PMC11831799; doi:10.1093/gigascience/giaf007)
Supplement: giaf007_Reviewer_1_Report_Original_Submission — Antoine Limasset, Ph.D -- 7/2/2024 [file giaf007_reviewer_1_report_original_submission.pdf]

Reviewer Report

Title: xRead: a coverage-guided approach for scalable construction of read overlapping graph

Version: Original Submission **Date:** 7/2/2024

Reviewer name: Antoine Limasset, Ph.D

Reviewer Comments to Author:

The manuscript describes xreads, a novel method that enables resource-efficient overlap graph computation based on new strategies to compute it quickly and with controlled memory usage. The authors introduce several quality metrics to assess the quality of the overlap graph and integrate their tool into NextDenovo, improving its resource usage.The manuscript is overall clear, although the section order can make it hard to read as concepts are defined backward. Some typos and minor phrasing issues should be corrected.Remarks:The manuscript spends a lot of time evaluating the quality of the overlap graph, which is a very commendable approach and is often overlooked. I thank the authors for this contribution. However, I have issues with the definition of ground truth overlap. Even if two reads do not come from successive parts of the genome, if they share, let's say, a very large perfect overlap, they should indeed overlap in the graph. Considering that the actual biological overlap is necessarily the best one found in the reads is a greedy strategy that could harm the final assembly. Because of this definition, I am not fully convinced by xreads' performance, which seems to employ an overall very greedy strategy.A key selling point of the abstract is the ability of xreads to work with controlled memory usage at the expense of time and external memory usage. Showing some results on this feature would be very interesting, such as a plot showing the time performance depending on memory usage, for example. Also, the amount of external memory used should be discussed.As far as I understand, the end goal of xreads is to perform efficient de novo assembly. The assembly results should be the primary results of the manuscript and not relegated to the supplementary section. The assembly benchmark should include other assemblers and not only NextDenovo. The assembly results and justification are not quite convincing since the proposed assembler is slightly more resource-efficient at the cost of degraded assembly quality. While the case studies are interesting, it is hard to avoid concluding that the overall quality is degraded compared to regular NextDenovo.

Level of Interest

Please indicate how interesting you found the manuscript: Choose an item.

Quality of Written English

Please indicate the quality of language in the manuscript: Choose an item.

Declaration of Competing Interests

Please complete a declaration of competing interests, considering the following questions:

- Have you in the past five years received reimbursements, fees, funding, or salary from an organisation that may in any way gain or lose financially from the publication of this manuscript, either now or in the future?
- Do you hold any stocks or shares in an organisation that may in any way gain or lose financially from the publication of this manuscript, either now or in the future?
- Do you hold or are you currently applying for any patents relating to the content of the manuscript?
- Have you received reimbursements, fees, funding, or salary from an organization that holds or has applied for patents relating to the content of the manuscript?
- Do you have any other financial competing interests?
- Do you have any non-financial competing interests in relation to this paper?

If you can answer no to all of the above, write 'I declare that I have no competing interests' below. If your reply is yes to any, please give details below.

I declare that I have no competing interests

I agree to the open peer review policy of the journal. I understand that my name will be included on my report to the authors and, if the manuscript is accepted for publication, my named report including any attachments I upload will be posted on the website along with the authors' responses. I agree for my report to be made available under an Open Access Creative Commons CC-BY license (http://creativecommons.org/licenses/by/4.0/). I understand that any comments which I do not wish to be included in my named report can be included as confidential comments to the editors, which will not be published.

Choose an item.

To further support our reviewers, we have joined with Publons, where you can gain additional credit to further highlight your hard work (see: https://publons.com/journal/530/gigascience). On publication of this paper, your review will be automatically added to Publons, you can then choose whether or not to claim your Publons credit. I understand this statement.

Yes Choose an item.
