# Supplementary material for: xRead: a coverage-guided approach for scalable construction of read overlapping graph
Source: Gigascience. 2025 Feb 17;14:giaf007. doi: 10.1093/gigascience/giaf007 (PMC11831799; doi:10.1093/gigascience/giaf007)
Supplement: giaf007_Reviewer_2_Report_Original_Submission — Anuradha Wickramarachchi -- 8/13/2024 [file giaf007_reviewer_2_report_original_submission.pdf]

Reviewer Report

Title: xRead: a coverage-guided approach for scalable construction of read overlapping graph

Version: Original Submission **Date:** 8/13/2024

Reviewer name: Anuradha Wickramarachchi

Reviewer Comments to Author:

Overall comments.---------------------Authors of the manuscript have developed an iterative overlap graph construction algorithm to support genome assembly. This is both an interesting and a demanding area of research due to very recent advancements in sequencing technologies.Although the text in the manuscript is interesting, grammar must be rechecked and revised. At some point it is difficult to keep track of the content and references to supplementary to make sense out of the content.Specific comments---------------------Page 1 Line 13: I believe the authors are talking about assembly sizes and not genome sizes. The sentences here could be a bit short to make them easy to understand.Page 2 Line 19: Theoretical time complexity O(m2n2) is bit of an overstatement due to the heuristics employed by most assemblers. For example, mash distance, minimisers and k-mer bins are there to prevent this explosion of complexity. Either acknowledge such methods or provide a range for the time complexity. I would be interesting to know the time complexities of the methods expressed in sentence starting Line 15.Page 5 Line 11: Was this performed with overlapping windows of 1gb? Otherwise, simulations may not have reads spanning across such regions.Page 5 Line 14: It seems you are simulating 9 + 4 + 4 datasets. This is unclear, please make this into bullet points or separate paragraphs and explain clearly. Include simulator information in the table itself by may be making it landscape (in supplementary).Fig 2: I believe authors should expand their analysis to more recent and popular assemblers. For example, wtdbg2 is designed for noisy reads and not specifically for more accurate R10/ HiFi reads. So please include, HiFi-asm, Flye where appropriate. Flye supports ONT out of the box and in my experience does produce good assemblies.Although, you are evaluating read overlaps, it is hard to ignore assemblers themselves just because they do not produce intermediate overlaps graphs.Page 5-9: In the benchmarks section, please include how True Positives and False Positives were labelled. Was this from simulation data?Page 11: Use of xRead has been evaluated on genome assemblies. This is a very important and it is a bit unfortunate that existing assemblers are not very flexible in terms of plugging in new intermediate steps. It might be worth exploring into creating a new assembler using the wtpoa2 cli command of wtdbg2.Page 16: What will happen if you only capture reads from a single chromosome due to longer length? I believe the objective is to gather longest reads capturing as much as possible covering the whole genome. Please comment on this.Page 19: In the Github Readme the download URL was wrong. Please correct it to the latest releaseCorrect: https://github.com/tcKong47/xRead/releases/download/xRead-v1.0.0.1/xRead-v1.0.0.tar.gzExisting: https://github.com/tcKong47/xRead/releases/download/v1.0.0/xRead-v1.0.0.tar.gzMake command failed withmake: *** No rule to make target `main.h', needed by `main.o'. Stop.It seems the release does not have source code, but rather the compiled version. Please update github instructing how to compile code properly with a git clone.

Level of Interest

Please indicate how interesting you found the manuscript: Choose an item.

Quality of Written English

Please indicate the quality of language in the manuscript: Choose an item.

Declaration of Competing Interests

Please complete a declaration of competing interests, considering the following questions:

- Have you in the past five years received reimbursements, fees, funding, or salary from an organisation that may in any way gain or lose financially from the publication of this manuscript, either now or in the future?
- Do you hold any stocks or shares in an organisation that may in any way gain or lose financially from the publication of this manuscript, either now or in the future?
- Do you hold or are you currently applying for any patents relating to the content of the manuscript?
- Have you received reimbursements, fees, funding, or salary from an organization that holds or has applied for patents relating to the content of the manuscript?
- Do you have any other financial competing interests?
- Do you have any non-financial competing interests in relation to this paper?

If you can answer no to all of the above, write 'I declare that I have no competing interests' below. If your reply is yes to any, please give details below.

I declare that I have no competing interests

I agree to the open peer review policy of the journal. I understand that my name will be included on my report to the authors and, if the manuscript is accepted for publication, my named report including any attachments I upload will be posted on the website along with the authors' responses. I agree for my report to be made available under an Open Access Creative Commons CC-BY license (http://creativecommons.org/licenses/by/4.0/). I understand that any comments which I do not wish to be included in my named report can be included as confidential comments to the editors, which will not be published.

Choose an item.

To further support our reviewers, we have joined with Publons, where you can gain additional credit to further highlight your hard work (see: https://publons.com/journal/530/gigascience). On publication of

this paper, your review will be automatically added to Publons, you can then choose whether or not to claim your Publons credit. I understand this statement.

Yes Choose an item.
